# Agreement between the Cochrane risk of bias tool and Physiotherapy Evidence Database (PEDro) scale: A meta-epidemiological study of randomized controlled trials of physical therapy interventions

**DOI:** 10.1371/journal.pone.0222770

**Published:** 2019-09-19

**Authors:** Anne M. Moseley, Prinon Rahman, George A. Wells, Joshua R. Zadro, Catherine Sherrington, Karine Toupin-April, Lucie Brosseau

**Affiliations:** 1 Institute for Musculoskeletal Health, Sydney School of Public Health, Faculty of Medicine and Health, The University of Sydney, Sydney, New South Wales, Australia; 2 Physiotherapy Program, School of Rehabilitation Sciences, Faculty of Health Sciences, University of Ottawa, Ottawa, Ontario, Canada; 3 School of Epidemiology and Public Health, Faculty of Medicine, University of Ottawa, Ottawa, Ontario, Canada; 4 Children’s Hospital of Eastern Ontario Research Institute, Ottawa, Ontario, Canada; 5 Department of Pediatrics, Faculty of Medicine, University of Ottawa, Ottawa, Ontario, Canada; 6 School of Rehabilitation Sciences, Faculty of Health Sciences, University of Ottawa, Ottawa, Ontario, Canada; Federal Joint Committee, GERMANY

## Abstract

**Background:**

The Cochrane risk of bias (CROB) tool and Physiotherapy Evidence Database (PEDro) scale are used to evaluate risk of bias of randomized controlled trials. We assessed the level of agreement between the instruments.

**Methods:**

We searched the Cochrane Library to identify trials included in systematic reviews evaluating physical therapy interventions. For trials that met our inclusion criteria (primary reference in Cochrane review, review used CROB (2008 version), indexed in PEDro), CROB items were extracted from the reviews and PEDro items and total score were downloaded from PEDro. Kappa statistics were used to determine the agreement between CROB and PEDro scale items that evaluate similar constructs (e.g., randomization). The total PEDro score was compared to the CROB summary score (% of items met) using an Intraclass Correlation Coefficient. Sensitivity analyses explored the impact of the CROB “unclear” category and variants of CROB blinding items. Kappa statistics were used to determine agreement between different thresholds for “acceptable” risk of bias between CROB and PEDro scale summary scores.

**Results:**

We included 1442 trials from 108 Cochrane reviews. Agreement was “moderate” for three of the six CROB and PEDro scale items that evaluate similar constructs (allocation concealment, participant blinding, assessor blinding; Kappa = 0.479–0.582). Agreement between the summary scores was “poor” (Intraclass Correlation Coefficient = 0.285). Agreement was highest when the CROB “unclear” category was collapsed with “high” and when participant, personnel and assessor blinding were evaluated separately in CROB. Agreement for different thresholds for “acceptable” risk of bias between CROB and PEDro summary scores was, at best, “fair”.

**Conclusion:**

There was moderate agreement for half of the PEDro and CROB items that evaluate similar constructs. Interpretation of the CROB “unclear” category and variants of the CROB blinding items substantially influenced agreement. Either instrument can be used to quantify risk of bias, but they can’t be used interchangeably.

## Introduction

Evidence-based practice is essential for health providers because it guides the adoption of effective interventions while eliminating those that are less effective or harmful [1]. Randomized controlled trials are recognized as the best study design to examine the effects of an intervention [1, 2]. Critical appraisal of trial risk of bias (methodological quality) is used to confirm that the findings and conclusions are valid, and is one of the five steps of the evidence-based practice process. Two commonly employed instruments used to assess the risk of bias of trials of physical therapy interventions are the Cochrane risk of bias (CROB) tool [3] and the Physiotherapy Evidence Database (PEDro) scale [4].

The Cochrane Collaboration started using the CROB tool in 2008 to assess and report risk of bias in trials included in Cochrane reviews [3]. The CROB tool evaluates potential bias for seven items across six domains: *selection bias (random sequence generation; allocation concealment)*, *performance bias (blinding of participants and personnel)*, *detection bias (blinding of outcome assessment)*, *attrition bias (incomplete outcome data)*, *reporting bias (selective reporting)*, and *other sources of bias*. Each domain (item) is rated as “high,” “unclear” or “low” risk of bias, and are reported separately (a summary score is not calculated). All items evaluate risk of bias. Clinimetric evaluation of the CROB tool has focused on reliability, suggesting that inter-rater agreement for individual items varies from “poor” (Kappa = -0.04 for ‘other bias’) to “substantial” (Kappa = 0.79 for ‘sequence generation) [5–7]. Inter-rater agreement for inexperienced raters with minimal training (Kappa = 0.00 to 0.38) can, however, be improved with standardized training (Kappa = 0.93 to 1.00) [8].

The PEDro scale was developed in 1999 to evaluate the risk of bias and completeness of statistical reporting of trial reports indexed in the PEDro evidence resource [4] and is now commonly used in systematic reviews [9]. This scale evaluates 11 items: *inclusion criteria and source*, *random allocation*, *concealed allocation*, *similarity at baseline*, *subject blinding*, *therapist blinding*, *assessor blinding*, *completeness of follow up*, *intention-to-treat analysis*, *between-group statistical comparisons*, and *point measures and variability*. Each item is rated as “yes” or “no,” and the total PEDro score is the number of items met (excluding the *inclusion criteria and source* item). Eight items evaluate risk of bias (*random allocation*, *concealed allocation*, *similarity at baseline*, *subject blinding*, *therapist blinding*, *assessor blinding*, *completeness of follow up*, *intention-to-treat analysis*) and two items evaluate the completeness of statistical reporting (*between-group statistical comparisons*, and *point measures and variability*). Evaluation of the clinimetric properties of the PEDro scale reveals acceptable validity and reliability. There is evidence for convergent and construct validity for eight out of 11 individual items [10] and acceptably high reliability for the total PEDro score (Interclass Correlation Coefficient = 0.56 to 0.91) [4, 11–13]and individual items (Kappa = 0.45 to 1.00) [4, 12, 14, 15]. Rasch analysis suggests that the PEDro scale can be used as a continuous scale [16].

There is recent evidence of convergent validity between the PEDro scale and the Cochrane Back and Neck Group risk of bias tool (which includes the seven items in the CROB tool plus intention-to-treat analysis, group similarity at baseline, co-interventions, compliance, and timing of outcome assessments) for pharmacological trials (Intraclass Correlation Coefficient = 0.83, 95% confidence interval (CI) 0.76 to 0.88) [17]. However, no research group has studied the convergent validity between the PEDro scale and CROB tool in trials evaluating the effects of physical therapy interventions. This would be interesting as trials evaluating physical therapy interventions, like exercise, do not have the same characteristics as pharmacological trials. Blinding participants and personnel in trials of complex physical therapy interventions is difficult and, usually, not possible [6, 12]. As both the PEDro scale and CROB tool are commonly used in systematic reviews, evaluation of the convergent validity between these instruments would assist clinicians to understand risk of bias across the review articles they read (i.e., do the tools have a similar interpretation and can they be used interchangeably) and possibly provide some guidance for systematic reviewers when they are selecting a tool to evaluate risk of bias in their reviews.

Although both the PEDro scale and CROB tool have different approaches to assessing risk of bias, they have six items in common (random allocation, concealed allocation, blinding of participants, personnel and assessors, and incomplete outcome data). To date, only one study has made a direct comparison between the PEDro scale and CROB tool in trials of physical therapy interventions [18]. This study found poor agreement between the two instruments [18]. These results do, however, need to be interpreted with caution; there was a small sample size (n = 353), the analysis only considered three CROB items (*random sequence generation*, *allocation concealment*, *assessor blinding*), the CROB tool was assumed to be the gold standard, and the cut-point (i.e., “low” risk of bias for all three CROB items) used for “adequate” risk of bias was not explored. This highlights the need to evaluate agreement between the PEDro scale and CROB tool for the items that evaluate similar constructs. While the Cochrane Methods and Statistical Methods Groups do not recommend the use of summary scores [3], the judicious use of a CROB summary score could facilitate the comparison of the two instruments by allowing agreement to be calculated for overall scores.

The primary objective of this study was to determine the convergent validity (level of agreement) between individual items from the PEDro scale and CROB tool that evaluate similar constructs and for summary scores. Convergent validity, a subtype of construct validity (defined as "studied when the tester has no definite criterion measure of the quality with which he is concerned, and must use indirect measures” [19]), is the degree to which two measures of a construct that theoretically should be related are in fact related [20]. Sensitivity analyses were used to explore the impact of the CROB “unclear” category and variants of CROB blinding items on agreement. The secondary objective was to determine the level of agreement between different thresholds for “acceptable” risk of bias between the summary scores for the CROB tool and PEDro scale. Between-review agreement (inter-rater reliability) for the CROB tool was also evaluated.

## Methods

### Study design

This meta-epidemiological study was conducted using two online public health research databases: Cochrane Library (www.cochranelibrary.com) and PEDro (www.pedro.org.au). The study type was identified as a meta-epidemiological study because we used a systematic review to provide data for methodological analysis and the unit of analysis is at the study level [21]. Ethical approval was obtained from the University of Ottawa ethics board (H12-13-03B). The study did not have a protocol because the PROSPERO registry for systematic reviews only accepts protocols where there is a health-related outcome [22].

### Literature search

Experienced librarians working for the Cochrane Collaboration searched the Cochrane Library to identify systematic reviews of randomized controlled trials evaluating physical therapy interventions that used the CROB tool and were published in the period January 2008 to October 2013. An author (AMM) repeated the search for November 2013 to December 2015; all previously included systematic reviews were updated to the most current version in this second search. The following key words were used: “physical therapy,” “physiotherapy,” “rehabilitation,” “exercise,” “electrophysical agents,” “acupuncture,” “massage,” “transcutaneous electrical stimulation (TENS),” “interferential current,” “ultrasound,” “stretching,” “chest therapy,” “pulmonary rehabilitation,” “manipulative therapy,” “mobilization.”

### Trial selection

The eligibility of retrieved Cochrane systematic reviews was assessed by two trained evaluators (PR, AMM) who independently screened the titles and abstracts and, where necessary, the full-text using pre-determined criteria. The criteria were: (1) examined the efficacy of a physical therapy intervention (i.e., physical rehabilitation, excluding surgical or pharmacological interventions); (2) not withdrawn from the Cochrane Library; and (3) used the 2008 version of the CROB tool [3] to evaluate included studies. Any disagreements were resolved by discussion between the two evaluators that led to consensus.

All references for the included studies in the eligible Cochrane reviews were extracted (citation, digital object identification number and PubMed identification number). These data were compiled into an Excel spreadsheet by one data extractor (AMM) and verified by a second extractor (PR). When there was more than one reference for an included study, only the primary reference was retained. In most cases, the review authors had tagged the primary reference using an asterisk. If the primary reference was not tagged, the data extractors selected the most important reference. Again, any disagreements were resolved by discussion between the two evaluators that led to consensus.

Trials that were not indexed on PEDro because they did not fulfil the PEDro inclusion criteria were excluded. The PEDro inclusion criteria are: involve comparison of at least two interventions, at least one intervention is part of physiotherapy practice, intervention applied to subjects who would receive the intervention as part of physiotherapy practice, random or intended-to-be-random allocation to groups, full paper in a peer-reviewed journal.

When trials were in more than one of the included Cochrane reviews, a trial from one review was randomly selected to be in the data set. The duplicate trials were used to evaluate between-review agreement (inter-rater reliability) for the CROB ratings.

### Data extraction and processing

CROB ratings (“low,” “unclear,” “high”) for *random sequence generation*, *allocation concealment*, *blinding of participants*, *blinding of personnel*, *blinding of outcome assessment*, *incomplete outcome data*, *selective reporting*, and *other sources of bias* for each included trial were extracted from the Cochrane reviews. A number of reporting methods were used for the blinding items in the included reviews: participants and personnel combined, participants only, personnel only, outcome assessment only, outcome assessment for subjective outcomes, outcome assessment for objective outcomes, participants and personnel and outcome assessment combined. Each method was extracted into a separate column in the Excel spreadsheet. Two extractors independently extracted the CROB data for the included trials. Any disagreements were resolved by discussion between the two extractors that led to a consensus.

The CROB summary score was calculated as the number of items with “low” risk of bias divided by the number of core items evaluated in the review, and was expressed as a percentage.

PEDro scale scores (11 individual items and total PEDro score), citation, PubMed identification number, digital object identification number, and PEDro identification number for the primary reference for each included trial were downloaded from the PEDro evidence resource (www.pedro.org.au) and added to the Excel spreadsheet. The digital object identification number, PubMed identification number and citation were used to verify that the PEDro citations matched the citations for included studies extracted from the Cochrane reviews.

PEDro items scored as “yes” (i.e., a positive rating) were recoded as “1” and items scored as “no” (i.e., a negative rating) were recoded as “0”. The total PEDro score is the number of items met, excluding the inclusion criteria and source item, and is expressed as a score ranging from 0 to 10.

### Statistical analysis

The number and percentage of trials rating “yes” for each PEDro scale item and “low,” “unclear” and “high” for each CROB item were tabulated. The mean and standard deviation (SD) for the total PEDro score and CROB summary score were calculated.

#### Agreement between the CROB tool and PEDro scale items

Under the supervision of a senior biostatistician (GAW), Kappa statistics, 95% CI and percent exact agreement were calculated to assess the level of agreement (or convergent validity) between individual items from the PEDro scale and CROB tool that evaluate similar constructs [23]. The items were: PEDro *random allocation* vs. CROB *random sequence generation*; PEDro *concealed allocation* vs. CROB *allocation concealment*; PEDro *subject blinding* vs. CROB *blinding of participants*; PEDro *therapist blinding* vs. CROB *blinding of personnel*; PEDro *assessor blinding* vs. CROB *blinding of outcome assessment*; PEDro *completeness of follow up* vs. CROB *incomplete outcome data*.

For the main analyses, the CROB categories were dichotomized by recoding “low” (i.e., a positive rating) as “1” and “unclear” or “high” (i.e., a negative rating) as “0.” Two sets of pre-specified sensitivity analyses were also performed. The first dichotomized the CROB categories into “low” or “unclear” as “1” and “high” as “0.” The second omitted trials rating “unclear” CROB and recoded “low” as “1” and “high” as “0.”

A second set of sensitivity analyses that included different variants of the CROB blinding items were also performed. PEDro *subject blinding* was compared to three groupings of variants of the CROB *blinding of participants* item. PEDro *therapist blinding* was compared to three groupings of variants of the CROB *blinding of personnel* item. PEDro *assessor blinding* was compared to six groupings of variants of the CROB *blinding of outcome assessment* item. For each of these analyses, the CROB categories were dichotomized by recoding “low” as “1” and “unclear” or “high” as “0.”

#### Agreement between the CROB summary score and total PEDro score

Intraclass Correlation Coefficients (type 1,1) and 95% CI were calculated to determine the level of agreement between the CROB summary score and the total PEDro score. In this analysis, the CROB summary score was calculated as the number of items with “low” risk of bias divided by the number of core items evaluated in the review, and was expressed as a percentage. An additional sensitivity analysis was used to test the impact of the “unclear” option. This sensitivity analysis computed the CROB summary score as the number of items with “low” or “unclear” risk of bias divided by the number of core items evaluated in the review.

#### Agreement between different thresholds for the CROB summary score and total PEDro score

To examine the agreement between different thresholds for “acceptable” risk of bias on the CROB tool and PEDro scale, a Kappa statistic matrix was calculated to determine the level of agreement for 10% increments in the CROB summary score and 1-point increments for the total PEDro score. In this analysis, the CROB summary score was calculated after dichotomizing the CROB categories for individual items into “1” for “low” and “0” for “high” and “unclear” (as per the main analyses for agreement between PEDro and CROB items).

#### Between-review agreement of CROB ratings

When trials were included in more than one Cochrane review, Kappa statistics and 95% CI plus percent exact agreement were calculated for each CROB item to quantify between-review agreement. The raw scores (“low,” “unclear,” and “high”) were used in this analysis. Intraclass Correlation Coefficients (type 1,1) and 95% CI were used to quantify between-review agreement for the CROB summary score.

Stata statistical software (version 12, College Station, Texas) was used to perform the analyses. With the exception of between-review agreement for CROB, all Kappa and Intraclass Correlation Coefficient analyses were clustered by review and used 5000 bootstrap replications to calculate the 95% CIs. The Landis and Koch (1977) criteria were used to interpret all of the Kappa statistics (0.81–1.00 = “almost perfect,” 0.61–0.80 = “substantial,” 0.41–0.60 = “moderate,” 0.21–0.40 = “fair,” 0.00–0.20 = “slight,” and <0.00 = “poor”) [24]. The Fleiss (1986) criteria were used to interpret the Intraclass Correlation Coefficients (>0.75 = “excellent,” 0.40–0.75 = “fair to good,” and <0.40 = "poor”) [25].

## Results

### Included trials

**Fig 1:**
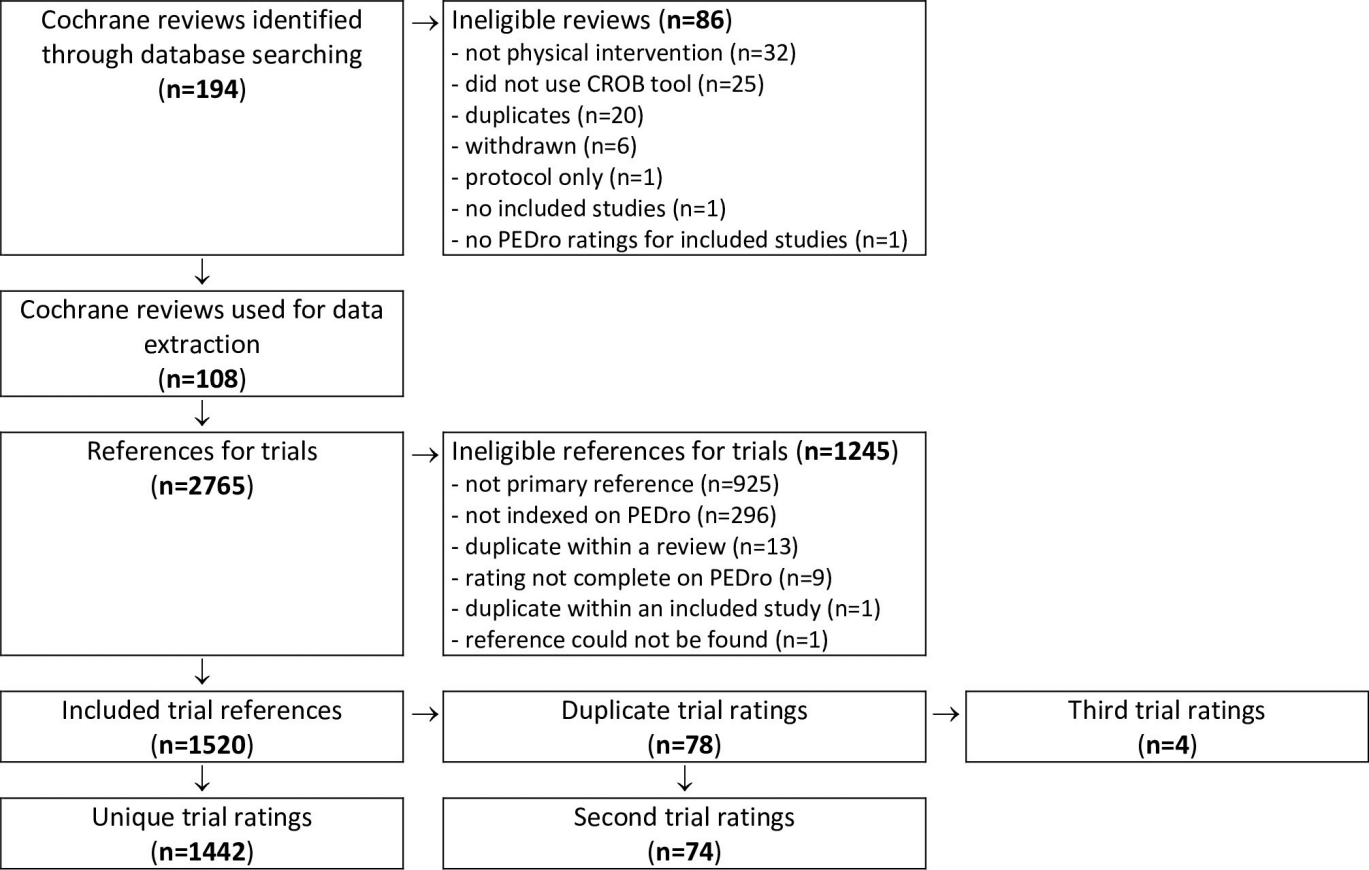
Flow diagram to summarize the process of selecting systematic reviews and trials.

The flow of reviews and trials in this analysis is illustrated in Fig 1. The literature search identified 194 Cochrane systematic reviews that appeared to be related to physical therapy interventions. Of these, 86 reviews were excluded, mostly because they did not evaluate a physical therapy intervention, did not use the 2008 version of the CROB tool, or were duplicates (Fig 1). The 108 eligible Cochrane reviews had a median of 12 included studies each (interquartile range 7; 22) (see S1 File for list of included reviews). The area of practice for the eligible Cochrane reviews was musculoskeletal (27 reviews), cardiorespiratory (20), continence and women's health (14), neurology (8), orthopedics (8), sports (8), oncology (7), endocrine and lifestyle (6), gerontology (5), ergonomics and occupational health (2), pediatrics (2), and mental health (1). The interventions evaluated were exercise (62 reviews), electrotherapy (11), behavioral (7), manual therapy (6), education (5), respiratory therapy (4), acupuncture (2), ergonomics (2), splinting (2), and a combination of the different treatment options (7). There were 2765 references from these included studies. Of these, 1520 were included in this analysis. There were 1442 unique trial ratings that were used in the CROB tool vs. PEDro scale analyses (see S1 File for list of included trials). There were 78 duplicate trial ratings, of which the second trial ratings (n = 74) were used to examine the between-review agreement for the CROB tool and the third trial ratings (n = 4) were excluded from the data set.

The number of trials classified as “low,” “unclear,” and “high” for the CROB items are listed in Table 1. The transformed CROB ratings are in Table 2. For the main analysis, the CROB items with the highest prevalence of having “low” risk of bias were *other source of bias* (56%), *blinding of outcome assessment for objective outcomes* (53%), *incomplete outcome data* (52%), and *random sequence generation* (51%). The mean (SD) CROB summary score was 40.0% (24.4) for the main analysis (i.e., number of items with “low” risk of bias divided by the number of core items evaluated), and 74.4% (19.5) for the sensitivity analysis (i.e., number of items with “low” or “unclear” risk of bias divided by the number of core items evaluated).

**Table 1:** Number and percentage of trials classified as “low”, “unclear” and “high” risk of bias using the Cochrane risk of bias tool.

Cochrane risk of bias tool item	Low risk of bias		Unclear risk of bias		High risk of bias	
	n	%	n	%	n	%
Random sequence generation (n = 1,441)	738	51%	603	42%	100	7%
Allocation concealment (n = 1,438)	517	36%	799	56%	122	8%
Blinding of participants and personnel (n = 601)	82	14%	81	13%	438	73%
Blinding of participants (n = 538)	75	14%	78	14%	385	72%
Blinding of personnel (n = 415)	51	12%	52	13%	312	75%
Blinding of outcome assessment (n = 1,081)	318	29%	375	35%	388	36%
Blinding of outcome assessment for subjective outcomes (n = 82)	20	24%	8	10%	54	66%
Blinding of outcome assessment for objective outcomes (n = 108)	57	53%	33	31%	18	17%
Blinding of participants and personnel and outcome assessment (n = 218)	72	33%	94	43%	52	24%
Incomplete outcome data (n = 1,432)	743	52%	339	24%	350	24%
Selective reporting (n = 1,314)	628	48%	517	39%	169	13%
Other sources of bias (n = 872)	490	56%	273	31%	109	13%

**Table 2:** Transformed Cochrane risk of bias tool ratings, number and percentage of trials coded as “1” for the main and two sensitivity analyses. Data for the main analyses are shaded gray.

Cochrane risk of bias tool item	Main analysis^a^		Sensitivity analysis 1^b^		Sensitivity analysis 2^c^	
	n	%	n	%	n	%
Random sequence generation (n = 1,441)	738	51%	1,341	93%	738	88%
Allocation concealment (n = 1,438)	517	36%	1,316	92%	517	81%
Blinding of participants and personnel combined (n = 601)	82	14%	163	27%	82	16%
Blinding of participants only (n = 538)	75	14%	153	28%	75	16%
Blinding of personnel only (n = 415)	51	12%	103	25%	51	14%
Blinding of outcome assessment only (n = 1,081)	318	29%	693	64%	318	45%
Blinding of outcome assessment for subjective outcomes (n = 82)	20	24%	28	34%	20	27%
Blinding of outcome assessment for objective outcomes (n = 108)	57	53%	90	83%	57	76%
Blinding of participants and personnel and outcome assessment combined (n = 218)	72	33%	166	76%	72	58%
Incomplete outcome data (n = 1,432)	743	52%	1,082	76%	743	68%
Selective reporting (n = 1,314)	628	48%	1,145	87%	628	79%
Other sources of bias (n = 872)	490	56%	763	88%	490	82%

^a^ “1” for “low,” “0” for “unclear’ or “high”

^b^ “1” for “low” or “unclear,” “0” for “high”

^c^ “1” for “low,” “0” for “high,” and “unclear” omitted.

The number of trials scored as “yes” for each PEDro scale item are listed in Table 3. The PEDro scale items with the highest prevalence of being achieved were *random allocation* (97%), *between-group statistical comparisons* (95%), *point measures and variability* (91%), *inclusion criteria and source* (81%), and *baseline comparability* (80%). The mean (SD) total PEDro score was 5.3 (1.6) out of 10.

**Table 3:** Number and percentage of trials achieving each item of the Physiotherapy Evidence Database scale (n = 1442).

Physiotherapy Evidence Database scale item	Trials scored as “yes”	
	n	%
Inclusion criteria and source	1,170	81%
Random allocation	1,399	97%
Concealed allocation	473	33%
Baseline comparability	1,153	80%
Subject blinding	70	5%
Therapist blinding	26	2%
Assessor blinding	529	37%
Completeness of follow up	877	61%
Intention-to-treat analysis	465	32%
Between-group statistical comparisons	1,372	95%
Point measures and variability	1,311	91%

### Agreement between the CROB tool and PEDro scale items

For the main analyses (i.e., dichotomizing the CROB ratings into “1” for “low” and “0” for “unclear” or “high”), the level of agreement between the PEDro and CROB items that evaluate similar constructs ranged from “slight” to “moderate” (see Table 4). Three items were classified as “moderate:” PEDro *concealed allocation* vs. CROB *allocation concealment*, PEDro *assessor blinding* vs. CROB *blinding of outcome assessment*, and PEDro *subject blinding* vs. CROB *blinding of participants* (Kappa = 0.479–0.582). The item with the lowest Kappa value was PEDro *random allocation* vs. CROB *random sequence generation* (Kappa = 0.054). The Kappa values for PEDro *therapist blinding* vs. CROB *blinding of personnel* and PEDro *subject blinding* vs. CROB *blinding of participants* need to be interpreted with caution because of the low base rate of therapist (i.e., 2%, see Table 3) and subject (i.e., 5%, see Table 3) blinding.

**Table 4:** Number of reports, percent exact agreement, Kappa, and Kappa 95% confidence interval for the level of agreement between individual items of the Physiotherapy Evidence Database scale and Cochrane risk of bias tool for the main analysis and two sensitivity analyses. Data for the main analyses are shaded gray.

Items	Main analysis^a^				Sensitivity analysis 1^b^				Sensitivity analysis 2^c^			
	n	Agreement	Kappa	95% CI	n	Agreement	Kappa	95% CI	n	Agreement	Kappa	95% CI
PEDro *random allocation* vs. CROB *random sequence generation*	1441	53.8%	0.054	0.031, 0.082	1441	94.9%	0.467	0.329, 0.595	838	91.9%	0.473	0.329, 0.610
PEDro *concealed allocation* vs. CROB *allocation concealment*	1438	81.2%	0.582	0.495, 0.656	1438	39.9%	0.067	0.042, 0.095	639	73.7%	0.421	0.300, 0.531
PEDro *subject blinding* vs. CROB *blinding of participants*	538	90.7%	0.479	0.250, 0.687	538	76.2%	0.227	0.085, 0.424	460	89.1%	0.471	0.248, 0.680
PEDro *therapist blinding* vs. CROB *blinding of personnel*	415	89.2%	0.251	0.025, 0.537	415	76.6%	0.110	0.000, 0.362	363	87.6%	0.245	0.023, 0.554
PEDro *assessor blinding* vs. CROB *blinding of outcome assessment*	1081	78.8%	0.519	0.427, 0.603	1081	53.4%	0.138	0.047, 0.242	706	74.6%	0.490	0.386, 0.593
PEDro *completeness of follow-up* vs. CROB *incomplete outcome data*	1432	63.0%	0.254	0.191, 0.308	1432	66.1%	0.238	0.171, 0.303	1093	69.1%	0.322	0.254, 0.383

^a^ “1” for “low,” “0” for “unclear’ or “high”

^b^ “1” for “low” or “unclear,” “0” for “high”

^c^ “1” for “low,” “0” for “high,” and “unclear” omitted.

The sensitivity analyses revealed that interpretation of the CROB “unclear” category had a large impact on the agreement values. With the exceptions of the PEDro *random allocation* vs. CROB *random sequence generation* and PEDro *completeness of follow-up* vs. CROB *incomplete outcome data*, Kappa values were lower in the sensitivity analyses (Table 4). Agreement for PEDro *random allocation* vs. CROB *random sequence generation* changed from “slight” in the main analysis to “moderate” in both sensitivity analyses. Agreement for PEDro *completeness of follow-up* vs. CROB *incomplete outcome data* was highest when trials with CROB “unclear” ratings were omitted from the analysis (i.e., sensitivity analysis 2).

The analyses exploring the impact of different groupings of variants of the CROB blinding items on agreement between the PEDro scale and CROB tool are in Table 5. Agreement was highest when blinding was reported separately for the participants (“moderate” agreement) and personnel (“fair” agreement). For example, PEDro *subject blinding* vs. CROB *blinding of participants* had a Kappa value of 0.479 (“moderate” agreement), compared to 0.328 for PEDro *subject blinding* vs. CROB *blinding of participants* + CROB *blinding of participants and personnel combined* + CROB *blinding of participants and personnel and outcome assessment combined* (“fair” agreement). In contrast, collapsing different methods of reporting blinding of outcome assessment was consistent with the main analyses (all classified as “moderate” agreement), with the exception of CROB *blinding of outcome assessment for subjective outcomes* (“slight” agreement) and CROB *blinding of outcome assessment for objective outcomes* (“substantial” agreement).

**Table 5:** Number of trials, percent exact agreement, Kappa, and Kappa 95% CI for the level of agreement between Physiotherapy Evidence Database scale subject blinding, therapist blinding, and assessor blinding and different groupings of the variants of Cochrane risk of bias tool blinding of participants, personnel and outcome assessment items. Note, the gray-shaded rows are repeated from the main analysis in Table 4.

Items	n	Agreement	Kappa	95% CI
**(a) PEDro *subject blinding* vs. groupings of variants of CROB *blinding of participants***				
*blinding of participants*	538	90.7%	0.479	0.250, 0.687
blinding of participants + blinding of participants and personnel combined + blinding of participants, personnel and outcome assessment combined	1356	86.4%	0.328	0.202, 0.458
blinding of participants and personnel combined	601	88.0%	0.254	0.066, 0.522
blinding of participants, personnel and outcome assessment combined	218	71.1%	0.204	-0.007, 0.331
**(b) PEDro *therapist blinding* vs. groupings of variants of CROB *blinding of personnel***				
*blinding of personnel*	415	89.2%	0.251	0.025, 0.537
blinding of personnel + blinding of participants and personnel combined + blinding of participants, personnel and outcome assessment combined	1233	84.6%	0.143	0.041, 0.271
blinding of participants and personnel combined	601	86.7%	0.058	0.000, 0.183
blinding of participants, personnel and outcome assessment combined	218	70.2%	0.134	-0.022, 0.320
**(c) PEDro *assessor blinding* vs. groupings of variants of CROB *blinding of outcome assessment***				
*blinding of outcome assessment*	1081	78.8%	0.519	0.427, 0.603
blinding of outcome assessment + blinding of participant, personnel and outcome assessment combined	1298	79.0%	0.527	0.449, 0.603
blinding of participant, personnel and outcome assessment combined	218	80.3%	0.565	0.411, 0.679
blinding of outcome assessment + blinding of outcome assessment for subjective outcomes + blinding of participants, personnel and outcome assessment combined	1380	77.7%	0.500	0.411, 0.582
blinding of outcome assessment for subjective outcomes	82	56.1%	0.154	-0.017, 0.563
blinding of outcome assessment + blinding of outcome assessment for objective outcomes + blinding of participant, personnel and outcome assessment combined	1406	79.3%	0.542	0.467, 0.612
blinding of outcome assessment for objective outcomes	108	82.4%	0.648	0.072, 0.777

### Agreement between the CROB summary score and total PEDro score

The agreement between the CROB summary score and total PEDro score was “poor” for the main analysis (CROB “unclear” collapsed with “high”), with an Intraclass Correlation Coefficient of 0.285 (95% CI -0.093 to 0.831). Results from the sensitivity analysis (CROB “unclear” collapsed with “low”) was also classified as “poor” agreement (Intraclass Correlation Coefficient = -0.150, 95% CI -0.064 to 0.771).

### Agreement between different thresholds for the CROB summary score and total PEDro score

The matrix of agreement between different thresholds for “acceptable” risk of bias for the CROB summary score and total PEDro score is in Table 6. At best, the Kappa scores could be categorized as “fair” for the total PEDro score thresholds of ≥5 to ≥8 and the CROB thresholds of ≥20% to ≥80%. The highest Kappa value (0.318, 95% CI 0.250 to 0.388) occurred for the total PEDro score threshold of ≥6 and the CROB summary score threshold of ≥50%.

**Table 6:** Matrix of number of trials achieving both the Cochrane risk of bias summary score and total Physiotherapy Evidence Database scale score thresholds for “acceptable” risk of bias (n), percent exact agreement, Kappa (K) and Kappa 95% confidence interval for the level of agreement between different thresholds for “acceptable” risk of bias for the Cochrane risk of bias summary score and total Physiotherapy Evidence Database scale score (N = 1442). Cells are shaded to indicate the degree of agreement: no shading = “poor” or not calculable, gray = “slight,” dark gray = “moderate” [note: no comparisons had “fair,” “substantial” or “almost perfect” agreement].

		Cochrane risk of bias tool summary score (out of 100%)									
		≥10%	≥20%	≥30%	≥40%	≥50%	≥60%	≥70%	≥80%	≥90%	≥100%
**Total** **Physiotherapy Evidence Database scale** **score** **(out** **of 10)**	**≥1**	n = 1310 [90.8%] (*)	n = 1092 [75.7%] (*)	n = 854 [59.2%] (*)	n = 760 [52.7%] (*)	n = 534 [37.0%] (*)	n = 302 [20.9%] (*)	n = 211 [14.6%] (*)	n = 106 [7.4%] (*)	n = 28 [1.9%] (*)	n = 28 [1.9%] (*)
	**≥2**	n = 1303 [90.6%] (K = 0.042; 0.000, 0.090)	n = 1086 [75.7%] (K = 0.013; -0.005, 0.032)	n = 850 [59.4%] (K = 0.009; -0.001, 0.019)	n = 756 [52.9%] (K = 0.005; -0.003, 0.013)	n = 532 [37.5%] (K = 0.005; -0.002, 0.011)	n = 301 [21.6%] (K = 0.002; -0.001, 0.006)	n = 211 [15.4%] (K = 0.003; 0.001, 0.004)	n = 106 [8.1%] (K = 0.001: 0.001, 0.002)	n = 28 [2.7%] (K = 0.000; 0.000, 0.001)	n = 28 [2.7%] (K = 0.000; 0.000, 0.001)
	**≥3**	n = 1274 [89.0%] (K = 0.068; 0.009, 0.131)	n = 1064 [75.0%] (K = 0.037; 0.008, 0.069)	n = 833 [59.5%] (K = 0.021; 0.000, 0.047)	n = 742 [53.4%] (K = 0.018; -0.002, 0.040)	n = 524 [38.8%] (K = 0.016; 0.004, 0.029)	n = 297 [23.4%] (K = 0.008; 0.001, 0.016)	n = 210 [17.7%] (K = 0.010; 0.005, 0.015)	n = 106 [10.5%] (K = 0.005; 0.003, 0.008)	n = 28 [5.1%] (K = 0.001; 0.001, 0.002)	n = 28 [5.1%] (K = 0.001; 0.001, 0.002)
	**≥4**	n = 1191 [85.4%] (K = 0.194; 0.116, 0.267)	n = 1014 [75.9%] (K = 0.196; 0.140, 0.255)	n = 800 [62.8%] (K = 0.130; 0.091, 0.173)	n = 718 [57.9%] (K = 0.121; 0.085, 0.157)	n = 510 [44.7%] (K = 0.080; 0.056, 0.109)	n = 292 [30.6%] (K = 0.044; 0.029, 0.062)	n = 208 [25.2%] (K = 0.036; 0.026, 0.049)	n = 105 [18.2%] (K = 0.018; 0.011, 0.026)	n = 28 [13.0%] (K = 0.005; 0.003, 0.008)	n = 28 [13.0%] (K = 0.005; 0.003, 0.008)
	**≥5**	n = 955 [72.2%] (K = 0.185; 0.109, 0.263)	n = 840 [71.4%] (K = 0.284; 0.218, 0.346)	n = 687 [66.6%] (K = 0.281; 0.226, 0.335)	n = 629 [65.1%] (K = 0.287; 0.235, 0.338)	n = 458 [57.1%] (K = 0.220; 0.171, 0.272)	n = 267 [46.7%] (K = 0.130; 0.094, 0.172)	n = 192 [42.6%] (K = 0.099; 0.070, 0.132)	n = 97 [36.7%] (K = 0.049; 0.030, 0.072)	n = 28 [32.5%] (K = 0.017; 0.010, 0.027)	n = 28 [32.5%] (K = 0.017; 0.010, 0.027)
	**≥6**	n = 622 [51.6%] (K = 0.121; 0.074, 0.176)	n = 579 [60.7%] (K = 0.262; 0.199, 0.329)	n = 492 [65.2%] (K = 0.319; 0.256, 0.383)	n = 456 [66.7%] (K = 0.339; 0.277, 0.398)	n = 345 [67.0%] (K = 0.318; 0.250, 0.388)	n = 218 [65.5%] (K = 0.256; 0.189, 0.332)	n = 165 [64.4%] (K = 0.220; 0.160, 0.287)	n = 85 [60.6%] (K = 0.119; 0.076, 0.174)	n = 28 [58.1%] (K = 0.050; 0.027, 0.079)	n = 28 [58.1%] (K = 0.050; 0.028, 0.079)
	**≥7**	n = 343 [32.9%] (K = 0.061; 0.038, 0.089)	n = 326 [45.7%] (K = 0.145; 0.105, 0.187)	n = 296 [58.0%] (K = 0.235; 0.183, 0.290)	n = 280 [62.3%] (K = 0.268; 0.209, 0.321)	n = 224 [70.2%] (K = 0.311; 0.245, 0.378)	n = 145 [75.4%] (K = 0.292; 0.213, 0.377)	n = 117 [77.8%] (K = 0.295; 0.216, 0.377)	n = 57 [76.8%] (K = 0.160; 0.095, 0.238)	n = 19 [76.9%] (K = 0.069; 0.030, 0.121)	n = 19 [76.9%] (K = 0.069; 0.030, 0.121)
	**≥8**	n = 135 [18.5%] (K = 0.021; 0.013, 0.030)	n = 131 [33.1%] (K = 0.056; 0.040, 0.076)	n = 127 [49.0%] (K = 0.113; 0.083, 0.149)	n = 124 [55.1%] (K = 0.140; 0.103, 0.182)	n = 110 [68.9%] (K = 0.211; 0.156, 0.271)	n = 72 [79.7%] (K = 0.230; 0.164, 0.299)	n = 58 [84.0%] (K = 0.250; 0.174, 0.323)	n = 32 [87.7%] (K = 0.200; 0.124, 0.275)	n = 11 [90.2%] (K = 0.106; 0.037, 0.183)	n = 11 [90.2%] (K = 0.106; 0.038, 0.186)
	**≥9**	n = 11 [9.9%] (K = 0.002; 0.001, 0.003)	n = 11 [25.0%] (K = 0.005; 0.002, 0.009)	n = 11 [41.5%] (K = 0.011; 0.005, 0.018)	n = 10 [47.9%] (K = 0.011; 0.005, 0.019)	n = 10 [63.6%] (K = 0.022; 0.010, 0.038)	n = 6 [79.1%] (K = 0.024; 0.001, 0.051)	n = 6 [85.4%] (K = 0.040; 0.008, 0.077)	n = 4 [92.4%] (K = 0.055; 0.003, 0.117)	n = 1 [97.4%] (K = 0.041; -0.014, 0.157)	n = 1 [97.4%] (K = 0.041; -0.014, 0.160)
	**≥10**	n = 1 [9.2%] (K = 0.000; 0.000, 0.000)	n = 1 [24.3%] (K = 0.000; 0.000, 0.002)	n = 1 [40.8%] (K = 0.001; 0.000, 0.003)	n = 1 [47.4%] (K = 0.001; 0.000, 0.004)	n = 1 [63.0%] (K = 0.002; 0.000, 0.008)	n = 0 [79.0%] (K = -0.001; -0.004, 0.000)	n = 0 [85.3%] (K = -0.001; -0.004, 0.000)	n = 0 [92.6%] (K = -0.001; -0.004, 0.000)	n = 0 [98.0%] (K = -0.001; -0.004, 0.000)	n = 0 [9.2%] (K = 0.000; 0.000, 0.000)

* Kappa values were not calculable.

### Between-review agreement of CROB ratings

The between-review agreement (inter-rater reliability) for individual CROB items are reported in Table 7 (note, agreement values were not calculable for five items because there were too few pairs of trials that had ratings for these items). One item was classified as “almost perfect” (*allocation concealment*, Kappa = 0.818), one as “substantial” (*random sequence generation*, Kappa = 0.686), two as “moderate” (*blinding of outcome assessment*, Kappa = 0.533; *selective reporting*, Kappa = 0.450), two as “fair” (*incomplete outcome data*, Kappa = 0.365; *other sources of bias*, Kappa = 0.314), and one as “poor” (*blinding of participants and personnel*, Kappa = -0.037). The agreement for the CROB summary score was “fair to good,” with an Intraclass Correlation Coefficient of 0.711 (95% CI 0.578 to 0.808).

**Table 7:** Number of reports, percent exact agreement, Kappa, and Kappa 95% confidence interval for the level of between-review agreement for the Cochrane risk of bias ratings (n = 74).

Cochrane risk of bias tool item	N	Agreement	Kappa	95% CI
Allocation concealment	73	90.4%	0.818	0.681, 0.944
Random sequence generation	74	85.1%	0.686	0.517, 0.787
Blinding of outcome assessment	57	70.2%	0.533	0.342, 0.702
Selective reporting	69	66.7%	0.450	0.275, 0.617
Incomplete outcome data	73	61.6%	0.365	0.201, 0.530
Other sources of bias	36	66.7%	0.314	0.059, 0.558
Blinding of participants and personnel	28	89.3%	-0.037	-0.091, 0.000
Blinding of participants	14	92.9%	^a^	^a^
Blinding of personnel	14	100.0%	^a^	^a^
Blinding of outcome assessment for subjective outcomes	0	^a^	^a^	^a^
Blinding of outcome assessment for objective outcomes	0	^a^	^a^	^a^
Blinding of participants and personnel and outcome assessment	0	^a^	^a^	^a^

^a^ not calculable.

## Discussion

There was “moderate” agreement between the PEDro scale and CROB tool for three of the six items that evaluate similar constructs: PEDro *concealed allocation* vs. CROB *allocation concealment*, PEDro *assessor blinding* vs. CROB *blinding of outcome assessment*, and PEDro *subject blinding* vs. CROB *blinding of participants* (Kappa = 0.479–0.582). Agreement was “slight” to “fair” for the other three items, and “poor” for the CROB summary score vs. total PEDro score. Agreement tended to be higher when the CROB “unclear” category was collapsed with “high” and when blinding of participants, personnel and outcome assessment were evaluated separately within the CROB tool. It was not possible to draw a strong conclusion about level of agreement between different thresholds for “acceptable” risk of bias between summary scores from the two instruments.

The main strengths of this meta-epidemiological study were the rigorous methods used for data extraction, large sample size, and comprehensive analysis of convergent validity that included all core items from the CROB tool and PEDro scale. Our sample (1442 trials from 108 reviews) represents a three-fold increase on previous studies [18]. This allowed us to assess the agreement between the instruments and to conduct a series of sensitivity analyses to explore the impact of the CROB “unclear” category and how blinding is quantified in the CROB tool. Calculating summary scores for instruments used to assess the risk of bias of trials is controversial. Critics of summary scores argue that summation is invalid because most instruments are comprised of heterogeneous items evaluating both quality of reporting and the conduct of trials [3, 7, 8, 26–28]. Proponents argue that summing is justified if an instrument has empirical evidence indicating a unidimensional structure [10, 16] and that summary scores facilitate analysis (e.g., being used as an independent variable in meta-regression). Perhaps there is room for some middle ground, with reporting of both individual items and summary scores for risk of bias assessment in systematic reviews and in the evaluation of the measurement properties of risk of bias instruments. Calculation of a summary score allowed us to perform a more rigorous comparison of the PEDro scale and CROB tool, including the systematic analysis of different thresholds for “acceptable” risk of bias.

The low agreement between some items of the CROB tool and PEDro scale could be due to the characteristics of the instruments and raters. Operationalization of some of the items that assess similar constructs differ between the instruments. This is particularly evident in the item with the lowest agreement, PEDro *random allocation* vs. CROB *random sequence generation* (Kappa = 0.054). In this instance, the CROB item is more stringent than the PEDro item, requiring the precise method of sequence generation to be specified. In contrast, agreement was “moderate” for items that had similar definitions (e.g., PEDro *concealed allocation* vs. CROB *allocation concealment*, Kappa = 0.582). Different pairs of raters generated the CROB ratings because they were extracted from Cochrane reviews evaluating physical therapy interventions. While we did not calculate Kappa using an approach that accommodates multiple raters, our agreement estimates are likely to be reasonable [29]. Online training for the 2008 version of the CROB tool is limited and the terminology used in the tool could be difficult for reviewers who do not have clinical epidemiology training; this may lead to increased usage of the “unclear” risk of bias rating [30–32]. While the interpretation of “unclear” is not well explained in the Cochrane handbook [3], we observed that the “unclear” category had a large impact on risk of bias scoring. For example, the CROB summary score was 40.0% when the number of items with “low” risk of bias was divided by the number of core items evaluated and 74.4% when the number of items with “low” or “unclear” risk of bias was divided by the number of core items evaluated. Inclusion of two items that evaluate completeness of statistical reporting in the total PEDro score may have contributed to the poor agreement between the CROB summary score and total PEDro score. However, because nearly all trials achieved the PEDro *between-group statistical comparisons* (95%) and *point measures and variability* (91%) items, the impact is likely to be small.

The focus of this meta-epidemiological study was on trials evaluating physical therapy interventions. These trials differ from pharmacological trials in methodological structure, particularly for blinding of participants (or subjects) and personnel (or therapists) [6, 12]. Blinding of participants and personnel was not possible for the majority of the included trials (5% had subject blinding and 2% had therapist blinding; Table 3) because of the complex nature of the interventions being evaluated, but assessor blinding was achieved in about one-third of trials (37% had assessor blinding; Table 3). This highlights the importance of evaluating blinding separately for subjects, therapists and assessors when evaluating risk of bias in physical therapy trials. While we classified the physical therapy interventions evaluated in the included trials into 10 categories (exercise, electrotherapy etc), we did not perform any subgroup analyses on the blinding items. This could be the focus of future research.

The difficulty in blinding subjects and therapists may make applying the CROB tool more challenging, as evidenced by the included reviews using seven variations of the blinding items. While variation in the implementation of the CROB tool made it difficult to evaluate the blinding items because of incomplete data, agreement between the CROB and PEDro blinding items was highest when blinding was reported separately for the participants (“moderate” agreement) and personnel (“fair” agreement). Variation also occurred for other CROB items, with none of the core items being assessed for all trials (Table 1) and 25 potentially eligible reviews being excluded because they did not use the 2008 version of the CROB tool (Fig 1). The difference in methodological structure and variation in the application of the CROB tool in the included reviews could contribute to the lower agreement between the CROB summary score and total PEDro score observed in our evaluation of physical therapy interventions (i.e., “poor”) compared to pharmacological trials (“strong” convergence) [17]. The observed variability in the implementation of the CROB tool could also add confusion for readers of Cochrane reviews.

Our evaluation of between-review agreement for the CROB tool revealed “almost perfect” agreement for *allocation concealment*, “substantial” for *random sequence generation*, “moderate” for *blinding of outcome assessment* and *selective reporting*, “fair” for *incomplete outcome data* and *other sources of bias*, and “fair to good” for the CROB summary score. With the exception of *incomplete outcome data* and *blinding of participants and personnel*, these levels of agreement were the same or better than the agreement observed between pairs of reviewers reported in the literature [6, 7, 33] and our sample size (n = 74) was larger than other studies [6, 33].

Our analyses have implications for risk of bias assessment in systematic reviews and as a component of evidence-based practice. Researchers and clinicians could use either the CROB tool or the PEDro scale, as neither can be considered the gold standard for risk of bias evaluation. However, the instruments cannot be used interchangeably because of the low convergent validity for the summary scores and some individual items. We were not able to identify a robust threshold for “acceptable” risk of bias and so caution against the use of thresholds for “acceptable” risk of bias for both the CROB tool and PEDro scale. Optimal cut-offs have not been rigorously established for either instrument, and could be the focus of future research.

The 2008 version of the CROB tool was compared to the PEDro scale in this study. The next version of CROB (called ROB 2.0) has recently been released [34], but has not yet been used to evaluate risk of bias in Cochrane reviews. It will take some time for the CROB 2.0 tool to be used in all Cochrane reviews and, because the ROB 2.0 tool will not be applied retrospectively, updated reviews may report risk of bias using the 2008 version of the CROB tool. Future meta-epidemiological studies could compare the two versions of the CROB tool or compare CROB 2.0 to the PEDro scale in order to provide empirical data that can be used to select the most robust risk of bias instrument. Our dataset may be used to facilitate future evaluations (S2 File).

## Conclusion

The agreement between the PEDro scale and CROB tool was “moderate” for three of the six items that evaluate similar constructs. Interpretation of the CROB “unclear” category and variants of the CROB blinding items substantially influenced agreement. We caution against the use of thresholds for “acceptable” risk of bias for both the CROB tool and PEDro scale. Either instrument can be used to quantify risk of bias, but they can’t be used interchangeably.

## Supporting information

S1 File List of included reviews. List of included trials.
(DOCX)

S2 File Full data set. (XLSX)
