# Supplementary material for: Agreement between the Cochrane risk of bias tool and Physiotherapy Evidence Database (PEDro) scale: A meta-epidemiological study of randomized controlled trials of physical therapy interventions
Source: PLoS One. 2019 Sep 19;14(9):e0222770. doi: 10.1371/journal.pone.0222770 (PMC6752782; doi:10.1371/journal.pone.0222770)
Supplement: S1 File — List of included trials. (DOCX) [file pone.0222770.s001.docx]

**Electronic supplement 1**

**List of included reviews**

1. Adie S, Kwan A, Naylor JM, Harris IA, Mittal R. Cryotherapy following total knee replacement. Cochrane Database Syst Rev. 2012; Issue 9:CD007911. doi: 10.1002/14651858.CD007911.pub2.

2. Almeida MO, Silva BNG, Andriolo RB, Atallah Á, Peccin MS. Conservative interventions for treating exercise‐related musculotendinous, ligamentous and osseous groin pain. Cochrane Database Syst Rev. 2013; Issue 6:CD009565. doi: 10.1002/14651858.CD009565.pub2.

3. Amatya B, Khan F, La Mantia L, Demetrios M, Wade DT. Non pharmacological interventions for spasticity in multiple sclerosis. Cochrane Database Syst Rev. 2013; Issue 2:CD009974. doi: 10.1002/14651858.CD009974.pub2.

4. Ammendolia C, Stuber KJ, Rok E, Rampersaud R, Kennedy CA, Pennick V, et al. Nonoperative treatment for lumbar spinal stenosis with neurogenic claudication. Cochrane Database Syst Rev. 2013; Issue 8:CD010712. doi: 10.1002/14651858.CD010712.

5. Amorim Adegboye AR, Linne YM. Diet or exercise, or both, for weight reduction in women after childbirth. Cochrane Database Syst Rev. 2013; Issue 7:CD005627. doi: 10.1002/14651858.CD005627.pub3.

6. Ayeleke RO, Hay‐Smith EJC, Omar MI. Pelvic floor muscle training added to another active treatment versus the same active treatment alone for urinary incontinence in women. Cochrane Database Syst Rev. 2013; Issue 11:CD010551. doi: https://doi.org/10.1002/14651858.CD010551.pub2.

7. Aziz Z, Bell‐Syer SEM. Electromagnetic therapy for treating pressure ulcers. Cochrane Database Syst Rev. 2015; Issue 9:CD002930. doi: 10.1002/14651858.CD002930.pub6.

8. Baker PRA, Francis DP, Soares J, Weightman AL, Foster C. Community wide interventions for increasing physical activity. Cochrane Database Syst Rev. 2015; Issue 1:CD008366. doi: 10.1002/14651858.CD008366.pub3.

9. Beggs S, Foong YC, Le HCT, Noor D, Wood‐Baker R, Walters JAE. Swimming training for asthma in children and adolescents aged 18 years and under. Cochrane Database Syst Rev. 2013; Issue 4:CD009607. doi: 10.1002/14651858.CD009607.pub2.

10. Berghmans B, Hendriks E, Bernards A, de Bie R, Omar MI. Electrical stimulation with non‐implanted electrodes for urinary incontinence in men. Cochrane Database Syst Rev. 2013; Issue 6:CD001202. doi: 10.1002/14651858.CD001202.pub5.

11. Berlowitz DJ, Tamplin J. Respiratory muscle training for cervical spinal cord injury. Cochrane Database Syst Rev. 2013; Issue 7:CD008507. doi: 10.1002/14651858.CD008507.pub2.

12. Bleakley C, McDonough S, Gardner E, Baxter GD, Hopkins JT, Davison GW. Cold‐water immersion (cryotherapy) for preventing and treating muscle soreness after exercise. Cochrane Database Syst Rev. 2012; Issue 2:CD008262. doi: 10.1002/14651858.CD008262.pub2.

13. Bourke L, Homer KE, Thaha MA, Steed L, Rosario DJ, Robb KA, et al. Interventions for promoting habitual exercise in people living with and beyond cancer. Cochrane Database Syst Rev. 2013; Issue 9:CD010192. doi: 10.1002/14651858.CD010192.pub2.

14. Boyle R, Hay‐Smith EJC, Cody JD, Mørkved S. Pelvic floor muscle training for prevention and treatment of urinary and faecal incontinence in antenatal and postnatal women. Cochrane Database Syst Rev. 2012; Issue 10:CD007471. doi: 10.1002/14651858.CD007471.pub2.

15. Braam KI, van der Torre P, Takken T, Veening MA, van Dulmen‐den Broeder E, Kaspers GJL. Physical exercise training interventions for children and young adults during and after treatment for childhood cancer. Cochrane Database Syst Rev. 2013; Issue 4:CD008796. doi: 10.1002/14651858.CD008796.pub2.

16. Bradt J, Goodill SW, Dileo C. Dance/movement therapy for improving psychological and physical outcomes in cancer patients. Cochrane Database Syst Rev. 2011; Issue 10:CD007103. doi: 10.1002/14651858.CD007103.pub2.

17. Busch AJ, Webber SC, Richards RS, Bidonde J, Schachter CL, Schafer LA, et al. Resistance exercise training for fibromyalgia. Cochrane Database Syst Rev. 2013; Issue 12:CD010884. doi: 10.1002/14651858.CD010884.

18. Caliandro P, La Torre G, Padua R, Giannini F, Padua L. Treatment for ulnar neuropathy at the elbow. Cochrane Database Syst Rev. 2012; Issue 7:CD006839. doi: 10.1002/14651858.CD006839.pub3.

19. Campbell SE, Glazener CMA, Hunter KF, Cody JD, Moore KN. Conservative management for postprostatectomy urinary incontinence. Cochrane Database Syst Rev. 2012; Issue 1:CD001843. doi: 10.1002/14651858.CD001843.pub4.

20. Carson KV, Chandratilleke MG, Picot J, Brinn MP, Esterman AJ, Smith BJ. Physical training for asthma. Cochrane Database Syst Rev. 2013; Issue 9:CD001116. doi: 10.1002/14651858.CD001116.pub4.

21. Carvalho APV, Vital FMR, Soares BGO. Exercise interventions for shoulder dysfunction in patients treated for head and neck cancer. Cochrane Database Syst Rev. 2012; Issue 4:CD008693. doi: 10.1002/14651858.CD008693.pub2.

22. Cavalheri V, Tahirah F, Nonoyama ML, Jenkins S, Hill K. Exercise training undertaken by people within 12 months of lung resection for non‐small cell lung cancer. Cochrane Database Syst Rev. 2013; Issue 7:CD009955. doi: 10.1002/14651858.CD009955.pub2.

23. Chalmer J, Blakeway M, Adams Z, Milan SJ. Conservative interventions for treating hyperextension injuries of the proximal interphalangeal joints of the fingers. Cochrane Database Syst Rev. 2013; Issue 2:CD009030. doi: 10.1002/14651858.CD009030.pub2.

24. Choi BKL, Verbeek JH, Tam WWS, Jiang JY. Exercises for prevention of recurrences of low‐back pain. Cochrane Database Syst Rev. 2010; Issue 1:CD006555. doi: 10.1002/14651858.CD006555.pub2.

25. Coggrave M, Norton C, Cody JD. Management of faecal incontinence and constipation in adults with central neurological diseases. Cochrane Database Syst Rev. 2014; Issue 1:CD002115. doi: 10.1002/14651858.CD002115.pub5.

26. Cooney GM, Dwan K, Greig CA, Lawlor DA, Rimer J, Waugh FR, et al. Exercise for depression. Cochrane Database Syst Rev. 2013; Issue 9:CD004366. doi: 10.1002/14651858.CD004366.pub6.

27. Cox NS, Alison JA, Holland AE. Interventions for promoting physical activity in people with cystic fibrosis. Cochrane Database Syst Rev. 2013; Issue 12:CD009448. doi: 10.1002/14651858.CD009448.pub2.

28. Crocker T, Forster A, Young J, Brown L, Ozer S, Smith J, et al. Physical rehabilitation for older people in long-term care. Cochrane Database Syst Rev. 2013; Issue 2:CD004294. doi: 10.1002/14651858.CD004294.pub3.

29. Dahm KT, Brurberg KG, Jamtvedt G, Hagen KB. Advice to rest in bed versus advice to stay active for acute low‐back pain and sciatica. Cochrane Database Syst Rev. 2010; Issue 6:CD007612. doi: 10.1002/14651858.CD007612.pub2.

30. Dal Bello‐Haas V, Florence JM. Therapeutic exercise for people with amyotrophic lateral sclerosis or motor neuron disease. Cochrane Database Syst Rev. 2013; Issue 5:CD005229. doi: 10.1002/14651858.CD005229.pub3.

31. de Vries JS, Krips R, Sierevelt IN, Blankevoort L, van Dijk CN. Interventions for treating chronic ankle instability. Cochrane Database Syst Rev. 2011; Issue 8:CD004124. doi: 10.1002/14651858.CD004124.pub3.

32. Deare JC, Zheng Z, Xue CCL, Liu JP, Shang J, Scott SW, et al. Acupuncture for treating fibromyalgia. Cochrane Database Syst Rev. 2013; Issue 5:CD007070. doi: 10.1002/14651858.CD007070.pub2.

33. do Nascimento Junior P, Módolo NSP, Andrade S, Guimarães MMF, Braz LG, El Dib R. Incentive spirometry for prevention of postoperative pulmonary complications in upper abdominal surgery. Cochrane Database Syst Rev. 2014; Issue 2:CD006058. doi: 10.1002/14651858.CD006058.pub3.

34. Dobbins M, Husson H, DeCorby K, LaRocca RL. School‐based physical activity programs for promoting physical activity and fitness in children and adolescents aged 6 to 18. Cochrane Database Syst Rev. 2013; Issue 2:CD007651. doi: 10.1002/14651858.CD007651.pub2.

35. Dodin S, Blanchet C, Marc I, Ernst E, Wu T, Vaillancourt C, et al. Acupuncture for menopausal hot flushes. Cochrane Database Syst Rev. 2013; Issue 7:CD007410. doi: 10.1002/14651858.CD007410.pub2.

36. Doyle S, Bennett S, Fasoli SE, McKenna KT. Interventions for sensory impairment in the upper limb after stroke. Cochrane Database Syst Rev. 2010; Issue 6:CD006331. doi: 10.1002/14651858.CD006331.pub2.

37. East CE, Begg L, Henshall NE, Marchant PR, Wallace K. Local cooling for relieving pain from perineal trauma sustained during childbirth. Cochrane Database Syst Rev. 2012; Issue 5:CD006304. doi: 10.1002/14651858.CD006304.pub3.

38. Engers AJ, Jellema P, Wensing M, van der Windt D, Grol R, van Tulder MW. Individual patient education for low back pain. Cochrane Database Syst Rev. 2008; Issue 1:CD004057. doi: 10.1002/14651858.CD004057.pub3.

39. Fokkenrood HJP, Bendermacher BLW, Lauret GJ, Willigendael EM, Prins MH, Teijink JAW. Supervised exercise therapy versus non‐supervised exercise therapy for intermittent claudication. Cochrane Database Syst Rev. 2013; Issue 8:CD005263. doi: 10.1002/14651858.CD005263.pub3.

40. Fransen M, McConnell S, Harmer AR, Van der Esch M, Simic M, Bennell KL. Exercise for osteoarthritis of the knee. Cochrane Database Syst Rev. 2015; Issue 1:CD004376. doi: 10.1002/14651858.CD004376.pub3.

41. Fransen M, McConnell S, Hernandez‐Molina G, Reichenbach S. Exercise for osteoarthritis of the hip. Cochrane Database Syst Rev. 2014; Issue 4:CD007912. doi: 10.1002/14651858.CD007912.pub2.

42. Freitas DA, Holloway EA, Bruno SS, Chaves GSS, Fregonezi GAF, Mendonça K. Breathing exercises for adults with asthma. Cochrane Database Syst Rev. 2013; Issue 10:CD001277. doi: 10.1002/14651858.CD001277.pub3.

43. Giangregorio LM, MacIntyre NJ, Thabane L, Skidmore CJ, Papaioannou A. Exercise for improving outcomes after osteoporotic vertebral fracture. Cochrane Database Syst Rev. 2013; Issue 1:CD008618. doi: 10.1002/14651858.CD008618.pub2.

44. Graham N, Gross A, Goldsmith CH, Klaber Moffett J, Haines T, Burnie SJ, et al. Mechanical traction for neck pain with or without radiculopathy. Cochrane Database Syst Rev. 2008; Issue 3:CD006408. doi: 10.1002/14651858.CD006408.pub2.

45. Griffin XL, Smith N, Parsons N, Costa ML. Ultrasound and shockwave therapy for acute fractures in adults. Cochrane Database Syst Rev. 2012; Issue 2:CD008579. doi: 10.1002/14651858.CD008579.pub2.

46. Gross A, Forget M, St George K, Fraser MMH, Graham N, Perry L, et al. Patient education for neck pain. Cochrane Database Syst Rev. 2012; Issue 3:CD005106. doi: 10.1002/14651858.CD005106.pub4.

47. Gross A, Langevin P, Burnie SJ, Bédard‐Brochu MS, Empey B, Dugas E, et al. Manipulation and mobilisation for neck pain contrasted against an inactive control or another active treatment. Cochrane Database Syst Rev. 2015; Issue 9:CD004249. doi: 10.1002/14651858.CD004249.pub4.

48. Hagen S, Stark D. Conservative prevention and management of pelvic organ prolapse in women. Cochrane Database Syst Rev. 2011; Issue 12:CD003882. doi: 10.1002/14651858.CD003882.pub4.

49. Harvey LA, Brosseau L, Herbert RD. Continuous passive motion following total knee arthroplasty in people with arthritis. Cochrane Database Syst Rev. 2014; Issue 2:CD004260. doi: 10.1002/14651858.CD004260.pub3.

50. Hawke F, Chuter V, Walter KEL, Burns J. Non‐drug therapies for lower limb muscle cramps. Cochrane Database Syst Rev. 2012; Issue 1:CD008496. doi: 10.1002/14651858.CD008496.pub2.

51. Hay‐Smith EJC, Herderschee R, Dumoulin C, Herbison GP. Comparisons of approaches to pelvic floor muscle training for urinary incontinence in women. Cochrane Database Syst Rev. 2011; Issue 12:CD009508. doi: 10.1002/14651858.CD009508.

52. Heiwe S, Jacobson SH. Exercise training for adults with chronic kidney disease. Cochrane Database Syst Rev. 2011; Issue 10:CD003236. doi: 10.1002/14651858.CD003236.pub2.

53. Henschke N, Ostelo R, van Tulder MW, Vlaeyen JWS, Morley S, Assendelft WJJ, et al. Behavioural treatment for chronic low‐back pain. Cochrane Database Syst Rev. 2010; Issue 7:CD002014. doi: 10.1002/14651858.CD002014.pub3.

54. Herbert RD, de Noronha M, Kamper SJ. Stretching to prevent or reduce muscle soreness after exercise. Cochrane Database Syst Rev. 2011; Issue 7:CD004577. doi: 10.1002/14651858.CD004577.pub3.

55. Herbison GP, Dean N. Weighted vaginal cones for urinary incontinence. Cochrane Database Syst Rev. 2013; Issue 7:CD002114. doi: 10.1002/14651858.CD002114.pub2.

56. Herderschee R, Hay‐Smith EJC, Herbison GP, Roovers JP, Heineman MJ. Feedback or biofeedback to augment pelvic floor muscle training for urinary incontinence in women. Cochrane Database Syst Rev. 2011; Issue 7:CD009252. doi: 10.1002/14651858.CD009252.

57. Holland AE, Hill CJ, Jones AY, McDonald CF. Breathing exercises for chronic obstructive pulmonary disease. Cochrane Database Syst Rev. 2012; Issue 10:CD008250. doi: 10.1002/14651858.CD008250.pub2.

58. Howe TE, Shea B, Dawson LJ, Downie F, Murray A, Ross C, et al. Exercise for preventing and treating osteoporosis in postmenopausal women. Cochrane Database Syst Rev. 2011; Issue 7:CD000333. doi: 10.1002/14651858.CD000333.pub2.

59. Hulzebos EH, Smit Y, Helders PP, van Meeteren NL. Preoperative physical therapy for elective cardiac surgery patients. Cochrane Database Syst Rev. 2012; Issue 11:CD010118. doi: 10.1002/14651858.CD010118.pub2.

60. Koopman FS, Uegaki K, Gilhus NE, Beelen A, de Visser M, Nollet F. Treatment for postpolio syndrome. Cochrane Database Syst Rev. 2011; Issue 2:CD007818. doi: 10.1002/14651858.CD007818.pub2.

61. Krisanaprakornkit T, Ngamjarus C, Witoonchart C, Piyavhatkul N. Meditation therapies for attention‐deficit/hyperactivity disorder (ADHD). Cochrane Database Syst Rev. 2010; Issue 6:CD006507. doi: 10.1002/14651858.CD006507.pub2.

62. Kroeling P, Gross A, Graham N, Burnie SJ, Szeto G, Goldsmith CH, et al. Electrotherapy for neck pain. Cochrane Database Syst Rev. 2013; Issue 8:CD004251. doi: 10.1002/14651858.CD004251.pub5.

63. Kroon FPB, van der Burg LRA, Buchbinder R, Osborne RH, Johnston RV, Pitt V. Self‐management education programmes for osteoarthritis. Cochrane Database Syst Rev. 2014; Issue 1:CD008963. doi: 10.1002/14651858.CD008963.pub2.

64. Kruis AL, Smidt N, Assendelft WJJ, Gussekloo J, Boland MRS, Rutten‐van Mölken M, et al. Integrated disease management interventions for patients with chronic obstructive pulmonary disease. Cochrane Database Syst Rev. 2013; Issue 10:CD009437. doi: 10.1002/14651858.CD009437.pub2.

65. Lacasse Y, Goldstein R, Lasserson TJ, Martin S. Pulmonary rehabilitation for chronic obstructive pulmonary disease. Cochrane Database Syst Rev. 2006; Issue 4:CD003793. doi: 10.1002/14651858.CD003793.pub2.

66. Lane R, Ellis B, Watson L, Leng GC. Exercise for intermittent claudication. Cochrane Database Syst Rev. 2014; Issue 7:CD000990. doi: 10.1002/14651858.CD000990.pub3.

67. Lin CWC, Donkers NAJ, Refshauge KM, Beckenkamp PR, Khera K, Moseley AM. Rehabilitation for ankle fractures in adults. Cochrane Database Syst Rev. 2012; Issue 11:CD005595. doi: 10.1002/14651858.CD005595.pub3.

68. Mason DL, Dickens VA, Vail A. Rehabilitation for hamstring injuries. Cochrane Database Syst Rev. 2012; Issue 12:CD004575. doi: 10.1002/14651858.CD004575.pub3.

69. Mastellos N, Gunn LH, Felix LM, Car J, Majeed A. Transtheoretical model stages of change for dietary and physical exercise modification in weight loss management for overweight and obese adults. Cochrane Database Syst Rev. 2014; Issue 2:CD008066. doi: 10.1002/14651858.CD008066.pub3.

70. McGregor AH, Probyn K, Cro S, Doré CJ, Burton AK, Balagué F, et al. Rehabilitation following surgery for lumbar spinal stenosis. Cochrane Database Syst Rev. 2013; Issue 12:CD009644. doi: 10.1002/14651858.CD009644.pub2.

71. McNamara RJ, McKeough ZJ, McKenzie DK, Alison JA. Water‐based exercise training for chronic obstructive pulmonary disease. Cochrane Database Syst Rev. 2013; Issue 12:CD008290. doi: 10.1002/14651858.CD008290.pub2.

72. Mishra SI, Scherer RW, Geigle PM, Berlanstein DR, Topaloglu O, Gotay CC, et al. Exercise interventions on health‐related quality of life for cancer survivors. Cochrane Database Syst Rev. 2012; Issue 8:CD007566. doi: 10.1002/14651858.CD007566.pub2.

73. Mishra SI, Scherer RW, Snyder C, Geigle PM, Berlanstein DR, Topaloglu O. Exercise interventions on health‐related quality of life for people with cancer during active treatment. Cochrane Database Syst Rev. 2012; Issue 8:CD008465. doi: 10.1002/14651858.CD008465.pub2.

74. Monaghan B, Caulfield B, O'Mathúna DP. Surface neuromuscular electrical stimulation for quadriceps strengthening pre and post total knee replacement. Cochrane Database Syst Rev. 2010; Issue 1:CD007177. doi: 10.1002/14651858.CD007177.pub2.

75. Muktabhant B, Lumbiganon P, Ngamjarus C, Dowswell T. Interventions for preventing excessive weight gain during pregnancy. Cochrane Database Syst Rev. 2012; Issue 4:CD007145. doi: 10.1002/14651858.CD007145.pub2.

76. O'Connor D, Page MJ, Marshall SC, Massy‐Westropp N. Ergonomic positioning or equipment for treating carpal tunnel syndrome. Cochrane Database Syst Rev. 2012; Issue 1:CD009600. doi: 10.1002/14651858.CD009600.

77. Orozco LJ, Buchleitner AM, Gimenez‐Perez G, Roqué i Figuls M, Richter B, Mauricio D. Exercise or exercise and diet for preventing type 2 diabetes mellitus. Cochrane Database Syst Rev. 2008; Issue 3:CD003054. doi: 10.1002/14651858.CD003054.pub3.

78. Ostelo R, Costa LOP, Maher CG, de Vet HCW, van Tulder MW. Rehabilitation after lumbar disc surgery. Cochrane Database Syst Rev. 2008; Issue 4:CD003007. doi: 10.1002/14651858.CD003007.pub2.

79. Page MJ, Massy‐Westropp N, O'Connor D, Pitt V. Splinting for carpal tunnel syndrome. Cochrane Database Syst Rev. 2012; Issue 7:CD010003. doi: 10.1002/14651858.CD010003.

80. Page MJ, O'Connor D, Pitt V, Massy‐Westropp N. Exercise and mobilisation interventions for carpal tunnel syndrome. Cochrane Database Syst Rev. 2012; Issue 6:CD009899. doi: 10.1002/14651858.CD009899.

81. Page MJ, O'Connor D, Pitt V, Massy‐Westropp N. Therapeutic ultrasound for carpal tunnel syndrome. Cochrane Database Syst Rev. 2013; Issue 3:CD009601. doi: 10.1002/14651858.CD009601.pub2.

82. Patel KC, Gross A, Graham N, Goldsmith CH, Ezzo J, Morien A, et al. Massage for mechanical neck disorders. Cochrane Database Syst Rev. 2012; Issue 9:CD004871. doi: 10.1002/14651858.CD004871.pub4.

83. Peng L, Wang J, Li F. Weight reduction for non‐alcoholic fatty liver disease. Cochrane Database Syst Rev. 2011; Issue 6:CD003619. doi: 10.1002/14651858.CD003619.pub3.

84. Peters S, Page MJ, Coppieters MW, Ross M, Johnston V. Rehabilitation following carpal tunnel release. Cochrane Database Syst Rev. 2013; Issue 6:CD004158. doi: 10.1002/14651858.CD004158.pub2.

85. Price JR, Mitchell E, Tidy E, Hunot V. Cognitive behaviour therapy for chronic fatigue syndrome in adults. Cochrane Database Syst Rev. 2008; Issue 3:CD001027. doi: 10.1002/14651858.CD001027.pub2.

86. Richards J, Hillsdon M, Thorogood M, Foster C. Face-to-face interventions for promoting physical activity. Cochrane Database Syst Rev. 2013; Issue 9:CD010392. doi: 10.1002/14651858.CD010392.pub2.

87. Rubinstein SM, van Middelkoop M, Assendelft WJJ, de Boer MR, van Tulder MW. Spinal manipulative therapy for chronic low‐back pain. Cochrane Database Syst Rev. 2011; Issue 2:CD008112. doi: 10.1002/14651858.CD008112.pub2.

88. Saunders DH, Sanderson M, Brazzelli M, Greig CA, Mead GE. Physical fitness training for stroke patients. Cochrane Database Syst Rev. 2013; Issue 10:CD003316. doi: 10.1002/14651858.CD003316.pub5.

89. Schaafsma FG, Whelan K, van der Beek AJ, van der Es‐Lambeek LC, Ojajärvi A, Verbeek JH. Physical conditioning as part of a return to work strategy to reduce sickness absence for workers with back pain. Cochrane Database Syst Rev. 2013; Issue 8:CD001822. doi: 10.1002/14651858.CD001822.pub3.

90. Silva IS, Fregonezi GAF, Dias FAL, Ribeiro CTD, Guerra RO, Ferreira GMH. Inspiratory muscle training for asthma. Cochrane Database Syst Rev. 2013; Issue 9:CD003792. doi: 10.1002/14651858.CD003792.pub2.

91. Sitjà Rabert M, Rigau Comas D, Fort Vanmeerhaeghe A, Santoyo Medina C, Roqué i Figuls M, Romero‐Rodríguez D, et al. Whole‐body vibration training for patients with neurodegenerative disease. Cochrane Database Syst Rev. 2012; Issue 2:CD009097. doi: 10.1002/14651858.CD009097.pub2.

92. Struik FM, Lacasse Y, Goldstein R, Kerstjens HA, Wijkstra PJ. Nocturnal non‐invasive positive pressure ventilation for stable chronic obstructive pulmonary disease. Cochrane Database Syst Rev. 2013; Issue 6:CD002878. doi: 10.1002/14651858.CD002878.pub2.

93. Taylor RS, Dalal H, Jolly K, Moxham T, Zawada A. Home‐based versus centre‐based cardiac rehabilitation. Cochrane Database Syst Rev. 2010; Issue 1:CD007130. doi: 10.1002/14651858.CD007130.pub2.

94. Teixeira LJ, Valbuza JS, Prado GF. Physical therapy for Bell's palsy (idiopathic facial paralysis). Cochrane Database Syst Rev. 2011; Issue 12:CD006283. doi: 10.1002/14651858.CD006283.pub3.

95. van den Bekerom MPJ, van der Windt D, ter Riet G, van der Heijden GJ, Bouter LM. Therapeutic ultrasound for acute ankle sprains. Cochrane Database Syst Rev. 2011; Issue 6:CD001250. doi: 10.1002/14651858.CD001250.pub2.

96. Verbeek JH, Martimo KP, Karppinen J, Kuijer P, Viikari‐Juntura E, Takala EP. Manual material handling advice and assistive devices for preventing and treating back pain in workers. Cochrane Database Syst Rev. 2011; Issue 6:CD005958. doi: 10.1002/14651858.CD005958.pub3.

97. Virgili G, Rubin G. Orientation and mobility training for adults with low vision. Cochrane Database Syst Rev. 2010; Issue 5:CD003925. doi: 10.1002/14651858.CD003925.pub3.

98. Voet NBM, van der Kooi EL, Riphagen, II, Lindeman E, van Engelen BGM, Geurts ACH. Strength training and aerobic exercise training for muscle disease. Cochrane Database Syst Rev. 2013; Issue 7:CD003907. doi: 10.1002/14651858.CD003907.pub4.

99. Walker BF, French SD, Grant W, Green S. Combined chiropractic interventions for low‐back pain. Cochrane Database Syst Rev. 2010; Issue 4:CD005427. doi: 10.1002/14651858.CD005427.pub2.

100. Walters JAE, Turnock AC, Walters EH, Wood‐Baker R. Action plans with limited patient education only for exacerbations of chronic obstructive pulmonary disease. Cochrane Database Syst Rev. 2010; Issue 5:CD005074. doi: 10.1002/14651858.CD005074.pub3.

101. Wegner I, Widyahening IS, van Tulder MW, Blomberg SEI, de Vet HCW, Brønfort G, et al. Traction for low‐back pain with or without sciatica. Cochrane Database Syst Rev. 2013; Issue 8:CD003010. doi: 10.1002/14651858.CD003010.pub5.

102. Weller CD, Buchbinder R, Johnston RV. Interventions for helping people adhere to compression treatments for venous leg ulceration. Cochrane Database Syst Rev. 2013; Issue 9:CD008378. doi: 10.1002/14651858.CD008378.pub2.

103. Wieland LS, Falzon L, Sciamanna CN, Trudeau KJ, Brodney Folse S, Schwartz JE, et al. Interactive computer‐based interventions for weight loss or weight maintenance in overweight or obese people. Cochrane Database Syst Rev. 2012; Issue 8:CD007675. doi: 10.1002/14651858.CD007675.pub2.

104. Wong CX, Carson KV, Smith BJ. Home care by outreach nursing for chronic obstructive pulmonary disease. Cochrane Database Syst Rev. 2012; Issue 4:CD000994. doi: 10.1002/14651858.CD000994.pub3.

105. Yeung SS, Yeung EW, Gillespie LD. Interventions for preventing lower limb soft‐tissue running injuries. Cochrane Database Syst Rev. 2011; Issue 7:CD001256. doi: 10.1002/14651858.CD001256.pub2.

106. Young J, Angevaren M, Rusted J, Tabet N. Aerobic exercise to improve cognitive function in older people without known cognitive impairment. Cochrane Database Syst Rev. 2015; Issue 4:CD005381. doi: 10.1002/14651858.CD005381.pub4.

107. Yousefi‐Nooraie R, Schonstein E, Heidari K, Rashidian A, Pennick V, Akbari‐Kamrani M, et al. Low level laser therapy for nonspecific low‐back pain. Cochrane Database Syst Rev. 2008; Issue 2:CD005107. doi: 10.1002/14651858.CD005107.pub4.

108. Zainuldin R, Mackey MG, Alison JA. Optimal intensity and type of leg exercise training for people with chronic obstructive pulmonary disease. Cochrane Database Syst Rev. 2011; Issue 11:CD008008. doi: 10.1002/14651858.CD008008.pub2.

**List of included trials**

1. Aalto TJ, Leinonen V, Herno A, Alen M, Kroger H, Turunen V, Savolainen S, Saari T, Airaksinen O. Postoperative rehabilitation does not improve functional outcome in lumbar spinal stenosis: a prospective study with 2-year postoperative follow-up. European Spine Journal 2011 Aug;20(8):1331-1340. doi: 10.1007/s00586-011-1781-y. PMID: 21523459.
2. Abbott JH, Robertson MC, Chapple C, Pinto D, Wright AA, de la Barra SL, Baxter GD, Theis J-C, Campbell AJ, on behalf of the MOA Trial team. Manual therapy, exercise therapy, or both, in addition to usual care, for osteoarthritis of the hip or knee: a randomized controlled trial 1: clinical effectiveness. Osteoarthritis and Cartilage 2013 Apr;21(4):525-534. doi: 10.1016/j.joca.2012.12.014. PMID: 23313532.
3. Ackerman IN, Buchbinder R, Osborne RH. Challenges in evaluating an arthritis self-management program for people with hip and knee osteoarthritis in real-world clinical settings. The Journal of Rheumatology 2012 May;39(5):1047-1055. doi: 10.3899/jrheum.111358. PMID: 22382340.
4. Ada L, Dean CM, Lindley R. Randomized trial of treadmill training to improve walking in community-dwelling people after stroke: the AMBULATE trial. International Journal of Stroke 2013 Aug;8(6):436-444. doi: 10.1111/j.1747-4949.2012.00934.x. PMID: 23464674.
5. Adamsen L, Quist M, Andersen C, Moller T, Herrstedt J, Kronborg D, Baadsgaard MT, Vistisen K, Midtgaard J, Christiansen B, Stage M, Kronborg MT, Rorth M. Effect of a multimodal high intensity exercise intervention in cancer patients undergoing chemotherapy: randomised controlled trial. BMJ 2009 Oct 13;339:b3410. doi: 10.1136/bmj.b3410. PMID: 19826172.
6. Ahamed Y, Macdonald H, Reed K, Naylor P-J, Liu-Ambrase T, McKay H. School-based physical activity does not compromise children's academic performance. Medicine and Science in Sports and Exercise 2007 Feb;39(1):371-376. doi: 10.1249/01.mss.0000241654.45500.8e. PMID: 17277603.
7. Ahl T, Dalen N, Holmberg S, Selvik G. Early weight bearing of displaced ankle fractures. Acta Orthopaedica Scandinavica 1987 Oct;58(5):535-538. PMID: 3425284.
8. Ahl T, Dalen N, Holmberg S, Selvik G. Early weight bearing of malleolar fractures. Acta Orthopaedica Scandinavica 1986 Dec;57(6):526-529. PMID: 3577722.
9. Ahl T, Dalen N, Lundberg A, Bylund C. Early mobilization of operated on ankle fractures. Prospective, controlled study of 40 bimalleolar cases. Acta Orthopaedica Scandinavica 1993 Feb;64(1):95-99. PMID: 8451961.
10. Ahl T, Dalen N, Selvik G. Mobilization after operation of ankle fractures. Good results of early motion and weight bearing. Acta Orthopaedica Scandinavica 1988 Jun;59(3):302-306. PMID: 3132812.
11. Ahmaidi SB, Varray AL, Savy-Pacaux AM, Prefaut CG. Cardiorespiratory fitness evaluation by the shuttle test in asthmatic subjects during aerobic training. Chest 1993 Apr;103(4):1135-1141. doi: 10.1378/chest.103.4.1135. PMID: 8131453.
12. Ahmed MT, Mohammed AH, Amansour A. Effect of pelvic floor electrical stimulation and biofeedback on the recovery of urinary continence after radical prostatectomy. Turkiye Fiziksel Tip ve Rehabilitasyon Dergisi [Turkish Journal of Physical Medicine and Rehabilitation] 2012;58(3):170-176. doi: 10.4274/tftr.66588.
13. Aidar FJ, de Oliveira RJ, Silva AJ, de Matos DG, Mazini Filho ML, Hickner RC, Machado Reis V. The influence of resistance exercise training on the levels of anxiety in ischemic stroke. Stroke Research and Treatment 2012 Nov 11;(298375):Epub. doi: 10.1155/2012/298375. PMID: 23213625.
14. Aidar FJ, Silva AJ, Reis VM, Carneiro A, Carneiro-Cotta S. Estudio de la calidad de vida en el accidente vascular isquemico y su relacion con la actividad fisica (A study on the quality of life in ischaemic vascular accidents and its relation to physical activity) [Spanish]. Revista de Neurologia 2007 Nov 1-15;45(9):518-522. PMID: 17979080.
15. Aiken LS, Butner J, Lockhart CA, Volk-Craft BE, Hamilton G, Williams FG. Outcome evaluation of a randomised trial of the PhoenixCare Intervention: program of case management and coordinated care for the seriously chronically Ill. Journal of Palliative Medicine 2006 Feb;9(1):111-126. doi: 10.1089/jpm.2006.9.111. PMID: 16430351.
16. Aittasalo M, Miilunpalo S, Suni J. The effectiveness of physical activity counseling in a work-site setting: a randomized, controlled trial. Patient Education and Counseling 2004 Nov;55(2):193-202. doi: 10.1016/j.pec.2003.09.003. PMID: 15530754.
17. Akalin E, El O, Peker O, Senocak O, Tamci S, Gulbahar S, Cakmur R, Oncel S. Treatment of carpal tunnel syndrome with nerve and tendon gliding exercises. American Journal of Physical Medicine & Rehabilitation 2002;81(2):108-113. PMID: 11807347.
18. Akiba T, Matsui N, Shinohara S, Fujiwara H, Nomura T, Marumo F. Effects of recombinant human erythropoietin and exercise training on exercise capacity in hemodialysis patients. Artificial Organs 1995 Dec;19(12):1262-1268. doi: 10.1111/j.1525-1594.1995.tb02297.x. PMID: 8967886.
19. Aksac B, Aki S, Karan A, Yalcin O, Isikoglu M, Eskiyurt N. Biofeedback and pelvic floor exercises for the rehabilitation of urinary stress incontinence. Gynecologic and Obstetric Investigation 2003;56(1):23-27. doi: 10.1159/000072327. PMID: 12867764.
20. Alakram P, Puckree T. Effects of electrical stimulation on house-brackmann scores in early Bell's palsy. Physiotherapy Theory and Practice 2010 Apr;26(3):160-166. doi: 10.3109/09593980902886339. PMID: 20331372.
21. Alaranta H, Hurme M, Einola S, Kallio V, Knuts LR, Torma T. Rehabilitation after surgery for lumbar disc herniation: results of a randomized clinical trial. International Journal of Rehabilitation Research 1986;9(3):247-257. PMID: 3536770.
22. Albrecht S, Le Blond R, Kohler V, Cordis R, Gill C, Kleihues H, Schluter S, Noack W. (Cryotherapy as an analgetic technique in direct postoperative management of elective joint replacement) [German]. Zeitschrift fur Orthopadie und Ihre Grenzgebiete 1997;135(1):45-51. doi: 10.1055/s-2008-1039554. PMID: 9199073.
23. Alessi CA, Yoon EJ, Schnelle JF, al-Samarrai NR, Cruise PA. A randomized trial of a combined physical activity and environmental intervention in nursing home residents: do sleep and agitation improve?. Journal of the American Geriatrics Society 1999 Jul;47(7):784-791. PMID: 10404920.
24. Alkire MR, Swank ML. Use of inpatient continuous passive motion versus no CPM in computer-assisted total knee arthroplasty. Orthopaedic Nursing 2010 Jan-Feb;29(1):36-40. doi: 10.1097/NOR.0b013e3181c8ce23. PMID: 20142693.
25. Allen KD, Oddone EZ, Coffman CJ, Datta SK, Juntilla KA, Lindquist JH, Walker TA, Weinberger M, Bosworth HB. Telephone-based self-management of osteoarthritis: a randomized trial. Annals of Internal Medicine 2010 Nov 2;153(9):570-579. doi: 10.1059/0003-4819-153-9-201011020-00006. PMID: 21041576.
26. Althuizen E, van der Wijden CL, van Mechelen W, Seidell JC, van Poppel MNM. The effect of a counselling intervention on weight changes during and after pregnancy: a randomised trial. BJOG 2013 Jan;120(1):92-99. doi: 10.1111/1471-0528.12014. PMID: 23121074.
27. Altmaier EM, Lehmann TR, Russell DW, Weinstein JN, Kao CF. The effectiveness of psychological interventions for the rehabilitation of low back pain: a randomized controlled trial evaluation. Pain 1992 Mar;49(3):329-335. PMID: 1408299.
28. Alves MPT, de Araujo GCS. Low-level laser therapy after carpal tunnel release. Revista Brasileira de Ortopedia 2011 Nov-Dec;46(6):697-701. doi: 10.1590/S0102-36162011000600012.
29. Amundsen T, Weber H, Nordal HJ, Magnaes B, Abdelnoor M, Lilleas F. Lumbar spinal stenosis: conservative or surgical management?: A prospective 10-year study. Spine 2000 Jun 1;25(11):1424-1435. PMID: 10828926.
30. An B, Dai K, Zhu Z, Wang Y, Hao Y, Tang T, Yan H. Baduanjin alleviates the symptoms of knee osteoarthritis. Journal of Alternative & Complementary Medicine 2008 Mar;14(2):167-174. doi: 10.1089/acm.2007.0600. PMID: 18315512.
31. Andersen LL, Kjaer M, Sogaard K, Hansen L, Kryger AI, Sjogaard G. Effect of two contrasting types of physical exercise on chronic neck muscle pain. Arthritis Care & Research 2008 Jan 15;59(1):84-91. doi: 10.1002/art.23256. PMID: 18163419.
32. Andersen LL, Saervoll CA, Mortensen OS, Poulsen OM, Hannerz H, Zebis MK. Effectiveness of small daily amounts of progressive resistance training for frequent neck/shoulder pain: randomised controlled trial. Pain 2011 Feb;152(2):440-446. doi: 10.1016/j.pain.2010.11.016. PMID: 21177034.
33. Andrish JT, Bergfeld JA, Walheim J. A prospective study on the management of shin splints. Journal of Bone and Joint Surgery -- American Volume 1974 Dec;56(8):1697-1700. PMID: 4434039.
34. Angelopoulos PD, Milionis HJ, Grammatikaki E, Moschonis G, Manios Y. Changes in BMI and blood pressure after a school based intervention: the CHILDREN study. European Journal of Public Health 2009 Jun;19(3):319-325. doi: 10.1093/eurpub/ckp004. PMID: 19208697.
35. Aquino RL, Caires PM, Furtado FC, Loureiro AV, Ferreira PH, Ferreira ML. Applying joint mobilization at different cervical vertebral levels does not influence immediate pain reduction in patients with chronic neck pain: a randomized clinical trial. The Journal of Manual & Manipulative Therapy 2009;17(2):95-100. doi: 10.1179/106698109790824686. PMID: 20046551.
36. Araujo-Soares V, McIntyre T, MacLennan G, Sniehotta FF. Development and exploratory cluster-randomised opportunistic trial of a theory-based intervention to enhance physical activity among adolescents. Psychology & Health 2009;24(7):805-822. doi: 10.1080/08870440802040707. PMID: 20205028.
37. Arbane G, Tropman D, Jackson D, Garrod R. Evaluation of an early exercise intervention after thoracotomy for non-small cell lung cancer (NSCLC), effects on quality of life, muscle strength and exercise tolerance: randomised controlled trial. Lung Cancer 2011 Feb;71(2):229-234. doi: 10.1016/j.lungcan.2010.04.025. PMID: 20541832.
38. Arias P, Chouza M, Vivas J, Cudeiro J. Effect of whole body vibration in Parkinson's disease: a controlled study. Movement Disorders 2009 Apr 30;24(6):891-898. doi: 10.1002/mds.22468. PMID: 19199362.
39. Armstrong K, Edwards H. The effects of exercise and social support on mothers reporting depressive symptoms: a pilot randomized controlled trial. International Journal of Mental Health Nursing 2003 Jun;12(2):130-138. doi: 10.1046/j.1440-0979.2003.00229.x. PMID: 12956024.
40. Armstrong K, Edwards H. The effectiveness of a pram-walking exercise programme in reducing depressive symptomatology for postnatal women. International Journal of Nursing Practice 2004 Aug;10(4):177-194. doi: 10.1111/j.1440-172X.2004.00478.x. PMID: 15265228.
41. Arnardottir RH, Boman G, Larsson K, Hedenstrom H, Emtner M. Interval training compared with continuous training in patients with COPD. Respiratory Medicine 2007 Jun;101(6):1196-1204. doi: 10.1016/j.rmed.2006.11.004. PMID: 17188853.
42. Arosio E, Minuz P, Prior M, Zuliani V, Gaino S, de Marchi S, Fontana L, Andrioli G, Lechi C, Lechi A. Vascular adhesion molecule-1 and markers of platelet function before and after a treatment with iloprost or a supervised physical exercise program in patients with peripheral arterial disease. Life Sciences 2001 Jun 15;69(4):421-433. doi: 10.1016/S0024-3205(01)01134-1. PMID: 11459433.
43. Arthur HM, Daniels C, McKelvie R, Hirsh J, Rush B. Effect of a preoperative intervention on preoperative and postoperative outcomes in low-risk patients awaiting elective coronary artery bypass graft surgery. A randomized, controlled trial. Annals of Internal Medicine 2000 Aug 15;133(4):253-262. doi: 10.7326/0003-4819-133-4-200008150-00036. PMID: 10929164.
44. Arthur HM, Smith KM, Kodis J, McKelvie R. A controlled trial of hospital versus home-based exercise in cardiac patients. Medicine and Science in Sports and Exercise 2002 Oct;34(10):1544-1550. PMID: 12370553.
45. Arvonen T, Fianu-Jonasson A, Tyni-Lenne R. Effectiveness of two conservative modes of physical therapy in women with urinary stress incontinence. Neurourology and Urodynamics 2001;20(5):591-599. doi: 10.1002/nau.1011. PMID: 11574936.
46. Asbee SM, Jenkins TR, Butler JR, White J, Elliot M, Rutledge A. Preventing excessive weight gain during pregnancy through dietary and lifestyle counselling. Obstetrics and Gynecology 2009 Feb;113(2 Pt 1):305-312. doi: 10.1097/AOG.0b013e318195baef. PMID: 19155899.
47. Assefi NP, Sherman KJ, Jacobsen C, Goldberg J, Smith WR, Buchwald D. A randomized clinical trial of acupuncture compared with sham acupuncture in fibromyalgia. Annals of Internal Medicine 2005 Jul 5;143(1):10-19. doi: 10.7326/0003-4819-143-1-200507050-00005. PMID: 15998750.
48. Atienza Meseguer A, Fernandez de las Penas C, Navarro-Poza JL, Rodriguez-Blanco C, Bosca Gandia JJ. Immediate effects of the strain/counterstrain technique in local pain evoked by tender points in the upper trapezius muscle. Clinical Chiropractic 2006 Sep;9(3):112-118. doi: 10.1016/j.clch.2006.06.003.
49. Aukee P, Immonen P, Penttinen J, Laippala P, Airaksinen O. Increase in pelvic floor muscle activity after 12 weeks' training: a randomized prospective pilot study. Urology 2002 Dec;60(6):1020-1023. PMID: 12475661.
50. Au-Yeung SSY, Ho HPY, Lai JWC, Lau RWK, Wong AYL, Lau SK. Did mobility and balance of residents living in private old age homes improve after a mobility exercise programme: a pilot study. Hong Kong Physiotherapy Journal 2002;20:16-21. doi: 10.1016/S1013-7025(09)70027-2.
51. Avis NE, Legault C, Coeytaux RR, Pian-Smith M, Shifren JL, Chen W, Valaskatgis P. A randomized, controlled pilot study of acupuncture treatment for menopausal hot flashes. Menopause 2008 Dec;15(6):1070-1078. doi: 10.1097/gme.0b013e31816d5b03. PMID: 18528313.
52. Bai S-M, Ma C, Liu Y-M, Xue W-P, Luo M, Ou Z-H. (Effects of cognitive behavior intervention and cinesiateics on the quality of life of patients with nasopharyngeal carcinoma after radiotherapy) [Chinese - simplified characters]. Zhongguo Linchuang Kangfu [Chinese Journal of Clinical Rehabilitation] 2004 Oct 15;8(29):6312-6313.
53. Bailey DM, Erith SJ, Griffin PJ, Dowson A, Brewer DS, Gant N, Williams C. Influence of cold-water immersion on indices of muscle damage following prolonged intermittent shuttle running. Journal of Sports Sciences 2007;25(11):1163-1170. doi: 10.1080/02640410600982659. PMID: 17654228.
54. Baker KR, Nelson ME, Felson DT, Layne JE, Sarno R, Roubenoff R. The efficacy of home based progressive strength training in older adults with knee osteoarthritis: a randomized controlled trial. The Journal of Rheumatology 2001 Jul;28(7):1655-1665. PMID: 11469475.
55. Bakhtiary AH, Rashidy-Pour A. Ultrasound and laser therapy in the treatment of carpal tunnel syndrome. Australian Journal of Physiotherapy 2004;50(3):147-151. doi: 10.1016/S0004-9514(14)60152-5. PMID: 15482245.
56. Bakken RC, Carey JR, di Fabio RP, Erlandson TJ, Hake JL, Intihar TW. Effect of aerobic exercise on tracking performance in elderly people: a pilot study. Physical Therapy 2001 Dec;81(12):1870-1879. PMID: 11736621.
57. Bale M, Strand L. Does functional strength training of the leg in subacute stroke improve physical performance? A pilot randomized controlled trial. Clinical Rehabilitation 2008 Oct-Nov;22(10-11):911-921. doi: 10.1177/0269215508090092. PMID: 18955423.
58. Bales GT, Gerber GS, Minor TX, Mhoon DA, McFarland JM, Kim HL, Brendler CB. Effect of preoperative biofeedback/pelvic floor training on continence in men undergoing radical prostatectomy. Urology 2000 Oct;56(4):627-630. doi: 10.1016/S0090-4295(00)00687-7. PMID: 11018619.
59. Banasik J, Williams H, Haberman M, Blank SE, Bendel R. Effect of Iyengar yoga practice on fatigue and diurnal salivary cortisol concentration in breast cancer survivors. Journal of the American Academy of Nurse Practitioners 2011 Mar;23(3):135-142. doi: 10.1111/j.1745-7599.2010.00573.x. PMID: 21355946.
60. Banerjee B, Vadiraj HS, Ram A, Rao R, Jayapal M, Gopinath KS, Ramesh BS, Rao N, Kumar A, Raghuram N, Hegde S, Nagendra HR, Prakash Hande M. Effects of an integrated yoga program in modulating psychological stress and radiation-induced genotoxic stress in breast cancer patients undergoing radiotherapy. Integrative Cancer Therapies 2007 Sep;6(3):242-250. doi: 10.1177/1534735407306214. PMID: 17761637.
61. Barakat R, Pelaez M, Montejo R, Luaces M, Zakynthinaki M. Exercise during pregnancy improves maternal health perception: a randomized controlled trial. American Journal of Obstetrics and Gynecology 2011 May;204(5):402. doi: 10.1016/j.ajog.2011.01.043. PMID: 21354547.
62. Barbara M, Antonini G, Vestri A, Volpini L, Monini S. Role of Kabat physical rehabilitation in Bell's palsy: a randomized trial. Acta Oto-Laryngologica 2010;130(1):167-172. doi: 10.3109/00016480902882469. PMID: 19430987.
63. Barbeau P, Johnson MH, Howe CA, Allison J, Davis CL, Gutin B, Lemmon CR. Ten months of exercise improves general and visceral adiposity, bone, and fitness in black girls. Obesity 2007 Aug;15(8):2077-2085. doi: 10.1038/oby.2007.247. PMID: 17712126.
64. Bardak AN, Alp M, Erhan B, Paker N, Kaya B, Onal AE. Evaluation of the clinical efficacy of conservative treatment in the management of carpal tunnel syndrome. Advances in Therapy 2009 Jan;26(1):107-116. doi: 10.1007/s12325-008-0134-7. PMID: 19165436.
65. Basford JR, Sheffield CG, Harmsen WS. Laser therapy: a randomized, controlled trial of the effects of low- intensity Nd-YAG laser irradiation on musculoskeletal back pain. Archives of Physical Medicine and Rehabilitation 1999 Jun;80(6):647-652. doi: 10.1016/S0003-9993(99)90167-3. PMID: 10378490.
66. Basler H, Jakle C, Kroner-Herwig B. Incorporation of cognitive-behavioral treatment into the medical care of chronic low back patients: a controlled randomized study in German pain treatment centers. Patient Education and Counseling 1997 Jun;31(2):113-124. doi: 10.1016/S0738-3991(97)00996-8. PMID: 9216352.
67. Bateman A, Culpan FJ, Pickering AD, Powell JH, Scott OM, Greenwood RJ. The effect of aerobic training on rehabilitation outcomes after recent severe brain injury: a randomized controlled evaluation. Archives of Physical Medicine and Rehabilitation 2001 Feb;82(2):174-182. doi: 10.1053/apmr.2001.19744. PMID: 11239307.
68. Battaglini CL, Mihalik JP, Bottaro M, Dennehy C, Petschauer MA, Hairston LS, Shields EW. Effect of exercise on the caloric intake of breast cancer patients undergoing treatment. Brazilian Journal of Medical and Biological Research [Revista Brasileira de Pesquisas Medicas e Biologicas] 2008 Aug;41(8):709-715. PMID: 18797706.
69. Baum EE, Jarjoura D, Polen AE, Faur D, Rutecki G. Effectiveness of a group exercise program in a long-term care facility: a randomized pilot trial. Journal of the American Medical Directors Association 2003 Mar-Apr;4(2):74-80. doi: 10.1016/S1525-8610(04)70279-0. PMID: 12807578.
70. Bautch JC, Malone DG, Vailas AC. Effects of exercise on knee joints with osteoarthritis: a pilot study of biologic markers. Arthritis Care & Research 1997 Feb;10(1):48-55. doi: 10.1002/art.1790100108. PMID: 9313390.
71. Bautmans I, van Hees E, Lemper JC, Mets T. The feasibility of whole body vibration in institutionalised elderly persons and its influence on muscle performance, balance and mobility: a randomised controlled trial. BMC Geriatrics 2005 Dec 22;5(17):Epub. doi: 10.1186/1471-2318-5-17. PMID: 16372905.
72. Bayne-Smith M, Fardy PS, Azzollini A, Magel J, Schmitz KH, Agin D. Improvements in heart health behaviors and reduction in coronary artery disease risk factors in urban teenaged girls through a school-based intervention: the PATH program. American Journal of Public Health 2004 Sep;94(9):1538-1543. PMID: 15333311.
73. Baysal O, Altay Z, Ozcan C, Ertem K, Yologlu S, Kayhan A. Comparison of three conservative treatment protocols in carpal tunnel syndrome. International Journal of Clinical Practice 2006 Jul;60(7):820-828. doi: 10.1111/j.1742-1241.2006.00867.x. PMID: 16704676.
74. Bemben DA, Fetters NL, Bemben MG, Nabavi N, Koh ET. Musculoskeletal responses to high- and low-intensity resistance training in early postmenopausal women. Medicine and Science in Sports and Exercise 2000 Nov;32(11):1949-1957. PMID: 11079527.
75. Bendix AF, Bendix T, Haestrup C, Busch E. A prospective, randomized 5-year follow-up study of functional restoration in chronic low back pain patients. European Spine Journal 1998 May;7(2):111-119. doi: 10.1007/s005860050040. PMID: 9629934.
76. Bendix AF, Bendix T, Vaegter K, Lund C, Frolund L, Holm L. Multidisciplinary intensive treatment for chronic low back pain: a randomized, prospective study. Cleveland Clinic Journal of Medicine 1996 Jan-Feb;63(1):62-69. PMID: 8590519.
77. Bendix T, Bendix A, Labriola M, Haestrup C, Ebbehoj N. Functional restoration versus outpatient physical training in chronic low back pain. A randomized comparative study. Spine 2000 Oct 1;25(19):2494-2500. PMID: 11013502.
78. Bendstrup KE, Ingemann Jensen J, Holm S, Bengtsson B. Out-patient rehabilitation improves activities of daily living, quality of life and exercise tolerance in chronic obstructive pulmonary disease. The European Respiratory Journal 1997 Dec;10(12):2801-2806. PMID: 9493664.
79. BenGal S, Lowe J, Mann G, Finsterbush A, Matan Y. The role of the knee brace in the prevention of anterior knee pain syndrome. The American Journal of Sports Medicine 1997 Jan-Feb;25(1):118-122. doi: 10.1177/036354659702500123. PMID: 9006705.
80. Bennell KL, Hinman RS, Metcalf BR, Buchbinder R, McConnell J, McColl G, Green S, Crossley KM. Efficacy of physiotherapy management of knee joint osteoarthritis: a randomised, double blind, placebo controlled trial. Annals of the Rheumatic Diseases 2005 Jun;64(6):906-912. doi: 10.1136/ard.2004.026526. PMID: 15897310.
81. Bennell KL, Hunt MA, Wrigley TV, Hunter DJ, McManus FJ, Hodges PW, Li L, Hinman RS. Hip strengthening reduces symptoms but not knee load in people with medial knee osteoarthritis and varus malalignment: a randomised controlled trial. Osteoarthritis and Cartilage 2010 May;18(5):621-628. doi: 10.1016/j.joca.2010.01.010. PMID: 20175973.
82. Bennell KL, Matthews B, Greig A, Briggs A, Kelly A, Sherburn M, Larsen J, Wark J. Effects of an exercise and manual therapy program on physical impairments, function and quality-of-life in people with osteoporotic vertebral fracture: a randomised, single-blind controlled pilot trial. BMC Musculoskeletal Disorders 2010 Feb 17;11(36):Epub. doi: 10.1186/1471-2474-11-36. PMID: 20163739.
83. Bennett GG, Herring SJ, Puleo E, Stein EK, Emmons KM, Gillman MW. Web-based weight loss in primary care: a randomized controlled trial. Obesity 2010 Feb;18(2):308-313. doi: 10.1038/oby.2009.242. PMID: 19696764.
84. Bennett LA, Brearley SC, Hart JA, Bailey MJ. A comparison of 2 continuous passive motion protocols after total knee arthroplasty: a controlled and randomized study. The Journal of Arthroplasty 2005 Feb;20(2):225-233. doi: 10.1016/j.arth.2004.08.009. PMID: 15902862.
85. Berghmans LCM, Frederiks CMA, de Bie RA, Weil EHJ, Smeets LWH, van Waalwijk van Doorn ESC, Janknegt RA. Efficacy of biofeedback, when included with pelvic floor muscle exercise treatment, for genuine stress incontinence. Neurourology and Urodynamics 1996;15(1):37-52. doi: 10.1002/(SICI)1520-6777(1996)15:1<37::AID-NAU4>3.0.CO;2-G. PMID: 8696355.
86. Bergland A, Thorsen H, Karesen R. Effect of exercise on mobility, balance, and health-related quality of life in osteoporotic women with a history of vertebral fracture: a randomized, controlled trial. Osteoporosis International 2011 Jun;22(6):1863-1871. doi: 10.1007/s00198-010-1435-7. PMID: 21060992.
87. Berglund G, Bolund C, Gustafsson U-L, Sjoden P-O. One-year follow-up of the 'starting again' group rehabilitation programme for cancer patients. European Journal of Cancer 1994;30(12):1744-1751. doi: 10.1016/0959-8049(94)00330-8. PMID: 7880598.
88. Bergner M, Hudson LD, Conrad DA, Patmont CM, McDonald GJ, Perrin EB, Gilson BS. The cost and efficacy of home care for patients with chronic lung disease. Medical Care 1988 Jun;26(6):566-579. PMID: 3379988.
89. Bergstrom I, Bergstrom K, Kronhed ACG, Karlsson S, Brinck J. Back extensor training increases muscle strength in postmenopausal women with osteoporosis, kyphosis and vertebral fractures. Advances in Physiotherapy 2011 Sep;13(3):110-117. doi: 10.3109/14038196.2011.581696.
90. Bergstrom I, Landgren BM, Brinck J, Freyschuss B. Physical training preserves bone mineral density in postmenopausal women with forearm fractures and low bone mineral density. Osteoporosis International 2008 Feb;19(2):177-183. doi: 10.1007/s00198-007-0445-6. PMID: 17768587.
91. Berman BM, Lao L, Langenberg P, Lee WL, Gilpin AM, Hochberg MC. Effectiveness of acupuncture as adjunctive therapy in osteoarthritis of the knee: a randomized, controlled trial. Annals of Internal Medicine 2004 Dec 21;141(12):901-910. doi: 10.7326/0003-4819-141-12-200412210-00006. PMID: 15611487.
92. Bethge M, Herbold D, Trowitzsch L, Jacobi C. Work status and health-related quality of life following multimodal work hardening: a cluster randomised trial. Journal of Back and Musculoskeletal Rehabilitation 2011;24(3):161-172. doi: 10.3233/BMR-2011-0290. PMID: 21849730.
93. Beurskens AJ, de Vet HC, Koke AJ, Lindeman E, Regtop W, van der Heijden GJ, Knipschild PG. Efficacy of traction for non-specific low back pain: a randomised clinical trial. Lancet 1995 Dec 16;346(8990):1596-1600. doi: 10.1016/S0140-6736(95)91930-9. PMID: 7500752.
94. Beurskens CH, Heymans PG. Positive effects of mime therapy on sequelae of facial paralysis: stiffness, lip mobility, and social and physical aspects of facial disability. Otology & Neurotology 2003 Jul;24(4):677-681. PMID: 12851564.
95. Beyerman KL, Palmerino MB, Zohn LE, Kane GM, Foster KA. Efficacy of treating low back pain and dysfunction secondary to osteoarthritis: chiropractic care compared with moist heat alone. Journal of Manipulative and Physiological Therapeutics 2006 Feb;29(2):107-114. doi: 10.1016/j.jmpt.2005.10.005. PMID: 16461169.
96. Bezalel T, Carmeli E, Katz-Leurer M. The effect of a group education programme on pain and function through knowledge acquisition and home-based exercise among patients with knee osteoarthritis: a parallel randomised single-blind clinical trial. Physiotherapy 2010 Jun;96(2):137-143. doi: 10.1016/j.physio.2009.09.009. PMID: 20420960.
97. Bhatia R, Field J, Grote J, Huma H. Does splintage help pain after carpal tunnel release?. Journal of Hand Surgery - British Volume 2000 Apr;25(2):150. doi: 10.1054/jhsb.2000.0365. PMID: 11062571.
98. Bialosky JE, Bishop MD, Price DD, Robinson ME, Vincent KR, George SZ. A randomized sham-controlled trial of a neurodynamic technique in the treatment of carpal tunnel syndrome. The Journal of Orthopaedic and Sports Physical Therapy 2009 Oct;39(10):709-723. doi: 10.2519/jospt.2009.3117. PMID: 19801812.
99. Bidwell AJ, Yazel B, Davin D, Fairchild TJ, Kanaley JA. Yoga training improves quality of life in women with asthma. Journal of Alternative & Complementary Medicine 2012 Aug;18(8):749-755. doi: 10.1089/acm.2011.0079. PMID: 22775424.
100. Bihaug O. Autotraksjon for ischialgipasienter [Norwegian]. Fysioterapeuten 1978 Oct;(45):377-379.
101. Biligici A, Ulusoy H, Kuru O, Canturk F. The comparison of ultrasound treatment and local steroid injection plus splinting in the carpal tunnel syndrome: a randomized controlled trial. Bratislavske Lekarske Listy [Bratislava Medical Journal] 2010;111(12):659-665. PMID: 21384736.
102. Bircan C, Karasel SA, Akgun B, El O, Alper S. Effects of muscle strengthening versus aerobic exercise program in fibromyalgia. Rheumatology International 2008 Apr;28(6):527-532. doi: 10.1007/s00296-007-0484-5. PMID: 17982749.
103. Bitterli J, Graf R, Robert F, Adler R, Mumenthaler M. Zur objektivierung der manualtherapeutischen beeinflussbarkeit des spondylogenen kopfschmerzes (Objective criteria for the evaluation of chiropractic treatment of spondylotic headache) [German]. Der Nervenarzt 1977;48:259-262. PMID: 895945.
104. Blikstad A, Gemmell H. Immediate effect of activator trigger point therapy and myofascial band therapy on non-specific neck pain in patients with upper trapezius trigger points compared to sham ultrasound: a randomised controlled trial. Clinical Chiropractic 2008 Mar;11(1):23-29. doi: 10.1016/j.clch.2007.11.002.
105. Blixen CE, Bramstedt KA, Hammel JP, Tilley BC. A pilot study of health education via a nurse-run telephone self-management programme for elderly people with osteoarthritis. Journal of Telemedicine and Telecare 2004 Feb;10(1):44-49. doi: 10.1258/135763304322764194. PMID: 15006216.
106. Blumenthal JA, Babyak MA, Doraiswamy PM, Watkins L, Hoffman BM, Barbour KA, Herman S, Craighead WE, Brosse AL, Waugh R, Hinderliter A, Sherwood A. Exercise and pharmacotherapy in the treatment of major depressive disorder. Psychosomatic Medicine 2007 Sep-Oct;69(7):587-596. doi: 10.1097/PSY.0b013e318148c19a. PMID: 17846259.
107. Blumenthal JA, Babyak MA, Moore KA, Craighead WE, Herman S, Khatri P, Waugh R, Napolitano MA, Forman LM, Appelbaum M, Doraiswamy PM, Krishnan KR. Effects of exercise training on older patients with major depression. Archives of Internal Medicine 1999 Oct 25;159(19):2349-2356. doi: 10.1001/archinte.159.19.2349. PMID: 10547175.
108. Blumenthal JA, Emery CF, Madden DJ, George LK, Coleman RE, Riddle MW, McKee DC, Reasoner J, Williams RS. Cardiovascular and behavioral effects of aerobic exercise training in healthy older men and women. Journal of Gerontology 1989 Sep;44(5):M147-M157. doi: 10.1093/geronj/44.5.M147. PMID: 2768768.
109. Blumenthal JA, Sherwood A, Babyak MA, Watkins LL, Smith PJ, Hoffman BM, O'Hayer CVF, Mabe S, Johnson J, Doraiswamy PM, Jiang W, Schocken DD, Hinderliter AL. Exercise and pharmacological treatment of depressive symptoms in patients with coronary heart disease: results from the UPBEAT (Understanding the Prognostic Benefits of Exercise and Antidepressant Therapy) study. Journal of the American College of Cardiology 2012 Sep 18;60(12):1053-1063. doi: 10.1016/j.jacc.2012.04.040. PMID: 22858387.
110. Bo K, Haakstad LAH. Is pelvic floor muscle training effective when taught in a general fitness class in pregnancy? A randomised controlled trial. Physiotherapy 2011 Sep;97(3):190-195. doi: 10.1016/j.physio.2010.08.014. PMID: 21820536.
111. Bo K, Hagen RH, Kvarstein B, Jorgensen J, Larsen S. Pelvic floor muscle exercise for the treatment of female stress urinary incontinence: III. effects of two different degrees of pelvic floor muscle exercises. Neurourology and Urodynamics 1990;9(5):489-502. doi: 10.1002/nau.1930090505.
112. Bo K, Talseth T, Holme I. Single blind, randomised controlled trial of pelvic floor exercises, electrical stimulation, vaginal cones, and no treatment in management of genuine stress incontinence in women. BMJ 1999 Feb 20;318(7182):487-493. PMID: 10024253.
113. Bo S, Ciccone G, Baldi C, Benini L, Dusio F, Forastiere G, Lucia C, Nuti C, Durazzo M, Cassader M, Gentile L, Pagano G. Effectiveness of a lifestyle intervention on metabolic syndrome: a randomized controlled trial. Journal of General Internal Medicine 2007 Dec;22(12):1695-1703. doi: 10.1007/s11606-007-0399-6. PMID: 17922167.
114. Bocalini DS, Serra AJ, dos Santos L, Murad N, Levy RF. Strength training preserves the bone mineral density of postmenopausal women without hormone replacement therapy. Journal of Aging and Health 2009 Jun;21(3):519-527. doi: 10.1177/0898264309332839. PMID: 19252142.
115. Bokmand S, Flyger H. Acupuncture relieves menopausal discomfort in breast cancer patients: a prospective, double blinded, randomized study. The Breast 2013 Jun;22(3):320-323. doi: 10.1016/j.breast.2012.07.015. PMID: 22906948.
116. Borello-France DF, Zyczynski HM, Downey PA, Rause CR, Wister JA. Effect of pelvic-floor muscle exercise position on continence and quality-of-life outcomes in women with stress urinary incontinence. Physical Therapy 2006 Jul;86(7):974-986. PMID: 16813477.
117. Borman P, Keskin D, Bodur H. The efficacy of lumbar traction in the management of patients with low back pain. Rheumatology International 2003;23(2):82-86. doi: 10.1007/s00296-002-0249-0. PMID: 12634941.
118. Borud EK, Alraek T, White A, Fonnebo V, Eggen AE, Hammar M, Astrand LL, Theodorsson E, Grimsgaard S. The acupuncture on hot flushes among menopausal women (ACUFLASH) study, a randomized controlled trial. Menopause 2009 May-Jun;16(3):484-493. doi: 10.1097/gme.0b013e31818c02ad. PMID: 19423996.
119. Bourbeau J, Julien M, Maltais F, Rouleau M, Beaupre A, Begin R, Renzi P, Nault D, Borycki E, Schwartzman K, Singh R, Collet JP. Reduction of hospital utilization in patients with chronic obstructive pulmonary disease: a disease-specific self-management intervention. Archives of Internal Medicine 2003 Mar 10;163(5):585-591. doi: 10.1001/archinte.163.5.585. PMID: 12622605.
120. Bourke L, Doll H, Crank H, Daley A, Rosario D, Saxton JM. Lifestyle intervention in men with advanced prostate cancer receiving androgen suppression therapy: a feasibility study. Cancer Epidemiology, Biomarkers & Prevention 2011 Apr;20(4):647-657. doi: 10.1158/1055-9965.EPI-10-1143. PMID: 21335510.
121. Bourke L, Thompson G, Gibson DJ, Daley A, Crank H, Adam I, Shorthouse A, Saxton J. Pragmatic lifestyle intervention in patients recovering from colon cancer: a randomized controlled pilot study. Archives of Physical Medicine and Rehabilitation 2011 May;92(5):749-755. doi: 10.1016/j.apmr.2010.12.020. PMID: 21530722.
122. Boxall AM, Barclay L, Sayers A, Caplan GA. Managing chronic obstructive pulmonary disease in the community. A randomized controlled trial of home-based pulmonary rehabilitation for elderly housebound patients. Journal of Cardiopulmonary Rehabilitation 2005 Nov-Dec;25(6):378-385. PMID: 16327534.
123. Boyd A, Yang CT, Estell K, Ms CT, Gerald LB, Dransfield M, Bamman M, Bonner J, Atkinson TP, Schwiebert LM. Feasibility of exercising adults with asthma: a randomized pilot study. Allergy, Asthma, and Clinical Immunology 2012 Aug 3;8(13):Epub. doi: 10.1186/1710-1492-8-13. PMID: 22863207.
124. Braekken IH, Majida M, Engh ME, Bo K. Can pelvic floor muscle training reverse pelvic organ prolapse and reduce prolapse symptoms? An assessor-blinded, randomized, controlled trial. American Journal of Obstetrics and Gynecology 2010 Aug;203(2):170.e171-170.e177. doi: 10.1016/j.ajog.2010.02.037. PMID: 20435294.
125. Bravo G, Gauthier P, Roy PM, Payette H, Gaulin P, Harvey M, Peloquin L, Dubois MF. Impact of a 12-month exercise program on the physical and psychological health of osteopenic women. Journal of the American Geriatrics Society 1996 Jul;44(7):756-762. PMID: 8675921.
126. Brenes GA, Williamson JD, Messier SP, Rejeski WJ, Pahor M, Ip E, Penninx BW. Treatment of minor depression in older adults: a pilot study comparing sertraline and exercise. Aging & Mental Health 2007;11(1):61-68. doi: 10.1080/13607860600736372. PMID: 17164159.
127. Brentano MA, Cadore EL, da Silva EM, Ambrosini AB, Coertjens M, Petkowicz R, Viero I, Kruel LFM. Physiological adaptations to strength and circuit training in postmenopausal women with bone loss. Journal of Strength & Conditioning Research 2008 Nov;22(6):1816-1825. doi: 10.1519/JSC.0b013e31817ae3f1. PMID: 18978624.
128. Briem K, Huijbregts P, Thorsteinsdottir M. Immediate effects of inhibitive distraction on active range of cervical flexion in patients with neck pain: a pilot study. The Journal of Manual & Manipulative Therapy 2007;15(2):82-92. PMID: 19066648.
129. Brill P, Jensen R, Koltyn K, Morgan LA, Morrow JR, Keller MJ, Jackson AW. The feasibility of conducting a group-based progressive strength training program in residents of a multi-level care facility. Activities, Adaptation & Aging 1998;22(4):53-63.
130. Brininger TL, Rogers JC, Holm MB, Baker NA, Li Z-M, Goitz RJ. Efficacy of a fabricated customized splint and tendon and nerve gliding exercises for the treatment of carpal tunnel syndrome: a randomized controlled trial. Archives of Physical Medicine and Rehabilitation 2007 Nov;88(11):1429-1435. doi: 10.1016/j.apmr.2007.07.019. PMID: 17964883.
131. Brink O, Staunstrup H, Sommer J. Stable lateral malleolar fractures treated with aircast ankle brace and DonJoy ROM-Walker brace: a prospective randomized study. Foot & Ankle International 1996 Nov;17(11):679-684. doi: 10.1177/107110079601701106. PMID: 8946182.
132. Brismee JM, Paige RL, Chyu MC, Boatright JD, Hagar JM, McCaleb JA, Quintela MM, Feng D, Xu KT, Shen CL. Group and home-based Tai Chi in elderly subjects with knee osteoarthritis: a randomized controlled trial. Clinical Rehabilitation 2007 Feb;21(2):99-111. doi: 10.1177/0269215506070505. PMID: 17264104.
133. Brison RJ, Hartling L, Dostaler S, Leger A, Rowe BH, Stiell I, Pickett W. A randomized controlled trial of an educational intervention to prevent the chronic pain of whiplash associated disorders following rear-end motor vehicle collisions. Spine 2005 Aug 15;30(16):1799-1807. PMID: 16103847.
134. British Association of Physical Medicine. Pain in the neck and arm: a multicentre trial of the effects of physiotherapy, arranged by the British Association of Physical Medicine. British Medical Journal 1966 Jan 29;1(5482):253-258. PMID: 5322503.
135. Brittle N, Patel S, Wright C, Baral S, Versfeld P, Sackley C. An exploratory cluster randomized controlled trial of group exercise on mobility and depression in care home residents. Clinical Rehabilitation 2009 Feb;23(2):146-154. doi: 10.1177/0269215508098891. PMID: 19164402.
136. Broekmans T, Roelants M, Alders G, Feys P, Thijs H, Eijnde BO. Exploring the effects of a 20-week whole-body vibration training programme on leg muscle performance and function in persons with multiple sclerosis. Journal of Rehabilitation Medicine 2010 Oct;42(9):866-872. doi: 10.2340/16501977-0609. PMID: 20878048.
137. Bronfort G. Chiropractic versus general medical treatment of low back pain: a small scale controlled clinical trial. American Journal of Chiropractic Medicine 1989;2(4):145-150.
138. Bronfort G, Evans R, Anderson AV, Svendsen KH, Bracha Y, Grimm RH. Spinal manipulation, medication, or home exercise with advice for acute and subacute neck pain: a randomized trial. Annals of Internal Medicine 2012 Jan 3;156(1 Pt 1):1-10. doi: 10.1059/0003-4819-156-1-201201030-00002. PMID: 22213489.
139. Bronfort G, Goldsmith CH, Nelson CF, Boline PD, Anderson AV. Trunk exercise combined with spinal manipulative or NSAID therapy for chronic low back pain: a randomized, observer-blinded clinical trial. Journal of Manipulative and Physiological Therapeutics 1996 Nov-Dec;19(9):570-582. PMID: 8976475.
140. Brown P, Clark MM, Atherton P, Huschka M, Sloan JA, Gamble G, Girardi J, Frost MH, Piderman K, Rummans TA. Will improvement in quality of life (QOL) impact fatigue in patients receiving radiation therapy for advanced cancer?. American Journal of Clinical Oncology 2006 Feb;29(1):52-58. doi: 10.1097/01.coc.0000190459.14841.55. PMID: 16462503.
141. Brox JI, Sorensen R, Friis A, Nygaard O, Indahl A, Keller A, Ingebrigtsen T, Eriksen HR, Holm I, Koller AK, Riise R, Reikeras O. Randomized clinical trial of lumbar instrumented fusion and cognitive intervention and exercises in patients with chronic low back pain and disc degeneration. Spine 2003 Sep 1;28(17):1913-1921. PMID: 12973134.
142. Bruce-Brand RA, Walls RJ, Ong JC, Emerson BS, O'Byrne JM, Moyna NM. Effects of home-based resistance training and neuromuscular electrical stimulation in knee osteoarthritis: a randomized controlled trial. BMC Musculoskeletal Disorders 2012 Jul 3;13(118):Epub. doi: 10.1186/1471-2474-13-118. PMID: 22759883.
143. Brushoj C, Larsen K, Albrecht-Beste E, Nielsen MB, Loye F, Holmich P. Prevention of overuse injuries by a concurrent exercise program in subjects exposed to an increase in training load: a randomized controlled trial of 1,020 army recruits. The American Journal of Sports Medicine 2008 Apr;36(4):663-670. doi: 10.1177/0363546508315469. PMID: 18337359.
144. Bruun-Olsen V, Heiberg KE, Mengshoel AM. Continuous passive motion as an adjunct to active exercises in early rehabilitation following total knee arthroplasty -- a randomized controlled trial. Disability and Rehabilitation 2009;31(4):277-283. doi: 10.1080/09638280801931204. PMID: 18608367.
145. Bruunsgaard H, Bjerregaard E, Schroll M, Pedersen BK. Muscle strength after resistance training is inversely correlated with baseline levels of soluble tumor necrosis factor receptors in the oldest old. Journal of the American Geriatrics Society 2004 Feb;52(2):237-241. doi: 10.1111/j.1532-5415.2004.52061.x. PMID: 14728633.
146. Bruyere O, Wuidart MA, di Palma E, Gourlay M, Ethgen O, Richy F, Reginster JY. Controlled whole body vibration to decrease fall risk and improve health-related quality of life of nursing home residents. Archives of Physical Medicine and Rehabilitation 2005 Feb;86(2):303-307. doi: 10.1016/j.apmr.2004.05.019. PMID: 15706558.
147. Buchheit M, Peiffer JJ, Abbiss CR, Laursen PB. Effect of cold water immersion on postexercise parasympathetic reactivation. American Journal of Physiology - Heart and Circulatory Physiology 2009 Feb;296(2):H421-H427. doi: 10.1152/ajpheart.01017.2008. PMID: 19074671.
148. Buettner LL, Farrario J. Therapeutic recreation-nursing team: a therapeutic intervention for nursing home residents with dementia. Annual in Therapeutic Recreation 1997-1998;7:21-28.
149. Buist I, Bredeweg SW, van Mechelen W, Lemmink KA, Pepping GJ, Diercks RL. No effect of a graded training program on the number of running-related injuries in novice runners: a randomized controlled trial. The American Journal of Sports Medicine 2008 Jan;36(1):33-39. doi: 10.1177/0363546507307505. PMID: 17940147.
150. Burgio KL, Goode PS, Locher JL, Umlauf MG, Roth DL, Richter HE, Varner RE, Lloyd LK. Behavioral training with and without biofeedback in the treatment of urge incontinence in older women: a randomized controlled trial. JAMA 2002 Nov 13;288(18):2293-2299. doi: 10.1001/jama.288.18.2293. PMID: 12425706.
151. Burgio KL, Goode PS, Richter HE, Markland AD, Johnson TM 2nd, Redden DT. Combined behavioral and individualized drug therapy versus individualized drug therapy alone for urge urinary incontinence in women. The Journal of Urology 2010 Aug;184(2):598-603. doi: 10.1016/j.juro.2010.03.141. PMID: 20639023.
152. Burgio KL, Goode PS, Urban DA, Umlauf MG, Locher JL, Bueschen A, Redden DT. Preoperative biofeedback assisted behavioral training to decrease post-prostatectomy incontinence: a randomized, controlled trial. The Journal of Urology 2006 Jan;175(1):196-201. doi: 10.1016/S0022-5347(05)00047-9. PMID: 16406909.
153. Burke DT, Burke MM, Stewart GW, Cambre A. Splinting for carpal tunnel syndrome: in search of the optimal angle. Archives of Physical Medicine and Rehabilitation 1994 Nov;75(11):1241-1244. PMID: 7979936.
154. Burke J, Buchberger DJ, Carey-Loghmani MT, Dougherty PE, Greco DS, Dishman JD. A pilot study comparing two manual therapy interventions for carpal tunnel syndrome. Journal of Manipulative and Physiological Therapeutics 2007 Jan;30(1):50-61. doi: 10.1016/j.jmpt.2006.11.014. PMID: 17224356.
155. Burke V, Milligan RA, Thompson C, Taggart AC, Dunbar DL, Spencer MJ, Medland A, Gracey MP, Vandongen R, Beilin LJ. A controlled trial of health promotion programs in 11-year-olds using physical activity "enrichment" for higher risk children. The Journal of Pediatrics 1998 May;132(5):840-848. PMID: 9602197.
156. Burnham TR, Wilcox A. Effects of exercise on physiological and psychological variables in cancer survivors. Medicine and Science in Sports and Exercise 2002 Dec;34(12):1863-1867. PMID: 12471288.
157. Burns PA, Pranikoff K, Nochajski TH, Hadley EC, Levy KJ, Ory MG. A comparison of effectiveness of biofeedback and pelvic muscle exercise treatment of stress incontinence in older community-dwelling women. Journal of Gerontology 1993 Jul;48(4):M167-M174. doi: 10.1093/geronj/48.4.M167. PMID: 8315230.
158. Buroker KC, Schwane JA. Does postexercise static stretching alleviate delayed muscle soreness?. The Physician and Sportsmedicine 1989 Jun;17(6):65-83.
159. Burridge JH, Mann GE, Malone L, Taylor PN. A randomized controlled pilot study to investigate the effects of neuromuscular electrical stimulation on upper limb function following stroke. Neurorehabilitation and Neural Repair 2002 Mar;16(1):11-13. doi: 10.1177/154596802400470285.
160. Bury TF, Akelman E, Weiss AP. Prospective, randomized trial of splinting after carpal tunnel release. Annals of Plastic Surgery 1995 Jul;35(1):19-22. PMID: 7574280.
161. Bush C, Ditto B, Feuerstein M. A controlled evaluation of paraspinal EMG biofeedback in the treatment of chronic low back pain. Health Psychology 1985;4(4):307-321. doi: 10.1037/0278-6133.4.4.307. PMID: 2932330.
162. Bush PJ, Zuckerman AE, Theiss PK, Taggart VS, Horowitz C, Sheridan MJ, Walter HJ. Cardiovascular risk factor prevention in black schoolchildren: two-year results of the "Know Your Body" program. American Journal of Epidemiology 1989 Mar;129(3):466-482. PMID: 2916540.
163. Buszewicz M, Rait G, Griffin M, Nazareth I, Patel A, Atkinson A, Barlow J, Haines A. Self management of arthritis in primary care: randomised controlled trial. BMJ 2006 Oct 28;333(7574):879-883. doi: 10.1136/bmj.38965.375718.80. PMID: 17040926.
164. Byl N, Roderick J, Mohamed O, Hanny M, Kotler J, Smith A, Tang M, Abrams G. Effectiveness of sensory and motor rehabilitation of the upper limb following the principles of neuroplasticity: patients stable poststroke. Neurorehabilitation and Neural Repair 2003 Sep;17(3):176-191. doi: 10.1177/0888439003257137. PMID: 14503438.
165. Cadmus LA, Salovey P, Yu H, Chung G, Kasl S, Irwin ML. Exercise and quality of life during and after treatment for breast cancer: results of two randomized controlled trials. Psycho-Oncology 2009 Apr;18(4):343-352. doi: 10.1002/pon.1525. PMID: 19242918.
166. Calfas KJ, Kaplan RM, Ingram RE. One-year evaluation of cognitive-behavioral intervention in osteoarthritis. Arthritis Care & Research 1992 Dec;5(4):202-209. doi: 10.1002/art.1790050404. PMID: 1489766.
167. Calfas KJ, Sallis JF, Nichols JF, Sarkin JA, Johnson MF, Caparosa S, Thompson S, Gehrman CA, Alcaraz JE. Project GRAD: two-year outcomes of a randomized controlled physical activity intervention among young adults. American Journal of Preventive Medicine 2000 Jan;18(1):28-37. PMID: 10808980.
168. Callaway LK, Colditz PB, Byrne NM, Lingwood BE, Rowlands IJ, Foxcroft K, McIntyre HD, Bambino Group. Prevention of gestational diabetes: feasibility issues for an exercise intervention in obese pregnant women. Diabetes Care 2010 Jul;33(7):1457-1459. doi: 10.2337/dc09-2336. PMID: 20357374.
169. Cambach W, Chadwick-Straver RV, Wagenaar RC, van Keimpema AR, Kemper HC. The effects of a community-based pulmonary rehabilitation programme on exercise tolerance and quality of life: a randomized controlled trial. The European Respiratory Journal 1997 Jan;10(1):104-113. PMID: 9032501.
170. Cambier DC, de Corte E, Danneels LA, Witvrouw EE. Treating sensory impairments in the post-stroke upper limb with intermittent pneumatic compression. Results of a preliminary trial. Clinical Rehabilitation 2003 Feb;17(1):14-20. doi: 10.1191/0269215503cr580oa. PMID: 12617375.
171. Cammu H, van Nylen M. Pelvic floor exercises versus vaginal weight cones in genuine stress incontinence. European Journal of Obstetrics, Gynecology, and Reproductive Biology 1998 Mar;77(1):89-93. PMID: 9550207.
172. Campbell A, Mutrie N, White F, McGuire F, Kearney N. A pilot study of a supervised group exercise programme as a rehabilitation treatment for women with breast cancer receiving adjuvant treatment. European Journal of Oncology Nursing 2005 Mar;9(1):56-63. doi: 10.1016/j.ejon.2004.03.007. PMID: 15774341.
173. Carlson JJ, Johnson JA, Franklin BA, VanderLaan RL. Program participation, exercise adherence, cardiovascular outcomes, and program cost of traditional versus modified cardiac rehabilitation. The American Journal of Cardiology 2000 Jul 1;86(1):17-23. doi: 10.1016/S0002-9149(00)00822-5. PMID: 10867086.
174. Carmack CL, Amaral-Melendez M, Boudreaux E, Brantley PJ, Franks JD, Jones GN, Franks BD, McKnight GT. Exercise as a component in the physical and psychological rehabilitation of hemodialysis patients. International Journal of Rehabilitation and Health 1995 Jan;1(1):13-23. doi: 10.1007/BF02214958.
175. Carney RM, Templeton B, Hong BA, Harter HR, Hagberg JM, Schechtman KB, Goldberg AP. Exercise training reduces depression and increases the performance of pleasant activities in hemodialysis patients. Nephron 1987;47(3):194-198. PMID: 3317091.
176. Casaburi R, Patessio A, Ioli F, Zanaboni S, Donner CF, Wasserman K. Reductions in exercise lactic acidosis and ventilation as a result of exercise training in patients with obstructive lung disease. The American Review of Respiratory Disease 1991 Jan;143(1):9-18. doi: 10.1164/ajrccm/143.1.9. PMID: 1986689.
177. Casanova C, Celli BR, Tost L, Soriano E, Abreu J, Velasco V, Santolaria F. Long-term controlled trial of nocturnal nasal positive pressure ventilation in patients with severe COPD. Chest 2000 Dec;118(6):1582-1590. doi: 10.1378/chest.118.6.1582. PMID: 11115443.
178. Cassidy JD, Lopes AA, Yong-Hing K. The immediate effect of manipulation versus mobilization on pain and range of motion in the cervical spine: a randomized controlled trial. Journal of Manipulative and Physiological Therapeutics 1992 Nov-Dec;15(9):570-575. PMID: 1469341.
179. Castaneda C, Gordon PL, Uhlin KL, Levey AS, Kehayias JJ, Dwyer JT, Fielding RA, Roubenoff R, Singh MF. Resistance training to counteract the catabolism of a low-protein diet in patients with chronic renal insufficiency -- a randomized, controlled trial. Annals of Internal Medicine 2001 Dec 4;135(11):965-976. doi: 10.7326/0003-4819-135-11-200112040-00008. PMID: 11730397.
180. Castro RA, Arruda RM, Zanetti MRD, Santos PD, Sartori MGF, Girao MJBC. Single-blind, randomized, controlled trial of pelvic floor muscle training, electrical stimulation, vaginal cones, and no active treatment in the management of stress urinary incontinence. Clinics (Sao Paulo, Brazil) 2008 Aug;63(4):465-472. doi: 10.1590/S1807-59322008000400009. PMID: 18719756.
181. Castro-Sanchez AM, Mataran-Penarrocha GA, Feriche-Fernandez-Castanys B, Fernandez-Sola C, Sanchez-Labraca N, Moreno-Lorenzo C. A program of 3 physical therapy modalities improves peripheral arterial disease in diabetes type 2 patients: a randomized controlled trial. The Journal of Cardiovascular Nursing 2013 Jan-Feb;28(1):74-82. doi: 10.1097/JCN.0b013e318239f419. PMID: 22222177.
182. Cebesoy O, Kose KC, Kuru I, Altinel L, Gul R, Demirtas M. Use of a splint following open carpal tunnel release: a comparative study. Advances in Therapy 2007 May;24(3):478-484. doi: 10.1007/BF02848769. PMID: 17660155.
183. Cejudo P, Bautista J, Montemayor T, Villagomez R, Jimenez L, Ortega F, Campos Y, Sanchez H, Arenas J. Exercise training in mitochondrial myopathy: a randomized controlled trial. Muscle & Nerve 2005 Sep;32(3):342-350. doi: 10.1002/mus.20368. PMID: 15962332.
184. Celiker R, Arslan S, Inanici F. Corticosteroid injection versus nonsteroidal antiinflammatory drug and splinting in carpal tunnel syndrome. American Journal of Physical Medicine & Rehabilitation 2002 Mar;81(3):182-186. PMID: 11989514.
185. Celli BR, Rodriguez KS, Snider GL. A controlled trial of intermittent positive pressure breathing, incentive spirometry, and deep breathing exercises in preventing pulmonary complications after abdominal surgery. The American Review of Respiratory Disease 1984 Jul;130(1):12-15. PMID: 6377994.
186. Cen SY, Loy SF, Sletten EG, Mclaine A. The effect of traditional Chinese Therapeutic Massage on individuals with neck pain. Clinical Acupuncture & Oriental Medicine 2003;4(2-3):88-93. doi: 10.1016/S1461-1449(03)00043-4.
187. Centemero A, Rigatti L, Giraudo D, Lazzeri M, Lughezzani G, Zugna D, Montorsi F, Rigatti P, Guazzoni G. Preoperative pelvic floor muscle exercise for early continence after radical prostatectomy: a randomised controlled study. European Urology 2010 Jun;57(6):1039-1044. doi: 10.1016/j.eururo.2010.02.028.
188. Chan AWK, Lee A, Suen LKP, Tam WWS. Tai Chi Qigong improves lung functions and activity tolerance in COPD clients: a single blind, randomized controlled trial. Complementary Therapies in Medicine 2011 Feb;19(1):3-11. doi: 10.1016/j.ctim.2010.12.007. PMID: 21296261.
189. Chan K, Qin L, Lau M, Woo J, Au S, Choy W, Lee K, Lee S. A randomized, prospective study of the effects of Tai Chi Chun exercise on bone mineral density in postmenopausal women. Archives of Physical Medicine and Rehabilitation 2004 May;85(5):717-722. doi: 10.1016/j.apmr.2003.08.091. PMID: 15129394.
190. Chan KM, Amirjani N, Sumrain M, Clarke A, Strohschein FJ. Randomized controlled trial of strength training in post-polio patients. Muscle & Nerve 2003 Mar;27(3):332-338. doi: 10.1002/mus.10327. PMID: 12635120.
191. Chandwani KD, Thornton B, Perkins GH, Arun B, Raghuram NV, Nagendra HR, Wei Q, Cohen L. Yoga improves quality of life and benefit finding in women undergoing radiotherapy for breast cancer. Journal of the Society for Integrative Oncology 2010 Spring;8(2):43-55. PMID: 20388445.
192. Chang P-H, Lai Y-H, Shun S-C, Lin L-Y, Chen M-L, Yang Y, Tsai J-C, Huang G-S, Cheng S-Y. Effects of a walking intervention on fatigue-related experiences of hospitalized acute myelogenous leukemia patients undergoing chemotherapy: a randomized controlled trial. Journal of Pain and Symptom Management 2008 May;35(5):524-534. doi: 10.1016/j.jpainsymman.2007.06.013. PMID: 18280104.
193. Chang T-F, Liou T-H, Chen C-H, Huang Y-C, Chang K-H. Effects of elastic-band exercise on lower-extremity function among female patients with osteoarthritis of the knee. Disability and Rehabilitation 2012;34(20):1727-1735. doi: 10.3109/09638288.2012.660598. PMID: 22397710.
194. Chauhan AJ, McLindon JP, Dillon P, Sawyer JP, Gray L, Leahy BC. Regular balloon inflation for patients with chronic bronchitis: a randomised controlled trial. BMJ 1992 Jun 27;304(6843):1668-1669. PMID: 1633520.
195. Cheetham DR, Burgess L, Ellis M, Williams A, Greenhalgh RM, Davies AH. Does supervised exercise offer adjuvant benefit over exercise advice alone for the treatment of intermittent claudication? A randomised trial. European Journal of Vascular and Endovascular Surgery 2004 Jan;27(1):17-23. doi: 10.1016/j.ejvs.2003.09.012. PMID: 14652832.
196. Chen J-C, Liang C-C, Shaw F-Z. Facilitation of sensory and motor recovery by thermal intervention for the hemiplegic upper limb in acute stroke patients: a single-blind randomized clinical trial. Stroke 2005 Dec;36(12):2665-2669. doi: 10.1161/01.STR.0000189992.06654.ab. PMID: 16269638.
197. Chen JLT, Godfrey S, Ng TT, Moorthi R, Liangos O, Ruthazer R, Jaber BL, Levey AS, Castaneda-Sceppa C. Effect of intra-dialytic, low-intensity strength training on functional capacity in adult haemodialysis patients: a randomized pilot trial. Nephrology, Dialysis, Transplantation 2010 Jun;25(6):1936-1943. doi: 10.1093/ndt/gfp739. PMID: 20100734.
198. Chen L, Zhang X-L, Ding H, Tao Y-Q, Zhan H-S. (Comparative study on effects of manipulation treatment and transcutaneous electrical nerve stimulation on patients with cervicogenic headache) [Chinese - simplified characters]. Zhong Xi Yi Jie He Xue Bao [Journal of Chinese Integrative Medicine] 2007 Jul;5(4):403-406. doi: 10.3736/jcim20070408. PMID: 17631795.
199. Cheng AS-K, Chan EP-S. The effect of individual job coaching and use of health threat in a job-specific occupational health education program on prevention of work-related musculoskeletal back injury. Journal of Occupational and Environmental Medicine 2009 Dec;51(12):1413-1421. doi: 10.1097/JOM.0b013e3181bfb2a8. PMID: 19952790.
200. Cheng S, Sipila S, Taaffe DR, Puolakka J, Suominen H. Change in bone mass distribution induced by hormone replacement therapy and high-impact physical exercise in post-menopausal women. Bone 2002 Jul;31(1):126-135. PMID: 12110425.
201. Cherkin DC, Deyo RA, Battie M, Street J, Barlow W. A comparison of physical therapy, chiropractic manipulation, and provision of an educational booklet for the treatment of patients with low back pain. The New England Journal of Medicine 1998 Oct 8;339(15):1021-1029. doi: 10.1056/NEJM199810083391502. PMID: 9761803.
202. Cherkin DC, Deyo RA, Street JH, Hunt M, Barlow W. Pitfalls of patient education. Limited success of a program for back pain in primary care. Spine 1996 Feb 1;21(3):345-355. PMID: 8742212.
203. Cherkin DC, Eisenberg D, Sherman KJ, Barlow W, Kaptchuk TJ, Street J, Deyo RA. Randomized trial comparing traditional Chinese medical acupuncture, therapeutic massage, and self-care education for chronic low back pain. Archives of Internal Medicine 2001 Apr 23;161(8):1081-1088. doi: 10.1001/archinte.161.8.1081. PMID: 11322842.
204. Cheung KKW, Au KY, Lam WWS, Jones AYM. Effects of a structured exercise programme on functional balance in visually impaired elderly living in a residential setting. Hong Kong Physiotherapy Journal 2008;26:45-50. doi: 10.1016/S1013-7025(09)70007-7.
205. Cheville AL, Girardi J, Clark MM, Rummans TA, Pittelkow T, Brown P, Hanson J, Atherton P, Johnson ME, Sloan JA, Gamble G. Therapeutic exercise during outpatient radiation therapy for advanced cancer: feasibility and impact on physical well-being. American Journal of Physical Medicine & Rehabilitation 2010 Aug;89(8):611-619. doi: 10.1097/PHM.0b013e3181d3e782. PMID: 20531162.
206. Chiarelli P, Cockburn J. Promoting urinary continence in women after delivery: randomised controlled trial. BMJ 2002 May 25;324(7348):1241-1246. doi: 10.1136/bmj.324.7348.1241. PMID: 12028976.
207. Chiarello CM, Gundersen L, O'Halloran T. The effect of continuous passive motion duration and increment on range of motion in total knee arthroplasty patients. The Journal of Orthopaedic and Sports Physical Therapy 1997 Feb;25(2):119-127. PMID: 9007770.
208. Chilibeck PD, Davison KS, Whiting SJ, Suzuki Y, Janzen CL, Peloso P. The effect of strength training combined with bisphosphonate (etidronate) therapy on bone mineral, lean tissue, and fat mass in postmenopausal women. Canadian Journal of Physiology and Pharmacology 2002 Oct;80(10):941-950. doi: 10.1139/y02-126. PMID: 12450060.
209. Chin A Paw MJM, van Poppel MN, Twisk JW, van Mechelen W. Effects of resistance and all-round, functional training on quality of life, vitality and depression of older adults living in long-term care facilities: a 'randomized' controlled trial (ISRCTN87177281). BMC Geriatrics 2004 Jul 2;4(5):Epub. doi: 10.1186/1471-2318-4-5. PMID: 15233841.
210. Chiu TT, Hui-Chan CW, Chein G. A randomized clinical trial of TENS and exercise for patients with chronic neck pain. Clinical Rehabilitation 2005 Dec;19(8):850-860. doi: 10.1191/0269215505cr920oa. PMID: 16323384.
211. Cho OH, Yoo YS, Kim NC. Efficacy of comprehensive group rehabilitation for women with early breast cancer in South Korea. Nursing & Health Sciences 2006 Sep;8(3):140-146. doi: 10.1111/j.1442-2018.2006.00271.x. PMID: 16911173.
212. Choi G, Raiturker PP, Kim MJ, Jin CD, Chae YS. The effect of early isolated lumbar extension exercise program for patients with herniated disc undergoing lumbar discectomy. Neurosurgery 2005 Oct;57(4):764-772. doi: 10.1227/01.NEU.0000175858.80925.38. PMID: 16239890.
213. Choi JH, Moon JS, Song R. Effects of Sun-style Tai Chi exercise on physical fitness and fall prevention in fall-prone older adults. Journal of Advanced Nursing 2005 Jul;51(2):150-157. doi: 10.1111/j.1365-2648.2005.03480.x. PMID: 15963186.
214. Chouza M, Arias P, Vinas S, Cudeiro J. Acute effects of whole-body vibration at 5, 6, and 9 Hz on balance and gait in patients with Parkinson's disease. Movement Disorders 2011 Apr;26(5):920-921. doi: 10.1002/mds.23582. PMID: 21425338.
215. Chow R, Harrison JE, Notarius C. Effect of two randomised exercise programmes on bone mass of healthy postmenopausal women. British Medical Journal 1987 Dec 5;295(6611):1441-1444. PMID: 3121055.
216. Chown M, Whittamore L, Rush M, Allan S, Stott D, Archer M. A prospective study of patients with chronic back pain randomised to, group exercise, physiotherapy or osteopathy. Physiotherapy 2008 Mar;94(1):21-28. doi: 10.1016/j.physio.2007.04.014.
217. Christie AD, Willoughby GL. The effect of interferential therapy on swelling following open reduction and internal fixation of ankle fractures. Physiotherapy Theory and Practice 1990;6(1):3-7.
218. Christofoletti G, Oliani MM, Gobbi S, Stella F, Bucken Gobbi LT, Renato Canineu P. A controlled clinical trial on the effects of motor intervention on balance and cognition in institutionalized elderly patients with dementia. Clinical Rehabilitation 2008 Jul;22(7):618-626. doi: 10.1177/0269215507086239. PMID: 18586813.
219. Chubak J, Ulrich CM, Tworoger SS, Sorensen B, Yasui Y, Irwin ML, Stanczyk FZ, Potter JD, McTiernan A. Effect of exercise on bone mineral density and lean mass in postmenopausal women. Medicine and Science in Sports and Exercise 2006 Jul;38(7):1236-1244. doi: 10.1249/01.mss.0000227308.11278.d7. PMID: 16826020.
220. Chuin A, Labonte M, Tessier D, Khalil A, Bobeuf F, Doyon CY, Rieth N, Dionne IJ. Effect of antioxidants combined to resistance training on BMD in elderly women: a pilot study. Osteoporosis International 2009 Jul;20(7):1253-1258. doi: 10.1007/s00198-008-0798-5. PMID: 19020919.
221. Ciuffetti G, Paltriccia R, Lombardini R, Lupattelli G, Pasqualini L, Mannarino E. Treating peripheral arterial occlusive disease: pentoxifylline versus exercise. International Angiology 1994 Mar;13(1):33-39. PMID: 7915754.
222. Clapp JF 3rd, Kim H, Burciu B, Schmidt S, Petry K, Lopez B. Continuing regular exercise during pregnancy: effect of exercise volume on fetoplacental growth. American Journal of Obstetrics and Gynecology 2002 Jan;186(1):142-147. doi: 10.1067/mob.2002.119109. PMID: 11810100.
223. Clark BA, Wade MG, Massey BH, van Dyke R. Response of institutionalized geriatric mental patients to a twelve-week program of regular physical activity. Journal of Gerontology 1975 Sep;30(5):565-573. doi: 10.1093/geronj/30.5.565. PMID: 1181361.
224. Clark VM, Burden AM. A 4-week wobble board exercise programme improved muscle onset latency and perceived stability in individuals with a functionally unstable ankle. Physical Therapy in Sport 2005 Nov;6(4):181-187. doi: 10.1016/j.ptsp.2005.08.003.
225. Cleland JA, Childs MJD, McRae M, Palmer JA, Stowell T. Immediate effects of thoracic manipulation in patients with neck pain: a randomized clinical trial. Manual Therapy 2005 May;10(2):127-135. doi: 10.1016/j.math.2004.08.005. PMID: 15922233.
226. Clini E, Sturani C, Rossi A, Viaggi S, Corrado A, Donner CF, Ambrosino N. The Italian multicentre study on noninvasive ventilation in chronic obstructive pulmonary disease patients. The European Respiratory Journal 2002 Sep;20(3):529-538. doi: 10.1183/09031936.02.02162001. PMID: 12358325.
227. Cochrane LM, Clark CJ. Benefits and problems of a physical training programme for asthmatic patients. Thorax 1990 May;45(5):345-351. doi: 10.1136/thx.45.5.345. PMID: 2116678.
228. Cohen L, Warneke C, Fouladi RT, Rodriguez MA, Chaoul-Reich A. Psychological adjustment and sleep quality in a randomized trial of the effects of a Tibetan yoga intervention in patients with lymphoma. Cancer 2004 May 15;100(10):2253-2260. doi: 10.1002/cncr.20236. PMID: 15139072.
229. Colin-Ramirez E, Castillo-Martinez L, Orea-Tejeda A, Vergara-Castaneda A, Keirns-Davis C, Villa-Romero A. Outcomes of a school-based intervention (RESCATE) to improve physical activity patterns in Mexican children aged 8 to 10 years. Health Education Research 2010 Dec;25(6):1042-1049. doi: 10.1093/her/cyq056. PMID: 20884847.
230. Collins EG, Langbein WE, Fehr L, O'Connell S, Jelinek C, Hagarty E, Edwards L, Reda D, Tobin MJ, Laghi F. Can ventilation-feedback training augment exercise tolerance in patients with chronic obstructive pulmonary disease?. American Journal of Respiratory and Critical Care Medicine 2008 Apr 15;177(8):844-852. doi: 10.1164/rccm.200703-477OC. PMID: 18202351.
231. Collins EG, Langbein WE, Orebaugh C, Bammert C, Hanson K, Reda D, Edwards LC, Littooy FN. Cardiovascular training effect associated with polestriding exercise in patients with peripheral arterial disease. The Journal of Cardiovascular Nursing 2005 May-Jun;20(3):177-185. PMID: 15870588.
232. Collins TC, Lunos S, Carlson T, Henderson K, Lightbourne M, Nelson B, Hodges JS. Effects of a home-based walking intervention on mobility and quality of life in people with diabetes and peripheral arterial disease: a randomized controlled trial. Diabetes Care 2011 Oct;34(10):2174-2179. doi: 10.2337/dc10-2399. PMID: 21873560.
233. Colwell CW Jr, Morris BA. The influence of continuous passive motion on the results of total knee arthroplasty. Clinical Orthopaedics and Related Research 1992 Mar;(276):225-228. PMID: 1537158.
234. Comorosan S, Vasilco R, Arghiropol M, Paslaru L, Jieanu V, Stelea S. The effect of diapulse therapy on the healing of decubitus ulcer. Romanian Journal of Physiology 1993 Jan-Jun;30(1-2):41-45. PMID: 7982015.
235. Cook AC, Szabo RM, Birkholz SW, King EF. Early mobilization following carpal tunnel release. A prospective randomized study. Journal of Hand Surgery - British Volume 1995 Apr;20(2):228-230. doi: 10.1016/S0266-7681(05)80057-9. PMID: 7797977.
236. Cooke EV, Tallis RC, Clark A, Pomeroy VM. Efficacy of functional strength training on restoration of lower-limb motor function early after stroke: phase I randomized controlled trial. Neurorehabilitation and Neural Repair 2010 Jan;24(1):88-96. doi: 10.1177/1545968309343216. PMID: 19704158.
237. Coppieters MW, Stappaerts KH, Wouters LL, Janssens K. The immediate effects of a cervical lateral glide treatment technique in patients with neurogenic cervicobrachial pain. The Journal of Orthopaedic and Sports Physical Therapy 2003 Jul;33(7):369-378. PMID: 12918862.
238. Coppin RJ, Wicke DM, Little PS. Managing nocturnal leg cramps -- calf-stretching exercises and cessation of quinine treatment: a factorial randomised controlled trial. British Journal of General Practice 2005 Mar;55(512):186-191. PMID: 15808033.
239. Cordero Y, Mottola MF, Vargas J, Blanco M, Barakat R. Exercise is associated with a reduction in gestational diabetes mellitus. Medicine and Science in Sports and Exercise 2015 Jul;47(7):1328-1333. doi: 10.1249/MSS.0000000000000547. PMID: 25333246.
240. Corey DT, Koepfler LE, Etlin D, Day HI. A limited functional restoration program for injured workers: a randomized trial. Journal of Occupational Rehabilitation 1996 Dec;6(4):239-249. doi: 10.1007/BF02110886. PMID: 24235022.
241. Cott CA, Dawson P, Sidani S, Wells D. The effects of a walking/talking program on communication, ambulation, and functional status in residents with Alzheimer disease. Alzheimer Disease & Associated Disorders 2002 Apr-Jun;16(2):81-87. PMID: 12040303.
242. Coultas D, Frederick J, Barnett B, Singh G, Wludyka P. A randomized trial of two types of nurse-assisted home care for patients with COPD. Chest 2005 Oct;128(4):2017-2024. doi: 10.1378/chest.128.4.2017. PMID: 16236850.
243. Counil FP, Varray A, Matecki S, Beurey A, Marchal P, Voisin M, Prefaut C. Training of aerobic and anaerobic fitness in children with asthma. The Journal of Pediatrics 2003 Feb;142(2):179-184. doi: 10.1067/mpd.2003.83. PMID: 12584541.
244. Courneya KS, Friedenreich CM, Quinney HA, Fields AL, Jones LW, Fairey AS. A randomized trial of exercise and quality of life in colorectal cancer survivors. European Journal of Cancer Care 2003 Dec;12(4):347-357. doi: 10.1046/j.1365-2354.2003.00437.x. PMID: 14982314.
245. Courneya KS, Friedenreich CM, Sela RA, Quinney HA, Rhodes RE, Handman M. The group psychotherapy and home-based physical exercise (group-hope) trial in cancer survivors: physical fitness and quality of life outcomes. Psycho-Oncology 2003 Jun;12(4):357-374. doi: 10.1002/pon.658. PMID: 12748973.
246. Courneya KS, Jones LW, Peddle CJ, Sellar CM, Reiman T, Joy AA, Chua N, Tkachuk L, Mackey JR. Effects of aerobic exercise training in anemic cancer patients receiving darbepoetin alfa: a randomized controlled trial. The Oncologist 2008 Sep;13(9):1012-1020. doi: 10.1634/theoncologist.2008-0017. PMID: 18779540.
247. Courneya KS, Mackey JR, Bell GJ, Jones LW, Field CJ, Fairey AS. Randomized controlled trial of exercise training in postmenopausal breast cancer survivors: cardiopulmonary and quality of life outcomes. Journal of Clinical Oncology 2003 May 1;21(9):1660-1668. doi: 10.1200/JCO.2003.04.093. PMID: 12721239.
248. Courneya KS, Segal RJ, Mackey JR, Gelmon K, Reid RD, Friedenreich CM, Ladha AB, Proulx C, Vallance JKH, Lane K, Yasui Y, McKenzie DC. Effects of aerobic and resistance exercise in breast cancer patients receiving adjuvant chemotherapy: a multicenter randomized controlled trial. Journal of Clinical Oncology 2007 Oct 1;25(28):4396-4404. doi: 10.1200/JCO.2006.08.2024. PMID: 17785708.
249. Courneya KS, Sellar CM, Stevinson C, McNeely ML, Peddle CJ, Friedenreich CM, Tankel K, Basi S, Chua N, Mazurek A, Reiman T. Randomized controlled trial of the effects of aerobic exercise on physical functioning and quality of life in lymphoma patients. Journal of Clinical Oncology 2009 Sep 20;27(27):4605-4612. doi: 10.1200/JCO.2008.20.0634. PMID: 19687337.
250. Cowie A, Thow MK, Granat MH, Mitchell SL. A comparison of home and hospital-based exercise training in heart failure: immediate and long-term effects upon physical activity level. European Journal of Cardiovascular Prevention and Rehabilitation 2011 Apr;18(2):158-166. doi: 10.1177/1741826710389389. PMID: 21450662.
251. Coxhead CE, Inskip H, Meade TW, North WR, Troup JD. Multicentre trial of physiotherapy in the management of sciatic symptoms. Lancet 1981 May 16;317(8229):1065-1068. doi: 10.1016/S0140-6736(81)92238-8. PMID: 6112444.
252. Craigie AM, MacLeod M, Barton KL, Treweek S, Anderson AS, on behalf of the WeighWell team. Supporting postpartum weight loss in women living in deprived communities: design implications for a randomised control trial. European Journal of Clinical Nutrition 2011 Aug;65(8):952-958. doi: 10.1038/ejcn.2011.56. PMID: 21559034.
253. Cramer GD, Humphreys CR, Hondras MA, McGregor M, Triano JJ. The Hmax/Mmax ratio as an outcome measure for acute low back pain. Journal of Manipulative and Physiological Therapeutics 1993 Jan;16(1):7-13. PMID: 8423429.
254. Craven JL, Evans GA, Davenport PJ, Williams RH. The evaluation of the incentive spirometer in the management of postoperative pulmonary complications. The British Journal of Surgery 1974 Oct;61(10):793-797. doi: 10.1002/bjs.1800611012. PMID: 4416262.
255. Crilly RG, Willems DA, Trenholm KJ, Hayes KC, Delaquerriere-Richardson LF. Effect of exercise on postural sway in the elderly. Gerontology 1989;35(2-3):137-143. PMID: 2792781.
256. Cronan TA, Groessl E, Kaplan RM. The effects of social support and education interventions on health care costs. Arthritis Care & Research 1997 Apr;10(2):99-110. doi: 10.1002/art.1790100205. PMID: 9313398.
257. Crotty M, Prendergast J, Battersby MW, Rowett D, Graves SE, Leach G, Giles LC. Self-management and peer support among people with arthritis on a hospital joint replacement waitinglist: a randomised controlled trial. Osteoarthritis and Cartilage 2009 Nov;17(11):1428-1433. doi: 10.1016/j.joca.2009.05.010. PMID: 19486959.
258. Crowther RG, Leicht AS, Spinks WL, Sangla K, Quigley F, Golledge J. Effects of a 6-month exercise program pilot study on walking economy, peak physiological characteristics, and walking performance in patients with peripheral arterial disease. Vascular Health and Risk Management 2012 Apr 17;8:225-232. doi: 10.2147/VHRM.S30056. PMID: 22566743.
259. Cucato GG, Chehuen MR, Costa LAR, Ritti-Dias RM, Wolosker N, Saxton JM, Forjaz CLM. Exercise prescription using the heart of claudication pain onset in patients with intermittent claudication. Clinics (Sao Paulo, Brazil) 2013 Jul;68(7):974-978. doi: 10.6061/clinics/2013(07)14. PMID: 23917662.
260. Culos-Reed SN, Carlson LE, Daroux LM, Hately-Aldous S. A pilot study of yoga for breast cancer survivors: physical and psychological benefits. Psycho-Oncology 2006 Oct;15(10):891-897. doi: 10.1002/pon.1021. PMID: 16374892.
261. Culos-Reed SN, Robinson JW, Lau H, Stephenson L, Keats M, Norris S, Kline G, Faris P. Physical activity for men receiving androgen deprivation therapy for prostate cancer: benefits from a 16-week intervention. Supportive Care in Cancer 2010 May;18(5):591-599. doi: 10.1007/s00520-009-0694-3. PMID: 19609570.
262. Cunningham DA, Rechnitzer PA, Howard JH, Donner AP. Exercise training of men at retirement: a clinical trial. Journal of Gerontology 1987 Jan;42(1):17-23. doi: 10.1093/geronj/42.1.17. PMID: 3540084.
263. Cussler EC, Teixeira PJ, Going SB, Houtkooper LB, Metcalfe LL, Blew RM, Ricketts JR, Lohman JF, Stanford VA, Lohman TG. Maintenance of weight loss in overweight middle-aged women through the internet. Obesity 2008 May;16(5):1052-1060. doi: 10.1038/oby.2008.19. PMID: 18309301.
264. da Cunha Filho IT Jr, Lim PA, Qureshy H, Henson H, Monga T, Protas EJ. Gait outcomes after acute stroke rehabilitation with supported treadmill ambulation training: a randomized controlled pilot study. Archives of Physical Medicine and Rehabilitation 2002 Sep;83(9):1258-1265. doi: 10.1053/apmr.2002.34267. PMID: 12235606.
265. Dahllof AG, Bjorntorp P, Holm J, Schersten T. Metabolic activity of skeletal muscle in patients with peripheral arterial insufficiency. European Journal of Clinical Investigation 1974 Feb;4(1):9-15. doi: 10.1111/j.1365-2362.1974.tb00365.x. PMID: 4819838.
266. dal Bello-Haas V, Florence JM, Kloos AD, Scheirbecker J, Lopate G, Hayes SM, Pioro EP, Mitsumoto H. A randomized controlled trial of resistance exercise in individuals with ALS. Neurology 2007 Jun 5;68(23):2003-2007. doi: 10.1212/01.wnl.0000264418.92308.a4. PMID: 17548549.
267. Dalal HM, Evans PH, Campbell JL, Taylor RS, Watt A, Read KL, Mourant AJ, Wingham J, Thompson DR, Pereira Gray DJ. Home-based versus hospital-based rehabilitation after myocardial infarction: a randomized trial with preference arms -- Cornwall Heart Attack Rehabilitation Management Study (CHARMS). International Journal of Cardiology 2007 Jul 10;119(2):202-211. doi: 10.1016/j.ijcard.2006.11.018. PMID: 17196274.
268. Daley AJ, Crank H, Saxton JM, Mutrie N, Coleman R, Roalfe A. Randomized trial of exercise therapy in women treated for breast cancer. Journal of Clinical Oncology 2007 May 1;25(13):1713-1721. doi: 10.1200/JCO.2006.09.5083. PMID: 17470863.
269. Daltroy LH, Iversen MD, Larson MG, Lew R, Wright E, Ryan J, Zwerling C, Fossel AH, Liang MH. A controlled trial of an educational program to prevent low back injuries. The New England Journal of Medicine 1997 Jul 31;337(5):322-328. doi: 10.1056/NEJM199707313370507. PMID: 9233870.
270. Danhauer SC, Mihalko SL, Russell GB, Campbell CR, Felder L, Daley K, Levine EA. Restorative yoga for women with breast cancer: finding from a randomized pilot study. Psycho-Oncology 2009 Apr;18(4):360-368. doi: 10.1002/pon.1503. PMID: 19242916.
271. Danielsen JM, Johnsen R, Kibsgaard SK, Hellevik E. Early aggressive exercise for postoperative rehabilitation after discectomy. Spine 2000 Apr 15;25(8):1015-1020. PMID: 10767815.
272. Dannecker C, Baur C, Ruckhaberle E, Peschers U, Jundt K, Reich A, Bauerle M, Schneider KTM. Einfluss des geburtstrainers Epi-No auf die mutterliche beckenbodenfunktion sechs monate nech entbinding -- follow-up einer prospektiven und randomisierten studie (The effect of the pelvic floor training device Epi-No on the maternal pelvic floor function six months after childbirth -- follow-up study of a randomised controlled trial) [German]. Geburtshilfe und Frauenheilkunde 2004 Nov;64(11):1192-1198.
273. Daskapan A, Arikan H, Caglar N, Tunali N, Ataman S. Comparison of supervised exercise training and home-based exercise training in chronic heart failure. Saudi Medical Journal 2005 May;26(5):842-847. PMID: 15951880.
274. David J, Modi S, Aluko AA, Robertshaw C, Farebrother J. Chronic neck pain: a comparison of acupuncture treatment and physiotherapy. British Journal of Rheumatology 1998 Oct;37(10):1118-1122. doi: 10.1093/rheumatology/37.10.1118. PMID: 9825752.
275. Davies S. Effects of continuous passive movement and plaster of Paris after internal fixation of ankle fractures. Physiotherapy 1991 Aug;77(8):516-520. doi: 10.1016/S0031-9406(10)61865-2.
276. Davis PT, Hulbert JR, Kassak KM, Meyer JJ. Comparative efficacy of conservative medical and chiropractic treatments for carpal tunnel syndrome: a randomized clinical trail. Journal of Manipulative and Physiological Therapeutics 1998 Jun;21(5):317-326. PMID: 9627862.
277. Dawson B, Cow S, Modra S, Bishop D, Stewart G. Effects of immediate post-game recovery procedures on muscle soreness, power and flexiblity levels over the next 48 hours. Journal of Science and Medicine in Sport 2005 Jun;8(2):210-221. doi: 10.1016/S1440-2440(05)80012-X. PMID: 16075781.
278. de Angelis MV, Pierfelice F, di Giovanni P, Staniscia T, Uncini A. Efficacy of a soft hand brace and a wrist splint for carpal tunnel syndrome: a randomized controlled study. Acta Neurologica Scandinavica 2009 Jan;119(1):68-74. doi: 10.1111/j.1600-0404.2008.01072.x. PMID: 18638040.
279. de Bruin ED, Murer K. Effect of additional functional exercises on balance in elderly people. Clinical Rehabilitation 2007 Feb;21(2):112-121. doi: 10.1177/0269215506070144. PMID: 17264105.
280. de Entrambasaguas M, Manez I, Girona G, Lopez-Santovena F, Poyatos YJ. Infiltracion de esteroides, ferula de muneca fonoforesis en el syndrome del tunel carpiano (Steroid injection, wrist splinting and phonophoresis in carpal tunnel syndrome) [Spanish]. Rehabilitacion [Rehabilitation] 2006;40(4):193-200.
281. de Kuiper WP, Nelson DL, White BE. Materials-based occupation versus imagery-based occupation versus rote exercise: a replication and extension. Occupational Therapy Journal of Research 1993 Summer;13(3):183-197.
282. de Macedo TM, Oliveira KM, Melo JB, de Medeiros MG, de Medeiros Filho WC, Ferreira GM, Borja RD, Campos TF, de Mendonca KM. Treinamento muscular inspiratorio em criancas com leucemia aguda: resultados preliminares (Inspiratory muscle training in patients with acute leukemia: preliminary results) [Portuguese]. Revista Paulista de Pediatria 2010 Dec;28(4):352-358. doi: 10.1590/S0103-05822010000400011.
283. de Oliveira Camargo F, Rodrigues AM, Arruda RM, Ferreira Sartori MG, Girao MJBC, Castro RA. Pelvic floor muscle training in female stress urinary incontinence: comparison between group training and individual treatment using PERFECT assessment scheme. International Urogynecology Journal and Pelvic Floor Dysfunction 2009 Dec;20(12):1455-1462. doi: 10.1007/s00192-009-0971-1. PMID: 19690792.
284. de Oliveria Melo AS, Silva JLP, Tavares JS, Barros VO, Leite DFB, Amorim MMR. Effect of a physical exercise program during pregnancy on uteroplacental and fetal blood flow and fetal growth: a randomized controlled trial. Obstetrics and Gynecology 2012 Aug;120(2 Pt 1):302-310. doi: 10.1097/AOG.0b013e31825de592. PMID: 22825089.
285. de Paul V, Moreland J, Eager T, Clase CM. The effectiveness of aerobic and muscle strength training in patients receiving hemodialysis and EPO: a randomized controlled trial. American Journal of Kidney Diseases 2002 Dec;40(6):1219-1229. doi: 10.1053/ajkd.2002.36887. PMID: 12460041.
286. de Souto Araujo ZT, de Miranda Silva Nogueira PA, Cabral EEA, de Paula dos Santos L, da Silva IS, Ferreira GMH. Effectiveness of low-intensity aquatic exercise on COPD: a randomized clinical trial. Respiratory Medicine 2012 Nov;106(11):1535-1543. doi: 10.1016/j.rmed.2012.06.022. PMID: 22809999.
287. Deale A, Chalder T, Marks I, Wessely S. Cognitive behavior therapy for chronic fatigue syndrome: a randomized controlled trial. The American Journal of Psychiatry 1997 Mar;154(3):408-414. PMID: 9054791.
288. Deligiannis A, Kouidi E, Tassoulas E, Gigis P, Tourkantonis A, Coats A. Cardiac effects of exercise rehabilitation in hemodialysis patients. International Journal of Cardiology 1999 Aug;70(3):253-266. doi: 10.1016/S0167-5273(99)00090-X. PMID: 10501340.
289. Deligiannis A, Kouidi E, Tourkantonis A. Effects of physical training on heart rate variability in patients on hemodialysis. The American Journal of Cardiology 1999 Jul 15;84(2):197-202. doi: 10.1016/S0002-9149(99)00234-9. PMID: 10426340.
290. Delneri C, di Benedetto P. Pelvic floor rehabilitation. A comparison of two methods of treatment: vaginal cones versus functional electrical stimulation. Europa Medicophysica [Mediterranean Journal of Physical and Rehabilitation Medicine] 2000;36(1):45-48.
291. Deluze C, Bosia L, Zirbs A, Chantraine A, Vischer TL. Electroacupuncture in fibromyalgia: results of a controlled trial. BMJ 1992 Nov 21;305(6864):1249-1252. PMID: 1477566.
292. Deng G, Vickers A, Yeung S, Cassileth B. Randomized, controlled trial of acupuncture for the treatment of hot flashes in breast cancer patients. Journal of Clinical Oncology 2007 Dec 10;25(35):5584-5590. doi: 10.1200/JCO.2007.12.0774. PMID: 18065731.
293. Denis M, Moffet H, Caron F, Ouellet D, Paquet J, Nolet L. Effectiveness of continuous passive motion and conventional physical therapy after total knee arthroplasty: a randomized clinical trial. Physical Therapy 2006 Feb;86(2):174-185. PMID: 16445331.
294. Depledge J, McNair PJ, Keal-Smith C, Williams M. Management of symphysis pubis dysfunction during pregnancy using exercise and pelvic support belts. Physical Therapy 2005 Dec;85(12):1290-1300. PMID: 16305268.
295. Derebery J, Giang GM, Gatchel RJ, Erickson K, Fogarty TW. Efficacy of a patient-educational booklet for neck-pain patients with workers' compensation: a randomized controlled trial. Spine 2009 Jan 15;34(2):206-213. doi: 10.1097/BRS.0b013e318193c9eb. PMID: 19139673.
296. Derrickson J, Ciesla N, Simpson N, Imle PC. A comparison of two breathing exercise programs for patients with quadriplegia. Physical Therapy 1992 Nov;72(11):763-769. PMID: 1409873.
297. Dewey KG, Lovelady CA, Nommsen-Rivers LA, McCrory MA, Lonnerdal B. A randomized study of the effects of aerobic exercise by lactating women on breast-milk volume and composition. The New England Journal of Medicine 1994 Feb 17;330(7):449-453. doi: 10.1056/NEJM199402173300701. PMID: 8289849.
298. Deyle GD, Henderson NE, Matekel RL, Ryder MG, Garber MB, Allison SC. Effectiveness of manual physical therapy and exercise in osteoarthritis of the knee: a randomized, controlled trial. Annals of Internal Medicine 2000 Feb 1;132(3):173-181. doi: 10.7326/0003-4819-132-3-200002010-00028. PMID: 10651597.
299. Deyo RA, Diehl AK, Rosenthal M. How many days of bed rest for acute low back pain? A randomized clinical trial. The New England Journal of Medicine 1986 Oct 23;315(17):1064-1070. doi: 10.1056/NEJM198610233151705. PMID: 2945109.
300. Deyo RA, Diehl AK, Rosenthal M. Reducing roentgenography use: can patient expectations be altered?. Archives of Internal Medicine 1987 Jan 1;147(1):141-145. doi: 10.1001/archinte.1987.00370010139029. PMID: 2948466.
301. Dheda K, Crawford A, Hagan G, Roberts CM. Implementation of British Thoracic Society guidelines for acute exacerbation of chronic obstructive pulmonary disease: impact on quality of life. Postgraduate Medical Journal 2004 Mar;80(941):169-171. doi: 10.1136/pgmj.2003.012831. PMID: 15016940.
302. di Stasio AJ 2nd, Jaggears FR, de Pasquale LV, Frassica FJ, Turen CH. Protected early motion versus cast immobilization in postoperative management of ankle fractures. Contemporary Orthopaedics 1994 Oct;29(4):273-277. PMID: 10150249.
303. Dijkstra-Eshuis J, van den Bos TWL, Splinter R, Bevers RFM, Zonneveld WCG, Putter H, Pelger RCM, Voorham-van der Zalm PJ. Effect of preoperative pelvic floor muscle therapy with biofeedback versus standard care on stress urinary incontinence and quality of life in men undergoing laparoscopic radical prostatectomy: a randomised control trial. Neurourology and Urodynamics 2015 Feb;34(2):144-150. doi: 10.1002/nau.22523. PMID: 24249542.
304. Dimeo FC, Stieglitz RD, Novelli-Fischer U, Fetscher S, Keul J. Effects of physical activity on the fatigue and psychologic status of cancer patients during chemotherapy. Cancer 1999 May 15;85(10):2273-2277. doi: 10.1002/(SICI)1097-0142(19990515)85:10<2273::AID-CNCR24>3.0.CO;2-B. PMID: 10326708.
305. Dimeo FC, Thomas F, Raabe-Menssen C, Propper F, Mathias M. Effect of aerobic exercise and relaxation training on fatigue and physical performance of cancer patients after surgery. A randomised controlled trial. Supportive Care in Cancer 2004;12(11):774-779. doi: 10.1007/s00520-004-0676-4. PMID: 15338385.
306. Dinc A, Kizilkaya Beji N, Yalcin O. Effect of pelvic floor muscle exercises in the treatment of urinary incontinence during pregnancy and the postpartum period. International Urogynecology Journal and Pelvic Floor Dysfunction 2009 Oct;20(10):1223-1231. doi: 10.1007/s00192-009-0929-3. PMID: 19649552.
307. Dincer U, Cakar E, Kiralp MZ, Kilac H, Dursun H. The effectiveness of conservative treatments of carpal tunnel syndrome: splinting, ultrasound, and low-level laser therapies. Photomedicine and Laser Surgery 2009 Feb;27(1):119-125. doi: 10.1089/pho.2008.2211. PMID: 19196106.
308. Dishman RK, Motl RW, Saunders R, Felton G, Ward DS, Dowda M, Pate RR. Self-efficacy partially mediates the effect of a school-based physical-activity intervention among adolescent girls. Preventive Medicine 2004 May;38(5):628-636. doi: 10.1016/j.ypmed.2003.12.007. PMID: 15066366.
309. Djavid GE, Mehrdad R, Ghasemi M, Hasan-Zadeh H, Sotoodeh-Manesh A, Pouryaghoub G. In chronic low back pain, low level laser therapy combined with exercise is more beneficial than exercise alone in the long term: a randomised trial. Australian Journal of Physiotherapy 2007;53(3):155-160. doi: 10.1016/S0004-9514(07)70022-3. PMID: 17725472.
310. Dodd JM, Turnbull D, McPhee AJ, Deussen AR, Grivell RM, Yelland LN, Crowther CA, Wittert G, Owens JA, Robinson JS, for the LIMIT Randomised Trial Group. Antenatal lifestyle advice for women who are overweight or obese: LIMIT randomised trial. BMJ 2014 Feb 10;348:g1285. doi: 10.1136/bmj.g1285. PMID: 24513442.
311. Dodd MJ, Cho MH, Miaskowski C, Painter PL, Paul SM, Cooper BA, Duda J, Krasnoff J, Bank KA. A randomized controlled trial of home-based exercise for cancer-related fatigue in women during and after chemotherapy with or without radiation therapy. Cancer Nursing 2010 Jul-Aug;33(4):245-257. doi: 10.1097/NCC.0b013e3181ddc58c. PMID: 20467301.
312. Dogra AS, Rangan A. Early mobilisation versus immobilisation of surgically treated ankle fractures. Prospective randomised control trial. Injury 1999 Aug;30(6):417-419. PMID: 10645355.
313. Dohi S, Gold MI. Comparison of two methods of postoperative respiratory care. Chest 1978 May;73(5):592-595. doi: 10.1378/chest.73.5.592. PMID: 648209.
314. Doi T, Akai M, Fujino K, Iwaya T, Kurosawa H, Hayashi K, Marui E. Effect of home exercise of quadriceps on knee osteoarthritis compared with nonsteroidal antiinflammatory drugs: a randomized controlled trial. American Journal of Physical Medicine & Rehabilitation 2008 Apr;87(4):258-269. doi: 10.1097/PHM.0b013e318168c02d. PMID: 18356618.
315. Dolan P, Greenfield K, Nelson RJ, Nelson IW. Can exercise therapy improve the outcome of microdiscectomy?. Spine 2000 Jun 15;25(12):1523-1532. PMID: 10851101.
316. Donaldson BL, Shipton EA, Inglis G, Rivett D, Frampton C. Comparison of usual surgical advice versus a nonaggravating six-month gym-based exercise rehabilitation program post-lumbar discectomy: results at one-year follow-up. The Spine Journal 2006 Jul-Aug;6(4):357-363. doi: 10.1016/j.spinee.2005.10.009. PMID: 16825039.
317. Donaldson C, Tallis R, Miller S, Sunderland A, Lemon R, Pomeroy V. Effects of conventional physical therapy and functional strength training on upper limb motor recovery after stroke: a randomized phase II study. Neurorehabilitation and Neural Repair 2009 May;23(4):389-397. doi: 10.1177/1545968308326635. PMID: 19109444.
318. Donaldson S, Romney D, Donaldson M, Skubick D. Randomized study of the application of single motor unit biofeedback training to chronic low back pain. Journal of Occupational Rehabilitation 1994 Mar;4(1):23-37. doi: 10.1007/BF02109994. PMID: 24234261.
319. Donat H, Ozcan A. Comparison of the effectiveness of two programmes on older adults at risk of falling: unsupervised home exercise and supervised group exercise. Clinical Rehabilitation 2007 Mar;21(3):273-283. doi: 10.1177/0269215506069486. PMID: 17329285.
320. Donceel P, du Bois M, Lahaye D. Return to work after surgery for lumbar disc herniation -- a rehabilitation-oriented approach in insurance medicine. Spine 1999 May 1;24(9):872-876. PMID: 10327508.
321. Donchin M, Woolf O, Kaplan L, Floman Y. Secondary prevention of low-back pain. A clinical trial. Spine 1990 Dec;15(12):1317-1320. PMID: 2149210.
322. Donesky-Cuenco D, Nguyen HQ, Paul S, Carrieri-Kohlman V. Yoga therapy decreases dyspnea-related distress and improves functional performance in people with chronic obstructive pulmonary disease: a pilot study. Journal of Alternative & Complementary Medicine 2009 Mar;15(3):225-234. doi: 10.1089/acm.2008.0389. PMID: 19249998.
323. Donnelly CM, Blaney JM, Lowe-Strong A, Rankin JP, Campbell A, McCrum-Gardner E, Gracey JH. A randomised controlled trial testing the feasibility and efficacy of a physical activity behavioural change intervention in managing fatigue with gynaecological cancer survivors. Gynecologic Oncology 2011 Sep;122(3):618-624. doi: 10.1016/j.ygyno.2011.05.029. PMID: 21689848.
324. Donnelly JE, Greene JL, Gibson CA, Smith BK, Washburn RA, Sullivan DK, du Bose K, Mayo MS, Schmelzle KH, Ryan JJ, Jacobsen DJ, Williams SL. Physical Activity Across the Curriculum (PAAC): a randomized controlled trial to promote physical activity and diminish overweight and obesity in elementary school children. Preventive Medicine 2009 Oct;49(4):336-341. doi: 10.1016/j.ypmed.2009.07.022. PMID: 19665037.
325. Dorner T, Kranz A, Zettl-Wiedner K, Ludwig C, Rieder A, Gisinger C. The effect of structured strength and balance training on cognitive function in frail, cognitive impaired elderly long-term care residents. Aging Clinical and Experimental Research 2007 Oct;19(5):400-405. PMID: 18007119.
326. Doyne EJ, Ossip-Klein DJ, Bowman ED, Osborn KM, McDougall-Wilson IB, Neimeyer RA. Running versus weight lifting in the treatment of depression. Journal of Consulting and Clinical Psychology 1987 Oct;55(5):748-754. doi: 10.1037/0022-006X.55.5.748. PMID: 3454786.
327. Drory VE, Goltsman E, Reznik JG, Mosek A, Korczyn AD. The value of muscle exercise in patients with amyotrophic lateral sclerosis. Journal of the Neurological Sciences 2001;191(1-2):133-137. PMID: 11677004.
328. Drouin JS, Armstrong H, Krause S, Orr J, Birk TJ, Hryniuk WM, Hryniuk LE. Effects of aerobic exercise training on peak aerobic capacity, fatigue, and psychological factors during radiation for breast cancer. Rehabilitation Oncology 2005;23(1):11-17.
329. Dubbelman Y, Groen J, Wildhagen M, Rikken B, Bosch R. The recovery of urinary continence after radical retropubic prostatectomy: a randomized trial comparing the effect of physiotherapist-guided pelvic floor muscle exercises with guidance by an instruction folder only. BJU International 2010 Aug;106(4):515-522. doi: 10.1111/j.1464-410X.2010.09159.x. PMID: 20201841.
330. Dumoulin C, Lemieux M, Bourbonnais D, Gravel D, Bravo G, Morin M. Physiotherapy for persistent postnatal stress urinary incontinence: a randomized controlled trial. Obstetrics and Gynecology 2004 Sep;104(3):504-510. doi: 10.1097/01.AOG.0000135274.92416.62. PMID: 15339760.
331. Duncan P, Richards L, Wallace D, Stoker-Yates J, Pohl P, Luchies C, Ogle A, Studenski S. A randomized, controlled pilot study of a home-based exercise program for individuals with mild and moderate stroke. Stroke 1998 Oct;29(10):2055-2060. PMID: 9756581.
332. Duncan P, Studenski S, Richards L, Gollub S, Lai SM, Reker D, Perera S, Yates J, Koch V, Rigler S, Johnson D. Randomized clinical trial of therapeutic exercise in subacute stroke. Stroke 2003 Sep;34(9):2173-2180. doi: 10.1161/01.STR.0000083699.95351.F2. PMID: 12920254.
333. Dunn AL, Trivedi MH, Kampert JB, Clark CG, Chambliss HO. Exercise treatment for depression: efficacy and dose response. American Journal of Preventive Medicine 2005 Jan;28(1):1-8. doi: 10.1016/j.amepre.2004.09.003. PMID: 15626549.
334. Duymaz T, Sindel D, Kesiktas N, Muslumanoglu L. Efficacy of some combined conservative methods in the treatment of carpal tunnel syndrome: a randomized controlled clinical and electrophysiological trial. Turkish Journal of Rheumatology 2012;27(1):38-46. doi: 10.5606/tjr.2012.005.
335. Ebenbichler GR, Resch KL, Nicolakis P, Wiesinger GF, Uhl F, Ghanem AH, Fialka V. Ultrasound treatment for treating the carpal tunnel syndrome: randomised "sham" controlled trial. BMJ 1998 Mar 7;316(7133):731-735. PMID: 9529407.
336. Ebersbach G, Edler D, Kaufhold O, Wissel J. Whole body vibration versus conventional physiotherapy to improve balance and gait in Parkinson's disease. Archives of Physical Medicine and Rehabilitation 2008 Mar;89(3):399-403. doi: 10.1016/j.apmr.2007.09.031. PMID: 18295614.
337. Ebrahim S, Thompson PW, Baskaran V, Evans K. Randomized placebo-controlled trial of brisk walking in the prevention of postmenopausal osteoporosis. Age and Ageing 1997 Jul;26(4):253-260. PMID: 9271287.
338. Edwards H, Courtney M, Finlayson K, Shuter P, Lindsay E. A randomised controlled trial of a community nursing intervention: improved quality of life and healing for clients with chronic leg ulcers. Journal of Clinical Nursing 2009 Jun;18(11):1541-1549. doi: 10.1111/j.1365-2702.2008.02648.x. PMID: 19490293.
339. Eggen MH, Stuge B, Mowinckel P, Jensen KS, Hagen KB. Can supervised group exercises including ergonomic advice reduce the prevalence and severity of low back pain and pelvic girdle pain in pregnancy? A randomized controlled trial. Physical Therapy 2012 Jun;92(6):781-790. doi: 10.2522/ptj.20110119. PMID: 22282770.
340. Egol KA, Dolan R, Koval KJ. Functional outcome of surgery for fractures of the ankle. A prospective, randomised comparison of management in a cast or a functional brace. Journal of Bone and Joint Surgery -- British Volume 2000 Mar;82-B(2):246-249. PMID: 10755435.
341. Eich HJ, Mach H, Werner C, Hesse S. Aerobic treadmill plus Bobath walking training improves walking in subacute stroke: a randomized controlled trial. Clinical Rehabilitation 2004 Sep;18(6):640-651. doi: 10.1191/0269215504cr779oa. PMID: 15473116.
342. Eidemak I, Haaber AB, Feldt-Rasmussen B, Kanstrup IL, Strandgaard S. Exercise training and the progression of chronic renal failure. Nephron 1997;75(1):36-40. PMID: 9031268.
343. Ekdahl L, Petersson K. Acupuncture treatment of pregnant women with low back and pelvic pain -- an intervention study. Scandinavian Journal of Caring Sciences 2010 Mar;24(1):175-182. doi: 10.1111/j.1471-6712.2009.00704.x. PMID: 20102541.
344. Elden H, Fagevik-Olsen M, Ostgaard HC, Stener-Victorin E, Hagberg H. Acupuncture as an adjunct to standard treatment for pelvic girdle pain in pregnant women: randomised double-blinded controlled trial comparing acupuncture with non-penetrating sham acupuncture. BJOG 2008 Dec;115(13):1655-1668. doi: 10.1111/j.1471-0528.2008.01904.x. PMID: 18947338.
345. Elden H, Ladfors L, Olsen MF, Ostgaard HC, Hagberg H. Effects of acupuncture and stabilising exercises as adjunct to standard treatment in pregnant women with pelvic girdle pain: randomised single blind controlled trial. BMJ 2005 Apr 2;330(7494):761-765. doi: 10.1136/bmj.38397.507014.E0. PMID: 15778231.
346. Emami A, Petren-Mallmin M, Larsson S. No effect of low-intensity ultrasound on healing time of intramedullary fixed tibial fractures. Journal of Orthopaedic Trauma 1999 May;13(4):252-257. PMID: 10342350.
347. Emery CF, Gatz M. Psychological and cognitive effects of an exercise program for community-residing older adults. The Gerontologist 1990 Apr;30(2):184-188. doi: 10.1093/geront/30.2.184. PMID: 2347498.
348. Englund U, Littbrand H, Sondell A, Pettersson U, Bucht G. A 1-year combined weight-bearing training program is beneficial for bone mineral density and neuromuscular function in older women. Osteoporosis International 2005 Sep;16(9):1117-1123. doi: 10.1007/s00198-004-1821-0. PMID: 16133653.
349. Engstrom C, Persson L, Larsson S, Sullivan M. Long-term effects of a pulmonary rehabilitation programme in outpatients with chronic obstructive pulmonary disease: a randomized controlled study. Scandinavian Journal of Rehabilitation Medicine 1999 Dec;31(4):207-213. PMID: 10599897.
350. Eqwu MO. Relative therapeutic efficacy of some vertebral mobolization techniques in the management of unilateral cervical spondylosis: a comparitive study. Journal of Physical Therapy Science 2008 Jun;20(2):103-108. doi: 10.1589/jpts.20.103.
351. Erdogmus CB, Resch KL, Sabitzer R, Muller H, Nuhr M, Schoggl A, Posch M, Osterode W, Ungersbock K, Ebenbichler GR. Physiotherapy-based rehabilitation following disc herniation operation: results of a randomized clinical trial. Spine 2007 Sep 1;32(19):2041-2049. doi: 10.1097/BRS.0b013e318145a386. PMID: 17762803.
352. Escortell Mayor E, Lebrijo Perez G, Perez Martin Y, Asunsolo del Barco A, Riesgo Fuertes R, Saa Requejo C, on Behalf of the TEMA-TENS Group. Ensayo clinico aleatorizado en pacientes con cervicalgia mecanica en atencion primaria: terapia manual frente a electroestimulacion nerviosa transcutanea (Randomised clinical trial for primary care patients with neck pain: manual therapy versus electrical stimulation) [Spanish]. Atencion Primaria 2008 Jul 1;40(7):337-343. PMID: 18620635.
353. Escortell-Mayor E, Riesgo-Fuertes R, Garrido-Elustondo S, Asunsolo-del Barco A, Diaz-Pulido B, Blanco-Diaz M, Bejerano-Alvarez E. Primary care randomized clinical trial: manual therapy effectiveness in comparison with TENS in patients with neck pain. Manual Therapy 2011 Feb;16(1):66-73. doi: 10.1016/j.math.2010.07.003. PMID: 20691631.
354. Ettinger WH Jr, Burns R, Messier SP, Applegate W, Rejeski WJ, Morgan T, Shumaker S, Berry MJ, O'Toole M, Monu J, Craven T. A randomized trial comparing aerobic exercise and resistance exercise with a health education program in older adults with knee osteoarthritis. The Fitness Arthritis and Seniors Trial (FAST). JAMA 1997 Jan 1;277(1):25-31. doi: 10.1001/jama.1997.03540250033028. PMID: 8980206.
355. Evans DP, Burke MS, Lloyd KN, Roberts EE, Roberts GM. Lumbar spinal manipulation on trial. Part I -- clinical assessment. Rheumatology and Rehabilitation 1978 Feb;17(1):46-53. doi: 10.1093/rheumatology/17.1.46. PMID: 153574.
356. Ewart CK, Young DR, Hagberg JM. Effects of school-based aerobic exercise on blood pressure in adolescent girls at risk for hypertension. American Journal of Public Health 1998 Jun;88(6):949-951. PMID: 9618627.
357. Ewings P, Spencer S, Marsh H, O'Sullivan M. Obstetric risk factors for urinary incontinence and preventative pelvic floor exercises: cohort study and nested randomized controlled trial. Journal of Obstetrics and Gynaecology 2005 Aug;25(6):558-564. doi: 10.1080/01443610500231435. PMID: 16234140.
358. Faas A, Chavannes AW, van Eijk JT, Gubbels JW. A randomized, placebo-controlled trial of exercise therapy in patients with acute low back pain. Spine 1993 Sep 1;18(11):1388-1395. PMID: 8235809.
359. Faas A, van Eijk JT, Chavannes AW, Gubbels JW. A randomized trial of exercise therapy in patients with acute low back pain. Efficacy on sickness absence. Spine 1995 Apr 15;20(8):941-947. PMID: 7644960.
360. Faber MJ, Bosscher RJ, Chin A Paw MJ, van Wieringen PC. Effects of exercise programs on falls and mobility in frail and pre-frail older adults: a multicenter randomized controlled trial. Archives of Physical Medicine and Rehabilitation 2006 Jul;87(7):885-896. doi: 10.1016/j.apmr.2006.04.005. PMID: 16813773.
361. Fabre C, Chamari K, Mucci P, Masse-Biron J, Prefaut C. Improvement of cognitive function by mental and/or individualized aerobic training in healthy elderly subjects. International Journal of Sports Medicine 2002 Aug;23(6):415-421. doi: 10.1055/s-2002-33735. PMID: 12215960.
362. Fagan DJ, Evans A, Ghandour A, Prabhkaran P, Clay NRC. A controlled clinical trial of postoperative hand elevation at home following day-case surgery. Journal of Hand Surgery - British Volume 2004 Oct;29(5):458-460. doi: 10.1016/J.JHSB.2004.04.008. PMID: 15336749.
363. Fairbank J, Frost H, Wilson-MacDonald J, Yu LM, Barker K, Collins R, for the Spine Stabilisation Trial Group. Randomised controlled trial to compare surgical stabilisation of the lumbar spine with an intensive rehabilitation programme for patients with chronic low back pain: the MRC spine stabilisation trial. BMJ 2005 May 28;330(7502):1233-1239. doi: 10.1136/bmj.38441.620417.8F. PMID: 15911537.
364. Fanelli A, Cabral ALB, Neder JA, Martins MA, Carvalho CRF. Exercise training on disease control and quality of life in asthmatic children. Medicine and Science in Sports and Exercise 2007 Sep;39(9):1474-1480. doi: 10.1249/mss.0b013e3180d099ad. PMID: 17805077.
365. Farid R, Jabbari Azad F, Ebrahimi Atri A, Baradaran Rahimi M, Khaledan A, Talaei-Khoei M, Ghafari J, Ghasemi R. Effect of aerobic exercise training on pulmonary function and tolerance of activity in asthmatic patients. Iranian Journal of Allergy, Asthma, and Immunology 2005 Sep;4(3):133-138. PMID: 17301436.
366. Farina S, Casarotto M, Benelle M, Tinazzi M, Fiaschi A, Goldoni M, Smania N. A randomized controlled study on the effect of two different treatments (FREMS and TENS) in myofascial pain syndrome. Europa Medicophysica [Mediterranean Journal of Physical and Rehabilitation Medicine] 2004 Dec;40(4):293-301. PMID: 16175154.
367. Fauno P, Kalund S, Andreasen I, Jorgensen U. Soreness in lower extremities and back is reduced by use of shock absorbing heel inserts. International Journal of Sports Medicine 1993;14(5):288-290. doi: 10.1055/s-2007-1021179. PMID: 8365838.
368. Felicissimo MF, Carneiro MM, Saleme CS, Pinto RZ, da Fonseca AM, da Silva-Filho AL. Intensive supervised versus unsupervised pelvic floor muscle training for the treatment of stress urinary incontinence: a randomized comparative trial. International Urogynecology Journal 2010 Jul;21(7):835-840. doi: 10.1007/s00192-010-1125-1. PMID: 20179901.
369. Ferguson KL, McKey PL, Bishop KR, Kloen P, Verheul JB, Dougherty MC. Stress urinary incontinence: effect of pelvic muscle exercise. Obstetrics and Gynecology 1990 Apr;75(4):671-675. PMID: 2314786.
370. Fernandes L, Storheim K, Sandvik L, Nordsletten L, Risberg MA. Efficacy of patient education and supervised exercise versus patient education alone in patients with hip osteoarthritis: a single blind randomized clinical trial. Osteoarthritis and Cartilage 2010 Oct;18(10):1237-1243. doi: 10.1016/j.joca.2010.05.015. PMID: 20633669.
371. Fernandez AM, Pascual J, Ferrando C, Arnal A, Vergara I, Sevila V. Home-based pulmonary rehabilitation in very severe COPD: is it safe and useful?. Journal of Cardiopulmonary Rehabilitation and Prevention 2009 Sep-Oct;29(5):325-331. doi: 10.1097/HCR.0b013e3181ac7b9d. PMID: 19561524.
372. Fernandez-de-las-Penas C, Alonso-Blanco C, Fernandez-Carnero J, Carlos Miangolarra-Page J. The immediate effect of ischemic compression technique and transverse friction massage on tenderness of active and latent myofascial trigger points: a pilot study. Journal of Bodywork and Movement Therapies 2006 Jan;10(1):3-9. doi: 10.1016/j.jbmt.2005.05.003.
373. Fernandez-de-las-Penas C, Cleland JA, Huijbregts P, Palomeque-del-Cerro L, Gonzalez-Iglesias J. Repeated applications of thoracic spine thrust manipulation do not lead to tolerance in patients presenting with acute mechanical neck pain: a secondary analysis. The Journal of Manual & Manipulative Therapy 2009;17(3):154-162. PMID: 20046622.
374. Fernandez-de-las-Penas C, Fernandez-Carnero J, Fernandez AP, Lomas-Vega R, Miangolarra-Page JC. Dorsal manipulation in whiplash injury treatment: a randomized controlled trial. Journal of Whiplash & Related Disorders 2004;3(2):55-72.
375. Ferrara A, Hedderson MM, Albright CL, Ehrlich SF, Quesenberry CP, Peng T, Feng J, Ching J, Crites Y. A pregnancy and postpartum lifestyle intervention in women with gestational diabetes mellitus reduces diabetes risk factors: a feasibility randomized control trial. Diabetes Care 2011 Jul;34(7):1519-1525. doi: 10.2337/dc10-2221. PMID: 21540430.
376. Ferrari R, Rowe BH, Majumdat SR, Cassidy JD, Blitz S, Wright SC, Russell AS. Simple educational intervention to improve the recovery from acute whiplash: results of a randomised, controlled trial. Academic Emergency Medicine 2005 Aug;12(8):699-706. doi: 10.1197/j.aem.2005.03.531. PMID: 16079422.
377. Ferreira ML, Ferreira PH, Latimer J, Herbert RD, Hodges PW, Jennings MD, Maher CG, Refshauge KM. Comparison of general exercise, motor control exercise and spinal manipulative therapy for chronic low back pain: a randomized trial. Pain 2007 Sep;131(1-2):31-37. doi: 10.1016/j.pain.2006.12.008. PMID: 17250965.
378. Ferreira PE, Rodrigues AJ, Evora PR. Effects of an inspiratory muscle rehabilitation program in the postoperative period of cardiac surgery. Arquivos Brasileiros de Cardiologia 2009 Apr;92(4):261-268. doi: 10.1590/S0066-782X2009000400005. PMID: 19565135.
379. Feys HM, de Weerdt WJ, Selz BE, Cox Steck GA, Spichiger R, Vereeck LE, Putman KD, van Hoydonck GA. Effect of a therapeutic intervention for the hemiplegic upper limb in the acute phase after stroke: a single-blind, randomized, controlled multicenter trial. Stroke 1998 Apr;29(4):785-792. PMID: 9550512.
380. Fialka V, Preisinger E, Bohler A. Zur physikalischen diagnostik und physikalischer therapie der distorsio columnae vertebrailis cervicalis (Physical diagnosis and physical therapy of whiplash injury) [German]. Zeitschrift fuer Physikalische Medizin, Balneologie, Medizinische Klimatologie 1989;18(6):390-397.
381. Fiatarone MA, O'Neill EF, Ryan ND, Clements KM, Solares GR, Nelson ME, Roberts SB, Kehayias JJ, Lipsitz LA, Evans WJ. Exercise training and nutritional supplementation for physical frailty in very elderly people. The New England Journal of Medicine 1994 Jun 23;330(25):1769-1775. doi: 10.1056/NEJM199406233302501. PMID: 8190152.
382. Field T, Diego M, Cullen C, Hartshorn K, Gruskin A, Hernandez-Reif M, Sunshine W. Carpal tunnel syndrome symptoms are lessened following massage therapy. Journal of Bodywork and Movement Therapies 2004 Jan;8(1):9-14. doi: 10.1016/S1360-8592(03)00064-0.
383. Filiz M, Cakmak A, Ozcan E. The effectiveness of exercise programmes after lumbar disc surgery: a randomized controlled study. Clinical Rehabilitation 2005 Jan;19(1):4-11. doi: 10.1191/0269215505cr836oa. PMID: 15704503.
384. Fillion L, Gagnon P, Leblond F, Gelinas C, Savard J, Dupuis R, Duval K, Larochelle M. A brief intervention for fatigue management in breast cancer survivors. Cancer Nursing 2008 Mar-Apr;31(2):145-159. doi: 10.1097/01.NCC.0000305698.97625.95. PMID: 18490891.
385. Filocamo MT, li Marzi V, del Popolo G, Cecconi F, Marzocco M, Tosto A, Nicita G. Effectiveness of early pelvic floor rehabilitation treatment for post-prostatectomy incontinence. European Urology 2005 Nov;48(5):734-738. doi: 10.1016/j.eururo.2005.06.004. PMID: 16002204.
386. Finestone A, Novack V, Farfel A, Berg A, Amir H, Milgrom C. A prospective study of the effect of foot orthoses composition and fabrication on comfort and the incidence of overuse injuries. Foot & Ankle International 2004 Jul;25(7):462-466. doi: 10.1177/107110070402500704. PMID: 15319103.
387. Finsen V, Andersen K, Russwurm H. No advantage from splinting the wrist after open carpal tunnel release. A randomized study of 82 wrists. Acta Orthopaedica Scandinavica 1999 Jun;70(3):288-292. PMID: 10429608.
388. Finsen V, Saetermo R, Kibsgaard L, Farran K, Engebretsen L, Bolz KD, Benum P. Early postoperative weight-bearing and muscle activity in patients who have a fracture of the ankle. Journal of Bone and Joint Surgery -- American Volume 1989 Jan;71(1):23-27. PMID: 2492286.
389. Fitts SS, Guthrie MR, Blagg CR. Exercise coaching and rehabilitation counseling improve quality of life for predialysis and dialysis patients. Nephron 1999 Jun;82(2):115-121. doi: 10.1159/000045386. PMID: 10364702.
390. Flansbjer U-B, Miller M, Downham D, Lexell J. Progressive resistance training after stroke: effects on muscle strength, muscle tone, gait performance and perceived participation. Journal of Rehabilitation Medicine 2008 Jan;40(1):42-48. doi: 10.2340/16501977-0129. PMID: 18176736.
391. Floratos DL, Sonke GS, Rapidou CA, Alivizatos GJ, Deliveliotis C, Constantinides CA, Theodorou C. Biofeedback versus verbal feedback as learning tools for pelvic muscle exercises in the early management of urinary incontinence after radical prostatectomy. BJU International 2002 May;89(7):714-719. doi: 10.1046/j.1464-410X.2002.02721.x. PMID: 11966630.
392. Fluge T, Richter J, Fabel H, Zysno E, Weller E, Wagner TO. (Long-term effects of breathing exercises and yoga in patients with bronchial asthma) [German]. Pneumologie 1994 Jul;48(7):484-490. PMID: 7937658.
393. Foley A, Halbert J, Hewitt T, Crotty M. Does hydrotherapy improve strength and physical function in patients with osteoarthritis--a randomised controlled trial comparing a gym based and a hydrotherapy based strengthening programme. Annals of the Rheumatic Diseases 2003 Dec;62(12):1162-1167. doi: 10.1136/ard.2002.005272. PMID: 14644853.
394. Foley LS, Prapavessis H, Osuch EA, de Pace JA, Murphy BA, Podolinsky NJ. An examination of potential mechanisms for exercise as a treatment for depression: a pilot study. Mental Health and Physical Activity 2008 Dec;1(2):69-73. doi: 10.1016/j.mhpa.2008.07.001.
395. Foley-Nolan D, Barry C, Coughlan RJ, O'Connor P, Roden D. Pulsed high frequency (27MHz) electromagnetic therapy for persistent neck pain. A double blind, placebo-controlled study of 20 patients. Orthopedics 1990 Apr;13(4):445-451. PMID: 2185460.
396. Foley-Nolan D, Moore K, Codd M, Barry C, O'Connor P, Coughlan RJ. Low energy high frequency pulsed electromagnetic therapy for acute whiplash injuries. A double blind randomized controlled study. Scandinavian Journal of Rehabilitation Medicine 1992;24(1):51-59. PMID: 1604262.
397. Foroughi N, Smith RM, Lange AK, Baker MK, Fiatarone Singh MA, Vanwanseele B. Lower limb muscle strengthening does not change frontal plane moments in women with knee osteoarthritis: a randomized controlled trial. Clinical Biomechanics 2011 Feb;26(2):167-174. doi: 10.1016/j.clinbiomech.2010.08.011. PMID: 20888096.
398. Franke J, Goldhahn S, Audige L, Kohler H, Wentzensen A. The dynamic vacuum orthosis: a functional and economical benefit?. International Orthopaedics 2008 Apr;32(2):153-158. doi: 10.1007/s00264-006-0306-5. PMID: 17206496.
399. Franke JJ, Gilbert WB, Grier J, Koch MO, Shyr Y, Smith JA Jr. Early post-prostatectomy pelvic floor biofeedback. The Journal of Urology 2000 Jan;163(1):191-193. PMID: 10604344.
400. Fransen M, Crosbie J, Edmonds J. Physical therapy is effective for patients with osteoarthritis of the knee: a randomized controlled clinical trial. The Journal of Rheumatology 2001 Jan;28(1):156-164. PMID: 11196518.
401. Fransen M, Nairn L, Winstanley J, Lam P, Edmonds J. Physical activity for osteoarthritis management: a randomized controlled clinical trial evaluating hydrotherapy or Tai Chi classes. Arthritis Care & Research 2007 Apr 15;57(3):407-414. doi: 10.1002/art.22621. PMID: 17443749.
402. Frawley HC, Phillips BA, Bo K, Galea MP. Physiotherapy as an adjunct to prolapse surgery: an assessor-blinded randomized controlled trial. Neurourology and Urodynamics 2010 Jun;29(5):719-725. doi: 10.1002/nau.20828. PMID: 19816918.
403. Fremont J, Wilcoxon Craighead L. Aerobic exercise and cognitive therapy in the treatment of dysphoric moods. Cognitive Therapy and Research 1987 Apr;11(2):241-251. doi: 10.1007/BF01183268.
404. French HP, Cusack T, Brennan MA, Caffrey MA, Conroy R, Cuddy MV, Fitzgerald OM, Gilsenan MC, Kane D, O'Connell PG, White MB, McCarthy GM. Exercise and Manual Therapy Arthritis Research Trial (EMPART) for osteoarthritis of the hip: a multicentre randomised controlled trial. Archives of Physical Medicine and Rehabilitation 2013 Feb;94(2):302-314. doi: 10.1016/j.apmr.2012.09.030. PMID: 23084955.
405. Frey S, Mir AR, Lucas M. Visceral protein status and caloric intake in exercising versus nonexercising individuals with end-stage renal disease. Journal of Renal Nutrition 1999 Apr;9(2):71-77. doi: 10.1016/S1051-2276(99)90003-1. PMID: 10089262.
406. Friedrich M, Gittler G, Halberstadt Y, Cermak T, Heiller I. Combined exercise and motivation program: effect on the compliance and level of disability of patients with chronic low back pain: a randomized controlled trial. Archives of Physical Medicine and Rehabilitation 1998 May;79(5):475-487. doi: 10.1016/S0003-9993(98)90059-4. PMID: 9596385.
407. Frisk J, Carlhall S, Kallstrom AC, Lindh-Astrand L, Malmstrom A, Hammar M. Long-term follow-up of acupuncture and hormone therapy on hot flushes in women with breast cancer: a prospective, randomized, controlled multicenter trial. Climacteric 2008 Apr;11(2):166-174. doi: 10.1080/13697130801958709. PMID: 18365859.
408. Fritz JM, Lindsay W, Matheson JW, Brennan GP, Hunter SJ, Moffit SD, Swalberg A, Rodriquez B. Is there a subgroup of patients with low back pain likely to benefit from mechanical traction? Results of a randomized clinical trial and subgrouping analysis. Spine 2007 Dec 15;32(26):E793-E800. doi: 10.1097/BRS.0b013e31815d001a. PMID: 18091473.
409. Frost H, Lamb SE, Doll HA, Carver PT, Stewart-Brown S. Randomised controlled trial of physiotherapy compared with advice for low back pain. BMJ 2004 Sep 25;329(7468):708-713. doi: 10.1136/bmj.38216.868808.7C. PMID: 15377573.
410. Fryer G, Hodgson L. The effect of manual pressure release on myofascial trigger points in the upper trapezius muscle. Journal of Bodywork and Movement Therapies 2005 Oct;9(4):248-255. doi: 10.1016/j.jbmt.2005.02.002.
411. Gabriele JM, Carpenter BD, Tate DF, Fisher EB. Directive and nondirective e-coach support for weight loss in overweight adults. Annals of Behavioral Medicine 2011 Apr;41(2):252-263. doi: 10.1007/s12160-010-9240-2. PMID: 21108032.
412. Gallie M, Pourghazi S, Grant JM. A randomized trial of pulsed electromagnetic energy compared with ice-packs for the relief of postnatal perineal pain. Journal of the Association of Chartered Physiotherapists in Women's Health 2003;(93):10-14.
413. Gallo ML, Staskin DR. Cues to action: pelvic floor muscle exercise compliance in women with stress urinary incontinence. Neurourology and Urodynamics 1997;16(3):167-177. doi: 10.1002/(SICI)1520-6777(1997)16:3<167::AID-NAU6>3.0.CO;2-C. PMID: 9136139.
414. Galvao DA, Taaffe DR, Spry N, Joseph D, Newton RU. Combined resistance and aerobic exercise program reverses muscle loss in men undergoing androgen suppression therapy for prostate cancer without bone metastases: a randomized controlled trial. Journal of Clinical Oncology 2010 Jan 10;28(2):340-347. doi: 10.1200/JCO.2009.23.2488. PMID: 19949016.
415. Galvin R, Cusack T, O'Grady E, Murphy TB, Stokes E. Family-mediated exercise intervention (FAME): evaluation of a novel form of exercise delivery after stroke. Stroke 2011 Mar;42(3):681-686. doi: 10.1161/STROKEAHA.110.594689. PMID: 21233462.
416. Gameiro MO, Moreira EH, Gameiro FO, Moreno JC, Padovani CR, Amaro JL. Vaginal weight cone versus assisted pelvic floor muscle training in the treatment of female urinary incontinence. A prospective, single-blind, randomized trial. International Urogynecology Journal 2010 Apr;21(4):395-399. doi: 10.1007/s00192-009-1059-7. PMID: 20052573.
417. Gardner AW, Katzel LI, Sorkin JD, Goldberg AP. Effects of long-term exercise rehabilitation on claudication distances in patients with peripheral arterial disease: a randomized controlled trial. Journal of Cardiopulmonary Rehabilitation 2002 May-Jun;22(3):192-198. PMID: 12042688.
418. Gardner AW, Montgomery PS, Parker DE. Optimal exercise program length for patients with claudication. Journal of Vascular Surgery 2012 May;55(5):1346-1354. doi: 10.1016/j.jvs.2011.11.123. PMID: 22459748.
419. Gardner AW, Parker DE, Montgomery PS, Scott KJ, Blevins SM. Efficacy of quantified home-based exercise and supervised exercise in patients with intermittent claudication: a randomized controlled trial. Circulation 2011 Feb 8;123(5):491-498. doi: 10.1161/CIRCULATIONAHA.110.963066. PMID: 21262997.
420. Gardner LI Jr, Dziados JE, Jones BH, Brundage JF, Harris JM, Sullivan R, Gill P. Prevention of lower extremity stress fractures: a controlled trial of a shock absorbent insole. American Journal of Public Health 1988 Dec;78(12):1563-1567. PMID: 3056045.
421. Garfinkel MS, Singhal A, Katz WA, Allan DA, Reshetar R, Schumacher HR Jr. Yoga-based intervention for carpal tunnel syndrome: a randomized trial. JAMA 1998 Nov 11;280(18):1601-1603. doi: 10.1001/jama.280.18.1601. PMID: 9820263.
422. Garshasbi A, Faghih Zadeh S. The effect of exercise on the intensity of low back pain in pregnant women. International Journal of Gynaecology and Obstetrics 2005 Mar;88(3):271-275. doi: 10.1016/j.ijgo.2004.12.001. PMID: 15733880.
423. Gary RA, Dunbar SB, Higgins MK, Musselman DL, Smith AL. Combined exercise and cognitive behavioral therapy improves outcomes in patients with heart failure. Journal of Psychosomatic Research 2010 Aug;69(2):119-131. doi: 10.1016/j.jpsychores.2010.01.013. PMID: 20624510.
424. Gatchel RJ, Polatin PB, Noe C, Gardea M, Pulliam C, Thompson J. Treatment- and cost-effectiveness of early intervention for acute low-back pain patients: a one-year prospective study. Journal of Occupational Rehabilitation 2003 Mar;13(1):1-9. doi: 10.1023/A:1021823505774. PMID: 12611026.
425. Gay PC, Hubmayr RD, Stroetz RW. Efficacy of nocturnal nasal ventilation in stable, severe chronic obstructive pulmonary disease during a 3-month controlled trial. Mayo Clinic Proceedings 1996 Jun;71(6):533-542. doi: 10.4065/71.6.533. PMID: 8642881.
426. Gelin J, Jivegard L, Taft C, Karlsson J, Sullivan M, Dahllof AG, Sandstrom R, Arfvidsson B, Lundholm K. Treatment efficacy of intermittent claudication by surgical intervention, supervised physical exercise training compared to no treatment in unselected randomised patients I: one year results of functional and physiological improvements. European Journal of Vascular and Endovascular Surgery 2001 Aug;22(2):107-113. doi: 10.1053/ejvs.2001.1413. PMID: 11472042.
427. Gemmell H, Allen A. Relative immediate effect of ischaemic compression and activator trigger point therapy on active upper trapezius trigger points: a randomised trial. Clinical Chiropractic 2008 Dec;11(4):175-181. doi: 10.1016/j.clch.2009.01.007.
428. Gemmell H, Miller P. Relative effectiveness and adverse effects of cervical manipulation, mobilisation and the activator instrument in patients with sub-acute non-specific neck pain: results from a stopped randomised trial. Chiropractic & Osteopathy 2010 Jul 9;18(20):Epub. doi: 10.1186/1746-1340-18-20. PMID: 20618936.
429. Gemmell H, Miller P, Nordstrom H. Immediate effect of ischaemic compression and trigger point pressure release on neck pain and upper trapezius trigger points: a randomised, controlled trial. Clinical Chiropractic 2008 Mar;11(1):30-36. doi: 10.1016/j.clch.2007.09.001.
430. Gentile DA, Welk G, Eisenmann JC, Reimer RA, Walsh DA, Russell DW, Callahan R, Walsh M, Strickland S, Fritz K. Evaluation of a multiple ecological level child obesity prevention program: Switch What you Do, View, and Chew. BMC Medicine 2009 Sep 18;7(49):Epub. doi: 10.1186/1741-7015-7-49. PMID: 19765270.
431. Geraerts I, van Poppel H, Devoogdt N, Joniau S, van Cleynenbreugel B, de Groef A, van Kampen M. Influence of preoperative and postoperative pelvic floor muscle training (PFMT) compared with postoperative PFMT on urinary incontinence after radical prostatectomy: a randomized controlled trial. European Urology 2013 Nov;64(5):766-772. doi: 10.1016/j.eururo.2013.01.013. PMID: 23357349.
432. Ghoniem GM, van Leeuwen JS, Elser DM, Freeman RM, Zhao YD, Yalcin I, Bump RC, Duloxetine/Pelvic Floor Muscle Training Clinical Trial Group. A randomized controlled trial of duloxetine alone, pelvic floor muscle training alone, combined treatment and no active treatment in women with stress urinary incontinence. The Journal of Urology 2005 May;173(5):1647-1653. doi: 10.1097/01.ju.0000154167.90600.c6. PMID: 15821528.
433. Ghroubi S, Elleuch H, Baklouti S, Elleuch MH. Les lombalgiques chroniques et manipulations vertebrales. Etude prospective a propos de 64 cas (Chronic low back pain and vertebral manipulation) [French]. Annales de Readaptation et de Medecine Physique 2007 Oct;50(7):570-576. doi: 10.1016/j.annrmp.2007.02.012. PMID: 17382426.
434. Ghroubi S, Kharrat O, Chaari M, Ben Ayed B, Guermazi M, Elleuch MH. Apport du traitement conservateur dans la prise en charge du prolapsus urogenital de bas grade. Le devenir apres deux ans (Effect of conservative treatment in the management of low-degree urogenital prolapse) [French]. Annales de Readaptation et de Medecine Physique 2008 Mar;51(2):96-102. doi: 10.1016/j.annrmp.2007.11.002. PMID: 18242753.
435. Gibbons CE, Solan MC, Ricketts DM, Patterson M. Cryotherapy compared with Robert Jones bandage after total knee replacement: a prospective randomized trial. International Orthopaedics 2001 Aug;25(4):250-252. doi: 10.1007/s002640100227. PMID: 11561502.
436. Gibson T, Grahame R, Harkness J, Woo P, Blagrave P, Hills R. Controlled comparison of short-wave diathermy treatment with osteopathic treatment in non-specific low back pain. Lancet 1985 Jun 1;325(8440):1258-1261. doi: 10.1016/S0140-6736(85)92323-2. PMID: 2860453.
437. Gilbert JR, Taylor DW, Hildebrand A, Evans C. Clinical trial of common treatments for low back pain in family practice. British Medical Journal 1985 Sep 21;291(6498):791-794. PMID: 2931153.
438. Giles LG, Muller R. Chronic spinal pain syndromes: a clinical pilot trial comparing acupuncture, a nonsteroidal anti-inflammatory drug, and spinal manipulation. Journal of Manipulative and Physiological Therapeutics 1999 Jul-Aug;22(6):376-381. PMID: 10478769.
439. Gillies E, Aitchison T, MacDonald J, Grant S. Outcomes of a 12-week functional exercise programme for institutionalised elderly people. Physiotherapy 1999 Jul;85(7):349-357. doi: 10.1016/S0031-9406(05)67187-8.
440. Ginandes CS, Rosenthal DI. Using hypnosis to accelerate the healing of bone fractures: a randomized controlled pilot study. Alternative Therapies in Health and Medicine 1999 Mar;5(2):67-75. PMID: 10069091.
441. Giovannelli M, Borriello G, Castri P, Prosperini L, Pozzilli C. Early physiotherapy after injection of Botulinum toxin increases the beneficial effects on spasticity in patients with multiple sclerosis. Clinical Rehabilitation 2007 Apr;21(4):331-337. doi: 10.1177/0269215507072772. PMID: 17613573.
442. Girodo M, Ekstrand KA, Metivier GJ. Deep diaphragmatic breathing: rehabilitation exercises for the asthmatic patient. Archives of Physical Medicine and Rehabilitation 1992 Aug;73(8):717-720. PMID: 1642520.
443. Glasser L. Effects of isokinetic training on the rate of movement during ambulation in hemiparetic patients. Physical Therapy 1986 May;66(5):673-676. PMID: 3703931.
444. Glavind K, Nohr SB, Walter S. Biofeedback and physiotherapy versus physiotherapy alone in the treatment of genuine stress urinary incontinence. International Urogynecology Journal and Pelvic Floor Dysfunction 1996 Nov;7(6):339-343. doi: 10.1007/BF01901111. PMID: 9203484.
445. Glazener C, Boachie C, Buckley B, Cochran C, Dorey G, Grant A, Hagen S, Kilonzo M, McDonald A, McPherson G, Moore K, N'Dow J, Norrie J, Ramsay C, Vale L. Conservative treatment for urinary incontinence in Men After Prostate Surgery (MAPS): two parallel randomised controlled trials. Health Technology Assessment (Winchester, England) 2011 Jun;15(24):1-296. doi: 10.3310/hta15240. PMID: 21640056.
446. Glazener CM, Herbison GP, Wilson PD, MacArthur C, Lang GD, Gee H, Grant AM. Conservative management of persistent postnatal urinary and faecal incontinence: randomised controlled trial. BMJ 2001 Sep 15;323(7313):593-596. doi: 10.1136/bmj.323.7313.593. PMID: 11557703.
447. Globas C, Becker C, Cerny J, Lam JM, Lindemann U, Forrester LW, Macko RF, Luft AR. Chronic stroke survivors benefit from high-intensity aerobic treadmill exercise: a randomized control trial. Neurorehabilitation and Neural Repair 2012 Jan;26(1):85-95. doi: 10.1177/1545968311418675. PMID: 21885867.
448. Glossop ES, Goldberg E, Smith DS, Williams HM. Patient compliance in back and neck pain. Physiotherapy 1982 Jul;68(7):225-226. PMID: 6215669.
449. Going S, Lohman T, Houtkooper L, Metcalfe L, Flint-Wagner H, Blew R, Stanford V, Cussler E, Martin J, Teixeira P, Harris M, Milliken L, Figueroa-Galvez A, Weber J. Effects of exercise on bone mineral density in calcium-replete postmenopausal women with and without hormone replacement therapy. Osteoporosis International 2003 Aug;14(8):637-643. doi: 10.1007/s00198-003-1436-x. PMID: 12844212.
450. Gold DT, Shipp KM, Pieper CF, Duncan PW, Martinez S, Lyles KW. Group treatment improves trunk strength and psychological status in older women with vertebral fractures: results of a randomized, clinical trial. Journal of the American Geriatrics Society 2004 Sep;52(9):1471-1478. doi: 10.1111/j.1532-5415.2004.52409.x. PMID: 15341548.
451. Goldberg AP, Geltman EM, Hagberg JM, Gavin JR 3rd, Delmez JA, Carney RM, Naumowicz A, Oldfield MH, Harter HR. Therapeutic benefits of exercise training for hemodialysis patients. Kidney International 1983 Dec;24(Suppl):S303-S309. PMID: 6588267.
452. Goldby LJ, Moore AP, Doust J, Trew ME. A randomized controlled trial investigating the efficiency of musculoskeletal physiotherapy on chronic low back disorder. Spine 2006 May 1;31(10):1083-1093. doi: 10.1097/01.brs.0000216464.37504.64. PMID: 16648741.
453. Goncalves RC, Nunes MPT, Cukier A, Stelmach R, Martins MA, Carvalho CRF. Efeito de um programa de condicionamento fisico aerobio nos aspectos psicossociais, na qualidade de vida, nos sintomas e no oxido nitrico exalado de portadores de asma persistente moderada ou grave (Effects of an aerobic physical training program on psychosocial characteristics, quality-of-life, symptoms and exhaled nitric oxide in individuals with moderate or severe persistent asthma) [Portuguese]. Revista Brasileira de Fisioterapia [Brazilian Journal of Physical Therapy] 2008 Mar-Apr;12(2):127-135. doi: 10.1590/S1413-35552008000200009.
454. Gonzales-Iglesias J, Fernandez-de-las-Penas C, Cleland JA, Alburquerque-Sendin F, Palomeque-del-Cerro L, Mendez-Sanchez R. Inclusion of thoracic spine thrust manipulation into an electrotherapy/thermal program for the management of patients with acute mechanical neck pain: a randomized clinical trial. Manual Therapy 2009 Jun;14(3):306-313. doi: 10.1016/j.math.2008.04.006. PMID: 18692428.
455. Gonzalez-Iglesias J, Fernandez-de-las-Penas C, Cleland J, Gutierrez-Vega MDR. Thoracic spine manipulation for the management of patients with neck pain: a randomized clinical trial. The Journal of Orthopaedic and Sports Physical Therapy 2009 Jan;39(1):20-27. doi: 10.2519/jospt.2009.2914. PMID: 19209478.
456. Goodall S, Howatson G. The effects of multiple cold water immersions on indices of muscle damage. Journal of Sports Science and Medicine 2008 Jun;7(2):235-241. PMID: 24149455.
457. Goode PS, Burgio KL, Johnson TM 2nd, Clay OJ, Roth DL, Markland AD, Burkhardt JH, Issa MM, Lloyd LK. Behavioral therapy with or without biofeedback and pelvic floor electrical stimulation for persistent postprostatectomy incontinence: a randomized controlled trial. JAMA 2011 Jan 12;305(2):151-159. doi: 10.1001/jama.2010.1972. PMID: 21224456.
458. Goode PS, Burgio KL, Locher JL, Roth DL, Umlauf MG, Richter HE, Varner RE, Lloyd LK. Effect of behavioral training with or without pelvic floor electrical stimulation on stress incontinence in women: a randomized controlled trial. JAMA 2003 Jul 16;290(3):345-352. doi: 10.1001/jama.290.3.345. PMID: 12865375.
459. Goossens ME, Rutten-van Molken MP, Kole-Snijders AM, Vlaeyen JW, van Breukelen G, Leidl R. Health economic assessment of behavioural rehabilitation in chronic low back pain: a randomised clinical trial. Health Economics 1998 Feb;7(1):39-51. doi: 10.1002/(SICI)1099-1050(199802)7:1<39::AID-HEC323>3.0.CO;2-S. PMID: 9541083.
460. Gorbea Chavez V, Velazquez Sanchez Mdel P, Kunhardt Rasch JR. Efecto de los ejercicios del piso pelvico durante el embarazo y el puerperio en la prevencion de la incontinencia urinaria de esfuerzo (Effect of pelvic floor exercise during pregnancy and puerperium on prevention of urinary stress incontinence) [Spanish]. Ginecologia y Obstetricia de Mexico 2004 Dec;72(12):628-636. PMID: 15813473.
461. Gordon NF, English CD, Contractor AS, Salmon RD, Leighton RF, Franklin BA, Haskell WL. Effectiveness of three models for comprehensive cardiovascular disease risk reduction. The American Journal of Cardiology 2002 Jun 1;89(11):1263-1268. doi: 10.1016/S0002-9149(02)02323-8. PMID: 12031725.
462. Gordon T, Amirjani N, Edwards DC, Chan KM. Brief postsurgical electrical stimulation accelerates axon regeneration and muscle reinnervation without affecting the functional measures in carpal tunnel syndrome patients. Experimental Neurology 2010 May;223(1):192-202. doi: 10.1016/j.expneurol.2009.09.020. PMID: 19800329.
463. Goren A, Yildiz N, Topuz O, Findikoglu G, Ardic F. Efficacy of exercise and ultrasound in patients with lumbar spinal stenosis: a prospective randomized controlled trial. Clinical Rehabilitation 2010 Jul;24(7):623-631. doi: 10.1177/0269215510367539. PMID: 20530650.
464. Gorodetskyi IG, Gorodnichenko AI, Tursin PS, Reshetnyak VK, Uskov ON. Use of noninvasive interactive neurostimulation to improve short-term recovery in patients with surgically repaired bimalleolar ankle fractures: a prospective, randomized clinical trial. The Journal of Foot and Ankle Surgery 2010 Sep-Oct;49(5):432-437. doi: 10.1053/j.jfas.2010.05.007. PMID: 20688546.
465. Gottlieb V, Lyngso AM, Nybo B, Frolich A, Backer V. Pulmonary rehabilitation for moderate COPD (GOLD 2) -- does it have an effect?. COPD 2011 Oct;8(5):380-386. doi: 10.3109/15412555.2011.610393. PMID: 21936683.
466. Gounden P. Progressive resistive loading on accessory expiratory muscles in tetraplegia. South African Journal of Physiotherapy 1990;46(4):4-16.
467. Grammatopoulou EP, Skordilis EK, Stavrou N, Myrianthefs P, Karteroliotis K, Baltopoulos G, Koutsouki D. The effect of physiotherapy-based breathing retraining on asthma control. The Journal of Asthma 2011 Aug;48(6):593-601. doi: 10.3109/02770903.2011.587583. PMID: 21668321.
468. Grandes G, Sanchez A, Montoya I, Sanchez-Pinilla RO, Torcal J. Two-year longitudinal analysis of a cluster randomized trial of physical activity promotion by general practitioners. PLoS ONE 2011 Mar;6(3):e18363. doi: 10.1371/journal.pone.0018363. PMID: 21479243.
469. Greist JH, Klein MH, Eischens RR, Faris J, Gurman AS, Morgan WP. Running as treatment for depression. Comprehensive Psychiatry 1979 Jan-Feb;20(1):41-54. doi: 10.1016/0010-440X(79)90058-0. PMID: 759100.
470. Griffith K, Wenzel J, Shang J, Thompson C, Stewart K, Mock V. Impact of a walking intervention on cardiorespiratory fitness, self-reported physical function, and pain in patients undergoing treatment for solid tumors. Cancer 2009 Oct 15;115(20):4874-4884. doi: 10.1002/cncr.24551. PMID: 19637345.
471. Grove KA, Londeree BR. Bone density in postmenopausal women: high impact versus low impact exercise. Medicine and Science in Sports and Exercise 1992 Nov;24(11):1190-1194. PMID: 1435170.
472. Gudavalli MR, Cambron JA, McGregor M, Jedlicka J, Keenum M, Ghanayem AJ, Patwardhan AG. A randomized clinical trial and subgroup analysis to compare flexion-distraction with active exercise for chronic low back pain. European Spine Journal 2006 Jul;15(7):1070-1082. doi: 10.1007/s00586-005-0021-8. PMID: 16341712.
473. Guell R, Casan P, Belda J, Sangenis M, Morante F, Guyatt GH, Sanchis J. Long-term effects of outpatient rehabilitation of COPD: a randomized trial. Chest 2000 Apr;117(4):976-983. doi: 10.1378/chest.117.4.976. PMID: 10767227.
474. Guell R, Resqueti V, Sangenis M, Morante F, Martorell B, Casan P, Guyatt GH. Impact of pulmonary rehabilitation on psychosocial morbidity in patients with severe COPD. Chest 2006 Apr;129(4):899-904. doi: 10.1378/chest.129.4.899. PMID: 16608936.
475. Guidon M, McGee H. One-year effect of a supervised exercise programme on functional capacity and quality of life in peripheral arterial disease. Disability and Rehabilitation 2013 Mar;35(5):397-404. doi: 10.3109/09638288.2012.694963. PMID: 22804715.
476. Gulick DT, Kimura IF, Sitler M, Paolone A, Kelly JD IV. Various treatment techniques on signs and symptoms of delayed onset muscle soreness. Journal of Athletic Training 1996 Jun;31(2):145-152. PMID: 16558388.
477. Guo Y, Sun Y-Z. (Clinical study on treatment of fibromyalgia syndrome with penetration needling at back) [Chinese - simplified characters]. Zhongguo Zhen Jiu [Chinese Acupuncture & Moxibustion] 2005 Feb;25(2):98-100. PMID: 16312890.
478. Gur A, Karakoc M, Cevik R, Nas K, Sarac AJ, Karakoc M. Efficacy of low power laser therapy and exercise on pain and functions in chronic low back pain. Lasers in Surgery and Medicine 2003 Mar;32(3):233-238. doi: 10.1002/lsm.10134. PMID: 12605431.
479. Gur H, Cakin N, Akova B, Okay E, Kucukoglu S. Concentric versus combined concentric-eccentric isokinetic training: effects on functional capacity and symptoms in patients with osteoarthrosis of the knee. Archives of Physical Medicine and Rehabilitation 2002 Mar;83(3):308-316. doi: 10.1053/apmr.2002.30620. PMID: 11887109.
480. Guvenol K, Tuzun C, Peker O, Goktay Y. A comparison of inverted spinal traction and conventional traction in the treatment of lumbar disc herniations. Physiotherapy Theory and Practice 2000;16(3):151-160. doi: 10.1080/095939800750036079.
481. Haakstad LAH, Bo K. Effect of regular exercise on prevention of excessive weight gain in pregnancy: a randomised controlled trial. The European Journal of Contraception & Reproductive Health Care 2011 Apr;16(2):116-125. doi: 10.3109/13625187.2011.560307. PMID: 21417561.
482. Haas CT, Turbanski S, Kessler K, Schmidtbleicher D. The effects of random whole-body-vibration on motor symptoms in Parkinson's disease. Neurorehabilitation 2006;21(1):29-36. PMID: 16720935.
483. Haas M, Groupp E, Aickin M, Fairweather A, Ganger B, Attwood M, Cummins C, Baffes L. Dose response for chiropractic care of chronic cervicogenic headache and associated neck pain: a randomized pilot study. Journal of Manipulative and Physiological Therapeutics 2004 Nov-Dec;27(9):547-553. doi: 10.1016/j.jmpt.2004.10.007. PMID: 15614241.
484. Haas M, Spegman A, Peterson D, Aickin M, Vavrek D. Dose-response and efficacy of spinal manipulation for chronic cervicogenic headache: a pilot randomized controlled trial. The Spine Journal 2010 Feb;10(2):117-128. doi: 10.1016/j.spinee.2009.09.002. PMID: 19837005.
485. Hacker ED, Larson J, Kujath A, Peace D, Rondelli D, Gaston L. Strength training following hematopoietic stem cell transplantation. Cancer Nursing 2011 May-Jun;34(3):238-249. doi: 10.1097/NCC.0b013e3181fb3686. PMID: 21116175.
486. Haerens L, Deforche B, Maes L, Cardon G, Stevens V, de Bourdeaudhuij I. Evaluation of a 2-year physical activity and healthy eating intervention in middle school children. Health Education Research 2006 Dec;21(6):911-921. doi: 10.1093/her/cyl115. PMID: 17032704.
487. Haerens L, Maes L, Vereecken C, de Henaux S, More L, de Bourdeaudhuij ID. Effectiveness of a computer tailored physical activity intervention in adolescents compared to a generic advice. Patient Education and Counseling 2009 Oct;77(1):38-41. doi: 10.1016/j.pec.2009.03.020. PMID: 19406608.
488. Haffner J, Roos J, Goldstein N, Parzer P, Resch F. Zur wirksamkeit korperorientierter therapieverfahren bei der behandlung hyperaktiver storungen: ergebnisse einer kontrollierten pilotstudie (The effectiveness of body-oriented methods of therapy in the treatment of attention-deficit hyperactivity disorder (ADHD): results of a controlled pilot study) [German]. Zeitschrift fur Kinder und Jugendpsychiatrie und Psychotherapie 2006 Jan;34(1):37-47. PMID: 16485612.
489. Hagen EM, Eriksen HR, Ursin H. Does early intervention with a light mobilization program reduce long-term sick leave for low back pain?. Spine 2000 Aug 1;25(15):1973-1976. PMID: 10908942.
490. Hagen EM, Grasdal A, Eriksen HR. Does early intervention with a light mobilization program reduce long-term sick leave for low back pain: a 3-year follow-up study. Spine 2003 Oct 15;28(20):2309-2316. PMID: 14560075.
491. Hagen S, Stark D, Glazener C, Sinclair L, Ramsay I. A randomized controlled trial of pelvic floor muscle training for stages I and II pelvic organ prolapse. International Urogynecology Journal and Pelvic Floor Dysfunction 2009 Jan;20(1):45-51. doi: 10.1007/s00192-008-0726-4. PMID: 18806910.
492. Hakkinen A, Hakkinen K, Hannonen P, Alen M. Strength training induced adaptations in neuromuscular function of premenopausal women with fibromyalgia: comparison with healthy women. Annals of the Rheumatic Diseases 2001 Jan;60(1):21-26. doi: 10.1136/ard.60.1.21. PMID: 11114277.
493. Hakkinen A, Ylinen J, Kautiainen H, Tarvainen U, Kiviranta I. Effects of home strength training and stretching versus stretching alone after lumbar disk surgery: a randomized study with a 1-year follow-up. Archives of Physical Medicine and Rehabilitation 2005 May;86(5):865-870. doi: 10.1016/j.apmr.2004.11.012. PMID: 15895329.
494. Hale SA, Hertel J, Olmsted-Kramer LC. The effect of a 4-week comprehensive rehabilitation program on postural control and lower extremity function in individuals with chronic ankle instability. The Journal of Orthopaedic and Sports Physical Therapy 2007 Jun;37(6):303-311. doi: 10.2519/jospt.2007.2322. PMID: 17612356.
495. Hall JC, Tarala R, Harris J, Tapper J, Christiansen K. Incentive spirometry versus routine chest physiotherapy for prevention of pulmonary complications after abdominal surgery. Lancet 1991 Apr 20;337(8747):953-956. doi: 10.1016/0140-6736(91)91580-N. PMID: 1678039.
496. Hall JC, Tarala RA, Tapper J, Hall JL. Prevention of respiratory complications after abdominal surgery: a randomised clinical trial. BMJ 1996 Jan 20;312(7024):148-152. PMID: 8563533.
497. Halson SL, Quod MJ, Martin DT, Gardner AS, Ebert TR, Laursen PB. Physiological responses to cold water immersion following cycling in the heat. International Journal of Sports Physiology and Performance 2008 Sep;3(3):331-346. PMID: 19211945.
498. Handolin L, Kiljunen V, Arnala I, Kiuru MJ, Pajarinen J, Partio EK, Rokkanen P. Effect of ultrasound therapy on bone healing of lateral malleolar fractures of the ankle joint fixed with bioabsorbable screws. Journal of Orthopaedic Science 2005 Jul;10(4):391-395. doi: 10.1007/s00776-005-0901-0. PMID: 16075172.
499. Handolin L, Kiljunen V, Arnala I, Kiuru MJ, Pajarinen J, Partio EK, Rokkanen P. No long-term effects of ultrasound therapy on bioabsorbable screw-fixed lateral malleolar fracture. Scandinavian Journal of Surgery 2005;94(3):239-242. PMID: 16259175.
500. Handolin L, Kiljunen V, Arnala I, Pajarinen J, Partio EK, Rokkanen P. The effect of low intensity ultrasound and bioabsorbable self-reinforced poly-L-lactide screw fixation on bone in lateral malleolar fractures. Archives of Orthopaedic and Trauma Surgery 2005 Jun;125(5):317-321. doi: 10.1007/s00402-005-0801-y. PMID: 15821899.
501. Hansson EE, Jonsson-Lundgren M, Ronnheden AM, Sorensson E, Bjarnung A, Dahlberg LE. Effect of an education programme for patients with osteoarthritis in primary care -- a randomized controlled trial. BMC Musculoskeletal Disorders 2010 Oct 25;11(244):Epub. doi: 10.1186/1471-2474-11-244. PMID: 20969809.
502. Hanten WP, Barrett M, Gillespie-Plesko M, Jump KA, Olson SL. Effects of active head retraction with retraction/extension and occipital release on the pressure pain threshold of cervical and scapular trigger points. Physiotherapy Theory and Practice 1997;13(4):285-291.
503. Hanten WP, Olson SL, Butts NL, Nowicki AL. Effectiveness of a home program of ischemic pressure followed by sustained stretch for treatment of myofascial trigger points. Physical Therapy 2000 Oct;80(10):997-1003. PMID: 11002435.
504. Harari D, Norton C, Lockwood L, Swift C. Treatment of constipation and fecal incontinence in stroke patients: randomised controlled trial. Stroke 2004 Nov;35(11):2549-2555. doi: 10.1161/01.STR.0000144684.46826.62. PMID: 15486330.
505. Harland J, White M, Drinkwater C, Chinn D, Farr L, Howel D. The Newcastle exercise project: a randomised controlled trial of methods to promote physical activity in primary care. BMJ 1999 Sep 25;319(7213):828-832. PMID: 10496829.
506. Harms M, Engstrom B. Continuous passive motion as an adjunct to treatment in the physiotherapy management of the total knee arthroplasty patient. Physiotherapy 1991 Apr;77(4):301-307. doi: 10.1016/S0031-9406(10)61768-3.
507. Harris RE, Tian X, Williams DA, Tian TX, Cupps TR, Petzke F, Groner KH, Biswas P, Gracely RH, Clauw DJ. Treatment of fibromyalgia with formula acupuncture: investigation of needle placement, needle stimulation, and treatment frequency. Journal of Alternative & Complementary Medicine 2005 Aug;11(4):663-671. doi: 10.1089/acm.2005.11.663. PMID: 16131290.
508. Harris RE, Zubieta J-K, Scott DJ, Napadow V, Gracely RH, Clauw DJ. Traditional Chinese acupuncture and placebo (sham) acupuncture are differentiated by their effects on mu-opioid receptors (MORs). NeuroImage 2009 Sep;47(3):1077-1088. doi: 10.1016/j.neuroimage.2009.05.083. PMID: 19501658.
509. Harrison CL, Lombard CB, Strauss BJ, Teede HJ. Optimizing healthy gestational weight gain in women at high risk of gestational diabetes: a randomized controlled trial. Obesity 2013 May;21(5):904-909. doi: 10.1002/oby.20163. PMID: 23784892.
510. Harte AA, Baxter GD, Gracey JH. The effectiveness of motorised lumbar traction in the management of LBP with lumbo sacral nerve root involvement: a feasibility study. BMC Musculoskeletal Disorders 2007 Nov 29;8(118):Epub. doi: 10.1186/1471-2474-8-118. PMID: 18047650.
511. Harter HR, Goldberg AP. Endurance exercise training. An effective therapeutic modality for hemodialysis patients. The Medical Clinics of North America 1985 Jan;69(1):159-175. PMID: 3883073.
512. Hartman A, te Winkel ML, van Beek RD, de Muinck Keizer-Schrama SMPF, Kemper HCG, Hop WCJ, van den Heuvel-Eibrink MM, Pieters R. A randomized trial investigating an exercise program to prevent reduction of bone mineral density and impairment of motor performance during treatment for childhood acute lymphoblastic leukemia. Pediatric Blood & Cancer 2009 Jul;53(1):64-71. doi: 10.1002/pbc.21942. PMID: 19283791.
513. Harvey-Berino J, Pintauro S, Buzzell P, di Giulio M, Gold BC, Moldovan C, Ramirez E. Does using the internet facilitate the maintenance of weight loss?. International Journal of Obesity and Related Metabolic Disorders 2002 Sep;26(9):1254-1260. doi: 10.1038/sj.ijo.0802051. PMID: 12187404.
514. Harvey-Berino J, West D, Krukowski R, Prewitt E, van Biervliet A, Ashikaga T, Skelly J. Internet delivered behavioral obesity treatment. Preventive Medicine 2010 Aug;51(2):123-128. doi: 10.1016/j.ypmed.2010.04.018. PMID: 20478333.
515. Hatori M, Hasegawa A, Adachi H, Shinozaki A, Hayashi R, Okano H, Mizunuma H, Murata K. The effects of walking at the anaerobic threshold level on vertebral bone loss in postmenopausal women. Calcified Tissue International 1993 Jun;52(6):411-414. doi: 10.1007/BF00571327. PMID: 8369985.
516. Hawk C, Long CR, Rowell RM, Gudavalli MR, Jedlicka J. A randomized trial investigating a chiropractic manual placebo: a novel design using standardized forces in the delivery of active and control treatments. Journal of Alternative & Complementary Medicine 2005 Feb;11(1):109-117. doi: 10.1089/acm.2005.11.109. PMID: 15750369.
517. Hawkins M, Hosker M, Marcus BH, Rosal MC, Braun B, Stanek EJ III, Markenson G, Chasan-Taber L. A pregnancy lifestyle intervention to prevent gestational diabetes risk factors in overweight Hispanic women: a feasibility randomized controlled trial. Diabetic Medicine 2015 Jan;32(1):108-115. doi: 10.1111/dme.12601. PMID: 25306925.
518. Hay EM, Foster NE, Thomas E, Peat G, Phelan M, Yates HE, Blenkinsopp A, Sim J. Effectiveness of community physiotherapy and enhanced pharmacy review for knee pain in people aged over 55 presenting to primary care: pragmatic randomised trial. BMJ 2006 Nov 11;333(7576):995-1003. doi: 10.1136/bmj.38977.590752.0B. PMID: 17056608.
519. Hayes SC, Reul-Hirche H, Turner J. Exercise and secondary lymphedema: safety, potential benefits, and research issues. Medicine and Science in Sports and Exercise 2009 Mar;41(3):483-489. doi: 10.1249/MSS.0b013e31818b98fb. PMID: 19204604.
520. Hazard RG, Reid S, Haugh LD, McFarlane G. A controlled trial of an educational pamphlet to prevent disability after occupational low back injury. Spine 2000 Jun 1;25(11):1419-1423. PMID: 10828925.
521. Headley JA, Ownby KK, John LD. The effect of seated exercise on fatigue and quality of life in women with advanced breast cancer. Oncology Nursing Forum 2004 Sep;31(5):977-983. doi: 10.1188/04.ONF.977-983. PMID: 15378098.
522. Healy WL, Seidman J, Pfeifer BA, Brown DG. Cold compressive dressing after total knee arthroplasty. Clinical Orthopaedics and Related Research 1994 Feb;(299):143-146. PMID: 7907012.
523. Hebestreit H, Kieser S, Junge S, Ballmann M, Hebestreit A, Schindler C, Schenk T, Posselt HG, Kriemler S. Long-term effects of a partially supervised conditioning programme in cystic fibrosis. The European Respiratory Journal 2010 Mar;35(3):578-583. doi: 10.1183/09031936.00062409. PMID: 19643946.
524. Heckman JD, Ryaby JP, McCabe J, Frey JJ, Kilcoyne RF. Acceleration of tibial fracture-healing by non-invasive, low-intensity pulsed ultrasound. Journal of Bone and Joint Surgery -- American Volume 1994 Jan;76(1):26-34. PMID: 8288661.
525. Hedstrom M, Ahl T, Dalen N. Early postoperative ankle exercise. A study of postoperative lateral malleolar fractures. Clinical Orthopaedics and Related Research 1994 Mar;(300):193-196. PMID: 8131334.
526. Heebner ML, Roddey TS. The effects of neural mobilization in addition to standard care in persons with carpal tunnel syndrome from a community hospital. Journal of Hand Therapy 2008 Jul-Sep;21(3):229-241. doi: 10.1197/j.jht.2007.12.001. PMID: 18652967.
527. Heim ME, Malsburg ME, Niklas A. Randomized controlled trial of a structured training program in breast cancer patients with tumor-related chronic fatigue. Onkologie 2007 Sep;30(8-9):429-434. doi: 10.1159/000104097. PMID: 17848814.
528. Heinen M, Borm G, van der Vleuten C, Evers A, Oostendorp R, van Achterberg T. The Lively Legs self-management programme increased physical activity and reduced wound days in leg ulcer patients: results from a randomized controlled trial. International Journal of Nursing Studies 2012 Feb;49(2):151-161. doi: 10.1016/j.ijnurstu.2011.09.005. PMID: 21959100.
529. Heldmann B, Kerkhoff G, Struppler A, Havel P, Jahn T. Repetitive peripheral magnetic stimulation alleviates tactile extinction. Neuroreport 2000;11(14):3193-3198. PMID: 11043547.
530. Hemat-Far A, Shahsavari A, Mousavi SR. Effects of selected aerobic exercises on the depression and concentrations of plasma serotonin in the depressed female students aged 18 to 25. The Journal of Applied Research 2012;12(1):47-52.
531. Hemmila HM, Keinanen-Kiukaanniemi SM, Levoska S, Puska P. Long-term effectiveness of bone-setting, light exercise therapy, and physiotherapy for prolonged back pain: a randomized controlled trial. Journal of Manipulative and Physiological Therapeutics 2002 Feb;25(2):99-104. doi: 10.1067/mmt.2002.122329. PMID: 11896377.
532. Hendriks O, Horgan A. Ultra-reiz current as an adjunct to standard physiotherapy treatment of the acute whiplash patient. Physiotherapy Ireland 1996;17(1):3-7.
533. Hermiz O, Comino E, Marks G, Daffurn K, Wilson S, Harris M. Randomised controlled trial of home based care of patients with chronic obstructive pulmonary disease. BMJ 2002 Oct 26;325(7370):938. doi: 10.1136/bmj.325.7370.938. PMID: 12399344.
534. Herrero F, San Juan AF, Fleck SJ, Balmer J, Perez M, Canete S, Earnest CP, Foster C, Lucia A. Combined aerobic and resistance training in breast cancer survivors: a randomized, controlled pilot trial. International Journal of Sports Medicine 2006 Jul;27(7):573-580. doi: 10.1055/s-2005-865848. PMID: 16802254.
535. Hervik J, Mjaland O. Acupuncture for the treatment of hot flashes in breast cancer patients, a randomized, controlled trial. Breast Cancer Research and Treatment 2009 Jul;116(2):311-316. doi: 10.1007/s10549-008-0210-3. PMID: 18839306.
536. Heuts PH, de Bie R, Drietelaar M, Aretz K, Hopman-Rock M, Bastiaenen CH, Metsemakers JF, van Weel C, van Schayck O. Self-management in osteoarthritis of hip or knee: a randomized clinical trial in a primary healthcare setting. The Journal of Rheumatology 2005 Mar;32(3):543-549. PMID: 15742451.
537. Heymans MW, de Vet HC, Bongers PM, Knol DL, Koes BW, van Mechelen W. The effectiveness of high-intensity versus low-intensity back schools in an occupational setting: a pragmatic randomized controlled trial. Spine 2006 May 1;31(10):1075-1082. doi: 10.1097/01.brs.0000216443.46783.4d. PMID: 16648740.
538. Hiatt WR, Regensteiner JG, Hargarten ME, Wolfel EE, Brass EP. Benefit of exercise conditioning for patients with peripheral arterial disease. Circulation 1990 Feb;81(2):602-609. doi: 10.1161/01.CIR.81.2.602. PMID: 2404633.
539. Hiatt WR, Wolfel EE, Meier RH, Regensteiner JG. Superiority of treadmill walking exercise versus strength training for patients with peripheral arterial disease. Implications for the mechanism of the training response. Circulation 1994 Oct;90(4):1866-1874. doi: 10.1161/01.CIR.90.4.1866. PMID: 7923674.
540. Hides JA, Jull GA, Richardson CA. Long-term effects of specific stabilizing exercises for first-episode low back pain. Spine 2001 Jun 1;26(11):E243-E248. PMID: 11389408.
541. High DM, Howley ET, Franks BD. The effects of static stretching and warm-up on prevention of delayed-onset muscle soreness. Research Quarterly for Exercise and Sport 1989 Dec;60(4):357-361. doi: 10.1080/02701367.1989.10607463. PMID: 2489863.
542. Hill PD. Effects of heat and cold on the perineum after episiotomy/laceration. Journal of Obstetric, Gynecologic, and Neonatal Nursing 1989 Mar-Apr;18(2):124-129. doi: 10.1111/j.1552-6909.1989.tb00475.x. PMID: 2709180.
543. Hillsdon M, Thorogood M, White I, Foster C. Advising people to take more exercise is ineffective: a randomized controlled trial of physical activity promotion in primary care. International Journal of Epidemiology 2002;31(4):808-815. doi: 10.1093/ije/31.4.808. PMID: 12177026.
544. Hobbs SD, Marshall T, Fegan C, Adam DJ, Bradbury AW. The effect of supervised exercise and cilostazol on coagulation and fibrinolysis in intermittent claudication: a randomized controlled trial. Journal of Vascular Surgery 2007 Jan;45(1):65-70. doi: 10.1016/j.jvs.2006.08.084. PMID: 17210383.
545. Hochberg J. A randomized prospective study to assess the efficacy of two cold-therapy treatments following carpal tunnel release. Journal of Hand Therapy 2001;14(3):208-215. PMID: 11511016.
546. Hodges LD, Sandercock GRH, Das SK, Brodie DA. Randomized controlled trial of supervised exercise to evaluate changes in cardiac function in patients with peripheral atherosclerotic disease. Clinical Physiology and Functional Imaging 2008 Jan;28(1):32-37. doi: 10.1111/j.1475-097X.2007.00770.x. PMID: 18005078.
547. Hofbauer J, Preisinger F, Nurnberger N. (The value of physical therapy in genuine female stress incontinence) [German]. Zeitschrift fur Urologie Und Nephrologie 1990 May;83(5):249-254. PMID: 2203214.
548. Hoffman JM, Bell KR, Powell JM, Behr J, Dunn EC, Dikmen S, Bombardier CH. A randomized controlled trial of exercise to improve mood after traumatic brain injury. PM&R 2010 Oct;2(10):911-919. doi: 10.1016/j.pmrj.2010.06.008. PMID: 20970760.
549. Hoffmann W, Liedke S, Dombo O, Otto U. Die elektrostimulation in der therapie der postoperativen harninkontinenz. Therapeutischer nutzen unter berucksichtigung der lebensqualitat (Electrical stimulation to treat postoperative incontinence. Therapeutic benefit in regard to quality of life) [German]. Der Urologe Ausg A 2005 Jan;44(1):33-40. doi: 10.1007/s00120-004-0732-1. PMID: 15580472.
550. Hofstee DJ, Gijtenbeek JMM, Hoogland PH, van Houwelingen HC, Kloet A, Lotters F, Tans JTJ. Westeinde Sciatica Trial: randomized controlled study of bed rest and physiotherapy for acute sciatica. Journal of Neurosurgery 2002 Jan;96(1 Suppl S):45-49. doi: 10.3171/spi.2002.96.1.0045. PMID: 11797655.
551. Hoiness P, Glott T, Ingjer F. High-intensity training with a bi-directional bicycle pedal improves performance in mechanically unstable ankles -- a prospective randomized study of 19 subjects. Scandinavian Journal of Medicine & Science in Sports 2003 Aug;13(4):266-271. doi: 10.1034/j.1600-0838.2003.10140.x. PMID: 12859610.
552. Holloway EA, West RJ. Integrated breathing and relaxation training (the Papworth method) for adults with asthma in primary care: a randomised controlled trial. Thorax 2007 Dec;62(12):1039-1042. doi: 10.1136/thx.2006.076430. PMID: 17573445.
553. Holmich P, Uhrskou P, Ulnits L, Kanstrup IL, Nielsen MB, Bjerg AM, Krogsgaard K. Effectiveness of active physical training as treatment for long-standing adductor-related groin pain in athletes: randomised trial. Lancet 1999 Feb 6;353(9151):439-443. doi: 10.1016/S0140-6736(98)03340-6. PMID: 9989713.
554. Hondras MA, Long CR, Cao Y, Rowell RM, Meeker WC. A randomized controlled trial comparing 2 types of spinal manipulation and minimal conservative medical care for adults 55 years and older with subacute or chronic low back pain. Journal of Manipulative and Physiological Therapeutics 2009 Jun;32(5):330-343. doi: 10.1016/j.jmpt.2009.04.012. PMID: 19539115.
555. Hong CZ, Lin JC, Bender LF, Schaeffer JN, Meltzer RJ, Causin P. Magnetic necklace: its therapeutic effectiveness on neck and shoulder pain. Archives of Physical Medicine and Rehabilitation 1982 Oct;63(10):462-466. PMID: 7138256.
556. Honigmann P, Goldhahn S, Rosenkranz J, Audige L, Geissmann D, Babst R. After treatment of malleolar fractures following ORIF -- functional compared to protected functional in a vacuum-stabilized orthesis: a randomized controlled trial. Archives of Orthopaedic and Trauma Surgery 2007 Apr;127(3):195-203. doi: 10.1007/s00402-006-0255-x. PMID: 17195934.
557. Hopman-Rock M, Westhoff MH. The effects of a health educational and exercise program for older adults with osteoarthritis for the hip or knee. The Journal of Rheumatology 2000 Aug;27(8):1947-1954. PMID: 10955337.
558. Horneij E, Hemborg B, Jensen I, Ekdahl C. No significant differences between intervention programmes on neck, shoulder and low back pain: a prospective randomized study among home-care personnel. Journal of Rehabilitation Medicine 2001 Jul;33(4):170-176. doi: 10.1080/16501970116887. PMID: 11506215.
559. Horng Y-S, Hsieh S-F, Tu Y-K, Lin M-C, Horng Y-S, Wang J-D. The comparative effectiveness of tendon and nerve gliding exercises in patients with carpal tunnel syndrome: a randomized trial. American Journal of Physical Medicine & Rehabilitation 2011 Jun;90(6):435-442. doi: 10.1097/PHM.0b013e318214eaaf. PMID: 21430512.
560. Hou C-P, Chen T-Y, Chang C-C, Lin Y-H, Chang P-L, Chen C-L, Hsu Y-C, Tsui K-H. Use of the SF-36 quality of life scale to assess the effect of pelvic floor muscle exercise on aging males who received transurethral prostate surgery. Clinical Interventions in Aging 2013;8:667-673. doi: 10.2147/CIA.S44321. PMID: 23766642.
561. Hou C-R, Tsai L-C, Cheng K-F, Chung K-C, Hong C-Z. Immediate effects of various physical therapeutic modalities on cervical myofascial pain and trigger-point sensitivity. Archives of Physical Medicine and Rehabilitation 2002 Oct;83(10):1406-1414. doi: 10.1053/apmr.2002.34834. PMID: 12370877.
562. Howe DH, Newcombe RG, Wade MT. Manipulation of the cervical spine -- a pilot study. Journal of the Royal College of General Practitioners 1983 Sep;33(254):574-579. PMID: 6355460.
563. Hruda KV, Hicks AL, McCartney N. Training for muscle power in older adults: effects on functional abilities. Revue Canadienne de Physiologie Appliquee [Canadian Journal of Applied Physiology] 2003 Apr;28(2):178-189. PMID: 12825328.
564. Hsieh CY, Adams AH, Tobis J, Hong CZ, Danielson C, Platt K, Hoehler F, Reinsch S, Rubel A. Effectiveness of four conservative treatments for subacute low back pain: a randomized clinical trial. Spine 2002 Jun 1;27(11):1142-1148. PMID: 12045509.
565. Hsieh CY, Phillips RB, Adams AH, Pope MH. Functional outcomes of low back pain: comparison of four treatment groups in a randomized controlled trial. Journal of Manipulative and Physiological Therapeutics 1992 Jan;15(1):4-9. PMID: 1531488.
566. Hsueh TC, Cheng PT, Kuan TS, Hong CZ. The immediate effectiveness of electrical nerve stimulation and electrical muscle stimulation on myofascial trigger points. American Journal of Physical Medicine & Rehabilitation 1997 Nov-Dec;76(6):471-476. PMID: 9431265.
567. Huang D, Peng Y, Su P, Ye W, Liang A. The effect of continuous passive motion after total knee arthroplasty on joint function. Zhongguo Linchuang Kangfu [Chinese Journal of Clinical Rehabilitation] 2003 May 25;7(11):1661-1662.
568. Huang MH, Lin YS, Yang RC, Lee CL. A comparison of various therapeutic exercises on the functional status of patients with knee osteoarthritis. Seminars in Arthritis and Rheumatism 2003 Jun;32(6):398-406. doi: 10.1053/sarh.2003.50021. PMID: 12833248.
569. Huang MH, Yang RC, Lee CL, Chen TW, Wang MC. Preliminary results of integrated therapy for patients with knee osteoarthritis. Arthritis Care & Research 2005 Dec 15;53(6):812-820. doi: 10.1002/art.21590. PMID: 16342083.
570. Huang T-T, Yeh C-Y, Tsai Y-C. A diet and physical activity intervention for preventing weight retention among Taiwanese childbearing women: a randomised controlled trial. Midwifery 2011 Apr;27(2):257-264. doi: 10.1016/j.midw.2009.06.009. PMID: 19775782.
571. Huemer GM, Koller M, Pachinger T, Dunst KM, Schwarz B, Hintringer T. Postoperative splinting after open carpal tunnel release does not improve functional and neurological outcome. Muscle & Nerve 2007 Oct;36(4):528-531. doi: 10.1002/mus.20839. PMID: 17617802.
572. Hughes SL, Seymour RB, Campbell R, Pollak N, Huber G, Sharma L. Impact of the fit and strong intervention on older adults with osteoarthritis. The Gerontologist 2004 Apr;44(2):217-228. doi: 10.1093/geront/44.2.217. PMID: 15075418.
573. Hui A, Back L, Ludwig S, Gardiner P, Sevenhuysen G, Dean H, Sellers E, McGavock J, Morris M, Bruce S, Murray R, Shen GX. Lifestyle intervention on diet and exercise reduced excessive gestational weight gain in pregnant women under a randomised controlled trial. BJOG 2012 Jan;119(1):70-77. doi: 10.1111/j.1471-0528.2011.03184.x. PMID: 22017967.
574. Hui AL, Back L, Ludwig S, Gardiner P, Sevenhuysen G, Dean HJ, Sellers E, McGavock J, Morris M, Jiang D, Shen GX. Effects of lifestyle intervention on dietary intake, physical activity level, and gestational weight gain in pregnant women with different pre-pregnancy body mass index in a randomized control trial. BMC Pregnancy and Childbirth 2014 Sep 24;14(331):Epub. doi: 10.1186/1471-2393-14-331. PMID: 25248797.
575. Hui AL, Ludwig S, Gardiner P, Sevenhuysen G, Murray R, Morris M, Shen GX. Community-based exercise and dietary intervention during pregnancy: a pilot study. Canadian Journal of Diabetes 2006 Jun;30(2):169-175. doi: 10.1016/S1499-2671(06)02010-7.
576. Huibers MJ, Beurskens AJ, van Schayck CP, Baselmans E, Metsemakers JF, Knottnerus JA, Bleijenberg G. Efficacy of cognitive-behavioural therapy by general practitioners for unexplained fatigue among employees: randomised controlled trial. British Journal of Psychiatry 2004 Mar;184(3):240-246. doi: 10.1192/bjp.184.3.240. PMID: 14990522.
577. Hulzebos EH, Helders PJ, Favie NJ, de Bie RA, Brutel de la Riviere A, van Meeteren NL. Preoperative intensive inspiratory muscle training to prevent postoperative pulmonary complications in high-risk patients undergoing CABG surgery: a randomized clinical trial. JAMA 2006 Oct 18;296(15):1851-1857. doi: 10.1001/jama.296.15.1851. PMID: 17047215.
578. Hulzebos EH, van Meeteren NL, van den Buijs BJ, de Bie RA, Brutel de la Riviere A, Helders PJ. Feasibility of preoperative inspiratory muscle training in patients undergoing coronary artery bypass surgery with a high risk of postoperative pulmonary complications: a randomized controlled pilot study. Clinical Rehabilitation 2006 Nov;20(11):949-959. doi: 10.1177/0269215506070691. PMID: 17065538.
579. Hung H-C, Hsiao S-M, Chih S-Y, Lin H-H, Tsauo J-Y. An alternative intervention for urinary incontinence: retraining diaphragmatic, deep abdominal and pelvic floor muscle coordinated function. Manual Therapy 2010 Jun;15(3):273-279. doi: 10.1016/j.math.2010.01.008. PMID: 20185357.
580. Hunter CM, Peterson AL, Alvarez LM, Poston WC, Brundige AR, Haddock CK, van Brunt DL, Foreyt JP. Weight management using the internet: a randomized controlled trial. American Journal of Preventive Medicine 2008 Feb;34(2):119-126. doi: 10.1016/j.amepre.2007.09.026. PMID: 18201641.
581. Hurley DA, Minder PM, McDonough SM, Walsh DM, Moore AP, Baxter DG. Interferential therapy electrode placement technique in acute low back pain: a preliminary investigation. Archives of Physical Medicine and Rehabilitation 2001 Apr;82(4):485-493. doi: 10.1053/apmr.2001.21934. PMID: 11295009.
582. Hurley MV, Walsh NE, Mitchell HL, Pimm TJ, Patel A, Williamson E, Jones RH, Dieppe PA, Reeves BC. Clinical effectiveness of a rehabilitation program integrating exercise, self-management, and active coping strategies for chronic knee pain: a cluster randomized trial. Arthritis Care & Research 2007 Oct 15;57(7):1211-1219. doi: 10.1002/art.22995. PMID: 17907147.
583. Hurri H. The Swedish back school in chronic low back pain. Part I. benefits. Scandinavian Journal of Rehabilitation Medicine 1989;21(1):33-40. PMID: 2523558.
584. Hurwitz EL, Morgenstern H, Harber P, Kominski GF, Belin TR, Yu F, Adams AH. A randomized trial of medical care with and without physical therapy and chiropractic care with and without physical modalities for patients with low back pain: 6-month follow-up outcomes from the UCLA low back pain study. Spine 2002 Oct 15;27(20):2193-2204. PMID: 12394892.
585. Hurwitz EL, Morgenstern H, Harber P, Kominski GF, Yu F, Adams AH. A randomized trial of chiropractic manipulation and mobilization for patients with neck pain: clinical outcomes from the UCLA neck-pain study. American Journal of Public Health 2002 Oct;92(10):1634-1641. PMID: 12356613.
586. Hwang JH, Chang HJ, Shim YH, Park WH, Park W, Huh SJ, Yang J-H. Effects of supervised exercise therapy in patients receiving radiotherapy for breast cancer. Yonsei Medical Journal 2008 Jun 30;49(3):443-450. doi: 10.3349/ymj.2008.49.3.443. PMID: 18581595.
587. Inaba M, Edberg E, Montgomery J, Gillis MK. Effectiveness of functional training, active exercise, and resistive exercise for patients with hemiplegia. Physical Therapy 1973 Jan;53(1):28-35. PMID: 4682697.
588. Indahl A, Velund L, Reikeraas O. Good prognosis for low back pain when left untampered. A randomized clinical trial. Spine 1995 Feb 15;20(4):473-477. PMID: 7747232.
589. Ingram J, Dawson B, Goodman C, Wallman K, Beilby J. Effect of water immersion methods on post-exercise recovery from simulated team sport exercise. Journal of Science and Medicine in Sport 2009 May;12(3):417-421. doi: 10.1016/j.jsams.2007.12.011. PMID: 18547863.
590. Irnich D, Behrens N, Molzen H, Konig A, Gleditsch J, Krauss M, Natalis M, Senn E, Beyer A, Schops P. Randomised trial of acupuncture compared with conventional massage and "sham" laser acupuncture for treatment of chronic neck pain. BMJ 2001 Jun 30;322(7302):1574-1578. doi: 10.1136/bmj.322.7302.1574. PMID: 11431299.
591. Ishiko O, Ushiroyama T, Saji F, Mitsuhashi Y, Tamura T, Yamamoto K, Kawamura Y, Ogita S. Beta2-adrenergic agonists and pelvic floor exercises for female stress incontinence. International Journal of Gynaecology and Obstetrics 2000 Oct;71(1):39-44. doi: 10.1016/S0020-7292(00)00254-X. PMID: 11044540.
592. Itoh K, Kitakoji H. Effects of acupuncture to treat fibromyalgia: a preliminary randomised controlled trial. Chinese Medicine 2010 Mar 23;5(11):Epub. doi: 10.1186/1749-8546-5-11. PMID: 20331844.
593. Ivey FM, Hafer-Macko CE, Ryan AS, Macko RF. Impaired leg vasodilatory function after stroke: adaptations with treadmill exercise training. Stroke 2010 Dec;41(12):2913-2917. doi: 10.1161/STROKEAHA.110.599977. PMID: 20966405.
594. Ivey FM, Ryan AS, Hafer-Macko CE, Macko RF. Improved cerebral vasomotor reactivity after exercise training in hemiparetic stroke survivors. Stroke 2011 Jul;42(7):1994-2000. doi: 10.1161/STROKEAHA.110.607879. PMID: 21636819.
595. Ivey M, Johnston RV, Uchida T. Cryotherapy for postoperative pain relief following knee arthroplasty. The Journal of Arthroplasty 1994 Jun;9(3):285-290. PMID: 8077977.
596. Iwamoto J, Takeda T, Ichimura S. Effect of exercise training and detraining on bone mineral density in postmenopausal women with osteoporosis. Journal of Orthopaedic Science 2001;6(2):128-132. doi: 10.1007/s007760100059. PMID: 11484097.
597. Iwamoto J, Takeda T, Sato Y, Uzawa M. Effect of whole-body vibration exercise on lumbar bone mineral density, bone turnover, and chronic back pain in post-menopausal osteoporotic women treated with alendronate. Aging Clinical and Experimental Research 2005 Apr;17(2):157-163. PMID: 15977465.
598. Jackson KJ, Merriman HL, Vanderburgh PM, Brahler CJ. Acute effects of whole-body vibration on lower extremity muscle performance in persons with multiple sclerosis. Journal of Neurologic Physical Therapy 2008 Dec;32(4):171-176. doi: 10.1097/NPT.0b013e31818ee760. PMID: 19265758.
599. Jackson LD. Maximizing treatment adherence among back-pain patients: an experimental study of the effects of physician-related cues in written medical messages. Health Communication 1994 Jul;6(3):173-191. doi: 10.1207/s15327027hc0603_1.
600. Jackson RA, Stotland NE, Caughey AB, Gerbert B. Improving diet and exercise in pregnancy with video doctor counseling: a randomized trial. Patient Education and Counseling 2011 May;83(2):203-209. doi: 10.1016/j.pec.2010.05.019. PMID: 21459255.
601. Jakeman JR, Macrae R, Eston R. A single 10-min bout of cold-water immersion therapy after strenuous plyometric exercise has no beneficial effect on recovery from the symptoms of exercise-induced muscle damage. Ergonomics 2009;52(4):456-460. doi: 10.1080/00140130802707733. PMID: 19401897.
602. Jamtvedt G, Herbert RD, Flottorp S, Odgaard-Jensen J, Havelsrud K, Barratt A, Mathieu E, Burls A, Oxman AD. A pragmatic randomised trial of stretching before and after physical activity to prevent injury and soreness. British Journal of Sports Medicine 2010 Nov;44(14):1002-1009. doi: 10.1136/bjsm.2009.062232. PMID: 19525241.
603. Jan M-H, Lin C-H, Lin Y-F, Lin J-J, Lin D-H. Effects of weight-bearing versus nonweight-bearing exercise on function, walking speed, and position sense in participants with knee osteoarthritis: a randomized controlled trial. Archives of Physical Medicine and Rehabilitation 2009 Jun;90(6):897-904. doi: 10.1016/j.apmr.2008.11.018. PMID: 19480863.
604. Jan M-H, Lin J-J, Liau J-J, Lin Y-F, Lin D-H. Investigation of clinical effects of high- and low-resistance training for patients with knee osteoarthritis: a randomized controlled trial. Physical Therapy 2008 Apr;88(4):427-436. doi: 10.2522/ptj.20060300. PMID: 18218827.
605. Janssen RG, Schwartz DA, Velleman PF. A randomized controlled study of contrast baths on patients with carpal tunnel syndrome. Journal of Hand Therapy 2009 Jul-Sep;22(3):200-208. doi: 10.1016/j.jht.2009.02.001. PMID: 19375278.
606. Jarden M, Baadsgaard MT, Hovgaard DJ, Boesen E, Adamsen L. A randomized trial on the effect of a multimodal intervention on physical capacity, functional performance and quality of life in adult patients undergoing allogeneic SCT. Bone Marrow Transplantation 2009 May;43(9):725-737. doi: 10.1038/bmt.2009.27. PMID: 19234513.
607. Jarvis SK, Hallam TK, Lujic S, Abbott JA, Vancaillie TG. Peri-operative physiotherapy improves outcomes for women undergoing incontinence and or prolapse surgery: results of a randomised controlled trial. Australian & New Zealand Journal of Obstetrics & Gynaecology 2005 Aug;45(4):300-303. doi: 10.1111/j.1479-828X.2005.00415.x. PMID: 16029296.
608. Jason LA, Torres-Harding S, Friedberg F, Corradi K, Njoku MG, Donalek J, Reynolds N, Brown M, Weitner BB, Rademaker A, Papernik M. Non-pharmacologic interventions for CFS: a randomized trial. Journal of Clinical Psychology in Medical Settings 2007 Dec;14(4):275-296. doi: 10.1007/s10880-007-9090-7.
609. Jeffries K, Shub A, Walker SP, Hiscock R, Permezel M. Reducing excessive weight gain in pregnancy: a randomised controlled trial. The Medical Journal of Australia 2009 Oct 19;191(8):429-433. PMID: 19835535.
610. Jellema P, van der Windt DA, van der Horst HE, Twisk JW, Stalman WA, Bouter LM. Should treatment of (sub)acute low back pain be aimed at psychosocial prognostic factors? Cluster randomised clinical trial in general practice. BMJ 2005 Jul 9;331(7508):84-90. doi: 10.1136/bmj.38495.686736.E0. PMID: 15967762.
611. Jenkinson CM, Doherty M, Avery AJ, Read A, Taylor MA, Sach TH, Silcocks P, Muir KR. Effects of dietary intervention and quadriceps strengthening exercises on pain and function in overweight people with knee pain: randomised controlled trial. BMJ 2009 Aug 18;339:b3170. doi: 10.1136/bmj.b3170. PMID: 19690345.
612. Jensen C, Jensen OK, Christiansen DH, Nielsen CV. One-year follow-up in employees sick-listed because of low back pain: randomized clinical trial comparing multidisciplinary and brief intervention. Spine 2011 Jul 1;36(15):1180-1189. doi: 10.1097/BRS.0b013e3181eba711. PMID: 21217456.
613. Jensen IB, Bergstrom G, Ljungquist T, Bodin L, Nygren AL. A randomized controlled component analysis of a behavioral medicine rehabilitation program for chronic spinal pain: are the effects dependent on gender?. Pain 2001 Mar;91(1-2):65-78. PMID: 11240079.
614. Jensen LD, Gonge H, Jors E, Ryom P, Foldspang A, Christensen M, Vesterdorf A, Bonde JP. Prevention of low back pain in female eldercare workers: randomized controlled work site trial. Spine 2006 Jul 15;31(16):1761-1769. doi: 10.1097/01.brs.0000227326.35149.38. PMID: 16845347.
615. Jensen P, Kenny D. The effects of yoga on the attention and behavior of boys with attention-deficit/hyperactivity disorder (ADHD). Journal of Attention Disorders 2004 May;7(4):205-216. doi: 10.1177/108705470400700403. PMID: 15487477.
616. Jessep SA, Walsh NE, Ratcliffe J, Hurley MV. Long-term clinical benefits and costs of an integrated rehabilitation programme compared with outpatient physiotherapy for chronic knee pain. Physiotherapy 2009 Jun;95(2):94-102. doi: 10.1016/j.physio.2009.01.005. PMID: 19627690.
617. Johannsen F, Remvig L, Kryger P, Beck P, Lybeck K, Larsen LH, Warming S, Dreyer V. Supervised endurance exercise training compared to home training after first lumbar diskectomy: a clinical trial. Clinical and Experimental Rheumatology 1994 Nov-Dec;12(6):609-614. PMID: 7895394.
618. Johansen KL, Painter PL, Sakkas GK, Gordon P, Doyle J, Shubert T. Effects of resistance exercise training and nandrolone decanoate on body composition and muscle function among patients who receive hemodialysis: a randomized, controlled trial. Journal of the American Society of Nephrology 2006 Aug;17(8):2307-2314. doi: 10.1681/ASN.2006010034. PMID: 16825332.
619. Johansson A-C, Linton SJ, Bergkvist L, Nilsson O, Cornefjord M. Clinic-based training in comparison to home-based training after first-time lumbar disc surgery: a randomised controlled trial. European Spine Journal 2009 Mar;18(3):398-409. doi: 10.1007/s00586-008-0826-3. PMID: 19020904.
620. Johansson PH, Lindstrom L, Sundelin G, Lindstrom B. The effects of preexercise stretching on muscular soreness, tenderness and force loss following heavy eccentric exercise. Scandinavian Journal of Medicine & Science in Sports 1999 Aug;9(4):219-225. doi: 10.1111/j.1600-0838.1999.tb00237.x. PMID: 10407930.
621. Johnson JL, King BT. Biofeedback versus verbal instruction for pelvic floor training in the treatment of urinary incontinence. Journal of Women's Health Physical Therapy 2000 Sep-Dec;24(3):7-13.
622. Johnson NA, Sachinwalla T, Walton DW, Smith K, Armstrong A, Thompson MW, George J. Aerobic exercise training reduces hepatic and visceral lipids in obese individuals without weight loss. Hepatology 2009 Oct;50(4):1105-1112. doi: 10.1002/hep.23129. PMID: 19637289.
623. Johnson RE, Jones GT, Wiles NJ, Chaddock C, Potter RG, Roberts C, Symmons DP, Watson PJ, Torgerson DJ, Macfarlane GJ. Active exercise, education, and cognitive behavioral therapy for persistent disabling low back pain: a randomized controlled trial. Spine 2007 Jul 1;32(15):1578-1585. doi: 10.1097/BRS.0b013e318074f890. PMID: 17621203.
624. Johnson SS, Paiva AL, Cummins CO, Johnson JL, Dyment SJ, Wright JA, Prochaska JO, Prochaska JM, Sherman K. Transtheoretical model-based multiple behavior intervention for weight management: effectiveness on a population basis. Preventive Medicine 2008 Mar;46(3):238-246. doi: 10.1016/j.ypmed.2007.09.010. PMID: 18055007.
625. Johnson VY. Effects of submaximal exercise protocol to recondition the pelvic floor musculature. Nursing Research 2001 Jan-Feb;50(1):33-41. PMID: 19785243.
626. Jolly K, Taylor R, Lip GY, Greenfield S, Raftery J, Mant J, Lane D, Jones M, Lee KW, Stevens A. The Birmingham Rehabilitation Uptake Maximisation Study (BRUM). Home-based compared with hospital-based cardiac rehabilitation in a multi-ethnic population: cost-effectiveness and patient adherence. Health Technology Assessment (Winchester, England) 2007 Sep;11(35):1-118. doi: 10.3310/hta11350. PMID: 17767899.
627. Jones D, Hoelscher DM, Kelder SH, Hergenroeder A, Sharma SV. Increasing physical activity and decreasing sedentary activity in adolescent girls -- the Incorporating More Physical Activity and Calcium in Teens (IMPACT) study. The International Journal of Behavioral Nutrition and Physical Activity 2008 Aug 21;5(42):Epub. doi: 10.1186/1479-5868-5-42. PMID: 18718012.
628. Jones KD, Burckhardt CS, Clark SR, Bennett RM, Potempa KM. A randomized controlled trial of muscle strengthening versus flexibility training in fibromyalgia. The Journal of Rheumatology 2002 May;29(5):1041-1048. PMID: 12022321.
629. Jongbloed L, Stacey S, Brighton C. Stroke rehabilitation: sensorimotor integrative treatment versus functional treatment. The American Journal of Occupational Therapy 1989 Jun;43(6):391-397. doi: 10.5014/ajot.43.6.391. PMID: 2741997.
630. Joseph AC, Chang MK. Comparison of behavior therapy methods for urinary incontinence following prostate surgery: a pilot study. Urologic Nursing 2000 Jun;20(3):203-204. PMID: 11998139.
631. Ju S, Park G, Kim E. Effects of an exercise treatment program on lumbar extensor muscle strength and pain of rehabilitation patients recovering from lumbar disc herniation surgery. Journal of Physical Therapy Science 2012 Jul;24(6):515-518. doi: 10.1589/jpts.24.515.
632. Juhakoski R, Tenhonen S, Malmivaara A, Kiviniemi V, Anttonen T, Arokoski JP. A pragmatic randomized controlled study of the effectiveness and cost consequences of exercise therapy in hip osteoarthritis. Clinical Rehabilitation 2011 Apr;25(4):370-383. doi: 10.1177/0269215510388313. PMID: 21078702.
633. Jung R, Wight J, Nusser R, Rosoff L. Comparison of three methods of respiratory care following upper abdominal surgery. Chest 1980 Jul;78(1):31-35. doi: 10.1378/chest.78.1.31. PMID: 7009083.
634. Kakkos SK, Geroulakos G, Nicolaides AN. Improvement of the walking ability in intermittent claudication due to superficial femoral artery occlusion with supervised exercise and pneumatic foot and calf compression: a randomised controlled trial. European Journal of Vascular and Endovascular Surgery 2005;30(2):164-175. doi: 10.1016/j.ejvs.2005.03.011. PMID: 15890545.
635. Kaltsatou A, Mameletzi D, Douka S. Physical and psychological benefits of a 24-week traditional dance program in breast cancer survivors. Journal of Bodywork and Movement Therapies 2011 Apr;15(2):162-167. doi: 10.1016/j.jbmt.2010.03.002. PMID: 21419356.
636. Kalus SM, Kornman LH, Quinlivan JA. Managing back pain in pregnancy using a support garment: a randomised trial. BJOG 2008 Jan;115(1):68-75. doi: 10.1111/j.1471-0528.2007.01538.x. PMID: 17999695.
637. Kamada M, Kitayuguchi J, Inoue S, Ishikawa Y, Nishiuchi H, Okada S, Harada K, Kamioka H, Shiwaku K. A community-wide campaign to promote physical activity in middle-aged and elderly people: a cluster randomized controlled trial. The International Journal of Behavioral Nutrition and Physical Activity 2013 Apr 9;10(44):Epub. doi: 10.1186/1479-5868-10-44. PMID: 23570536.
638. Kamwendo K, Linton SJ. A controlled study of the effect of neck school in medical secretaries. Scandinavian Journal of Rehabilitation Medicine 1991;23(3):143-152. PMID: 1962157.
639. Kang H-K, Kim Y, Chung Y, Hwang S. Effects of treadmill training with optic flow on balance and gait in individuals following stroke: randomized controlled trials. Clinical Rehabilitation 2012 Mar;26(3):246-255. doi: 10.1177/0269215511419383. PMID: 21971754.
640. Kanlayanaphotporn R, Chiradejnant A, Vachalathiti R. The immediate effects of mobilization technique on pain and range of motion in patients presenting with unilateral neck pain: a randomized controlled trial. Archives of Physical Medicine and Rehabilitation 2009 Feb;90(2):187-192. doi: 10.1016/j.apmr.2008.07.017. PMID: 19236972.
641. Kanlayanaphotporn R, Chiradejnant A, Vachalathiti R. Immediated effects of the central posteroanterior mobilization technique on pain and range of motion in patients with mechanical neck pain. Disability and Rehabilitation 2010;32(8):622-628. doi: 10.3109/09638280903204716. PMID: 20205574.
642. Kao M-J, Wu M-P, Tsai M-W, Chang W-W, Wu S-F. The effectiveness of a self-management program on quality of life for knee osteoarthritis (OA) patients. Archives of Gerontology and Geriatrics 2012 Mar-Apr;54(2):317-324. doi: 10.1016/j.archger.2011.05.018. PMID: 21726907.
643. Karapolat H, Demir E, Bozkaya YT, Eyigor S, Nalbantgel S, Durmaz B, Zoghi M. Comparison of hospital-based versus home-based exercise training in patients with heart failure: effects on functional capacity, quality of life, psychological symptoms, and hemodynamic parameters. Clinical Research in Cardiology 2009 Oct;98(10):635-642. doi: 10.1007/s00392-009-0049-6. PMID: 19641843.
644. Karinkanta S, Heinonen A, Sievanen H, Uusi-Rasi K, Pasanen M, Ojala K, Fogelholm M, Kannus P. A multi-component exercise regimen to prevent functional decline and bone fragility in home-dwelling elderly women: randomized, controlled trial. Osteoporosis International 2007 Apr;18(4):453-462. doi: 10.1007/s00198-006-0256-1. PMID: 17103296.
645. Karjalainen K, Malmivaara A, Mutanen P, Roine R, Hurri H, Pohjolainen T. Mini-intervention for subacute low back pain: two-year follow-up and modifiers of effectiveness. Spine 2004 May 15;29(10):1069-1076. PMID: 15131431.
646. Karjalainen K, Malmivaara A, Pohjolainen T, Hurri H, Mutanen P, Rissanen P, Pahkajarvi H, Levon H, Karpoff H, Roine R. Mini-intervention for subacute low back pain: a randomized controlled trial. Spine 2003 Mar 15;28(6):533-540. PMID: 12642757.
647. Karl CA. The effect of an exercise programme on self-care activities for the institutionalized elderly. Journal of Gerontological Nursing 1982 May;8(5):282-285. PMID: 6918434.
648. Karlsson J, Lundin O, Lind K, Styf J. Early mobilization versus immobilization after ankle ligament stabilization. Scandinavian Journal of Medicine & Science in Sports 1999 Oct;9(5):299-303. doi: 10.1111/j.1600-0838.1999.tb00250.x. PMID: 10512212.
649. Karlsson J, Rudholm O, Bergsten T, Faxen E, Styf J. Early range of motion training after ligament reconstruction of the ankle joint. Knee Surgery, Sports Traumatology, Arthroscopy 1995;3(3):173-177. doi: 10.1007/BF01565478. PMID: 8821274.
650. Kashanian M, Akbari Z, Alizadeh MH. The effect of exercise on back pain and lordosis in pregnant women. International Journal of Gynaecology and Obstetrics 2009 Nov;107(2):160-161. doi: 10.1016/j.ijgo.2009.06.018. PMID: 19682681.
651. Kassaian M, Maleki M, Noohi F, Eftekharzadeh M, Arya A, Roshanali F, Momtahen M. Comparing effects of supervised versus home-based cardiac rehabilitation. Iranian Heart Journal 2000 Winter;1(2):95-100.
652. Katiyar SK, Bihari S. Role of Pranayama in the rehabilitation of COPD patients -- a randomized controlled study. Indian Journal of Allergy and Applied Immunology 2006;20(2):98-104.
653. Katz DL, Shuval K, Comerford BP, Faridi Z, Njike VY. Impact of an educational intervention on internal medicine residents' physical activity counselling: the pressure system model. Journal of Evaluation in Clinical Practice 2008 Apr;14(2):294-299. doi: 10.1111/j.1365-2753.2007.00853.x. PMID: 18324934.
654. Katz-Leurer M, Carmeli E, Shochina M. The effect of early aerobic training on independence six months post stroke. Clinical Rehabilitation 2003 Nov;17(7):735-741. doi: 10.1191/0269215503cr671oa. PMID: 14606739.
655. Kayo AH, Peccin MS, Sanches CM, Trevisani VFM. Effectiveness of physical activity in reducing pain in patients with fibromyalgia: a blinded randomized clinical trial. Rheumatology International 2012 Aug;32(8):2285-2292. doi: 10.1007/s00296-011-1958-z. PMID: 21594719.
656. Keefe FJ, Blumenthal J, Baucom D, Affleck G, Waugh R, Caldwell DS, Beaupre P, Kashikar-Zuck S, Wright K, Egert J, Lefebvre J. Effects of spouse-assisted coping skills training and exercise training in patients with osteoarthritic knee pain: a randomized controlled study. Pain 2004 Aug;110(3):539-549. doi: 10.1016/j.pain.2004.03.022. PMID: 15288394.
657. Keefe FJ, Caldwell DS, Baucom D, Salley A, Robinson E, Timmons K, Beaupre P, Weisberg J, Helms M. Spouse-assisted coping skills training in the management of osteoarthritic knee pain. Arthritis Care & Research 1996 Aug;9(4):279-291. PMID: 8997917.
658. Keefe FJ, Caldwell DS, Williams DA, Gil KM, Mitchell D, Robertson C, Martinez S, Nunley J, Beckham J, Crisson J, Helms M. Pain coping skills training in the management of osteoarthritic knee pain: a comparative study. Behavior Therapy 1990 Winter;21(1):49-62. doi: 10.1016/S0005-7894(05)80188-1.
659. Kellett KM, Kellett DA, Nordholm LA. Effects of an exercise program on sick leave due to back pain. Physical Therapy 1991 Apr;71(4):283-291. PMID: 1826175.
660. Kerr D, Ackland T, Maslen B, Morton A, Prince R. Resistance training over 2 years increases bone mass in calcium-replete postmenopausal women. Journal of Bone and Mineral Research 2001 Jan;16(1):175-181. doi: 10.1359/jbmr.2001.16.1.175. PMID: 11149482.
661. Kerse N, Peri K, Robinson E, Wilkinson T, von Randow M, Kiata L, Parsons J, Latham N, Parsons M, Willingale J, Brown P, Arroll B. Does a functional activity programme improve function, quality of life, and falls for residents in long term care? Cluster randomised controlled trial. BMJ 2008 Oct 18;337(7675):a1445. doi: 10.1136/bmj.a1445. PMID: 18845605.
662. Kierkegaard M, Harms-Ringdahl K, Edstrom L, Widen Holmqvist L, Tollback A. Feasibility and effects of a physical exercise programme in adults with myotonic dystrophy type 1: a randomized controlled pilot study. Journal of Rehabilitation Medicine 2011 Jul;43(8):695-702. doi: 10.2340/16501977-0833. PMID: 21670942.
663. Kihlstrand M, Stenman B, Nilsson S, Axelsson O. Water-gymnastics reduced the intensity of back/low back pain in pregnant women. Acta Obstetricia et Gynecologica Scandinavica 1999 Mar;78(3):180-185. doi: 10.1034/j.1600-0412.1999.780302.x. PMID: 10078577.
664. Kim CJ, Kang DH, Smith BA, Landers KA. Cardiopulmonary responses and adherence to exercise in women newly diagnosed with breast cancer undergoing adjuvant therapy. Cancer Nursing 2006 Mar-Apr;29(2):156-165. PMID: 16565627.
665. Kim CM, Eng JJ, MacIntyre DL, Dawson AS. Effects of isokinetic strength training on walking in persons with stroke: a double-blind controlled pilot study. Journal of Stroke & Cerebrovascular Diseases 2001 Nov;10(6):265-273. doi: 10.1053/jscd.2001.123775. PMID: 17903837.
666. Kim DI, Jeong JC, Kim KH, Rho JJ, Choi MS, Yoon SH, Choi S-M, Kang KW, Ahn HY, Lee MS. Acupuncture for hot flushes in perimenopausal and postmenopausal women: a randomised, sham-controlled trial. Acupuncture in Medicine 2011 Dec;29(4):249-256. doi: 10.1136/aim.2011.004085. PMID: 21653660.
667. Kim H, Yoshida H, Suzuki T. Effects of exercise treatment with or without heat and steam generating sheet on urine loss in community-dwelling Japanese elderly women with urinary incontinence. Geriatrics & Gerontology International 2011 Oct;11(4):452-459. doi: 10.1111/j.1447-0594.2011.00705.x. PMID: 21545385.
668. Kim KH, Kang KW, Kim DI, Kim HJ, Yoon HM, Lee JM, Jeong JC, Lee MS, Jung HJ, Choi SM. Effects of acupuncture on hot flashes in perimenopausal and postmenopausal women-a multicenter randomized clinical trial. Menopause 2010 Mar;17(2):269-280. doi: 10.1097/gme.0b013e3181bfac3b. PMID: 19907348.
669. King AC, Haskell WL, Taylor CB, Kraemer HC, de Busk RF. Group- versus home-based exercise training in healthy older men and women. A community-based clinical trial. JAMA 1991 Sep 18;266(11):1535-1542. doi: 10.1001/jama.1991.03470110081037. PMID: 1880885.
670. Kinion ES, Christie N, Villella AM. Promoting activity in the elderly through interdisciplinary linkages. Nursing Connections 1993 Fall;6(3):19-26. PMID: 8107894.
671. Kipping RR, Payne C, Lawlor DA. Randomised controlled trial adapting US school obesity prevention to England. Archives of Disease in Childhood 2008 Jun;93(6):469-473. doi: 10.1136/adc.2007.116970. PMID: 18252756.
672. Kjellby-Wendt G, Styf J. Early active training after lumbar discectomy. A prospective, randomized, and controlled study. Spine 1998 Nov 1;23(21):2345-2351. PMID: 9820916.
673. Klaber Moffett JA, Hughes GI, Griffiths P. An investigation of the effects of cervical traction. Part 1: clinical effectiveness. Clinical Rehabilitation 1990;4:205-211. doi: 10.1177/026921559000400304.
674. Klaber Moffett JA, Jackson DA, Richmond S, Hahn S, Coulton S, Farrin A, Manca A, Torgerson DJ. Randomised trial of a brief physiotherapy intervention compared with usual physiotherapy for neck pain patients: outcomes and patients' preference. BMJ 2005 Jan 8;330(7482):75-78. doi: 10.1136/bmj.38286.493206.82. PMID: 15585539.
675. Klein MH, Greist JH, Gurman AS, Neimeyer RA, Lesser DP, Bushnell NJ, Smith RE. A comparative outcome study of group psychotherapy versus exercise treatments for depression. International Journal of Mental Health 1985;13(3-4):148-176.
676. Klein RG, Eek BC. Low-energy laser treatment and exercise for chronic low back pain: double-blind controlled trial. Archives of Physical Medicine and Rehabilitation 1990 Jan;71(1):34-37. PMID: 2136991.
677. Klijn PHC, Oudshoorn A, van der Ent CK, van der Net J, Kimpen JL, Helders PJM. Effects of anaerobic training in children with cystic fibrosis: a randomized controlled study. Chest 2004 Apr;125(4):1299-1305. doi: 10.1378/chest.125.4.1299. PMID: 15078738.
678. Kluge J, Hall D, Louw Q, Theron G, Grove D. Specific exercises to treat pregnancy-related low back pain in a South African population. International Journal of Gynaecology and Obstetrics 2011 Jun;113(3):187-191. doi: 10.1016/j.ijgo.2010.10.030. PMID: 21458811.
679. Knapik JJ, Brosch LC, Venuto M, Swedler DI, Bullock SH, Gaines LS, Murphy RJ, Tchandja J, Jones BH. Effect on injuries of assigning shoes based on foot shape in air force basic training. American Journal of Preventive Medicine 2010 Jan;38(1 Suppl):S197-S211. doi: 10.1016/j.amepre.2009.10.013. PMID: 20117594.
680. Knapik JJ, Swedler DI, Grier TL, Hauret KG, Bullock SH, Williams KW, Darakjy SS, Lester ME, Tobler SK, Jones BH. Injury reduction effectiveness of selecting running shoes based on plantar shape. Journal of Strength & Conditioning Research 2009 May;23(3):685-697. doi: 10.1519/JSC.0b013e3181a0fc63. PMID: 19387413.
681. Knols RH, de Bruin ED, Uebelhart D, Aufdemkampe G, Schanz U, Stenner-Liewen F, Hitz F, Taverna C, Aaronson NK. Effects of an outpatient physical exercise program on hematopoietic stem-cell transplantation recipients: a randomized clinical trial. Bone Marrow Transplantation 2011 Sep;46(9):1245-1255. doi: 10.1038/bmt.2010.288. PMID: 21132025.
682. Knowler WC, Barrett-Connor E, Fowler SE, Hamman RF, Lachin JM, Walker EA, Nathan DM, Diabetes Prevention Program Research Group. Reduction in the incidence of type 2 diabetes with lifestyle intervention or metformin. The New England Journal of Medicine 2002 Feb 7;346(6):393-403. doi: 10.1056/NEJMoa012512. PMID: 11832527.
683. Knubben K, Reischies FM, Adli M, Schlattmann P, Bauer M, Dimeo F. A randomised, controlled study on the effects of a short-term endurance training programme in patients with major depression. British Journal of Sports Medicine 2007 Jan;41(1):29-33. doi: 10.1136/bjsm.2006.030130. PMID: 17062659.
684. Ko P-C, Liang C-C, Chang S-D, Lee J-T, Chao A-S, Cheng P-J. A randomized controlled trial of antenatal pelvic floor exercises to prevent and treat urinary incontinence. International Urogynecology Journal 2011 Jan;22(1):17-22. doi: 10.1007/s00192-010-1248-4. PMID: 20737135.
685. Koc Z, Ozcakir S, Sivrioglu K, Gurbet A, Kucukoglu S. Effectiveness of physical therapy and epidural steroid injections in lumbar spinal stenosis. Spine 2009 May 1;34(10):985-989. doi: 10.1097/BRS.0b013e31819c0a6b. PMID: 19404172.
686. Koes BW, Bouter LM, van Mameren H, Essers AH, Verstegen GM, Hofhuizen DM, Houben JP, Knipschild PG. The effectiveness of manual therapy, physiotherapy, and treatment by the general practitioner for nonspecific back and neck complaints. A randomized clinical trial. Spine 1992 Jan;17(1):28-35. PMID: 1531552.
687. Koff PB, Jones RH, Cashman JM, Voelkel NF, Vandivier RW. Proactive integrated care improves quality of life in patients with COPD. The European Respiratory Journal 2009 May;33(5):1031-1038. doi: 10.1183/09031936.00063108. PMID: 19129289.
688. Kogstad OA, Karterud S, Gudmundsen J. Cervicobrachialgi. Et kontrollert forsook med konvensjonell behandling og manipulasjon (Cervicobrachialgia. A controlled trial with conventional therapy and manipulation) [Norwegian]. Tidsskrift for den Norske Laegeforening 1978 Jun 10;98(16):845-848. PMID: 354088.
689. Koh KP, Fassett RG, Sharman JE, Coombes JS, Williams AD. Effect of intradialytic versus home-based aerobic exercise training on physical function and vascular parameters in hemodialysis patients: a randomized pilot study. American Journal of Kidney Diseases 2010 Jan;55(1):88-99. doi: 10.1053/j.ajkd.2009.09.025. PMID: 19932545.
690. Kong KL, Campbell CG, Foster RC, Peterson AD, Lanningham-Foster L. A pilot walking program promotes moderate-intensity physical activity during pregnancy. Medicine and Science in Sports and Exercise 2014 Apr;46(3):462-471. doi: 10.1249/MSS.0000000000000141. PMID: 24002348.
691. Kongsted A, Qerama E, Kasch H, Bach FW, Korsholm L, Jensen TS, Bendix T. Education of patients after whiplash injury: is oral advice any better than a pamphlet?. Spine 2008 Oct 15;33(22):E843-E848. doi: 10.1097/BRS.0b013e318182bee2. PMID: 18824949.
692. Kongsted A, Qerama E, Kasch H, Bendix T, Winther F, Korsholm L, Jensen TS. Neck collar, 'act-as-usual' or active mobilization for whiplash injury: a randomized parallel-group trial. Spine 2007 Mar 15;32(6):618-626. doi: 10.1097/01.brs.0000257535.77691.bd. PMID: 17413465.
693. Konrad K, Tatrai T, Hunka A, Vereckei E, Korondi I. Controlled trial of balneotherapy in treatment of low back pain. Annals of the Rheumatic Diseases 1992 Jun;51(6):820-822. doi: 10.1136/ard.51.6.820. PMID: 1535495.
694. Konstantinidou E, Apostolidis A, Kondelidis N, Tsimtsiou Z, Hatzichristou D, Ioannides E. Short-term efficacy of group pelvic floor training under intensive supervision versus unsupervised home training for female stress urinary incontinence: a randomized pilot study. Neurourology and Urodynamics 2007;26(4):486-491. doi: 10.1002/nau.20380. PMID: 17245777.
695. Konstantinidou E, Koukouvou G, Kouidi E, Deligiannis A, Tourkantonis A. Exercise training in patients with end-stage renal disease on hemodialysis: comparison of three rehabilitation programs. Journal of Rehabilitation Medicine 2002 Jan;34(1):40-45. doi: 10.1080/165019702317242695. PMID: 11900261.
696. Koo D, So SM, Lim JS. (Effect of extracorporeal magnetic innervation (ExMI) pelvic floor therapy on urinary incontinence after radical prostatectomy) [Korean]. Korean Journal of Urology 2009 Jan;50(1):23-27. doi: 10.4111/kju.2009.50.1.23.
697. Kool JP, Oesch PR, Bachmann S, Knuesel O, Dierkes JG, Russo M, de Bie RA, van den Brandt PA. Increasing days at work using function-centered rehabilitation in nonacute nonspecific low back pain: a randomized controlled trial. Archives of Physical Medicine and Rehabilitation 2005 May;86(5):857-864. doi: 10.1016/j.apmr.2004.10.044. PMID: 15895328.
698. Kopple JD, Wang H, Casaburi R, Fournier M, Lewis MI, Taylor W, Storer TW. Exercise in maintenance hemodialysis patients induces transcriptional changes in genes favoring anabolic muscle. Journal of the American Society of Nephrology 2007 Nov;18(11):2975-2986. doi: 10.1681/ASN.2006070794. PMID: 17942969.
699. Korpelainen R, Keinanen-Kiukaanniemi S, Heikkinen J, Vaananen K, Korpelainen J. Effect of impact exercise on bone mineral density in elderly women with low BMD: a population-based randomized controlled 30-month intervention. Osteoporosis International 2006 Jan;17(1):109-118. doi: 10.1007/s00198-005-1924-2. PMID: 15889312.
700. Korpi-Hyovalti EAL, Laaksonen DE, Schwab US, Vanhapiha TH, Vihla KR, Heinonen ST, Niskanen LK. Feasibility of a lifestyle intervention in early pregnancy to prevent deterioration of glucose tolerance. BMC Public Health 2011 Mar 24;11(179):Epub. doi: 10.1186/1471-2458-11-179. PMID: 21429234.
701. Kosaka K, Noda M, Kuzuya T. Prevention of type 2 diabetes by lifestyle intervention: a Japanese trial in IGT males. Diabetes Research and Clinical Practice 2005 Feb;67(2):152-162. doi: 10.1016/j.diabres.2004.06.010. PMID: 15649575.
702. Kostopoulos D, Nelson AJ Jr, Ingber RS, Larkin RW. Reduction of spontaneous electrical activity and pain perception of trigger points in the upper trapezius muscle through trigger point compression and passive stretching. Journal of Musculoskeletal Pain 2008;16(4):266-278. doi: 10.1080/10582450802479594.
703. Koufaki P, Mercer TH, Naish PF. Effects of exercise training on aerobic and functional capacity of end-stage renal disease patients. Clinical Physiology and Functional Imaging 2002;22(2):115-124. doi: 10.1046/j.1365-2281.2002.00405.x. PMID: 12005153.
704. Kouidi E, Iacovides A, Iordanidis P, Vassiliou S, Deligiannis A, Ierodiakonou C, Tourkantonis A. Exercise renal rehabilitation program: psychosocial effects. Nephron 1997;77(2):152-158. PMID: 9346380.
705. Kouidi EJ, Grekas DM, Deligiannis AP. Effects of exercise training on noninvasive cardiac measures in patients undergoing long-term hemodialysis: a randomized controlled trial. American Journal of Kidney Diseases 2009 Sep;54(3):511-521. doi: 10.1053/j.ajkd.2009.03.009. PMID: 19646801.
706. Kovar PA, Allegrante JP, MacKenzie CR, Peterson MG, Gutin B, Charlson ME. Supervised fitness walking in patients with osteoarthritis of the knee. A randomized, controlled trial. Annals of Internal Medicine 1992 Apr 1;116(7):529-534. doi: 10.7326/0003-4819-116-7-529. PMID: 1543305.
707. Kraus JF, Schaffer KB, Rice T, Maroosis J, Harper J. A field trial of back belts to reduce the incidence of acute low back injuries in New York City home attendants. International Journal of Occupational and Environmental Health 2002 Apr;8(2):97-104. PMID: 12019686.
708. Krauss J, Creighton D, Ely JD, Podlewska-Ely J. The immediate effects of upper thoracic translatoric spinal manipulation on cervical pain and range of motion: a randomized clinical trial. The Journal of Manual & Manipulative Therapy 2008;16(2):93-99. PMID: 19119394.
709. Kriemler S, Zahner L, Schindler C, Meyer U, Hartmann T, Hebestreit H, Brunner-la Rocca HP, van Mechelen W, Puder JJ. Effect of school based physical activity programme (KISS) on fitness and adiposity in primary schoolchildren: cluster randomised controlled trial. BMJ 2010 Feb 23;340:c785. doi: 10.1136/bmj.c785. PMID: 20179126.
710. Kriska AM, Bayles C, Cauley JA, la Porte RE, Sandler RB, Pambianco G. A randomized exercise trial in older women: increased activity over two years and the factors associated with compliance. Medicine and Science in Sports and Exercise 1986 Oct;18(5):557-562. PMID: 3534509.
711. Kristiansen TK, Ryaby JP, McCabe J, Frey JJ, Roe LR. Accelerated healing of distal radial fractures with the use of specific, low-intensity ultrasound. A multicenter, prospective, randomized, double-blind, placebo-controlled study. Journal of Bone and Joint Surgery -- American Volume 1997 Jul;79(7):961-973. PMID: 9234872.
712. Krogh J, Saltin B, Gluud C, Nordentoft M. The DEMO trial: a randomized, parallel-group, observer-blinded clinical trial of strength versus aerobic versus relaxation training for patients with mild to moderate depression. The Journal of Clinical Psychiatry 2009 Jun;70(6):790-800. doi: 10.4088/JCP.08m04241. PMID: 19573478.
713. Kulig K, Beneck GJ, Selkowitz DM, Popovich JM Jr, Ge TT, Flanagan SP, Poppert EM, Yamada KA, Powers CM, Azen S, Winstein CJ, Gordon J, Samudrala S, Chen TC, Shamie AN, Khoo LT, Spoonamore MJ, Wang JC, Physical Therapy Clinical Research Network (PTClinResNet). An intensive, progressive exercise program reduces disability and improves functional performance in patients after single-level lumbar microdiskectomy. Physical Therapy 2009 Nov;89(11):1145-1157. doi: 10.2522/ptj.20080052. PMID: 19778981.
714. Kulkarni SR, Fletcher E, McConnell AK, Poskitt KR, Whyman MR. Pre-operative inspiratory muscle training preserves postoperative inspiratory muscle strength following major abdominal surgery -- a randomised pilot study. Annals of the Royal College of Surgeons of England 2010 Nov;92(8):700-705. doi: 10.1308/003588410X12771863936648. PMID: 20663275.
715. Kullenberg B, Ylipaa S, Soderlund K, Resch S. Postoperative cryotherapy after total knee arthroplasty: a prospective study of 86 patients. The Journal of Arthroplasty 2006 Dec;21(8):1175-1179. doi: 10.1016/j.arth.2006.02.159. PMID: 17162178.
716. Kumar PJ, McPherson EJ, Dorr LD, Wan Z, Baldwin K. Rehabilitation after total knee arthroplasty: a comparison of 2 rehabilitation techniques. Clinical Orthopaedics and Related Research 1996 Oct;(331):93-101. PMID: 8895624.
717. Kumnerddee W, Kaewtong A. Efficacy of acupuncture versus night splinting for carpal tunnel syndrome: a randomized clinical trial. Chotmaihet Thangphaet [Journal of the Medical Association of Thailand] 2010 Dec;93(12):1463-1469. PMID: 21344811.
718. Kuys SS, Brauer SG, Ada L. Higher-intensity treadmill walking during rehabilitation after stroke in feasible and not detrimental to walking pattern or quality: a pilot randomized trial. Clinical Rehabilitation 2011 Apr;25(4):316-326. doi: 10.1177/0269215510382928. PMID: 20921032.
719. Kvorning N, Holmberg C, Grennert L, Aberg A, Akeson J. Acupuncture relieves pelvic and low-back pain in late pregnancy. Acta Obstetricia et Gynecologica Scandinavica 2004 Mar;83(3):246-250. doi: 10.1111/j.0001-6349.2004.0215.x. PMID: 14995919.
720. Lambeek LC, van Mechelen W, Knol DL, Loisel P, Anema JR. Randomised controlled trial of integrated care to reduce disability from chronic low back pain in working and private life. BMJ 2010 Mar 16;340:c1035. doi: 10.1136/bmj.c1035. PMID: 20234040.
721. Lang EM, Nelson DL, Bush MA. Comparison of performance in materials-based occupation, imagery-based occupation, and rote exercise in nursing home residents. The American Journal of Occupational Therapy 1992 Jul;46(7):607-611. doi: 10.5014/ajot.46.7.607. PMID: 1621796.
722. Langhammer B, Lindmark B, Stanghelle JK. Stroke patients and long-term training: is it worthwhile: a randomized comparison of two different training strategies after rehabilitation. Clinical Rehabilitation 2007 Jun;21(6):495-510. doi: 10.1177/0269215507075207. PMID: 17613581.
723. Langlois F, Vu TTM, Chasse K, Dupuis G, Kergoat M-J, Bherer L. Benefits of physical exercise training on cognition and quality of life in frail older adults. The Journals of Gerontology. Series B, Psychological Sciences and Social Sciences 2013 May;68(3):400-404. doi: 10.1093/geronb/gbs069. PMID: 22929394.
724. Lappin MS, Lawrie FW, Richards TL, Kramer ED. Effects of a pulsed electromagnetic therapy on multiple sclerosis fatigue and quality of life: a double-blind, placebo controlled trial. Alternative Therapies in Health and Medicine 2003 Jul-Aug;9(4):38-48. PMID: 12868251.
725. Larsen K, Weidich F, Leboeuf-Yde C. Can custom-made biomechanic shoe orthoses prevent problems in the back and lower extremities? A randomized, controlled intervention trial of 146 military conscripts. Journal of Manipulative and Physiological Therapeutics 2002 Jun;25(5):326-331. doi: 10.1067/mmt.2002.124419. PMID: 12072853.
726. Larsen OA, Lassen NA. Effect of daily muscular exercise in patients with intermittent claudication. Lancet 1966 Nov 19;288(7473):1093-1096. doi: 10.1016/S0140-6736(66)92191-X. PMID: 4162526.
727. Larsson U, Choler U, Lidstrom A, Lind G, Nachemson A, Nilsson B, Roslund J. Auto-traction for treatment of lumbago-sciatica. A multicentre controlled investigation. Acta Orthopaedica Scandinavica 1980 Oct;51(5):791-798. PMID: 6451138.
728. Lau EM, Woo J, Leung PC, Swaminathan R, Leung D. The effects of calcium supplementation and exercise on bone density in elderly Chinese women. Osteoporosis International 1992 Jul;2(4):168-173. doi: 10.1007/BF01623922. PMID: 1611221.
729. Lau HMC, Wing Chiu TT, Lam T-H. The effectiveness of thoracic manipulation on patients with chronic mechanical neck pain -- a randomized controlled trial. Manual Therapy 2011 Apr;16(2):141-147. doi: 10.1016/j.math.2010.08.003. PMID: 20813577.
730. Lau SK, Chiu KY. Use of continuous passive motion after total knee arthroplasty. The Journal of Arthroplasty 2001 Apr;16(3):336-339. doi: 10.1054/arth.2001.21453. PMID: 11307132.
731. Lauchlan DT, McCaul JA, McCarron T, Patil S, McManners J, McGarva J. An exploratory trial of preventative rehabilitation on shoulder disability and quality of life in patients following neck dissection surgery. European Journal of Cancer Care 2011 Jan;20(1):113-122. doi: 10.1111/j.1365-2354.2009.01149.x. PMID: 20148940.
732. Laurienzo CE, Sacomani CAR, Rodrigues TR, Zequi SC, Guimaraes GC, Lopes A. Results of preoperative electrical stimulation of pelvic floor muscles in the continence status following radical retropubic prostatectomy. International Brazilian Journal of Urology 2013 Mar-Apr;39(2):182-188. doi: 10.1590/S1677-5538.IBJU.2013.02.05. PMID: 23683683.
733. Lausin G, Gouilly P. Etude des effets de la ventilation dirigee abdomino-diaphragmatique (vdad) chez des patients BPCO de stade I et II (Study of the effects of controlled abdominodiaphragmatic ventilation in patients with level I and II COPD) [French]. Kinesitherapie La Revue 2009 Mar;9(87):29-38. doi: 10.1016/S1779-0123(09)70776-4.
734. Lavender SA, Lorenz EP, Andersson GBJ. Can a new behaviorally oriented training process to improve lifting technique prevent occupationally related back injuries due to lifting?. Spine 2007 Feb 15;32(4):487-494. doi: 10.1097/01.brs.0000255203.96898.f2. PMID: 17304142.
735. Laycock J, Brown J, Cusack C, Green S, Jerwood D, Mann K, McLachlan Z, Schofield A. Pelvic floor reeducation for stress incontinence: comparing three methods. British Journal of Community Nursing 2001 May;6(5):230-237. PMID: 11893948.
736. Laycock J, Jerwood D. Does pre-modulated interferential therapy cure genuine stress incontinence?. Physiotherapy 1993 Aug;79(8):553-560. doi: 10.1016/S0031-9406(10)60297-0.
737. Lazo M, Solga SF, Horska A, Bonekamp S, Diehl AM, Brancati FL, Wagenknecht LE, Pi-Sunyer FX, Kahn SE, Clark JM, for the Fatty Liver Subgroup of the Look AHEAD Research Group. Effect of a 12-month intensive lifestyle intervention on hepatic steatosis in adults with type 2 diabetes. Diabetes Care 2010 Oct;33(10):2156-2163. doi: 10.2337/dc10-0856. PMID: 20664019.
738. Lazowski D-A, Ecclestone NA, Myers AM, Paterson DH, Tudor-Locke C, Fitzgerald C, Jones G, Shima N, Cunningham DA. A randomized outcome evaluation of group exercise programs in long-term care institutions. The Journals of Gerontology. Series A, Biological Sciences and Medical Sciences 1999 Dec;54(12):M621-M628. PMID: 10647968.
739. Leaver AM, Maher CG, Herbert RD, Latimer J, McAuley JH, Jull G, Refshauge KM. A randomized controlled trial comparing manipulation with mobilization for recent onset neck pain. Archives of Physical Medicine and Rehabilitation 2010 Sep;91(9):1313-1318. doi: 10.1016/j.apmr.2010.06.006. PMID: 20801246.
740. Lee HJ, Park HJ, Chae Y, Kim SY, Kim SN, Kim ST, Kim JH, Yin CS, Lee H. Tai Chi Qigong for the quality of life of patients with knee osteoarthritis: a pilot, randomized, waiting list controlled trial. Clinical Rehabilitation 2009 Jun;23(6):504-511. doi: 10.1177/0269215508101746. PMID: 19389743.
741. Lee J-H, Han E-Y. A comparison of the effects of PNF, ESWT, and TPI on pain and function of patients with myofascial pain syndrome. Journal of Physical Therapy Science 2013 Mar;25(3):341-344. doi: 10.1589/jpts.25.341.
742. Lee LYK, Lee DTF, Woo J. Tai Chi and health-related quality of life in nursing home residents. Journal of Nursing Scholarship 2009 Mar;41(1):35-43. doi: 10.1111/j.1547-5069.2009.01249.x. PMID: 19335676.
743. Lee YK, KimC, Pyo JH, Kim CH, Ji JW. (Endurance exercise training before hemodialysis: an effective therapeutic modality for end-stage renal disease patients) [Korean]. Korean Journal of Nephrology 2001 Mar;20(2):290-297.
744. Leehey DJ, Moinuddin I, Bast JP, Qureshi S, Jelinek CS, Cooper C, Edwards LC, Smith BM, Collins EG. Aerobic exercise in obese diabetic patients with chronic kidney disease: a randomized and controlled pilot study. Cardiovascular Diabetology 2009 Dec 9;8(62):Epub. doi: 10.1186/1475-2840-8-62. PMID: 20003224.
745. Leermakers EA, Anglin K, Wing RR. Reducing postpartum weight retention through a correspondence intervention. International Journal of Obesity 1998 Nov;22(11):1103-1109. PMID: 9822949.
746. Leeuw M, Goossens ME, van Breukelen GJ, de Jong JR, Heuts PH, Smeets RJ, Koke AJ, Vlaeyen JW. Exposure in vivo versus operant graded activity in chronic low back pain patients: results of a randomized controlled trial. Pain 2008 Aug 15;138(1):192-207. doi: 10.1016/j.pain.2007.12.009. PMID: 18242858.
747. Legault C, Jennings JM, Katula JA, Dagenbach D, Gaussoin SA, Sink KM, Rapp SR, Rejeski WJ, Shumaker SA, Espeland MA, SHARP-P Study Group. Designing clinical trials for assessing the effects of cognitive training and physical activity interventions on cognitive outcomes: the Seniors Health and Activity Research Program Pilot (SHARP-P) study, a randomized controlled trial. BMC Geriatrics 2011 May 26;11(27):Epub. doi: 10.1186/1471-2318-11-27. PMID: 21615936.
748. Lehtonen H, Jarvinen TL, Honkonen S, Nyman M, Vihtonen K, Jarvinen M. Use of a cast compared with a functional ankle brace after operative treatment of an ankle fracture. A prospective, randomized study. Journal of Bone and Joint Surgery -- American Volume 2003 Feb;85(2):205-211. PMID: 12571295.
749. Leicht AS, Crowther RG, Golledge J. Influence of peripheral arterial disease and supervised walking on heart rate variability. Journal of Vascular Surgery 2011 Nov;54(5):1352-1359. doi: 10.1016/j.jvs.2011.05.027. PMID: 21784603.
750. Lennon O, Carey A, Gaffney N, Stephenson J, Blake C. A pilot randomized controlled trial to evaluate the benefit of the cardiac rehabilitation paradigm for the non-acute ischaemic stroke population. Clinical Rehabilitation 2008 Feb;22(2):125-133. doi: 10.1177/0269215507081580. PMID: 18212034.
751. Lenssen AF, de Bie RA, Bulstra SK, van Steyn MJA. Continuous passive motion (CPM) in rehabilitation following total knee arthroplasty: a randomised controlled trial. Physical Therapy Reviews 2003;8(3):123-129. doi: 10.1179/108331903225003019.
752. Lenssen TAF, van Steyn MJA, Crijns YHF, Waltje EMH, Roox GM, Geesink RJT, van den Brandt PA, de Bie RA. Effectiveness of prolonged use of continuous passive motion (CPM), as an adjunct to physiotherapy, after total knee arthroplasty. BMC Musculoskeletal Disorders 2008 Apr 29;9(60):Epub. doi: 10.1186/1471-2474-9-60. PMID: 18442423.
753. Letchuman R, Deusinger RH. Comparison of sacrospinalis myoelectric activity and pain levels in patients undergoing static and intermittent lumbar traction. Spine 1993 Aug;18(10):1361-1365. PMID: 8211369.
754. Leung K-S, Lee W-S, Tsui H-F, Liu PP-L, Cheung W-H. Complex tibial fracture outcomes following treatment with low-intensity pulsed ultrasound. Ultrasound in Medicine & Biology 2004 Mar;30(3):389-395. doi: 10.1016/j.ultrasmedbio.2003.11.008. PMID: 15063521.
755. Leventhal LC, de Oliveira SMJV, Nobre MRC, da Silva FMB. Perineal analgesia with an ice pack after spontaneous vaginal birth: a randomized controlled trial. Journal of Midwifery & Women's Health 2011 Mar-Apr;56(2):141-146. doi: 10.1111/j.1542-2011.2010.00018.x. PMID: 21429079.
756. Levy AS, Marmar E. The role of cold compression dressings in the postoperative treatment of total knee arthroplasty. Clinical Orthopaedics and Related Research 1993 Dec;(297):174-178. PMID: 7902225.
757. Li G, Tian D, Yu J, Li W, Meng J. Synergistic effects of compound physical factor treatment on neurological outcome after peripheral nerve entrapment surgery: a randomized controlled study. Neural Regeneration Research 2008 Jan;3(1):97-100.
758. Li Y-L. (Nutritional supplementation and respiratory gym in patients with chronic obstructive pulmonary disease) [Chinese - simplified characters]. Zhongguo Linchuang Kangfu [Chinese Journal of Clinical Rehabilitation] 2002 May 15;6(9):1260,1262.
759. Liaw MY, Lin MC, Cheng PT, Wong MK, Tang FT. Resistive inspiratory muscle training: its effectiveness in patients with acute complete cervical cord injury. Archives of Physical Medicine and Rehabilitation 2000 Jun;81(6):752-756. doi: 10.1016/S0003-9993(00)90106-0. PMID: 10857519.
760. Licciardone JC, Buchanan S, Hensel KL, King HH, Fulda KG, Stoll ST. Osteopathic manipulative treatment of back pain and related symptoms during pregnancy: a randomized controlled trial. American Journal of Obstetrics and Gynecology 2010 Jan;202(1):43.e1-43.e8. doi: 10.1016/j.ajog.2009.07.057. PMID: 19766977.
761. Licciardone JC, Stoll ST, Fulda KG, Russo DP, Siu J, Winn W, Swift J Jr. Osteopathic manipulative treatment for chronic low back pain: a randomized controlled trial. Spine 2003 Jul 1;28(13):1355-1362. PMID: 12838090.
762. Lidstrom A, Zachrisson M. Physical therapy on low back pain and sciatica. An attempt at evaluation. Scandinavian Journal of Rehabilitation Medicine 1970;2(1):37-42. PMID: 4257208.
763. Liebergall-Wischnitzer M, Hochner-Celnikier D, Lavy Y, Manor O, Arbel R, Paltiel O. Paula method of circular muscle exercises for urinary stress incontinence -- a clinical trial. International Urogynecology Journal and Pelvic Floor Dysfunction 2005 Oct;16(5):345-351. doi: 10.1007/s00192-004-1261-6. PMID: 15660184.
764. Liebergall-Wischnitzer M, Hochner-Celnikier D, Lavy Y, Manor O, Shveiky D, Paltiel O. Randomized trial of circular muscle versus pelvic floor training for stress urinary incontinence in women. Journal of Women's Health 2009 Mar;18(3):377-385. doi: 10.1089/jwh.2007.0740. PMID: 19281321.
765. Lim B-W, Hinman RS, Wrigley TV, Sharma L, Bennell KL. Does knee malalignment mediate the effects of quadriceps strengthening on knee adduction moment, pain, and function in medial knee osteoarthritis? A randomized controlled trial. Arthritis Care & Research 2008 Jul 15;59(7):943-951. doi: 10.1002/art.23823. PMID: 18576289.
766. Lin C-WC, Moseley AM, Haas M, Refshauge KM, Herbert RD. Manual therapy in addition to physiotherapy does not improve clinical or economic outcomes after ankle fracture. Journal of Rehabilitation Medicine 2008 Jun;40(6):433-439. doi: 10.2340/16501977-0187. PMID: 18509557.
767. Lin D-H, Lin C-HJ, Lin Y-F, Jan M-H. Efficacy of 2 non-weight-bearing interventions, proprioception training versus strength training, for patients with knee osteoarthritis: a randomized clinical trial. The Journal of Orthopaedic and Sports Physical Therapy 2009 Jun;39(6):450-457. doi: 10.2519/jospt.2009.2923. PMID: 19531879.
768. Lin JH, Shen T, Chung RCK, Chiu TTW. The effectiveness of Long's manipulation on patients with chronic mechanical neck pain: a randomized controlled trial. Manual Therapy 2013 Aug;18(4):308-315. doi: 10.1016/j.math.2012.11.005. PMID: 23352180.
769. Lindeman E, Leffers P, Spaans F, Drukker J, Reulen J, Kerckhoffs M, Koke A. Strength training in patients with myotonic dystrophy and hereditary motor and sensory neuropathy: a randomized clinical trial. Archives of Physical Medicine and Rehabilitation 1995 Jul;76(7):612-620. doi: 10.1016/S0003-9993(95)80629-6. PMID: 7605179.
770. Lindstrom I, Ohlund C, Eek C, Wallin L, Peterson LE, Nachemson A. Mobility, strength, and fitness after a graded activity program for patients with subacute low back pain. A randomized prospective clinical study with a behavioral therapy approach. Spine 1992 Jun;17(6):641-652. PMID: 1385658.
771. Linton SJ, Andersson T. Can chronic disability be prevented? A randomized trial of a cognitive-behavior intervention and two forms of information for patients with spinal pain. Spine 2000 Nov 1;25(21):2825-2831. PMID: 11064530.
772. Linton SJ, Boersma K, Jansson M, Overmeer T, Lindblom K, Vlaeyen JWS. A randomized controlled trial of exposure in vivo for patients with spinal pain reporting fear of work-related activities. European Journal of Pain 2008 Aug;12(6):722-730. doi: 10.1016/j.ejpain.2007.11.001. PMID: 18155934.
773. Linton SJ, Bradley LA, Jensen I, Spangfort E, Sundell L. The secondary prevention of low back pain: a controlled study with follow-up. Pain 1989 Feb;36(2):197-207. PMID: 2521930.
774. Litchke L, Lloyd L, Schmidt E, Russian C, Reardon R. Effects of concurrent respiratory resistance training on health-related quality of life in wheelchair rugby athletes: a pilot study. Topics in Spinal Cord Injury Rehabilitation 2012 Summer;18(3):264-272. doi: 10.1310/sci1803-264. PMID: 23459144.
775. Litchke LG, Russian CJ, Lloyd LK, Schmidt EA, Price L, Walker JL. Effects of respiratory resistance training with a concurrent flow device on wheelchair athletes. The Journal of Spinal Cord Medicine 2008;31(1):65-71. PMID: 18533414.
776. Little P, Roberts L, Bolwers H, Garwood J, Cantrell T, Langridge J, Chapman J. Should we give detailed advice and information booklets to patients with back pain? A randomized controlled factorial trial of a self-management booklet and doctor advice to take exercise for back pain. Spine 2001 Oct 1;26(19):2065-2072. PMID: 11698879.
777. Littlejohns P, Baveystock CM, Parnell H, Jones PW. Randomised controlled trial of the effectiveness of a respiratory health worker in reducing impairment, disability, and handicap due to chronic airflow limitation. Thorax 1991 Aug;46(8):559-564. doi: 10.1136/thx.46.8.559. PMID: 1926024.
778. Liu F, Yao L-P, Mai H-X, Liu H-L, Yuan J-L, Wang F-L, Nan L-N, Wang H. (Extracorporeal magnetic innervation in the treatment of urinary incontinence after radical prostatectomy) [Chinese - simplified characters]. Zhongguo Zuzhi Gongcheng yu Linchuang Kangfu [Journal of Clinical Rehabilitative Tissue Engineering Research] 2008 Apr 22;12(17):3289-3292.
779. Liu M-S, Huang H, Ke J-B, Geng L. (Relationship between gastrocnemius flexibility and lower extremity overuse injuries in physical fitness training) [Chinese - simplified characters]. Zhongguo Zuzhi Gongcheng yu Linchuang Kangfu [Journal of Clinical Rehabilitative Tissue Engineering Research] 2008 Jul 8;12(28):5541-5544.
780. Ljunggren AE, Walker L, Weber H, Amundsen T. Manual traction versus isometric exercises in patients with herniated intervertebral lumbar discs. Physiotherapy Theory and Practice 1992;8(4):207-213.
781. Ljunggren AE, Weber H, Kogstad O, Thom E, Kirkesola G. Effect of exercise on sick leave due to low back pain. A randomized, comparative, long-term study. Spine 1997 Jul 15;22(14):1610-1617. PMID: 9253097.
782. Ljunggren AE, Weber H, Larsen S. Autotraction versus manual traction in patients with prolapsed lumbar intervertebral discs. Scandinavian Journal of Rehabilitation Medicine 1984;16(3):117-124. PMID: 6494835.
783. Lloyd AR, Hickie I, Brockman A, Hickie C, Wilson A, Dwyer J, Wakefield D. Immunologic and psychologic therapy for patients with chronic fatigue syndrome: a double-blind, placebo-controlled trial. The American Journal of Medicine 1993 Feb;94(2):197-203. PMID: 8430715.
784. Logue E, Sutton K, Jarjoura D, Smucker W, Baughman K, Capers C. Transtheoretical model-chronic disease care for obesity in primary care: a randomized trial. Obesity Research 2005 May;13(5):917-927. doi: 10.1038/oby.2005.106. PMID: 15919846.
785. Loisel P, Abenhaim L, Durand P, Esdaile JM, Suissa S, Gosselin L, Simard R, Turcotte J, Lemaire J. A population-based, randomized clinical trial on back pain management. Spine 1997 Dec 15;22(24):2911-2918. PMID: 9431627.
786. Longo L, Tamburini A, Monti A. Treatment with 904 nm and 10,600 nm laser of acute lumbago -- double blind control-laser. Laser 1991;1(3):16-20.
787. Lord SR, Ward JA, Williams P, Zivanovic E. The effects of a community exercise program on fracture risk factors in older women. Osteoporosis International 1996 Sep;6(5):361-367. doi: 10.1007/BF01623009. PMID: 8931030.
788. Lorig KR, Ritter PL, Laurent DD, Plant K. The internet-based arthritis self-management program: a one-year randomized trial for patients with arthritis or fibromyalgia. Arthritis Care & Research 2008 Jul 15;59(7):1009-1017. doi: 10.1002/art.23817. PMID: 18576310.
789. Lovelady CA, Bopp MJ, Colleran HL, MacKie HK, Wideman L. Effect of exercise training on loss of bone mineral density during lactation. Medicine and Science in Sports and Exercise 2009 Oct;41(10):1902-1907. doi: 10.1249/MSS.0b013e3181a5a68b. PMID: 19727023.
790. Lovelady CA, Garner KE, Moreno KL, Williams JP. The effect of weight loss in overweight, lactating women on the growth of their infants. The New England Journal of Medicine 2000 Feb 17;342(7):449-453. doi: 10.1056/NEJM200002173420701. PMID: 10675424.
791. Loveridge B, Badour M, Dubo H. Ventilatory muscle endurance training in quadriplegia: effects on breathing pattern. Paraplegia 1989 Oct;27(5):329-339. PMID: 2689970.
792. Lubans DR, Morgan PJ, Callister R, Collins CE. Effects of integrating pedometers, parental materials, and E-mail support within an extracurricular school sport intervention. Journal of Adolescent Health 2009 Feb;44(2):176-183. doi: 10.1016/j.jadohealth.2008.06.020. PMID: 19167667.
793. Lubbert PH, van der Rijt RH, Hoorntje LE, van der Werken C. Low-intensity pulsed ultrasound (LIPUS) in fresh clavicle fractures: a multi-centre double blind randomised controlled trial. Injury 2008 Oct;39(10):1444-1452. doi: 10.1016/j.injury.2008.04.004. PMID: 18656872.
794. Luepker RV, Perry CL, McKinlay SM, Nader PR, Parcel GS, Stone EJ, Webber LS, Elder JP, Feldman HA, Johnson CC, Kelder SH, Wu M. Outcomes of a field trial to improve children's dietary patterns and physical activity. The Child and Adolescent Trial for Cardiovascular Health. CATCH collaborative group. JAMA 1996 Mar 13;275(10):768-776. doi: 10.1001/jama.1996.03530340032026. PMID: 8598593.
795. Lund H, Weile U, Christensen R, Rostock B, Downey A, Bartels EM, Danneskiold-Samsoe B, Bliddal H. A randomized controlled trial of aquatic and land-based exercise in patients with knee osteoarthritis. Journal of Rehabilitation Medicine 2008 Feb;40(2):137-144. doi: 10.2340/16501977-0134. PMID: 18509579.
796. Lund I, Lundeberg T, Lonnberg L, Svensson E. Decrease of pregnant women's pelvic pain after acupuncture: a randomized controlled single-blind study. Acta Obstetricia et Gynecologica Scandinavica 2006 Jan;85(1):12-19. doi: 10.1080/00016340500317153. PMID: 16521674.
797. Luoto R, Kinnunen T, Aittasalo M, Kolu P, Raitanen J, Ojala K, Mansikkamaki K, Lamberg S, Vasankari T, Komulainen T, Tulokas S. Primary prevention of gestational diabetes mellitus and large-for-gestational-age newborns by lifestyle counseling: a cluster-randomized controlled trial. PLoS Medicine 2011 May;8(5):e1001036. doi: 10.1371/journal.pmed.1001036. PMID: 21610860.
798. Lyager S, Wernberg M, Rajani N, Boggild-Madsen B, Nielsen L, Nielsen HC, Andersen M, Moller J, Silberschmid M. Can postoperative pulmonary conditions be improved by treatment with the Bartlett-Edwards incentive spirometer after upper abdominal surgery?. Acta Anaesthesiologica Scandinavica 1979 Aug;23(4):312-319. doi: 10.1111/j.1399-6576.1979.tb01456.x. PMID: 495034.
799. MacDonald SJ, Bourne RB, Rorabeck CH, McCalden RW, Kramer J, Vaz M. Prospective randomized clinical trial of continuous passive motion after total knee arthroplasty. Clinical Orthopaedics and Related Research 2000 Nov;(380):30-35. PMID: 11064970.
800. Maddalozzo GF, Widrick JJ, Cardinal BJ, Winters-Stone KM, Hoffman MA, Snow CM. The effects of hormone replacement therapy and resistance training on spine bone mineral density in early postmenopausal women. Bone 2007 May;40(5):1244-1251. doi: 10.1016/j.bone.2006.12.059. PMID: 17291843.
801. Madden DJ, Blumenthal JA, Allen PA, Emery CF. Improving aerobic capacity in healthy older adults does not necessarily lead to improved cognitive performance. Psychology and Aging 1989 Sep;4(3):307-320. doi: 10.1037/0882-7974.4.3.307. PMID: 2803624.
802. Madjdinasab N, Zadeh NS, Assarzadegan F, Ali AMA, Pipelzadeh M. Efficacy comparison of splint and oral steroid therapy in nerve conduction velocity and latency median nerve in carpal tunnel syndrome. Pakistan Journal of Medical Sciences 2008 Oct-Dec;24(5 Pt 1):725-728.
803. Mador MJ, Krawza M, Alhajhusian A, Khan AI, Shaffer M, Kufel TJ. Interval training versus continuous training in patients with chronic obstructive pulmonary disease. Journal of Cardiopulmonary Rehabilitation and Prevention 2009 Mar-Apr;29(2):126-132. doi: 10.1097/HCR.0b013e31819a024f. PMID: 19305238.
804. Madson TJ, Cieslak KR, Gay RE. Joint mobilization versus massage for chronic mechanical neck pain: a pilot study to assess recruitment strategies and estimate outcome measure variability. Journal of Manipulative and Physiological Therapeutics 2010 Nov-Dec;33(9):644-651. doi: 10.1016/j.jmpt.2010.08.008. PMID: 21109054.
805. Maisiak R, Austin J, Heck L. Health outcomes of two telephone interventions for patients with rheumatoid arthritis or osteoarthritis. Arthritis and Rheumatism 1996 Aug;39(8):1391-1399. doi: 10.1002/art.1780390818. PMID: 8702449.
806. Makita M, Nakadaira H, Yamamoto M. Randomized controlled trial to evaluate effectiveness of exercise therapy (Takizawa Program) for frail elderly. Environmental Health and Preventive Medicine 2006 Sep;11(5):221-227. doi: 10.1007/BF02898010. PMID: 21432349.
807. Makuloluwe RTB, Mouzas GL. Ultrasound in the treatment of sprained ankles. The Practitioner 1977;218:586-588. PMID: 323842.
808. Malliaropoulos N, Papalexandris S, Papalada A, Papacostas E. The role of stretching in rehabilitation of hamstring injuries: 80 athletes follow-up. Medicine and Science in Sports and Exercise 2004 May;36(5):756-759. doi: 10.1249/01.MSS.0000126393.20025.5E. PMID: 15126706.
809. Malmivaara A, Hakkinen U, Aro T, Heinrichs ML, Koskenniemi L, Kuosma E, Lappi S, Paloheimo R, Servo C, Vaaranen V, Hernberg S. The treatment of acute low back pain -- bed rest, exercises, or ordinary activity?. The New England Journal of Medicine 1995 Feb 9;332(6):351-355. doi: 10.1056/NEJM199502093320602. PMID: 7823996.
810. Malmivaara A, Slatis P, Heliovaara M, Sainio P, Kinnunen H, Kankare J, Dalin-Hirvonen N, Seitsalo S, Herno A, Kortekangas P, Niinimaki T, Ronty H, Tallroth K, Turunen V, Knekt P, Harkanen T, Hurri H, Finnish Lumbar Spinal Research Group. Surgical or nonoperative treatment for lumbar spinal stenosis? A randomized controlled trial. Spine 2007 Jan 1;32(1):1-8. doi: 10.1097/01.brs.0000251014.81875.6d. PMID: 17202885.
811. Malmros B, Mortensen L, Jensen MB, Charles P. Positive effects of physiotherapy on chronic pain and performance in osteoporosis. Osteoporosis International 1998 May;8(3):215-221. doi: 10.1007/s001980050057. PMID: 9797905.
812. Maltais F, Bourbeau J, Shapiro S, Lacasse Y, Perrault H, Baltzan M, Hernandez P, Rouleau M, Julien M, Parenteau S, Paradis B, Levy RD, Camp P, Lecours R, Audet R, Hutton B, Penrod JR, Picard D, Bernard S, Chronic Obstructive Pulmonary Disease Axis of Respiratory Health Network Fonds de Recherche en Sante du Quebec. Effects of home-based pulmonary rehabilitation in patients with chronic obstructive pulmonary disease: a randomized trial. Annals of Internal Medicine 2008 Dec 16;149(12):869-878. doi: 10.7326/0003-4819-149-12-200812160-00006. PMID: 19075206.
813. Manassero F, Traversi C, Ales V, Pistolesi D, Panicucci E, Valent F, Selli C. Contribution of early intensive prolonged pelvic floor exercises on urinary continence recovery after bladder neck-sparing radical prostatectomy: results of a prospective controlled randomized trial. Neurourology and Urodynamics 2007;26(7):985-989. doi: 10.1002/nau.20442. PMID: 17487874.
814. Manente G, Torrieri F, di Blasio F, Staniscia T, Romano F, Uncini A. An innovative hand brace for carpal tunnel syndrome: a randomized controlled trial. Muscle & Nerve 2001 Aug;24(8):1020-1025. doi: 10.1002/mus.1105. PMID: 11439376.
815. Maniar RN, Baviskar JV, Singhi T, Rathi SS. To use or not to use continuous passive motion post-total knee arthroplasty presenting functional assessment results in early recovery. The Journal of Arthroplasty 2012 Feb;27(2):193.e191-200.e191. doi: 10.1016/j.arth.2011.04.009. PMID: 21752575.
816. Manikandan N. Effect of facial neuromuscular re-education on facial symmetry in patients with Bell's palsy: a randomized controlled trial. Clinical Rehabilitation 2007 Apr;21(4):338-343. doi: 10.1177/0269215507070790. PMID: 17613574.
817. Mannarino E, Pasqualini L, Innocente S, Scricciolo V, Rignanese A, Ciuffetti G. Physical training and antiplatelet treatment in stage II peripheral arterial occlusive disease: alone or combined?. Angiology 1991 Jul;42(7):513-521. doi: 10.1177/000331979104200701. PMID: 1863010.
818. Manniche C, Asmussen K, Lauritsen B, Vinterberg H, Karbo H, Abildstrup S, Fischer-Nielsen K, Krebs R, Ibsen K. Intensive dynamic back exercises with or without hyperextension in chronic back pain after surgery for lumbar disc protrusion. A clinical trial. Spine 1993 Apr;18(5):560-567. PMID: 8484146.
819. Manniche C, Skall HF, Braendholt L, Christensen BH, Christophersen L, Ellegaard B, Heilbuth A, Ingerslev M, Jorgensen OE, Larsen E, Lorentzen L, Nielsen C, Nielsen J, Windelin M. Clinical trial of postoperative dynamic back exercises after first lumbar discectomy. Spine 1993 Jan;18(1):92-97. PMID: 8434331.
820. Mannion AF, Denzler R, Dvorak J, Muntener M, Grob D. A randomised controlled trial of post-operative rehabilitation after surgical decompression of the lumbar spine. European Spine Journal 2007 Aug;16(8):1101-1117. doi: 10.1007/s00586-007-0399-6. PMID: 17593405.
821. Marchese VG, Chiarello LA, Lange BJ. Effects of physical therapy intervention for children with acute lymphoblastic leukemia. Pediatric Blood & Cancer 2004 Feb;42(2):127-133. doi: 10.1002/pbc.10481. PMID: 14752875.
822. Marchionni N, Fattirolli F, Fumagalli S, Oldridge N, del Lungo F, Morosi L, Burgisser C, Masotti G. Improved exercise tolerance and quality of life with cardiac rehabilitation of older patients after myocardial infarction: results of a randomized, controlled trial. Circulation 2003 May 6;107(17):2201-2206. doi: 10.1161/01.CIR.0000066322.21016.4A. PMID: 12707240.
823. Marchiori D, Bertaccini A, Manferrari F, Ferri C, Martorana G. Pelvic floor rehabilitation for continence recovery after radical prostatectomy: role of a personal training re-educational program. Anticancer Research 2010 Feb;30(2):553-556. PMID: 20332469.
824. Mariotti G, Sciarra A, Gentilucci A, Salciccia S, Alfarone A, Pierro GD, Gentile V. Early recovery of urinary continence after radical prostatectomy using early pelvic floor electrical stimulation and biofeedback associated treatment. The Journal of Urology 2009 Apr;181(4):1788-1793. doi: 10.1016/j.juro.2008.11.104. PMID: 19233390.
825. Martel J, Dugas C, Dubois J-D, Descarreaux M. A randomised controlled trial of preventive spinal manipulation with and without a home exercise program for patients with chronic neck pain. BMC Musculoskeletal Disorders 2011 Feb 8;12(41):Epub. doi: 10.1186/1471-2474-12-41. PMID: 21303529.
826. Martin D, Notelovitz M. Effects of aerobic training on bone mineral density of postmenopausal women. Journal of Bone and Mineral Research 1993 Aug;8(8):931-936. doi: 10.1002/jbmr.5650080805. PMID: 8213255.
827. Martin DP, Sletten CD, Williams BA, Berger IH. Improvement in fibromyalgia symptoms with acupuncture: results of a randomized controlled trial. Mayo Clinic Proceedings 2006 Jun;81(6):749-757. doi: 10.4065/81.6.749. PMID: 16770975.
828. Martin IR, McNamara D, Sutherland FR, Tilyard MW, Taylor DR. Care plans for acutely deteriorating COPD: a randomized controlled trial. Chronic Respiratory Disease 2004 Oct;1(4):191-195. doi: 10.1191/1479972304cd047oa. PMID: 16281645.
829. Martinez Vizcaino V, Salcedo Aguilar F, Franquelo Gutierrez R, Solera Martinez M, Sanchez Lopez M, Serrano Martinez S, Lopez Garcia E, Rodriguez Artalejo F. Assessment of an after-school physical activity program to prevent obesity among 9- to 10-year-old children: a cluster randomized trial. International Journal of Obesity 2008 Jan;32(1):12-22. doi: 10.1038/sj.ijo.0803738. PMID: 17895883.
830. Martinez-Segura R, Fernandez-de-las-Penas C, Ruiz-Saez M, Lopez-Jimenez C, Rodriguez-Blanco C. Immediate effects on neck pain and active range of motion after a single cervical high-velocity low-amplitude manipulation in subjects presenting with mechanical neck pain: a randomized controlled trial. Journal of Manipulative and Physiological Therapeutics 2006 Sep;29(7):511-517. doi: 10.1016/j.jmpt.2006.06.022. PMID: 16949939.
831. Martins RF, Pinto e Silva JL. Tratamento da lombalgia e dor pelvica posterior na gestacao por um metodo de exercicios (An exercise method for the treatment of lumbar and posterior pelvic pain in pregnancy) [Portuguese]. Revista Brasileira de Ginecologia e Obstetricia 2005 May;27(5):275-282. doi: 10.1590/S0100-72032005000500008.
832. Martins RS, Siqueira MG, Simplicio H. Wrist immobilization after carpal tunnel release: a prospective study. Arquivos de Neuro-Psiquiatria 2006 Sep;64(3A):596-599. doi: 10.1590/S0004-282X2006000400013. PMID: 17119800.
833. Martinsen EW, Medhus A, Sandvik L. Effects of aerobic exercise on depression: a controlled study. British Medical Journal 1985 Jul 13;291(6488):109. PMID: 3926074.
834. Martire LM, Schulz R, Keefe FJ, Rudy TE, Starz TW. Couple-oriented education and support intervention: effects on individuals with osteoarthritis and their spouses. Rehabilitation Psychology 2007 May;52(2):121-132. doi: 10.1037/0090-5550.52.2.121.
835. Masaracchio M, Cleland JA, Hellman M, Hagins M. Short-term combined effects of thoracic spine thrust manipulation and cervical spine nonthrust manipulation in individuals with mechanical neck pain: a randomized clinical trial. The Journal of Orthopaedic and Sports Physical Therapy 2013 Mar;43(3):118-127. doi: 10.2519/jospt.2013.4221. PMID: 23221367.
836. Mather AS, Rodriguez C, Guthrie MF, McHarg AM, Reid IC, McMurdo MET. Effects of exercise on depressive symptoms in older adults with poorly responsive depressive disorder -- randomised controlled trial. British Journal of Psychiatry 2002;180:411-415. PMID: 11983637.
837. Mathews JA, Hickling J. Lumbar traction: a double-blind controlled study for sciatica. Rheumatology and Rehabilitation 1975 Nov;14(4):222-225. doi: 10.1093/rheumatology/14.4.222. PMID: 1105752.
838. Mathews W, Morkel M, Mathews J. Manipulation and traction for lumbago and sciatica: physiotherapeutic techniques used in two controlled trials. Physiotherapy Practice 1988;4(4):201-206.
839. Mathewson-Chapman M. Pelvic muscle exercise/biofeedback for urinary incontinence after prostatectomy: an education program. Journal of Cancer Education 1997 Winter;12(4):218-223. doi: 10.1080/08858199709528492. PMID: 9440013.
840. Matsumoto I, Araki H, Tsuda K, Odajima H, Nishima S, Higaki Y, Tanaka H, Tanaka M, Shindo M. Effects of swimming training on aerobic capacity and exercise induced bronchoconstriction in children with bronchial asthma. Thorax 1999 Mar;54(3):196-201. doi: 10.1136/thx.54.3.196. PMID: 10325893.
841. Matsumoto Y, Furuta A, Furuta S, Miyajima M, Sugino T, Nagata K, Sawada S. The impact of pre-dialytic endurance training on nutritional status and quality of life in stable hemodialysis patients (Sawada study). Renal Failure 2007;29(5):587-593. doi: 10.1080/08860220701392157. PMID: 17654322.
842. Maurer BT, Stern AG, Kinossian B, Cook KD, Schumacher HR. Osteoarthritis of the knee: isokinetic quadriceps exercise versus and educational intervention. Archives of Physical Medicine and Rehabilitation 1999 Oct;80(10):1293-1299. PMID: 10527090.
843. May LA, Busse W, Zayac D, Whitridge MR. Comparison of continuous passive motion (CPM) machines and lower limb mobility boards (LLiMB) in the rehabilitation of patients with total knee arthroplasty. Canadian Journal of Rehabilitation 1999;12(4):257-263.
844. Mayer JM, Ralph L, Look M, Erasala GN, Verna JL, Matheson LN, Mooney V. Treating acute low back pain with continuous low-level heat wrap therapy and/or exercise: a randomized controlled trial. The Spine Journal 2005 Jul-Aug;5(4):395-403. doi: 10.1016/j.spinee.2005.03.009. PMID: 15996609.
845. Mayr E, Rudzki MM, Rudzki M, Borchardt B, Hausser H, Ruter A. (Does low intensity, pulsed ultrasound speed healing of scaphoid fractures?) [German]. Handchirurgie, Mikrochirurgie, Plastische Chirurgie 2000 Mar;32(2):115-122. doi: 10.1055/s-2000-19253. PMID: 10857066.
846. Mazzuca SA, Brandt KD, Katz BP, Ragozzino LR, G'Sell PM. Can a nurse-directed intervention reduce the exposure of patients with knee osteoarthritis to nonsteroidal antiinflammatory drugs?. Journal of Clinical Rheumatology 2004 Dec;10(6):315-322. PMID: 17043539.
847. McCann IL, Holmes DS. Influence of aerobic exercise on depression. Journal of Personality & Social Psychology 1984 May;46(5):1142-1147. doi: 10.1037/0022-3514.46.5.1142. PMID: 6737208.
848. McCauley JD, Thelen MH, Frank RG, Willard RR, Callen KE. Hypnosis compared to relaxation in the outpatient management of chronic low back pain. Archives of Physical Medicine and Rehabilitation 1983 Nov;64(11):548-552. PMID: 6227304.
849. McClurg D, Ashe RG, Marshall K, Lowe-Strong AS. Comparison of pelvic floor muscle training, electromyography biofeedback, and neuromuscular electrical stimulation for bladder dysfunction in people with multiple sclerosis: a randomized pilot study. Neurourology and Urodynamics 2006;25(4):337-348. doi: 10.1002/nau.20209. PMID: 16637070.
850. McClurg D, Hagen S, Hawkins S, Lowe-Strong A. Abdominal massage for the alleviation of constipation symptoms in people with multiple sclerosis: a randomized controlled feasibility study. Multiple Sclerosis Journal 2011 Feb;17(2):223-233. doi: 10.1177/1352458510384899. PMID: 20940182.
851. McCrory MA, Nommsen-Rivers LA, Mole PA, Lonnerdal B, Dewey KG. Randomized trial of the short-term effects of dieting compared with dieting plus aerobic exercise on lactation performance. The American Journal of Clinical Nutrition 1999 May;69(5):959-967. PMID: 10232637.
852. McDermott MM, Liu K, Guralnik JM, Criqui MH, Spring B, Tian L, Domanchuk K, Ferrucci L, Lloyd-Jones D, Kibbe M, Tao H, Zhao L, Liao Y, Rejeski WJ. Home-based walking exercise intervention in peripheral artery disease: a randomized clinical trial. JAMA 2013 Jul 3;310(1):57-65. doi: 10.1001/jama.2013.7231. PMID: 23821089.
853. McEvoy RD, Pierce RJ, Hillman D, Esterman A, Ellis EE, Catcheside PG, O'Donoghue FJ, Barnes DJ, Grunstein RR, on behalf of the Australian trial of noninvasive Ventilation in Chronic Airflow Limitation (AVCAL) Study Group. Nocturnal non-invasive nasal ventilation in stable hypercapnic COPD: a randomised controlled trial. Thorax 2009 Jul;64(7):561-566. doi: 10.1136/thx.2008.108274. PMID: 19213769.
854. McGlynn GH, Laughlin NT, Rowe V. Effect of electromyographic feedback and static stretching on artificially induced muscle soreness. American Journal of Physical Medicine 1979 Jun;58(3):139-148. PMID: 453340.
855. McGregor AH, Dor CJ, Morris TP, Morris S, Jamrozik K. Function After Spinal Treatment, Exercise, and Rehabilitation (FASTER): a factorial randomized trial to determine whether the functional outcome of spinal surgery can be improved. Spine 2011 Oct 1;36(21):1711-1720. doi: 10.1097/BRS.0b013e318214e3e6. PMID: 21378603.
856. McInnes J, Larson MG, Daltroy LH, Brown T, Fossel AH, Eaton HM, Shulman-Kirwan B, Steindorf S, Poss R, Liang MH. A controlled evaluation of continuous passive motion in patients undergoing total knee arthroplasty. JAMA 1992 Sep 16;268(11):1423-1428. doi: 10.1001/jama.1992.03490110061030. PMID: 1512910.
857. McKenzie DC, Kalda AL. Effect of upper extremity exercise on secondary lymphedema in breast cancer patients: a pilot study. Journal of Clinical Oncology 2003 Feb 1;21(3):463-466. doi: 10.1200/JCO.2003.04.069. PMID: 12560436.
858. McKeon PO, Ingersoll CD, Kerrigan DC, Saliba E, Bennett BC, Hertel J. Balance training improves function and postural control in those with chronic ankle instability. Medicine and Science in Sports and Exercise 2008 Oct;40(10):1810-1819. doi: 10.1249/MSS.0b013e31817e0f92. PMID: 18799992.
859. McKinney LA, Dornan JO, Ryan M. The role of physiotherapy in the management of acute neck sprains following road-traffic accidents. Archives of Emergency Medicine 1989 Mar;6(1):27-33. PMID: 2712985.
860. McKnight PE, Kasle S, Going S, Villanueva I, Cornett M, Farr J, Wright J, Streeter C, Zautra A. A comparison of strength training, self-management, and the combination for early osteoarthritis of the knee. Arthritis Care & Research 2010 Jan;62(1):45-53. doi: 10.1002/acr.20013. PMID: 20191490.
861. McManus AM, Masters RSW, Laukkanen RMT, Yu CCW, Sit CHP, Ling FCM. Using heart-rate feedback to increase physical activity in children. Preventive Medicine 2008 Oct;47(4):402-408. doi: 10.1016/j.ypmed.2008.06.001. PMID: 18590757.
862. McMurdo ME, Rennie L. A controlled trial of exercise by residents of old people's homes. Age and Ageing 1993 Jan;22(1):11-15. PMID: 8438659.
863. McMurdo ME, Rennie LM. Improvements in quadriceps strength with regular seated exercise in the institutionalized elderly. Archives of Physical Medicine and Rehabilitation 1994 May;75(5):600-603. PMID: 8185457.
864. McNamara RJ, McKeough ZJ, McKenzie DK, Alison JA. Water-based exercise in COPD with physical co-morbidities: a randomised controlled trial. The European Respiratory Journal 2013 Jun;41(6):1284-1291. doi: 10.1183/09031936.00034312. PMID: 22997217.
865. McNeely ML, Parliament M, Courneya KS, Seikaly H, Jha N, Scrimger R, Hanson J. A pilot study of a randomized controlled trial to evaluate the effects of progressive resistance exercise training on shoulder dysfunction caused by spinal accessory neurapraxia/neurectomy in head and neck cancer survivors. Head & Neck 2004 Jun;26(6):518-530. doi: 10.1002/hed.20010. PMID: 15162353.
866. McNeely ML, Parliament MB, Seikaly H, Jha N, Magee DJ, Haykowsky MJ, Courneya KS. Effect of exercise on upper extremity pain and dysfunction in head and neck cancer survivors: a randomized controlled trial. Cancer 2008 Jul 1;113(1):214-222. doi: 10.1002/cncr.23536. PMID: 18457329.
867. McNeil JK, LeBlanc EM, Joyner M. The effect of exercise on depressive symptoms in the moderately depressed elderly. Psychology and Aging 1991 Sep;6(3):487-488. doi: 10.1037/0882-7974.6.3.487. PMID: 1930766.
868. Mead GE, Greig CA, Cunningham I, Lewis SJ, Dinan S, Saunders DH, Fitzsimons C, Young A. Stroke: a randomized trial of exercise or relaxation. Journal of the American Geriatrics Society 2007 Jun;55(6):892-899. doi: 10.1111/j.1532-5415.2007.01185.x. PMID: 17537090.
869. Meade TW, Dyer S, Browne W, Townsend J, Frank AO. Low back pain of mechanical origin: randomised comparison of chiropractic and hospital outpatient treatment. BMJ 1990 Jun 2;300(6737):1431-1437. PMID: 2143092.
870. Meecham Jones DJ, Paul EA, Jones PW, Wedzicha JA. Nasal pressure support ventilation plus oxygen compared with oxygen therapy alone in hypercapnic COPD. American Journal of Respiratory and Critical Care Medicine 1995 Aug;152(2):538-544. doi: 10.1164/ajrccm.152.2.7633704. PMID: 7633704.
871. Mehnert A, Veers S, Howaldt D, Braumann K-M, Koch U, Schulz K-H. Effects of a physical exercise rehabilitation group program on anxiety, depression, body image, and health-related quality of life among breast cancer patients. Onkologie 2011 May;34(5):248-253. doi: 10.1159/000327813. PMID: 21577030.
872. Mendes de Oliveira JC, Studart Leitao Filho FS, Malosa Sampaio LM, Negrinho de Oliveira AC, Hirata RP, Costa D, Donner CF, de Oliveira LVF. Outpatient versus home-based pulmonary rehabilitation in COPD: a randomized controlled trial. Multidisciplinary Respiratory Medicine 2010;5(6):401-408. doi: 10.1186/2049-6958-5-6-401. PMID: 22958267.
873. Mendes FA, Goncalves RC, Nunes MP, Saraiva-Romanholo BM, Cukier A, Stelmach R, Jacob-Filho W, Martins MA, Carvalho CR. Effects of aerobic training on psychosocial morbidity and symptoms in patients with asthma: a randomized clinical trial. Chest 2010 Aug;138(2):331-337. doi: 10.1378/chest.09-2389. PMID: 20363839.
874. Mendes FAR, Almeida FM, Cukier A, Stelmach R, Jacob-Filho W, Martins MA, Carvalho CRF. Effects of aerobic training on airway inflammation in asthmatic patients. Medicine and Science in Sports and Exercise 2011 Feb;43(2):197-203. doi: 10.1249/MSS.0b013e3181ed0ea3. PMID: 20581719.
875. Messier SP, Loeser RF, Miller GD, Morgan TM, Rejeski WJ, Sevick MA, Ettinger WH Jr, Pahor M, Williamson JD. Exercise and dietary weight loss in overweight and obese older adults with knee osteoarthritis: the arthritis, diet, and activity promotion trial. Arthritis and Rheumatism 2004 May;50(5):1501-1510. doi: 10.1002/art.20256. PMID: 15146420.
876. Metcalfe L, Lohman T, Going S, Houtkooper L, Ferriera D, Flint-Wagner H, Guido T, Martin J, Wright J, Cussler E. Postmenopausal women and exercise for prevention of osteoporosis: the Bone, Estrogen, Strength Training (BEST) study. ACSM's Health and Fitness Journal 2001 May-Jun;5(3):6-14.
877. Meuleman JR, Brechue WF, Kubilis PS, Lowenthal DT. Exercise training in the debilitated aged: strength and functional outcomes. Archives of Physical Medicine and Rehabilitation 2000 Mar;81(3):312-318. doi: 10.1016/S0003-9993(00)90077-7. PMID: 10724076.
878. Meyer K, Fransen J, Huwiler H, Uebelhart D, Klipstein A. Feasibility and results of a randomised pilot-study of a work rehabilitation programme. Journal of Back and Musculoskeletal Rehabilitation 2005;18(3-4):67-78.
879. Meyer S, Hohlfeld P, Achtari C, de Grandi P. Pelvic floor education after vaginal delivery. Obstetrics and Gynecology 2001 May;97(5 Pt 1):673-677. doi: 10.1016/S0029-7844(00)01101-7. PMID: 11339914.
880. Mihalko SL, McAuley E. Strength training effects on subjective wellbeing and physical function in the elderly. Journal of Aging and Physical Activity 1996 Jan;4(1):56-68.
881. Mika P, Spodaryk K, Cencora A, Mika A. Red blood cell deformability in patients with claudication after pain-free treadmill training. Clinical Journal of Sport Medicine 2006 Jul;16(4):335-340. PMID: 16858218.
882. Mika P, Spodaryk K, Cencora A, Unnithan VB, Mika A. Experimental model of pain-free treadmill training in patients with claudication. American Journal of Physical Medicine & Rehabilitation 2005 Oct;84(10):756-762. PMID: 16205431.
883. Mika P, Wilk B, Mika A, Marchewka A, Nizankowski R. The effect of pain-free treadmill training on fibrinogen, haematocrit, and lipid profile in patients with claudication. European Journal of Cardiovascular Prevention and Rehabilitation 2011 Oct;18(5):754-760. doi: 10.1177/1741826710389421. PMID: 21450630.
884. Mikesky AE, Mazzuca SA, Brandt KD, Perkins SM, Damush T, Lane KA. Effects of strength training on the incidence and progression of knee osteoarthritis. Arthritis Care & Research 2006 Oct 15;55(5):690-699. doi: 10.1002/art.22245. PMID: 17013851.
885. Miller L, Mattison P, Paul L, Wood L. The effects of transcutaneous electrical nerve stimulation (TENS) on spasticity in multiple sclerosis. Multiple Sclerosis 2007 May;13(4):527-533. doi: 10.1177/1352458506071509. PMID: 17463075.
886. Miller NH, Haskell WL, Berra K, de Busk RF. Home versus group exercise training for increasing functional capacity after myocardial infarction. Circulation 1984 Oct;70(4):645-649. doi: 10.1161/01.CIR.70.4.645. PMID: 6478567.
887. Milne HM, Wallman KE, Gordon S, Courneya KS. Effects of a combined aerobic and resistance exercise program in breast cancer survivors: a randomized controlled trial. Breast Cancer Research and Treatment 2008 Mar;108(2):279-288. doi: 10.1007/s10549-007-9602-z. PMID: 17530428.
888. Minor MA, Hewett JE, Webel RR, Anderson SK, Kay DR. Efficacy of physical conditioning exercise in patients with rheumatoid arthritis and osteoarthritis. Arthritis and Rheumatism 1989 Nov;32(11):1396-1405. doi: 10.1002/anr.1780321108. PMID: 2818656.
889. Mishra S, Prabhakar S, Lal V, Modi M, Das CP, Khurana D. Efficacy of splinting and oral steroids in the treatment of carpal tunnel syndrome: a prospective randomized clinical and electrophysiological study. Neurology India 2006 Jul-Sep;54(3):286-290. doi: 10.4103/0028-3886.27155. PMID: 16936391.
890. Mitchell RI, Carmen GM. The functional restoration approach to the treatment of chronic pain in patients with soft tissue and back injuries. Spine 1994 Mar 15;19(6):633-642. PMID: 8009327.
891. Moadel AB, Shah C, Wylie-Rosett J, Harris MS, Patel SR, Hall CB, Sparano JA. Randomized controlled trial of yoga among a multiethnic sample of breast cancer patients: effects on quality of life. Journal of Clinical Oncology 2007 Oct 1;25(28):4387-4395. doi: 10.1200/JCO.2006.06.6027. PMID: 17785709.
892. Mock V, Burke MB, Sheehan P, Creaton EM, Winningham ML, McKenney-Tedder S, Schwager LP, Liebman M. A nursing rehabilitation program for women with breast cancer receiving adjuvant chemotherapy. Oncology Nursing Forum 1994 Jun;21(5):899-907. PMID: 7937251.
893. Mock V, Dow KH, Meares CJ, Grimm PM, Dienemann JA, Haisfield-Wolfe ME, Quitasol W, Mitchell S, Chakravarthy A, Gage I. Effects of exercise on fatigue, physical functioning, and emotional distress during radiation therapy for breast cancer. Oncology Nursing Forum 1997 Jul;24(6):991-1000. PMID: 9243585.
894. Mock V, Frangakis C, Davidson NE, Ropka ME, Pickett M, Poniatowski B, Stewart KJ, Cameron L, Zawacki K, Podewils LJ, Cohen G, McCorkle R. Exercise manages fatigue during breast cancer treatment: a randomized controlled trial. Psycho-Oncology 2005 Jun;14(6):464-477. doi: 10.1002/pon.863. PMID: 15484202.
895. Mock V, Pickett M, Ropka ME, Muscari Lin E, Stewart KJ, Rhodes VA, McDaniel R, Grimm PM, Krumm S, McCorkle R. Fatigue and quality of life outcomes of exercise during cancer treatment. Cancer Practice 2001;9(3):119-127. doi: 10.1046/j.1523-5394.2001.009003119.x. PMID: 11879296.
896. Moholdt T, Bekken Vold M, Grimsmo J, Slordahl SA, Wisloff U. Home-based aerobic interval training improves peak oxygen uptake equal to residential cardiac rehabilitation: a randomized, controlled trial. PLoS ONE 2012 Jul;7(7):e41199. doi: 10.1371/journal.pone.0041199. PMID: 22815970.
897. Mohseni-Bandpei MA, Critchley J, Staunton T, Richardson B. A prospective randomised controlled trial of spinal manipulation and ultrasound in the treatment of chronic low back pain. Physiotherapy 2006 Mar;92(1):34-42. doi: 10.1016/j.physio.2005.05.005.
898. Molsted S, Eidemak I, Sorensen HT, Kristensen JH. Five months of physical exercise in hemodialysis patients: effects on aerobic capacity, physical function and self-rated health. Nephron 2004;96(3):c76-c81. doi: 10.1159/000076744. PMID: 15056989.
899. Monga U, Garber SL, Thornby J, Vallbona C, Kerrigan AJ, Monga TN, Zimmermann KP. Exercise prevents fatigue and improves quality of life in prostate cancer patients undergoing radiotherapy. Archives of Physical Medicine and Rehabilitation 2007 Nov;88(11):1416-1422. doi: 10.1016/j.apmr.2007.08.110. PMID: 17964881.
900. Montgomery F, Eliasson M. Continuous passive motion compared to active physical therapy after knee arthroplasty -- similar hospitalization times in a randomized study of 68 patients. Acta Orthopaedica Scandinavica 1996;67(1):7-9. PMID: 8615108.
901. Montgomery PG, Pyne DB, Hopkins WG, Dorman JC, Cook K, Minahan CL. The effect of recovery strategies on physical performance and cumulative fatigue in competitive basketball. Journal of Sports Sciences 2008;26(11):1135-1145. doi: 10.1080/02640410802104912. PMID: 18608847.
902. Moore JL, Roth EJ, Killian C, Hornby TG. Locomotor training improves daily stepping activity and gait efficiency in individuals poststroke who have reached a "plateau" in recovery. Stroke 2010 Jan;41(1):129-135. doi: 10.1161/STROKEAHA.109.563247. PMID: 19910547.
903. Moore KN, Griffiths D, Hughton A. Urinary incontinence after radical prostatectomy: a randomized controlled trial comparing pelvic muscle exercises with or without electrical stimulation. BJU International 1999 Jan;83(1):57-65. doi: 10.1046/j.1464-410x.1999.00894.x. PMID: 10233453.
904. Moore KN, Valiquette L, Chetner MP, Byrniak S, Herbison GP. Return to continence after radical retropubic prostatectomy: a randomized trial of verbal and written instructions versus therapist-directed pelvic floor muscle therapy. Urology 2008 Dec;72(6):1280-1286. doi: 10.1016/j.urology.2007.12.034. PMID: 18384853.
905. Moore W, James DK. A random trial of three topical analgesic agents in the treatment of episiotomy pain following instrumental vaginal delivery. Journal of Obstetrics and Gynaecology 1989;10(1):35-39. doi: 10.3109/01443618909151090.
906. Moraska A, Chandler C, Edmiston-Schaetzel A, Franklin G, Calenda EL, Enebo B. Comparison of a targeted and general massage protocol on strength, function, and symptoms associated with carpal tunnel syndrome: a randomized pilot study. Journal of Alternative & Complementary Medicine 2008 Apr;14(3):259-267. doi: 10.1089/acm.2007.0647. PMID: 18370581.
907. Moreira A, Delgado L, Haahtela T, Fonseca J, Moreira P, Lopes C, Mota J, Santos P, Rytila P, Castel-Branco MG. Physical training does not increase allergic inflammation in asthmatic children. The European Respiratory Journal 2008 Dec;32(6):1570-1575. doi: 10.1183/09031936.00171707. PMID: 18684843.
908. Morgan PJ, Lubans DR, Collins CE, Warren JM, Callister R. The SHED-IT randomized controlled trial: evaluation of an internet-based weight-loss program for men. Obesity 2009 Nov;17(11):2025-2032. doi: 10.1038/oby.2009.85. PMID: 19343018.
909. Mori F, Ljoka C, Magni E, Codeca C, Kusayanagi H, Monteleone F, Sancesario A, Bernardi G, Koch G, Foti C, Centonze D. Transcranial magnetic stimulation primes the effects of exercise therapy in multiple sclerosis. Journal of Neurology 2011 Jul;258(7):1281-1287. doi: 10.1007/s00415-011-5924-1. PMID: 21286740.
910. Morkved S, Bo K, Fjortoft T. Effect of adding biofeedback to pelvic floor muscle training to treat urodynamic stress incontinence. Obstetrics and Gynecology 2002;100(4):730-739. PMID: 12383542.
911. Morkved S, Bo K, Schei B, Salvesen KA. Pelvic floor muscle training during pregnancy to prevent urinary incontinence: a single-blind randomized controlled trial. Obstetrics and Gynecology 2003 Feb;101(2):313-319. doi: 10.1016/S0029-7844(02)02711-4. PMID: 12576255.
912. Morkved S, Salvesen KA, Schei B, Lydersen S, Bo K. Does group training during pregnancy prevent lumbopelvic pain? A randomized clinical trial. Acta Obstetricia et Gynecologica Scandinavica 2007 Mar;86(3):276-282. doi: 10.1080/00016340601089651. PMID: 17364300.
913. Moros MT, Ruidiaz M, Caballero A, Serrano E, Martinez V, Tres A. Ejercicio fisico en mujeres con cancer de mama (Effects of an exercise training program on the quality of life of women with breast cancer on chemotherapy) [Spanish]. Revista Medica de Chile 2010 Jun;138(6):715-722. doi: 10.4067/S0034-98872010000600008. PMID: 20919481.
914. Morris JN, Fiatarone M, Kiely DK, Belleville-Taylor P, Murphy K, Littlehale S, Ooi WL, O'Neill E, Doyle N. Nursing rehabilitation and exercise strategies in the nursing home. The Journals of Gerontology. Series A, Biological Sciences and Medical Sciences 1999 Oct;54(10):M494-M500. PMID: 10568531.
915. Moseley AM, Herbert RD, Nightingale EJ, Taylor DA, Evans TM, Robertson GJ, Gupta SK, Penn J. Passive stretching does not enhance outcomes in patients with plantarflexion contracture after cast immobilization for ankle fracture: a randomized controlled trial. Archives of Physical Medicine and Rehabilitation 2005 Jun;86(6):1118-1126. doi: 10.1016/j.apmr.2004.11.017. PMID: 15954049.
916. Moseley GL, Nicholas MK, Hodges PW. A randomized controlled trial of intensive neurophysiology education in chronic low back pain. The Clinical Journal of Pain 2004 Sep-Oct;20(5):324-330. PMID: 15322439.
917. Mosforth J, Taverner D. Physiotherapy for Bell's palsy. British Medical Journal 1958 Sep 13;2(5097):675-677. PMID: 13572865.
918. Mota-Pereira J, Silverio J, Carvalho S, Ribeiro JC, Fonte D, Ramos J. Moderate exercise improves depression parameters in treatment-resistant patients with major depressive disorder. Journal of Psychiatric Research 2011 Aug;45(8):1005-1011. doi: 10.1016/j.jpsychires.2011.02.005. PMID: 21377690.
919. Moul JL, Goldman B, Warren B. Physical activity and cognitive performance in the older population. Journal of Aging and Physical Activity 1995 Apr;3(2):135-145.
920. Moyer-Mileur LJ, Ransdell L, Bruggers CS. Fitness of children with standard-risk acute lymphoblastic leukemia during maintenance therapy: response to a home-based exercise and nutrition program. Journal of Pediatric Hematology/Oncology 2009 Apr;31(4):259-266. doi: 10.1097/MPH.0b013e3181978fd4. PMID: 19346877.
921. Mudge S, Barber PA, Stott NS. Circuit-based rehabilitation improves gait endurance but not usual walking activity in chronic stroke: a randomized controlled trial. Archives of Physical Medicine and Rehabilitation 2009 Dec;90(12):1989-1996. doi: 10.1016/j.apmr.2009.07.015. PMID: 19969159.
922. Mueller G, Hopman MTE, Perret C. Comparison of respiratory muscle training methods in individuals with motor and sensory complete tetraplegia: a randomized controlled trial. Journal of Rehabilitation Medicine 2013 Mar;45(3):248-253. doi: 10.2340/16501977-1097. PMID: 23389554.
923. Muller K, Schwesig R, Leuchte S, Riede D. Koordinationstraining und lebensqualitat -- eine langsschnittuntersuchung bei pflegepersonal mit ruckenschmerzen (Coordinative treatment and quality of life -- a randomised trial of nurses with back pain) [German]. Das Gesundheitswesen 2001;63(10):609-618. doi: 10.1055/s-2001-17872. PMID: 11607869.
924. Muller R, Giles LG. Long-term follow-up of a randomized clinical trial assessing the efficacy of medication, acupuncture, and spinal manipulation for chronic mechanical spinal pain syndromes. Journal of Manipulative and Physiological Therapeutics 2005 Jan;28(1):3-11. doi: 10.1016/j.jmpt.2004.12.004. PMID: 15726029.
925. Mulrow CD, Gerety MB, Kanten D, Cornell JE, de Nino LA, Chiodo L, Aguilar C, O'Neil MB, Rosenberg J, Solis RM. A randomized trial of physical rehabilitation for very frail nursing home residents. JAMA 1994 Feb 16;271(7):519-524. doi: 10.1001/jama.1994.03510310049037. PMID: 8301766.
926. Murphy SL, Strasburg DM, Lyden AK, Smith DM, Koliba JF, Dadabhoy DP, Wallis SM. Effects of activity strategy training on pain and physical activity in older adults with knee or hip osteoarthritis: a pilot study. Arthritis Care & Research 2008 Oct 15;59(10):1480-1487. doi: 10.1002/art.24105. PMID: 18821646.
927. Murtezani A, Pacarada M, Ibraimi Z, Nevzati A, Abazi N. The impact of exercise during pregnancy on neonatal outcomes: a randomized controlled trial. The Journal of Sports Medicine and Physical Fitness 2014 Dec;54(6):802-808. PMID: 25350037.
928. Musanti R. A study of exercise modality and physical self-esteem in breast cancer survivors. Medicine and Science in Sports and Exercise 2012 Feb;44(2):352-361. doi: 10.1249/MSS.0b013e31822cb5f2. PMID: 21796050.
929. Mustian KM, Katula JA, Gill DL, Roscoe JA, Lang D, Murphy K. Tai Chi Chuan, health-related quality of life and self-esteem: a randomized trial with breast cancer survivors. Supportive Care in Cancer 2004;12(12):871-876. doi: 10.1007/s00520-004-0682-6. PMID: 15599776.
930. Mustian KM, Peppone L, Darling TV, Palesh O, Heckler CE, Morrow GR. A 4-week home-based aerobic and resistance exercise program during radiation therapy: a pilot randomized clinical trial. The Journal of Supportive Oncology 2009 Sep-Oct;7(5):158-167. PMID: 19831159.
931. Mutrie N, Campbell AM, Whyte F, McConnachie A, Emslie C, Lee L, Kearney N, Walker A, Ritchie D. Benefits of supervised group exercise programme for women being treated for early stage breast cancer: pragmatic randomised controlled trial. BMJ 2007 Mar 10;334(7592):517-523. doi: 10.1136/bmj.39094.648553.AE. PMID: 17307761.
932. Nabkasorn C, Miyai N, Sootmongkol A, Junprasert S, Yamamoto H, Arita M, Miyashita K. Effects of physical exercise on depression, neuroendocrine stress hormones and physiological fitness in adolescent females with depressive symptoms. European Journal of Public Health 2006 Apr;16(2):179-184. doi: 10.1093/eurpub/cki159. PMID: 16126743.
933. Nagarathna R, Nagendra HR. Yoga for bronchial asthma: a controlled study. British Medical Journal 1985 Oct 19;291(6502):1077-1079. PMID: 3931802.
934. Nascimento S, Surita F, Parpinelli M, Siani S, Pinto E Silva J. The effect of an antenatal physical exercise programme on maternal/perinatal outcomes and quality of life in overweight and obese pregnant women: a randomised clinical trial. BJOG 2011 Nov;118(12):1455-1463. doi: 10.1111/j.1471-0528.2011.03084.x. PMID: 21895947.
935. Nasis IG, Vogiatzis I, Stratakos G, Athanasopoulos D, Koutsoukou A, Daskalakis A, Spetsioti S, Evangelodimou A, Roussos C, Zakynthinos S. Effects of interval-load versus constant-load training on the BODE index in COPD patients. Respiratory Medicine 2009 Sep;103(9):1392-1398. doi: 10.1016/j.rmed.2009.03.003. PMID: 19349153.
936. Naso F, Carner E, Blankfort-Doyle W, Coughey K. Endurance training in the elderly nursing home patient. Archives of Physical Medicine and Rehabilitation 1990 Mar;71(3):241-243. PMID: 2317144.
937. Navabi S, Abedian Z, Steen-Greaves M. Effectivness of cooling gel pads and ice packs on perineal pain. British Journal of Midwifery 2009 Nov;17(11):724,726-729.
938. Nedstrand E, Wyon Y, Hammar M, Wijma K. Psychological well-being improves in women with breast cancer after treatment with applied relaxation or electro-acupuncture for vasomotor symptom. Journal of Psychosomatic Obstetrics and Gynecology 2006 May;27(4):193-199. doi: 10.1080/01674820600724797. PMID: 17225620.
939. Nelson ME, Fiatarone MA, Morganti CM, Trice I, Greenberg RA, Evans WJ. Effects of high-intensity strength training on multiple risk factors for osteoporotic fractures. A randomized controlled trial. JAMA 1994 Dec 28;272(24):1909-1914. doi: 10.1001/jama.1994.03520240037038. PMID: 7990242.
940. Newsome RJ, May S, Chiverton N, Cole AA. A prospective, randomised trial of immediate exercise following lumbar microdiscectomy: a preliminary study. Physiotherapy 2009 Dec;95(4):273-279. doi: 10.1016/j.physio.2009.06.004. PMID: 19892091.
941. Newstead A, Smith KI, Bruder J, Keller C. The effect of a jumping exercise intervention on bone mineral density in postmenopausal women. Journal of Geriatric Physical Therapy 2004;27(2):47-52.
942. Newton-John TR, Spence SH, Schotte D. Cognitive-behavioural therapy versus EMG biofeedback in the treatment of chronic low back pain. Behaviour Research and Therapy 1995 Jul;33(6):691-697. doi: 10.1016/0005-7967(95)00008-L. PMID: 7654161.
943. Ng S-C, Lin T-L, Chang S-J, Tai H-L, Hu S-W, Chen G-D. Nursing intervention to enhance efficacy of home practice of pelvic floor muscle exercises in treating mixed urinary incontinence. International Urogynecology Journal and Pelvic Floor Dysfunction 2008 May;19(5):637-642. doi: 10.1007/s00192-007-0492-8. PMID: 18004495.
944. Ng TS, Yeo SJ. An alternative early knee flexion regimen of continuous passive motion for total knee arthroplasty. Physiotherapy Singapore 1999;2(2):53-63.
945. Nicholas MK, Wilson PH, Goyen J. Operant-behavioural and cognitive-behavioural treatment for chronic low back pain. Behaviour Research and Therapy 1991;29(3):225-238. doi: 10.1016/0005-7967(91)90112-G. PMID: 1831972.
946. Nicholas MK, Wilson PH, Goyen J. Comparison of cognitive-behavioral group treatment and an alternative non-psychological treatment for chronic low back pain. Pain 1992 Mar;48(3):339-347. PMID: 1534400.
947. Nicolai SPA, Hendriks EJM, Prins MH, Teijink JAW, EXITPAD Study Group. Optimizing supervised exercise therapy for patients with intermittent claudication. Journal of Vascular Surgery 2010 Nov;52(5):1226-1233. doi: 10.1016/j.jvs.2010.06.106. PMID: 20692797.
948. Nield MA, Soo Hoo GW, Roper JM, Santiago S. Efficacy of pursed-lips breathing: a breathing pattern retraining strategy for dyspnea reduction. Journal of Cardiopulmonary Rehabilitation and Prevention 2007 Jul-Aug;27(4):237-244. doi: 10.1097/01.HCR.0000281770.82652.cb. PMID: 17667021.
949. Nielsen JF, Sinkjaer T, Jakobsen J. Treatment of spasticity with repetitive magnetic stimulation; a double-blind placebo-controlled study. Multiple Sclerosis 1996 Dec;2(5):227-232. PMID: 9050361.
950. Nielsen PT, Rechnagel K, Nielsen SE. No effect of continuous passive motion after arthroplasty of the knee. Acta Orthopaedica Scandinavica 1988 Oct;59(5):580-581. PMID: 3188866.
951. Nilsson GM, Jonsson K, Ekdahl CS, Eneroth M. Effects of a training program after surgically treated ankle fracture: a prospective randomised controlled trial. BMC Musculoskeletal Disorders 2009 Sep 25;10(118):Epub. doi: 10.1186/1471-2474-10-118. PMID: 19781053.
952. Nilsson N, Christensen HW, Hartvigsen J. The effect of spinal manipulation in the treatment of cervicogenic headache. Journal of Manipulative and Physiological Therapeutics 1997 Jun;20(5):326-330. PMID: 9200048.
953. Nir Y, Huang MI, Schnyer R, Chen B, Manber R. Acupuncture for postmenopausal hot flashes. Maturitas 2007 Apr 20;56(4):383-395. doi: 10.1016/j.maturitas.2006.11.001. PMID: 17182200.
954. Nordemar R, Thorner C. Treatment of acute cervical pain -- a comparative group study. Pain 1981 Feb;10(1):93-101. PMID: 6972028.
955. Norregaard O, Jakobsen J, Nielsen KK. Hyperextension injuries of the PIP finger joint. Comparison of early motion and immobilization. Acta Orthopaedica Scandinavica 1987 Jun;58(3):239-240. PMID: 3307283.
956. Noseda A, Carpiaux JP, Vandeput W, Prigogine T, Schmerber J. Resistive inspiratory muscle training and exercise performance in COPD patients. A comparative study with conventional breathing retraining. Bulletin Europeen de Physiopathologie Respiratoire 1987 Sep-Oct;23(5):457-463. PMID: 3450328.
957. Nouwen A. EMG biofeedback used to reduce standing levels of paraspinal muscle tension in chronic low back pain. Pain 1983 Dec;17(4):353-360. PMID: 6229707.
958. Nunez M, Nunez E, Segur JM, Macule F, Quinto L, Hernandez MV, Vilalta C. The effect of an educational program to improve health-related quality of life in patients with osteoarthritis on waiting list for total knee replacement: a randomized study. Osteoarthritis and Cartilage 2006 Mar;14(3):279-285. doi: 10.1016/j.joca.2005.10.002. PMID: 16309929.
959. Nyanzi CS, Langridge J, Heyworth JRC, Mani R. Randomized controlled study of ultrasound therapy in the management of acute lateral ligament sprains of the ankle joint. Clinical Rehabilitation 1999 Feb;13(1):16-22. doi: 10.1191/026921599701532081. PMID: 10327093.
960. Oakland C. A comparison of the efficacy of the topical NSAID felbinac and ultrasound in the treatment of acute ankle injuries. British Journal of Clinical Research 1993;4:89-96.
961. O'Connor M, Tattersall MP, Carter JA. An evaluation of the incentive spirometer to improve lung function after cholecystectomy. Anaesthesia 1988 Sep;43(9):785-787. doi: 10.1111/j.1365-2044.1988.tb05759.x. PMID: 3177858.
962. O'Dowd H, Gladwell P, Rogers CA, Hollinghurst S, Gregory A. Cognitive behavioural therapy in chronic fatigue syndrome: a randomised controlled trial of an outpatient group programme. Health Technology Assessment (Winchester, England) 2006 Oct;10(37):1-121. doi: 10.3310/hta10370. PMID: 17014748.
963. Oerkild B, Frederiksen M, Hansen JF, Simonsen L, Skovgaard LT, Prescott E. Home-based cardiac rehabilitation is as effective as centre-based cardiac rehabilitation among elderly with coronary heart disease: results from a randomised clinical trial. Age and Ageing 2011 Jan;40(1):78-85. doi: 10.1093/ageing/afq122. PMID: 20846961.
964. Oh B, Butow P, Mullan B, Clarke S. Medical Qigong for cancer patients: pilot study of impact on quality of life, side effects of treatment and inflammation. The American Journal of Chinese Medicine 2008;36(3):459-472. doi: 10.1142/S0192415X08005904. PMID: 18543381.
965. Oh B, Butow P, Mullan B, Clarke S, Beale P, Pavlakis N, Kothe E, Lam L, Rosenthal D. Impact of medical Qigong on quality of life, fatigue, mood and inflammation in cancer patients: a randomized controlled trial. Annals of Oncology 2010 Mar;21(3):608-614. doi: 10.1093/annonc/mdp479. PMID: 19880433.
966. Ohira T, Schmitz KH, Ahmed RL, Yee D. Effects of weight training on quality of life in recent breast cancer survivors: the Weight Training for Breast Cancer Survivors (WTBS) study. Cancer 2006 May 1;106(9):2076-2083. doi: 10.1002/cncr.21829. PMID: 16568409.
967. Oken BS, Zajdel D, Kishiyama S, Flegal K, Dehen C, Haas M, Kraemer DF, Lawrence J, Leyva J. Randomized, controlled, six-month trial of yoga in healthy seniors: effects on cognition and quality of life. Alternative Therapies in Health and Medicine 2006 Jan-Feb;12(1):40-47. PMID: 16454146.
968. Olah KS, Bridges N, Denning J, Farrar DJ. The conservative management of patients with symptoms of stress incontinence: a randomized, prospective study comparing weighted vaginal cones and interferential therapy. American Journal of Obstetrics and Gynecology 1990 Jan;162(1):87-92. PMID: 2301521.
969. Oldham JA, Howe TE, Petterson T, Smith GP, Tallis RC. Electrotherapeutic rehabilitation of the quadriceps in elderly osteoarthritic patients: a double blind assessment of patterned neuromuscular stimulation. Clinical Rehabilitation 1995 Feb;9(1):10-20. doi: 10.1177/026921559500900102.
970. Oldroyd JC, Unwin NC, White M, Mathers JC, Alberti KG. Randomised controlled trial evaluating lifestyle interventions in people with impaired glucose tolerance. Diabetes Research and Clinical Practice 2006 May;72(2):117-127. doi: 10.1016/j.diabres.2005.09.018. PMID: 16297488.
971. Oliveira MMF, Souza GA, Miranda MS, Okubo MAP, Amaral MT, Silva MPP, Gurgel MSC. Exercicios para membros superiores durante radioterapia para cancer de mama e qualidade de vida (Upper limbs exercises during radiotherapy for breast cancer and quality of life) [Portuguese]. Revista Brasileira de Ginecologia e Obstetricia 2010 Mar;32(3):133-138. doi: 10.1590/S0100-72032010000300006. PMID: 20512260.
972. Oostdam N, van Poppel MNM, Wouters MGAJ, Eekhoff EMW, Bekedam DJ, Kuchenbecker WKH, Quartero HWP, Heres MHB, van Mechelen W. No effect of the FitFor2 exercise programme on blood glucose, insulin sensitivity, and birthweight in pregnant women who were overweight and at risk for gestational diabetes: results of a randomised controlled trial. BJOG 2012 Aug;119(9):1098-1107. doi: 10.1111/j.1471-0528.2012.03366.x. PMID: 22616913.
973. O'Reilly SC, Muir KR, Doherty M. Effectiveness of home exercise on pain and disability from osteoarthritis of the knee: a randomised controlled trial. Annals of the Rheumatic Diseases 1999 Jan;58(1):15-19. doi: 10.1136/ard.58.1.15. PMID: 10343535.
974. Ostbye T, Krause KM, Lovelady CA, Morey MC, Bastian LA, Peterson BL, Swamy GK, Brouwer RJ, McBride CM. Active Mothers Postpartum: a randomized controlled weight-loss intervention trial. American Journal of Preventive Medicine 2009 Sep;37(3):173-180. doi: 10.1016/j.amepre.2009.05.016. PMID: 19595557.
975. Ostelo RW, de Vet HC, Berfelo MW, Kerckhoffs MR, Vlaeyen JW, Wolters PM, van den Brandt PA. Effectiveness of behavioral graded activity after first-time lumbar disc surgery: short term results of a randomized controlled trial. European Spine Journal 2003 Dec;12(6):637-644. doi: 10.1007/s00586-003-0560-9. PMID: 14505118.
976. O'Toole ML, Sawicki MA, Artal R. Structured diet and physical activity prevent postpartum weight retention. Journal of Women's Health 2003 Dec;12(10):991-998. doi: 10.1089/154099903322643910. PMID: 14709187.
977. Ouellette MM, LeBrasseur NK, Bean JF, Phillips E, Stein J, Frontera WR, Fielding RA. High-intensity resistance training improves muscle strength, self-reported function, and disability in long-term stroke survivors. Stroke 2004 Jun;35(6):1404-1409. doi: 10.1161/01.STR.0000127785.73065.34. PMID: 15105515.
978. Ouslander JG, Griffiths P, McConnell E, Riolo L, Schnelle J. Functional incidental training: applicability and feasibility in the veterans affairs nursing home patient population. Journal of the American Medical Directors Association 2005 Mar-Apr;6(2):121-127. doi: 10.1016/j.jamda.2005.01.004. PMID: 15871887.
979. Ouzouni S, Kouidi E, Sioulis A, Grekas D, Deligiannis A. Effects of intradialytic exercise training on health-related quality of life indices in haemodialysis patients. Clinical Rehabilitation 2009 Jan;23(1):53-63. doi: 10.1177/0269215508096760. PMID: 19114437.
980. Overgard M, Angelsen A, Lydersen S, Morkved S. Does physiotherapist-guided pelvic floor muscle training reduce urinary incontinence after radical prostatectomy? A randomised controlled trial. European Urology 2008 Aug;54(2):438-448. doi: 10.1016/j.eururo.2008.04.021. PMID: 18448233.
981. Ozdemir EP, Solak O, Fidan F, Demirdal US, Evcik D, Unlu M, Kavuncu V. The effect of water-based pulmonary rehabilitation on anxiety and quality of life in chronic pulmonary obstructive disease patients. Turkiye Klinikleri Tip Bilimleri Dergisi [Turkish Journal of Medical Science] 2010;30(3):880-887.
982. Oztas O, Turan B, Bora I, Karakaya MK. Ultrasound therapy effect in carpal tunnel syndrome. Archives of Physical Medicine and Rehabilitation 1998 Dec;79(12):1540-1544. doi: 10.1016/S0003-9993(98)90416-6. PMID: 9862296.
983. Ozturk B, Gunduz OH, Ozoran K, Bostanoglu S. Effect of continuous lumbar traction on the size of herniated disc material in lumbar disc herniation. Rheumatology International 2006 May;26(7):622-626. doi: 10.1007/s00296-005-0035-x. PMID: 16249899.
984. Paatelma M, Kilpikoski S, Simonen R, Heinonen A, Alen M, Videman T. Orthopaedic manual therapy, McKenzie method or advice only for low back pain in working adults: a randomized controlled trial with one year follow-up. Journal of Rehabilitation Medicine 2008 Nov;40(10):858-863. doi: 10.2340/16501977-0262. PMID: 19242624.
985. Pages IH, Jahr S, Schaufele MK, Conradi E. Comparative analysis of biofeedback and physical therapy for treatment of urinary stress incontinence in women. American Journal of Physical Medicine & Rehabilitation 2001 Jul;80(7):494-502. PMID: 11421517.
986. Painovich JM, Shufelt CL, Azziz R, Yang Y, Goodarzi MO, Braunstein GD, Karlan BY, Stewart PM, Merz CNB. A pilot randomized, single-blind, placebo-controlled trial of traditional acupuncture for vasomotor symptoms and mechanistic pathways of menopause. Menopause 2012 Jan;19(1):54-61. doi: 10.1097/gme.0b013e31821f9171. PMID: 21968279.
987. Painter P, Moore G, Carlson L, Paul S, Myll J, Phillips W, Haskell W. Effects of exercise training plus normalization of hematocrit on exercise capacity and health-related quality of life. American Journal of Kidney Diseases 2002 Feb;39(2):257-265. doi: 10.1053/ajkd.2002.30544. PMID: 11840365.
988. Painter PL, Hector L, Ray K, Lynes L, Dibble S, Paul SM, Tomlanovich SL, Ascher NL. A randomized trial of exercise training after renal transplantation. Transplantation 2002;74(1):42-48. PMID: 12134097.
989. Painter PL, Hector L, Ray K, Lynes L, Paul SM, Dodd M, Tomlanovich SL, Ascher NL. Effects of exercise training on coronary heart disease risk factors in renal transplant recipients. American Journal of Kidney Diseases 2003 Aug;42(2):362-369. doi: 10.1016/S0272-6386(03)00673-5. PMID: 12900820.
990. Pal B, Mangion P, Hossain MA, Diffey BL. A controlled trial of continuous lumbar traction in the treatment of back pain and sciatica. British Journal of Rheumatology 1986 May;25(2):181-183. doi: 10.1093/rheumatology/25.2.181. PMID: 3011174.
991. Pan L. (Acupuncture plus short wave for 38 peripheral facial paralysis) [Chinese - simplified characters]. Zhenjiu Linchuan Zazhi [Journal of Clinical Acupuncture & Moxibustion] 2004 Apr;20(4):26-27.
992. Pan XR, Li GW, Hu YH, Wang JX, Yang WY, An ZX, Hu ZX, Lin J, Xiao JZ, Cao HB, Liu PA, Jiang XG, Jiang YY, Wang JP, Zheng H, Zhang H, Bennett PH, Howard BV. Effects of diet and exercise in preventing NIDDM in people with impaired glucose tolerance. The Da Qing IGT and Diabetes Study. Diabetes Care 1997 Apr;20(4):537-544. doi: 10.2337/diacare.20.4.537. PMID: 9096977.
993. Panton LB, Graves JE, Pollock ML, Hagberg JM, Chen W. Effect of aerobic and resistance training on fractionated reaction time and speed of movement. Journal of Gerontology 1990 Jan;45(1):M26-M31. doi: 10.1093/geronj/45.1.M2. PMID: 2295775.
994. Papaioannou A, Adachi JD, Winegard K, Ferko N, Parkinson W, Cook RJ, Webber C, McCartney N. Efficacy of home-based exercise for improving quality of life among elderly women with symptomatic osteoporosis-related vertebral fractures. Osteoporosis International 2003 Aug;14(8):677-682. doi: 10.1007/s00198-003-1423-2. PMID: 12879220.
995. Parekh AR, Feng MI, Kirages D, Bremner H, Kaswick J, Aboseif S. The role of pelvic floor exercises on post-prostatectomy incontinence. The Journal of Urology 2003 Jul;170(1):130-133. doi: 10.1097/01.ju.0000072900.82131.6f. PMID: 12796664.
996. Park H-J, Oh D-W, Kim S-Y, Choi J-D. Effectiveness of community-based ambulation training for walking function of post-stroke hemiparesis: a randomized controlled pilot trial. Clinical Rehabilitation 2011 May;25(5):451-459. doi: 10.1177/0269215510389200. PMID: 21245205.
997. Park J-E, Lee MS, Jung S, Kim A, Kang K, Choi J, Park J, Choi S-M. Moxibustion for treating menopausal hot flashes: a randomized clinical trial. Menopause 2009 Jul-Aug;16(4):660-665. doi: 10.1097/gme.0b013e318198cdf7. PMID: 19293729.
998. Park S-W, Kim TN, Nam J-K, Ha HK, Shin DG, Lee W, Kim M-S, Chung MK. Recovery of overall exercise ability, quality of life, and continence after 12-week combined exercise intervention in elderly patients who underwent radical prostatectomy: a randomized controlled study. Urology 2012 Aug;80(2):299-306. doi: 10.1016/j.urology.2011.12.060. PMID: 22749425.
999. Parkin-Smith GF, Penter CS. A clinical trial investigating the effect of two manipulative approaches in the treatment of mechanical neck pain: a pilot study. Journal of the Neuromusculoskeletal System 1998 Spring;6(1):6-16.
1000. Parr BM, Noakes TD, Derman EW. Peripheral arterial disease and intermittent claudication: efficacy of short-term upper body strength training, dynamic exercise training, and advice to exercise at home. Suid-Afrikaanse Tydskrif vir Geneeskunde [South African Medical Journal] 2009 Nov;99(11):800-804. PMID: 20218480.
1001. Parsons TL, Toffelmire EB, King-van Vlack CE. The effect of an exercise program during hemodialysis on dialysis efficacy, blood pressure and quality of life in end-stage renal disease (ESRD) patients. Clinical Nephrology 2004 Apr;61(4):261-274. PMID: 15125032.
1002. Patterson RB, Pinto B, Marcus B, Colucci A, Braun T, Roberts M. Value of a supervised exercise program for the therapy of arterial claudication. Journal of Vascular Surgery 1997 Feb;25(2):312-319. PMID: 9052565.
1003. Payne JK, Held J, Thorpe J, Shaw H. Effect of exercise on biomarkers, fatigue, sleep disturbances, and depressive symptoms in older women with breast cancer receiving hormonal therapy. Oncology Nursing Forum 2008 Jul;35(4):635-642. doi: 10.1188/08.ONF.635-642. PMID: 18591167.
1004. Peloquin L, Bravo G, Gauthier P, Lacombe G, Billiard J-S. Effects of a cross-training exercise program in persons with osteoarthritis of the knee. A randomized controlled trial. Journal of Clinical Rheumatology 1999 Jun;5(3):126-136. PMID: 19078371.
1005. Penttinen HM, Saarto T, Kellokumpu-Lehtinen P, Blomqvist C, Huovinen R, Kautiainen H, Jarvenpaa S, Nikander R, Idman I, Luoto R, Sievanen H, Utriainen M, Vehmanen L, Jaaskelainen AS, Elme A, Ruohola J, Luoma M, Hakamies-Blomqvist L. Quality of life and physical performance and activity of breast cancer patients after adjuvant treatments. Psycho-Oncology 2011 Nov;20(11):1211-1220. doi: 10.1002/pon.1837. PMID: 20878646.
1006. Pereira VS, de Melo MV, Correia GN, Driusso P. Vaginal cone for postmenopausal women with stress urinary incontinence: randomized, controlled trial. Climacteric 2012 Feb;15(1):45-51. doi: 10.3109/13697137.2011.593211. PMID: 22066898.
1007. Peri K, Kerse N, Robinson E, Parsons M, Parsons J, Latham N. Does functionally based activity make a difference to health status and mobility? A randomised controlled trial in residential care facilities (The Promoting Independent Living Study; PILS). Age and Ageing 2008 Jan;37(1):57-63. doi: 10.1093/ageing/afm135. PMID: 17965045.
1008. Perna FM, Craft L, Freund KM, Skrinar G, Stone M, Kachnic L, Youren C, Battaglia T. The effect of a cognitive behavioral exercise intervention on clinical depression in a multiethnic sample of women with breast cancer: a randomized controlled trial. International Journal of Sport and Exercise Psychology 2010;8(1):36-47. doi: 10.1080/1612197X.2010.9671932.
1009. Peterson CD, Haas M, Gregory T. A pilot randomized controlled trial comparing the efficacy of exercise, spinal manipulation, and neuro emotional technique for the treatment of pregnancy-related low back pain. Chiropractic & Manual Therapies 2012 Jun 13;20(18):Epub. doi: 10.1186/2045-709X-20-18. PMID: 22694756.
1010. Petrella E, Malavolti M, Bertarini V, Pignatti L, Neri I, Battistini NC, Facchinetti F. Gestational weight gain in overweight and obese women enrolled in a healthy lifestyle and eating habits program. The Journal of Maternal-Fetal & Neonatal Medicine 2014 Sep;27(13):1348-1352. doi: 10.3109/14767058.2013.858318. PMID: 24175912.
1011. Petrov Fieril K, Glantz A, Fagevik Olsen M. The efficacy of moderate-to-vigorous resistance exercise during pregnancy: a randomized controlled trial. Acta Obstetricia et Gynecologica Scandinavica 2015 Jan;94(1):35-42. doi: 10.1111/aogs.12525. PMID: 25287282.
1012. Phelan S, Phipps MG, Abrams B, Darroch F, Schaffner A, Wing RR. Randomized trial of a behavioral intervention to prevent excessive gestational weight gain: the Fit for Delivery Study. The American Journal of Clinical Nutrition 2011 Apr;93(4):772-779. doi: 10.3945/ajcn.110.005306. PMID: 21310836.
1013. Philipson T, Haagensen N, Laumann V, Nies M, Thorup K, Hansen TI. Effekten af diadynamisk strom pa kroniske bloddelssmerter i nakke-skulderaget (The effect of diadynamic current on chronic soft-tissue pain in the neck and shoulder girdle) [Danish]. Ugeskrift for Laeger 1983 Feb 14;145(7):479-481. PMID: 6342247.
1014. Pieber D, Zivkovic F, Tamussino K, Ralph G, Lippitt G, Fauland B. Pelvic floor exercise alone or with vaginal cones for the treatment of mild to moderate stress urinary incontinence in premenopausal women. International Urogynecology Journal and Pelvic Floor Dysfunction 1995 Feb;6(1):14-17. doi: 10.1007/BF01961842.
1015. Pilu A, Sorba M, Hardoy MC, Floris AL, Mannu F, Seruis ML, Velluti C, Carpiniello B, Salvi M, Carta MG. Efficacy of physical activity in the adjunctive treatment of major depressive disorders: preliminary results. Clinical Practice and Epidemiology in Mental Health 2007 Jul 9;3(8):Epub. doi: 10.1186/1745-0179-3-8. PMID: 17620123.
1016. Pinar L, Enhos A, Ada S, Gungor N. Can we use nerve gliding exercises in women with carpal tunnel syndrome?. Advances in Therapy 2005 Sep-Oct;22(5):467-475. doi: 10.1007/BF02849867. PMID: 16418156.
1017. Pinchasov B, Shurgaja AM, Grischin OV, Putilov AA. Mood and energy regulation in seasonal and non-seasonal depression before and after midday treatment with physical exercise or bright light. Psychiatry Research 2000 Apr 24;94(1):29-42. doi: 10.1016/S0165-1781(00)00138-4. PMID: 10788675.
1018. Pinto BM, Clark MM, Maruyama NC, Feder SI. Psychological and fitness changes associated with exercise participation among women with breast cancer. Psycho-Oncology 2003 Mar;12(2):118-126. doi: 10.1002/pon.618. PMID: 12619144.
1019. Pinto BM, Frierson GM, Rabin C, Trunzo JJ, Marcus BH. Home-based physical activity intervention for breast cancer patients. Journal of Clinical Oncology 2005 May 20;23(15):3577-3587. doi: 10.1200/JCO.2005.03.080. PMID: 15908668.
1020. Pinto BM, Papandonatos GD, Goldstein MG, Marcus BH, Farrell N. Home-based physical activity intervention for colorectal cancer survivors. Psycho-Oncology 2013 Jan;22(1):54-64. doi: 10.1002/pon.2047. PMID: 21905158.
1021. Pinzon DC, Zamora K, Martinez JH, Florez-Lopez ME, de Plata ACA, Mosquera M, Ramirez-Velez R. Type of delivery and gestational age is not affected by pregnant Latin-American women engaging in vigorous exercise: a secondary analysis of data from a controlled randomized trial. Revista de Salud Publica 2012 Oct;14(5):731-743. PMID: 24652353.
1022. Piotrowicz E, Baranowski R, Bilinska M, Stepnowska M, Piotrowska M, Wojcik A, Korewicki J, Chojnowska L, Malek LA, Klopotowski M, Piotrowski W, Piotrowicz R. A new model of home-based telemonitored cardiac rehabilitation in patients with heart failure: effectiveness, quality of life, and adherence. European Journal of Heart Failure 2010 Feb;12(2):164-171. doi: 10.1093/eurjhf/hfp181. PMID: 20042423.
1023. Piravej K, Boonhong J. Effect of ultrasound thermotherapy in mild to moderate carpal tunnel syndrome. Chotmaihet Thangphaet [Journal of the Medical Association of Thailand] 2004 Sep;87(Suppl 2):S100-S106. PMID: 16083171.
1024. Piya-Anant M, Therasakvichya S, Leelaphatanadit C, Techatrisak K. Integrated health research program for the Thai elderly: prevalence of genital prolapse and effectiveness of pelvic floor exercise to prevent worsening of genital prolapse in elderly women. Chotmaihet Thangphaet [Journal of the Medical Association of Thailand] 2003;86(6):509-515. PMID: 12924798.
1025. Pohl M, Mehrholz J, Ritschel C, Ruckriem S. Speed-dependent treadmill training in ambulatory hemiparetic stroke patients: a randomized controlled trial. Stroke 2002 Feb;33(2):553-558. PMID: 11823669.
1026. Pollak KI, Alexander SC, Bennett G, Lyna P, Coffman CJ, Bilheimer A, Farrell D, Bodner ME, Swamy GK, Ostbye T. Weight-related SMS texts promoting appropriate pregnancy weight gain: a pilot study. Patient Education and Counseling 2014 Nov;97(2):256-260. doi: 10.1016/j.pec.2014.07.030. PMID: 25153313.
1027. Polley BA, Wing RR, Sims CJ. Randomized controlled trial to prevent excessive weight gain in pregnant women. International Journal of Obesity 2002 Nov;26(11):1494-1502. doi: 10.1038/sj.ijo.0802130. PMID: 12439652.
1028. Pomerance J, Fine I. Outcomes of carpal tunnel surgery with and without supervised postoperative therapy. The Journal of Hand Surgery - American Volume 2007 Oct;32(8):1159-1163. doi: 10.1016/j.jhsa.2007.05.001. PMID: 17923296.
1029. Pomeroy VM. The effect of physiotherapy input on mobility skills of elderly people with severe dementing illness. Clinical Rehabilitation 1993 May;7(2):163-170. doi: 10.1177/026921559300700212.
1030. Poole H, Glenn S, Murphy P. A randomised controlled study of reflexology for the management of chronic low back pain. European Journal of Pain 2007 Nov;11(8):878-887. doi: 10.1016/j.ejpain.2007.01.006. PMID: 17459741.
1031. Poole JL, Whitney SL, Hangeland N, Baker C. The effectiveness of inflatable pressure splints on motor function in stroke patients. Occupational Therapy Journal of Research 1990;10(6):360-366. doi: 10.1177/153944929001000605.
1032. Pope MH, Phillips RB, Haugh LD, Hsieh CY, MacDonald L, Haldeman S. A prospective randomized three-week trial of spinal manipulation, transcutaneous muscle stimulation, massage and corset in the treatment of subacute low back pain. Spine 1994 Nov 15;19(22):2571-2577. PMID: 7855683.
1033. Pope R, Herbert R, Kirwan J. Effects of ankle dorsiflexion range and pre-exercise calf muscle stretching on injury risk in Army recruits. Australian Journal of Physiotherapy 1998;44(3):165-172. doi: 10.1016/S0004-9514(14)60376-7. PMID: 11676730.
1034. Pope RP, Herbert RD, Kirwan JD, Graham BJ. A randomized trial of preexercise stretching for prevention of lower-limb injury. Medicine and Science in Sports and Exercise 2000 Feb;32(2):271-277. PMID: 10694106.
1035. Porru D, Campus G, Caria A, Madeddu G, Cucchi A, Rovereto B, Scarpa RM, Pili P, Usai E. Impact of early pelvic floor rehabilitation after transurethral resection of the prostate. Neurourology and Urodynamics 2001;20(1):53-59. doi: 10.1002/1520-6777(2001)20:1<53::AID-NAU7>3.0.CO;2-B. PMID: 11135382.
1036. Postacchini F, Facchini M, Palieri P. Efficacy of various forms of conservative treatment in low back pain. A comparative study. Neuro-Orthopedics 1988;6(1):28-35.
1037. Poston L, Briley AL, Barr S, Bell R, Croker H, Coxon K, Essex HN, Hunt C, Hayes L, Howard LM, Khazaezadeh N, Kinnunen T, Nelson SM, Oteng-Ntim E, Robson SC, Sattar N, Seed PT, Wardle J, Sanders TAB, Sandall J. Developing a complex intervention for diet and activity behaviour change in obese pregnant women (the UPBEAT trial); assessment of behavioural change and process evaluation in a pilot randomised controlled trial. BMC Pregnancy and Childbirth 2013 Jul;13(148):Epub. doi: 10.1186/1471-2393-13-148. PMID: 23855708.
1038. Potempa K, Lopez M, Braun LT, Szidon JP, Fogg L, Tincknell T. Physiological outcomes of aerobic exercise training in hemiparetic stroke patients. Stroke 1995 Jan;26(1):101-105. PMID: 7839377.
1039. Preisinger E, Alacamlioglu Y, Pils K, Saradeth T, Schneider B. Therapeutic exercise in the prevention of bone loss. A controlled trial with women after menopause. American Journal of Physical Medicine & Rehabilitation 1995 Mar-Apr;74(2):120-123. PMID: 7710725.
1040. Prem V, Sahoo RC, Adhikari P. Comparison of the effects of Buteyko and Pranayama breathing techniques on quality of life in patients with asthma -- a randomized controlled trial. Clinical Rehabilitation 2013 Feb;27(2):133-141. doi: 10.1177/0269215512450521. PMID: 22837543.
1041. Premoselli S, Sioli P, Grossi A, Cerri C. Neutral wrist splinting in carpal tunnel syndrome: a 3- and 6-month clinical and neurophysiologic follow-up evaluation of nightonly splint therapy. Europa Medicophysica [Mediterranean Journal of Physical and Rehabilitation Medicine] 2006 Jun;42(2):121-126. PMID: 16767058.
1042. Price BB, Amini SB, Kappeler K. Exercise in pregnancy: effect on fitness and obstetric outcomes -- a randomized trial. Medicine and Science in Sports and Exercise 2012 Dec;44(12):2263-2269. doi: 10.1249/MSS.0b013e318267ad67. PMID: 22843114.
1043. Prince R, Devine A, Dick I, Criddle A, Kerr D, Kent N, Price R, Randell A. The effects of calcium supplementation (milk powder or tablets) and exercise on bone density in postmenopausal women. Journal of Bone and Mineral Research 1995 Jul;10(7):1068-1075. doi: 10.1002/jbmr.5650100711. PMID: 7484282.
1044. Prins JB, Bleijenberg G, Bazelmans E, Elving LD, de Boo TM, Severens JL, van der Wilt GJ, Spinhoven P, van der Meer JW. Cognitive behaviour therapy for chronic fatigue syndrome: a multicentre randomised controlled trial. Lancet 2001 Mar 17;357(9259):841-847. doi: 10.1016/S0140-6736(00)04198-2. PMID: 11265953.
1045. Promrat K, Kleiner DE, Niemeier HM, Jackvony E, Kearns M, Wands JR, Fava JL, Wing RR. Randomized controlled trial testing the effects of weight loss on nonalcoholic steatohepatitis. Hepatology 2010 Jan;51(1):121-129. doi: 10.1002/hep.23276. PMID: 19827166.
1046. Provinciali L, Giattini A, Splendiani G, Logullo F. Usefulness of hand rehabilitation after carpal tunnel surgery. Muscle & Nerve 2000 Feb;23(2):211-216. doi: 10.1002/(SICI)1097-4598(200002). PMID: 10639613.
1047. Pruitt LA, Taaffe DR, Marcus R. Effects of a one-year high-intensity versus low-intensity resistance training program on bone mineral density in older women. Journal of Bone and Mineral Research 1995 Nov;10(11):1788-1795. doi: 10.1002/jbmr.5650101123. PMID: 8592957.
1048. Przybylski BR, Dumont ED, Watkins ME, Warren SA, Beaulne AP, Lier DA. Outcomes of enhanced physical and occupational therapy service in a nursing home setting. Archives of Physical Medicine and Rehabilitation 1996 Jun;77(6):554-561. doi: 10.1016/S0003-9993(96)90294-4. PMID: 8831471.
1049. Pua YH, Cai CC, Lim KC. Treadmill walking with body weight support is no more effective than cycling when added to an exercise program for lumbar spinal stenosis: a randomised controlled trial. Australian Journal of Physiotherapy 2007;53(2):83-89. doi: 10.1016/S0004-9514(07)70040-5. PMID: 17535143.
1050. Puentedura EJ, Landers MR, Cleland JA, Mintken PE, Huijbregts P, Fernandez-de-las-Penas C. Thoracic spine thrust manipulation versus cervical spine thrust manipulation in patients with acute neck pain: a randomized clinical trial. The Journal of Orthopaedic and Sports Physical Therapy 2011 Apr;41(4):208-220. doi: 10.2519/jospt.2011.3640. PMID: 21335931.
1051. Puhan MA, Busching G, Schunemann HJ, van Oort E, Zaugg C, Frey M. Interval versus continuous high-intensity exercise in chronic obstructive pulmonary disease: a randomized trial. Annals of Internal Medicine 2006 Dec 5;145(11):816-825. doi: 10.7326/0003-4819-145-11-200612050-00006. PMID: 17146066.
1052. Qu Y. (Clinical observation on acupuncture by stages combined with exercise therapy for treatment of Bell palsy at acute stage) [Chinese - simplified characters]. Zhongguo Zhen Jiu [Chinese Acupuncture & Moxibustion] 2005 Aug;25(8):545-547. PMID: 16309052.
1053. Quilty B, Tucker M, Campbell R, Dieppe P. Physiotherapy, including quadriceps exercises and patellar taping, for knee osteoarthritis with predominant patello-femoral joint involvement: randomized controlled trial. The Journal of Rheumatology 2003 Jun;30(6):1311-1317. PMID: 12784408.
1054. Radkowski CA, Pietrobon R, Vail TP, Nunley JA 2nd, Jain NB, Easley ME. Cryotherapy temperature differences after total knee arthroplasty: a prospective randomized trial. Journal of Surgical Orthopaedic Advances 2007 Jun 1;16(2):67-72. PMID: 17592713.
1055. Raghavendra RM, Nagarathna R, Nagendra HR, Gopinath KS, Srinath BS, Ravi BD, Patil S, Ramesh BS, Nalini R. Effects of an integrated yoga programme on chemotherapy-induced nausea and emesis in breast cancer patients. European Journal of Cancer Care 2007 Nov;16(6):462-474. doi: 10.1111/j.1365-2354.2006.00739.x. PMID: 17944760.
1056. Ragonese J. A randomized trial comparing manual physical therapy to therapeutic exercises, to a combination of therapies, for the treatment of cervical radiculopathy. Orthopaedic Physical Therapy Practice 2009;21(3):71-76.
1057. Rajendran A, Pandurangi U, Murali R, Gomathi S, Vijayan V, Cherian K. Pre-operative short-term pulmonary rehabilitation for patients of chronic obstructive pulmonary disease undergoing coronary artery bypass graft surgery. Indian Heart Journal 1998 Sep-Oct;50(5):531-534. PMID: 10052279.
1058. Ramachandran A, Snehalatha C, Mary S, Mukesh B, Bhaskar AD, Vijay V. The Indian diabetes prevention programme shows that lifestyle modification and metformin prevent type 2 diabetes in Asian Indian subjects with impaired glucose tolerance (IDPP-1). Diabetologia 2006 Feb;49(2):289-297. doi: 10.1007/s00125-005-0097-z. PMID: 16391903.
1059. Rasmussen J, Laetgaard J, Lindecrona AL, Qvistgaard E, Bliddal H. Manipulation does not add to the effect of extension exercises in chronic low-back pain (LBP). A randomized, controlled, double blind study. Joint, Bone, Spine 2008 Dec;75(6):708-713. doi: 10.1016/j.jbspin.2007.12.011. PMID: 19028434.
1060. Rasmussen-Barr E, Nilsson-Wikmar L, Arvidsson I. Stabilizing training compared with manual treatment in sub-acute and chronic low-back pain. Manual Therapy 2003 Nov;8(4):233-241. doi: 10.1016/S1356-689X(03)00053-5. PMID: 14559046.
1061. Rauh K, Gabriel E, Kerschbaum E, Schuster T, von Kries R, Amann-Gassner U, Hauner H. Safety and efficacy of a lifestyle intervention for pregnant women to prevent excessive maternal weight gain: a cluster-randomized controlled trial. BMC Pregnancy and Childbirth 2013 Jul 16;13(151):Epub. doi: 10.1186/1471-2393-13-151. PMID: 23865624.
1062. Rea H, McAuley S, Stewart A, Lamont C, Roseman P, Didsbury P. A chronic disease management programme can reduce days in hospital for patients with chronic obstructive pulmonary disease. Internal Medicine Journal 2004 Nov;34(11):608-614. doi: 10.1111/j.1445-5994.2004.00672.x. PMID: 15546454.
1063. Reddell CR, Congleton JJ, Huchingson RD, Montgomery JF. An evaluation of a weightlifting belt and back injury prevention training class for airline baggage handlers. Applied Ergonomics 1992;23(5):319-329. doi: 10.1016/0003-6870(92)90293-5. PMID: 15676878.
1064. Regensteiner JG, Meyer TJ, Krupski WC, Cranford LS, Hiatt WR. Hospital versus home-based exercise rehabilitation for patients with peripheral arterial occlusive disease. Angiology 1997 Apr;48(4):291-300. doi: 10.1177/000331979704800402. PMID: 9112877.
1065. Reilly ETC, Freeman RM, Waterfield MR, Waterfield AE, Steggles P, Pedlar F. Prevention of postpartum stress incontinence in primigravidae with increased bladder neck mobility: a randomised controlled trial of antenatal pelvic floor exercises. BJOG 2002 Jan;109(1):68-76. doi: 10.1111/j.1471-0528.2002.t01-1-01116.x. PMID: 11845813.
1066. Rempel D, Tittiranonda P, Burastero S, Hudes M, So Y. Effect of keyboard keyswitch design on hand pain. Journal of Occupational and Environmental Medicine 1999 Feb;41(2):111-119. PMID: 10029956.
1067. Renault KM, Norgaard K, Nilas L, Carlsen EM, Cortes D, Pryds O, Secher NJ. The Treatment of Obese Pregnant Women (TOP) study: a randomized controlled trial of the effect of physical activity intervention assessed by pedometer with or without dietary intervention in obese pregnant women. American Journal of Obstetrics and Gynecology 2014 Feb;210(2):134.e131-134.e139. doi: 10.1016/j.ajog.2013.09.029. PMID: 24060449.
1068. Resnick B, Galik E, Pretzer-Aboff I, Gruber-Baldini AL, Russ K, Cayo J, Zimmerman S. Treatment fidelity in nursing home research: the Res-Care intervention study. Research in Gerontological Nursing 2009 Jan;2(1):30-38. doi: 10.3928/19404921-20090101-09. PMID: 20077991.
1069. Reust P, Chantraine A, Vischer TL. (Treatment of lumbar sciatica with or without neurological deficit using mechanical traction. A double-blind study) [French]. Schweizerische Medizinische Wochenschrift 1988 Feb 27;118(8):271-274. PMID: 2965827.
1070. Revel M, Mayoux-Benhamou MA, Rabourdin JP, Bagheri F, Roux C. One-year psoas training can prevent lumbar bone loss in postmenopausal women: a randomized controlled trial. Calcified Tissue International 1993 Nov;53(5):307-311. doi: 10.1007/BF01351834. PMID: 8287317.
1071. Ribeiro LHS, Prota C, Gomes CM, de Bessa J Jr, Boldarine MP, Dall'Oglio MF, Bruschini H, Srougi M. Long-term effect of early postoperative pelvic floor biofeedback on continence in men undergoing radical prostatectomy: a prospective, randomized, controlled trial. The Journal of Urology 2010 Sep;184(3):1034-1039. doi: 10.1016/j.juro.2010.05.040. PMID: 20643454.
1072. Riccio CM, Nelson DL, Bush MA. Adding purpose to the repetitive exercise of elderly women through imagery. The American Journal of Occupational Therapy 1990 Aug;44(8):714-719. doi: 10.5014/ajot.44.8.714. PMID: 2399925.
1073. Rice KL, Dewan N, Bloomfield HE, Grill J, Schult TM, Nelson DB, Kumari S, Thomas M, Geist LJ, Beaner C, Caldwell M, Niewoehner DE. Disease management program for chronic obstructive pulmonary disease: a randomized controlled trial. American Journal of Respiratory and Critical Care Medicine 2010 Oct 1;182(7):890-896. doi: 10.1164/rccm.200910-1579OC. PMID: 20075385.
1074. Richards CL, Malouin F, Bravo G, Dumas F, Wood-Dauphinee S. The role of technology in task-oriented training in persons with subacute stroke: a randomized controlled trial. Neurorehabilitation and Neural Repair 2004 Dec;18(4):199-211. doi: 10.1177/1545968304269397. PMID: 15537991.
1075. Richards CL, Malouin F, Wood-Dauphinee S, Williams JI, Bouchard JP, Brunet D. Task-specific physical therapy for optimization of gait recovery in acute stroke patients. Archives of Physical Medicine and Rehabilitation 1993 Jun;74(6):612-620. PMID: 8503751.
1076. Richards TL, Lappin MS, Acosta-Urquidi J, Kraft GH, Heide AC, Lawrie FW, Merrill TE, Melton GB, Cunningham CA. Double-blind study of pulsing magnetic field effects on multiple sclerosis. Journal of Alternative & Complementary Medicine 1997 Spring;3(1):21-29. doi: 10.1089/acm.1997.3.21. PMID: 9395691.
1077. Richter HE, Burgio KL, Brubaker L, Nygaard IE, Ye W, Weidner A, Bradley CS, Handa VL, Borello-France D, Goode PS, Zyczynski H, Lukacz ES, Schaffer J, Barber M, Meikle S, Spino C. A trial of continence pessary versus behavioral therapy versus combined therapy for stress incontinence. Obstetrics and Gynecology 2010 Mar;115(3):609-617. doi: 10.1097/AOG.0b013e3181d055d4. PMID: 20177294.
1078. Ricksten SE, Bengtsson A, Soderberg C, Thorden M, Kvist H. Effects of periodic positive airway pressure by mask on postoperative pulmonary function. Chest 1986 Jun;89(6):774-781. doi: 10.1378/chest.89.6.774. PMID: 3519107.
1079. Ridsdale L, Darbishire L, Seed PT. Is graded exercise better than cognitive behaviour therapy for fatigue? A UK randomized trial in primary care. Psychological Medicine 2004 Jan;34(1):37-49. doi: 10.1017/S0033291703001247. PMID: 14971625.
1080. Ridsdale L, Godfrey E, Chalder T, Seed P, King M, Wallace P, Wessely S, Fatigue Trialists' Group. Chronic fatigue in general practice: is counselling as good as cognitive behaviour therapy? A UK randomised trial. British Journal of General Practice 2001 Jan;51(462):19-24. PMID: 11271868.
1081. Ritter MA, Gandolf VS, Holston KS. Continuous passive motion versus physical therapy in total knee arthroplasty. Clinical Orthopaedics and Related Research 1989 Jul;(244):239-243. PMID: 2743665.
1082. Roberts L, Little P, Chapman J, Cantrell T, Pickering R, Langridge J. The back home trial. General practitioner-supported leaflets may change back pain behavior. Spine 2002 Sep 1;27(17):1821-1828. PMID: 12221342.
1083. Robinson JP, Bradway CW, Nuamah I, Pickett M, McCorkle R. Systematic pelvic floor training for lower urinary tract symptoms post-prostatectomy: a randomized clinical trial. International Journal of Urological Nursing 2008 Mar;2(1):3-13. doi: 10.1111/j.1749-771X.2007.00033.x.
1084. Robinson TN. Reducing children's television viewing to prevent obesity: a randomized controlled trial. JAMA 1999 Oct 27;282(16):1561-1567. doi: 10.1001/jama.282.16.1561. PMID: 10546696.
1085. Roche G, Ponthieux A, Parot-Shinkel E, Jousset N, Bontoux L, Dubus V, Penneau-Fontbonne D, Roquelaure Y, Legrand E, Colin D, Richard I, Fanello S. Comparison of a functional restoration program with active individual physical therapy for patients with chronic low back pain: a randomized controlled trial. Archives of Physical Medicine and Rehabilitation 2007 Oct;88(10):1229-1235. doi: 10.1016/j.apmr.2007.07.014. PMID: 17908562.
1086. Rogers LQ, Hopkins-Price P, Vicari S, Markwell S, Pamenter R, Courneya KS, Hoelzer K, Naritoku C, Edson B, Jones L, Dunnington G, Verhulst S. Physical activity and health outcomes three months after completing a physical activity behavior change intervention: persistent and delayed effects. Cancer Epidemiology, Biomarkers & Prevention 2009 May;18(5):1410-1418. doi: 10.1158/1055-9965.EPI-08-1045. PMID: 19383889.
1087. Rogers LQ, Hopkins-Price P, Vicari S, Pamenter R, Courneya KS, Markwell S, Verhulst S, Hoelzer K, Naritoku C, Jones L, Dunnington G, Lanzotti V, Wynstra J, Shah L, Edson B, Graff A, Lowy M. A randomized trial to increase physical activity in breast cancer survivors. Medicine and Science in Sports and Exercise 2009 Apr;41(4):935-946. doi: 10.1249/MSS.0b013e31818e0e1b. PMID: 19276838.
1088. Rogind H, Bibow-Nielsen B, Jensen B, Moller HC, Frimodt-Moller H, Bliddal H. The effects of a physical training program on patients with osteoarthritis of the knees. Archives of Physical Medicine and Rehabilitation 1998 Nov;79(11):1421-1427. doi: 10.1016/S0003-9993(98)90238-6. PMID: 9821904.
1089. Roland M, Dixon M. Randomized controlled trial of an educational booklet for patients presenting with back pain in general practice. Journal of the Royal College of General Practitioners 1989 Jun;39(323):244-246. PMID: 2556518.
1090. Rolland Y, Pillard F, Klapouszczak A, Reynish E, Thomas D, Andrieu S, Riviere D, Vellas B. Exercise program for nursing home residents with Alzheimer's disease: a 1-year randomized, controlled trial. Journal of the American Geriatrics Society 2007 Feb;55(2):158-165. doi: 10.1111/j.1532-5415.2007.01035.x. PMID: 17302650.
1091. Romero Zepeda EE, Cadenas Tovar M, Vargas Espinosa JM, Huape Arreola MS, Garcia Tizoc SO. Estudio comparativo de la utilidad del vendaje tipo Robert Jones y la ferula en U en las fracturas de tobillo (Comparison between Robert Jones dressing and U splint in ankle fractures) [Spanish]. Acta Ortopedica Mexicana 2008 Jan-Feb;22(1):40-44. PMID: 18672751.
1092. Ronnberg AK, Ostlund I, Fadl H, Gottvall T, Nilsson K. Intervention during pregnancy to reduce excessive gestational weight gain -- a randomised controlled trial. BJOG 2015 Mar;122(4):537-544. doi: 10.1111/1471-0528.13131. PMID: 25367823.
1093. Rootmensen GN, van Keimpema ARJ, Looysen EE, van der Schaaf L, de Haan RJ, Jansen HM. The effects of additional care by a pulmonary nurse for asthma and COPD patients at a respiratory outpatient clinic: results from a double blind, randomized clinical trial. Patient Education and Counseling 2008 Feb;70(2):179-186. doi: 10.1016/j.pec.2007.09.021. PMID: 18031971.
1094. Rose MJ, Reilly JP, Pennie B, Bowen-Jones K, Stanley IM, Slade PD. Chronic low back pain rehabilitation programs: a study of the optimum duration of treatment and a comparison of group and individual therapy. Spine 1997 Oct 1;22(19):2246-2253. PMID: 9346145.
1095. Rosendahl E, Lindelof N, Littbrand H, Yifter-Lindgren E, Lundin-Olsson L, Haglin L, Gustafson Y, Nyberg L. High-intensity functional exercise program and protein-enriched energy supplement for older persons dependent in activities of daily living: a randomised controlled trial. Australian Journal of Physiotherapy 2006;52(2):105-113. doi: 10.1016/S0004-9514(06)70045-9. PMID: 16764547.
1096. Rosenfeldt F, Braun L, Spitzer O, Bradley S, Shepherd J, Bailey M, van der Merwe J, Leong J-Y, Esmore D. Physical conditioning and mental stress reduction -- a randomised trial in patients undergoing cardiac surgery. BMC Complementary and Alternative Medicine 2011 Mar 9;11(20):Epub. doi: 10.1186/1472-6882-11-20. PMID: 21385466.
1097. Roth EJ, Stenson KW, Powley S, Oken J, Primack S, Nussbaum SB, Berkowitz M. Expiratory muscle training in spinal cord injury: a randomized controlled trial. Archives of Physical Medicine and Rehabilitation 2010 Jun;91(6):857-861. doi: 10.1016/j.apmr.2010.02.012. PMID: 20510974.
1098. Rowsell GJ, Coutts AJ, Reaburn P, Hill-Haas S. Effects of cold-water immersion on physical performance between successive matches in high-performance junior male soccer players. Journal of Sports Sciences 2009;27(6):565-573. doi: 10.1080/02640410802603855. PMID: 19308790.
1099. Rozenberg S, Delval C, Rezvani Y, Olivieri-Apicella N, Kuntz JL, Legrand E, Valat JP, Blotman F, Meadeb J, Rolland D, Hary S, Duplan B, Feldmann JL, Bourgeois P. Bed rest or normal activity for patients with acute low back pain: a randomized controlled trial. Spine 2002 Jul 15;27(14):1487-1493. PMID: 12131705.
1100. Rubin C, Recker R, Cullen D, Ryaby J, McCabe J, McLeod K. Prevention of postmenopausal bone loss by a low-magnitude, high-frequency mechanical stimuli: a clinical trial assessing compliance, efficacy, and safety. Journal of Bone and Mineral Research 2004 Mar;19(3):343-351. doi: 10.1359/JBMR.0301251. PMID: 15040821.
1101. Ruchat S-M, Davenport MH, Giroux I, Hillier M, Batada A, Sopper MM, Hammond JMS, Mottola MF. Nutrition and exercise reduce excessive weight gain in normal-weight pregnant women. Medicine and Science in Sports and Exercise 2012 Aug;44(8):1419-1426. doi: 10.1249/MSS.0b013e31825365f1. PMID: 22453250.
1102. Rudzki SJ. Injuries in Australian Army recruits. Part II: location and cause of injuries seen in recruits. Military Medicine 1997 Jul;162(7):477-480. PMID: 9232977.
1103. Rue J-PH, Armstrong DW 3rd, Frassica FJ, Deafenbaugh M, Wilckens JH. The effect of pulsed ultrasound in the treatment of tibial stress fractures. Orthopedics 2004 Nov;27(11):1192-1195. PMID: 15566133.
1104. Ruiz JR, Perales M, Pelaez M, Lopez C, Lucia A, Barakat R. Supervised exercise-based intervention to prevent excessive gestational weight gain: a randomized controlled trial. Mayo Clinic Proceedings 2013 Dec;88(12):1388-1397. doi: 10.1016/j.mayocp.2013.07.020. PMID: 24290112.
1105. Russo CR, Lauretani F, Bandinelli S, Bartali B, Cavazzini C, Guralnik JM, Ferrucci L. High-frequency vibration training increases muscle power in postmenopausal women. Archives of Physical Medicine and Rehabilitation 2003 Dec;84(12):1854-1857. doi: 10.1016/S0003-9993(03)00357-5. PMID: 14669194.
1106. Saavedra-Hernandez M, Arroyo-Morales M, Cantarero-Villanueva I, Fernandez-Lao C, Castro-Sanchez AM, Puentedura EJ, Fernandez-de-las-Penas C. Short-term effects of spinal thrust joint manipulation in patients with chronic neck pain: a randomized clinical trial. Clinical Rehabilitation 2013 Jun;27(6):504-512. doi: 10.1177/0269215512464501. PMID: 23129812.
1107. Saavedra-Hernandez M, Castro-Sanchez AM, Arroyo-Morales M, Cleland JA, Lara-Palomo IC, Fernandez-de-las-Penas C. Short-term effects of kinesiotaping versus cervical thrust manipulation in patients with mechanical neck pain: a randomized clinical trial. The Journal of Orthopaedic and Sports Physical Therapy 2012 Aug;42(8):724-730. doi: 10.2519/jospt.2012.4086. PMID: 22523090.
1108. Saayman L, Hay C, Abrahamse H. Chiropractic manipulative therapy and low-level laser therapy in the management of cervical facet dysfunction: a randomized controlled study. Journal of Manipulative and Physiological Therapeutics 2011 Mar;34(3):153-163. doi: 10.1016/j.jmpt.2011.02.010. PMID: 21492750.
1109. Sackley C, Wade DT, Mant D, Atkinson JC, Yudkin P, Cardoso K, Levin S, Lee VB, Reel K. Cluster randomized pilot controlled trial of an occupational therapy intervention for residents with stroke in UK care homes. Stroke 2006 Sep;37(9):2336-2341. doi: 10.1161/01.STR.0000237124.20596.92. PMID: 16888263.
1110. Sackley CM, Rodriguez NA, van den Berg M, Badger F, Wright C, Besemer J, van Reeuwijk KT, van Wely L. A phase II exploratory cluster randomized controlled trial of a group mobility training and staff education intervention to promote urinary continence in UK care homes. Clinical Rehabilitation 2008 Aug;22(8):714-721. doi: 10.1177/0269215508089058. PMID: 18678571.
1111. Sackley CM, van den Berg ME, Lett K, Patel S, Hollands K, Wright CC, Hoppitt TJ. Effects of a physiotherapy and occupational therapy intervention on mobility and activity in care home residents: a cluster randomised controlled trial. BMJ 2009 Sep 1;339:b3123. doi: 10.1136/bmj.b3123. PMID: 19723707.
1112. Sahin E, Akalin E, Bircan C, Karaoglan O, Tatari H, Alper S, Peker O. The effects of continuous passive motion on outcome in total knee arthroplasty. Romatoloji ve Tibbi Rehabilitasyon Dergisi [Journal of Rheumatology and Medical Rehabilitation] 2006 Jun;17(2):85-90.
1113. Sakai A, Oshige T, Zenke Y, Yamanaka Y, Nagaishi H, Nakamura T. Unipedal standing exercise and hip bone mineral density in postmenopausal women: a randomized controlled trial. Journal of Bone and Mineral Metabolism 2010 Jan;28(1):42-48. doi: 10.1007/s00774-009-0100-8. PMID: 19521657.
1114. Salacinski AJ, Krohn K, Lewis SF, Holland ML, Ireland K, Marchetti G. The effects of group cycling on gait and pain-related disability in individuals with mild-to-moderate knee osteoarthritis: a randomized controlled trial. The Journal of Orthopaedic and Sports Physical Therapy 2012 Dec;42(12):985-995. doi: 10.2519/jospt.2012.3813. PMID: 22951360.
1115. Salbach NM, Mayo NE, Robichaud-Ekstrand S, Hanley JA, Richards CL, Wood-Dauphinee S. The effect of a task-oriented walking intervention on improving balance self-efficacy poststroke: a randomized controlled trial. Journal of the American Geriatrics Society 2005 Apr;53(4):576-582. doi: 10.1111/j.1532-5415.2005.53203.x. PMID: 15817001.
1116. Salli A, Sahin N, Baskent A, Ugurlu H. The effect of two exercise programs on various functional outcome measures in patients with osteoarthritis of the knee: a randomized controlled clinical trial. Isokinetics and Exercise Science 2010 Nov;18(4):201-209. doi: 10.3233/IES-2010-0385.
1117. Salmon J, Ball K, Hume C, Booth M, Crawford D. Outcomes of a group-randomized trial to prevent excess weight gain, reduce screen behaviours and promote physical activity in 10-year-old children: switch-play. International Journal of Obesity 2008 Apr;32(4):601-612. doi: 10.1038/sj.ijo.0803805. PMID: 18253162.
1118. Salzberg CA, Cooper-Vastola SA, Perez F, Viehbeck MG, Byrne DW. The effects of non-thermal pulsed electromagnetic energy on wound healing of pressure ulcers in spinal cord-injured patients: a randomized, double-blind study. Ostomy/Wound Management 1995 Apr;41(3):42-51. PMID: 7546114.
1119. Sampaio LMM, Jamami M, Pires VA, e Silva AB, Costa D. Forca muscular respiratoria em pacientes asmaticos submetidos ao treinamento muscular respiratorio e treinamento fisico (Respiratory muscle strength in asthmatic patient submitted by respiratory muscle training and physical training) [Portuguese]. Revista de Fisioterapia da Universidade de Sao Paulo 2002 Jul-Dec;9(2):43-48.
1120. Sampselle CM, Miller JM, Mims BL, Delancey JOL, Ashton-Miller JA, Antonakos CL. Effect of pelvic muscle exercise on transient incontinence during pregnancy and after birth. Obstetrics and Gynecology 1998;91(3):406-412. PMID: 9491869.
1121. Sandel SL, Judge JO, Landry N, Faria L, Ouellette R, Majczak M. Dance and movement program improves quality-of-life measures in breast cancer survivors. Cancer Nursing 2005 Jul-Aug;28(4):301-309. PMID: 16046894.
1122. Sanderson B, Askew C, Stewart I, Walker P, Gibbs H, Green S. Short-term effects of cycle and treadmill training on exercise tolerance in peripheral arterial disease. Journal of Vascular Surgery 2006 Jul;44(1):119-127. doi: 10.1016/j.jvs.2006.03.037. PMID: 16828435.
1123. Santana-Sosa E, Barriopedro MI, Lopez-Mojares LM, Perez M, Lucia A. Exercise training is beneficial for Alzheimer's patients. International Journal of Sports Medicine 2008 Oct;29(10):845-850. doi: 10.1055/s-2008-1038432. PMID: 18401810.
1124. Santos IA, Stein R, Fuchs SC, Duncan BB, Ribeiro JP, Kroeff LR, Carballo MT, Schmidt MI. Aerobic exercise and submaximal functional capacity in overweight pregnant women: a randomized trial. Obstetrics and Gynecology 2005 Aug;106(2):243-249. doi: 10.1097/01.AOG.0000171113.36624.86. PMID: 16055571.
1125. Santos PFD, Oliveira E, Zanetti MRD, Arruda RM, Sartori MGF, Girao MJBC, Castro RA. Eletroestimulacao funcional do assoalho pelvico versus terapia com os cones vaginais para o tratamento de incontinencia urinaria de esforco (Electrical stimulation of the pelvic floor versus vaginal cone therapy for the treatment of stress urinary incontinence) [Portuguese]. Revista Brasileira de Ginecologia e Obstetricia 2009 Sep;31(9):447-452. doi: 10.1590/S0100-72032009000900005. PMID: 19876576.
1126. Saunders KB, White JE. Controlled trial of breathing exercise. British Medical Journal 1965 Sep 18;2(5463):680-682. PMID: 14337735.
1127. Sauvage LR Jr, Myklebust BM, Crow-Pan J, Novak S, Millington P, Hoffman MD, Hartz AJ, Rudman D. A clinical trial of strengthening and aerobic exercise to improve gait and balance in elderly male nursing home residents. American Journal of Physical Medicine & Rehabilitation 1992 Dec;71(6):333-342. PMID: 1466871.
1128. Savage AM. Is lumbopelvic stability training (using the Pilates model) an effective treatment strategy for women with stress urinary incontinence? A review of the literature and report of a pilot study. Journal of the Association of Chartered Physiotherapists in Women's Health 2005 Autumn;97:33-48.
1129. Savage P, Ricci MA, Lynn M, Gardner A, Knight S, Brochu M, Ades P. Effects of home versus supervised exercise for patients with intermittent claudication. Journal of Cardiopulmonary Rehabilitation 2001 May-Jun;21(3):152-157. PMID: 11409225.
1130. Savolainen A, Ahlberg J, Nummila H, Nissinen M. Active or passive treatment for neck-shoulder pain in occupational health care? A randomized controlled trial. Occupational Medicine 2004 Sep;54(6):422-424. doi: 10.1093/occmed/kqh070. PMID: 15358840.
1131. Scarcella JB, Cohn BT. The effect of cold therapy on the postoperative course of total hip and knee arthroplasty patients. American Journal of Orthopedics 1995 Nov;24(11):847-852. PMID: 8581442.
1132. Schilke JM, Johnson GO, Housh TJ, O'Dell JR. Effects of muscle-strength training on the functional status of patients with osteoarthritis of the knee joint. Nursing Research 1996 Mar-Apr;45(2):68-72. PMID: 8604366.
1133. Schimmel JJ, de Kleuver M, Horsting PP, Spruit M, Jacobs WC, van Limbeek J. No effect of traction in patients with low back pain: a single centre, single blind, randomized controlled trial of intervertebral differential dynamics therapy. European Spine Journal 2009 Dec;18(12):1843-1850. doi: 10.1007/s00586-009-1044-3. PMID: 19484433.
1134. Schlager O, Giurgea A, Schuhfried O, Seidinger D, Hammer A, Groger M, Fialka-Moser V, Gschwandtner M, Koppensteiner R, Steiner S. Exercise training increases endothelial progenitor cells and decreases asymmetric dimethylarginine in peripheral arterial disease: a randomized controlled trial. Atherosclerosis 2011 Jul;217(1):240-248. doi: 10.1016/j.atherosclerosis.2011.03.018. PMID: 21481871.
1135. Schmidt AP, Sanches PR, Silva DP Jr, Ramos JG, Nohama P. A new pelvic muscle trainer for the treatment of urinary incontinence. International Journal of Gynaecology and Obstetrics 2009 Jun;105(3):218-222. doi: 10.1016/j.ijgo.2009.01.013. PMID: 19232601.
1136. Schneiderman-Walker J, Pollock SL, Corey M, Wilkes DD, Canny GJ, Pedder L, Reisman JJ. A randomized controlled trial of a 3-year home exercise program in cystic fibrosis. The Journal of Pediatrics 2000 Mar;136(3):304-310. doi: 10.1067/mpd.2000.103408. PMID: 10700685.
1137. Schnelle JF, Alessi CA, Simmons SF, al-Samarrai NR, Beck JC, Ouslander JG. Translating clinical research into practice: a randomized controlled trial of exercise and incontinence care with nursing home residents. Journal of the American Geriatrics Society 2002 Sep;50(9):1476-1483. doi: 10.1046/j.1532-5415.2002.50401.x. PMID: 12383143.
1138. Schnelle JF, MacRae PG, Giacobassi K, MacRae HS, Simmons SF, Ouslander JG. Exercise with physically restrained nursing home residents: maximizing benefits of restraint reduction. Journal of the American Geriatrics Society 1996 May;44(5):507-512. PMID: 8617897.
1139. Schnelle JF, MacRae PG, Ouslander JG, Simmons SF, Nitta M. Functional incidental training, mobility performance, and incontinence care with nursing home residents. Journal of the American Geriatrics Society 1995 Dec;43(12):1356-1362. PMID: 7490386.
1140. Schoenfelder DP. A fall prevention program for elderly individuals. Exercise in long-term care settings. Journal of Gerontological Nursing 2000 Mar;26(3):43-51. PMID: 11111630.
1141. Schoenfelder DP, Rubenstein LM. An exercise program to improve fall-related outcomes in elderly nursing home residents. Applied Nursing Research 2004 Feb;17(1):21-31. doi: 10.1016/j.apnr.2003.10.008. PMID: 14991552.
1142. Schomacher J. The effect of an analgesic mobilization technique when applied at symptomatic or asymptomatic levels of the cervical spine in subjects with neck pain: a randomized controlled trial. The Journal of Manual & Manipulative Therapy 2009;17(2):101-108. PMID: 20046552.
1143. Schuch FB, Vasconcelos-Moreno MP, Borowsky C, Fleck MP. Exercise and severe depression: preliminary results of an add-on study. Journal of Affective Disorders 2011 Oct;133(3):615-618. doi: 10.1016/j.jad.2011.04.030. PMID: 21616540.
1144. Schuhfried O, Mittermaier C, Jovanovic T, Pieber K, Paternostro-Sluga T. Effects of whole-body vibration in patients with multiple sclerosis: a pilot study. Clinical Rehabilitation 2005 Dec;19(8):834-842. doi: 10.1191/0269215505cr919oa. PMID: 16323382.
1145. Schweikert B, Jacobi E, Seitz R, Cziske R, Ehlert A, Knab J, Leidl R. Effectiveness and cost-effectiveness of adding a cognitive-behavioral treatment to the rehabilitation of chronic low back pain. The Journal of Rheumatology 2006 Dec;33(12):2519-2526. PMID: 17143986.
1146. Schwieger I, Gamulin Z, Forster A, Meyer P, Gemperle M, Suter PM. Absence of benefit of incentive spirometry in low-risk patients undergoing elective cholecystectomy. A controlled randomized study. Chest 1986 May;89(5):652-656. doi: 10.1378/chest.89.5.652. PMID: 3698694.
1147. Schyns F, Paul L, Finlay K, Ferguson C, Noble E. Vibration therapy in multiple sclerosis: a pilot study exploring its effects on tone, muscle force, sensation and functional performance. Clinical Rehabilitation 2009 Sep;23(9):771-781. doi: 10.1177/0269215508101758. PMID: 19561035.
1148. Scrimshaw SV, Maher CG. Randomized controlled trial of neural mobilization after spinal surgery. Spine 2001 Dec 15;26(24):2647-2652. PMID: 11740347.
1149. Sedaghati P, Ziaee V, Ardjmand A. The effect of an ergometric training program on pregnants weight gain and low back pain. Gazzetta Medica Italiana 2007 Dec;166(6):209-213.
1150. Segal R, Evans W, Johnson D, Smith J, Colletta S, Gayton J, Woodard S, Wells G, Reid R. Structured exercise improves physical functioning in women with stages I and II breast cancer: results of a randomized controlled trial. Journal of Clinical Oncology 2001 Feb 1;19(3):657-665. PMID: 11157015.
1151. Segal RJ, Reid RD, Courneya KS, Malone SC, Parliament MB, Scott CG, Venner PM, Quinney HA, Jones LW, d'Angelo MES, Wells GA. Resistance exercise in men receiving androgen deprivation therapy for prostate cancer. Journal of Clinical Oncology 2003 May 1;21(9):1653-1659. doi: 10.1200/JCO.2003.09.534. PMID: 12721238.
1152. Segal RJ, Reid RD, Courneya KS, Sigal RJ, Kenny GP, Prud'Homme DG, Malone SC, Wells GA, Scott CG, Slovinec d'Angelo ME. Randomized controlled trial of resistance or aerobic exercise in men receiving radiation therapy for prostate cancer. Journal of Clinical Oncology 2009 Jan 20;27(3):344-351. doi: 10.1200/JCO.2007.15.4963. PMID: 19064985.
1153. Segar ML, Katch VL, Roth RS, Garcia AW, Portner TI, Glickman SG, Haslanger S, Wilkins EG. The effect of aerobic exercise on self-esteem and depressive and anxiety symptoms among breast cancer survivors. Oncology Nursing Forum 1998 Jan-Feb;25(1):107-113. PMID: 9460778.
1154. Segura-Orti E, Kouidi E, Lison JF. Effect of resistance exercise during hemodialysis on physical function and quality of life: randomized controlled trial. Clinical Nephrology 2009 May;71(5):527-537. PMID: 19473613.
1155. Sellwood KL, Brukner P, Williams D, Nicol A, Hinman R. Ice-water immersion and delayed-onset muscle soreness: a randomised controlled trial. British Journal of Sports Medicine 2007 Jun;41(6):392-397. doi: 10.1136/bjsm.2006.033985. PMID: 17261562.
1156. Selvadurai HC, Blimkie CJ, Meyers N, Mellis CM, Cooper PJ, van Asperen PP. Randomized controlled study of in-hospital exercise training programs in children with cystic fibrosis. Pediatric Pulmonology 2002;33(3):194-200. doi: 10.1002/ppul.10015. PMID: 11836799.
1157. Seo JT, Yoon H, Kim YH. A randomized prospective study comparing new vaginal cone and FES-biofeedback. Yonsei Medical Journal 2004;45(5):879-884. PMID: 15515199.
1158. Sevim S, Dogu O, Camdeviren H, Kaleagasi H, Aral M, Arslan E, Milcan A. Long-term effectiveness of steroid injections and splinting in mild and moderate carpal tunnel syndrome. Neurological Sciences 2004 Jun;25(2):48-52. doi: 10.1007/s10072-004-0229-0. PMID: 15221621.
1159. Shahidi M, Mojtahed A, Modabbernia A, Mojtahed M, Shafiabady A, Delavar A, Honari H. Laughter yoga versus group exercise program in elderly depressed women: a randomized controlled trial. International Journal of Geriatric Psychiatry 2011 Mar;26(3):322-327. doi: 10.1002/gps.2545. PMID: 20848578.
1160. Shakoor MA, Ahmed MS, Kibria G, Khan AA, Mian MA, Hasan SA, Nahar S, Hossain MA. Effects of cervical traction and exercise therapy in cervical spondylosis. Bangladesh Medical Research Council Bulletin 2002 Aug;28(2):61-69. PMID: 12825763.
1161. Sharpe M, Hawton K, Simkin S, Surawy C, Hackmann A, Klimes I, Peto T, Warrell D, Seagroatt V. Cognitive behaviour therapy for the chronic fatigue syndrome: a randomized controlled trial. BMJ 1996 Jan 6;312(7022):22-26. PMID: 8555852.
1162. Sheiken F, Jahdi F, Khoie EM, Alizadeh NS, Sheikhan H, Haghani H. Episiotomy discomforts relief using cold gel pads in primparous Iranian women (a comparative study). Research Journal of Medical Sciences 2011;5(3):150-154. doi: 10.3923/rjmsci.2011.150.154.
1163. Shepherd A, Montgomery E, Anderson RS. A pilot study of a pelvic exerciser in women with stress urinary incontinence. Journal of Obstetrics and Gynaecology 1983;3(3):201-202.
1164. Sherman KJ, Cherkin DC, Erro J, Miglioretti DL, Deyo RA. Comparing yoga, exercise, and a self-care book for chronic low back pain: a randomized, controlled trial. Annals of Internal Medicine 2005 Dec 20;143(12):849-856. doi: 10.7326/0003-4819-143-12-200512200-00003. PMID: 16365466.
1165. Sherman KJ, Cherkin DC, Hawkes RJ, Miglioretti DL, Deyo RA. Randomized trial of therapeutic massage for chronic neck pain. The Clinical Journal of Pain 2009 Mar-Apr;25(3):233-238. doi: 10.1097/AJP.0b013e31818b7912. PMID: 19333174.
1166. Sherman RA, Davis GD, Wong MF. Behavioral treatment of exercise-induced urinary incontinence among female soldiers. Military Medicine 1997 Oct;162(10):690-694. PMID: 9339085.
1167. Sherry E, Kitchener P, Smart R. A prospective randomized controlled study of VAX-D and TENS for the treatment of chronic low back pain. Neurological Research 2001;23(7):780-784. doi: 10.1179/016164101101199180. PMID: 11680522.
1168. Sherry MA, Best TM. A comparison of 2 rehabilitation programs in the treatment of acute hamstring strains. The Journal of Orthopaedic and Sports Physical Therapy 2004 Mar;34(3):116-125. doi: 10.2519/jospt.2004.1062. PMID: 15089024.
1169. Sihvonen SE, Sipila S, Era PA. Changes in postural balance in frail elderly women during a 4-week visual feedback training: a randomized controlled trial. Gerontology 2004 Mar-Apr;50(2):87-95. doi: 10.1159/000075559. PMID: 14963375.
1170. Sillevis R, Cleland J, Hellman M, Beekhuizen K. Immediate effects of a thoracic spine thrust manipulation on the autonomic nervous system: a randomized clinical trial. The Journal of Manual & Manipulative Therapy 2010 Dec;18(4):181-190. doi: 10.1179/106698110X12804993427126. PMID: 22131791.
1171. Silva CS, Torres LA, Rahal A, Terra Filho J, Vianna EO. Comparison of morning and afternoon exercise training for asthmatic children. Brazilian Journal of Medical and Biological Research [Revista Brasileira de Pesquisas Medicas e Biologicas] 2006 Jan;39(1):71-78. doi: 10.1590/S0100-879X2006000100008. PMID: 16400466.
1172. Simao AP, Avelar NC, Tossige-Gomes R, Neves CD, Mendonca VA, Miranda AS, Teixeira MM, Teixeira AL, Andrade AP, Coimbra CC, Lacerda AC. Functional performance and inflammatory cytokines after squat exercises and whole-body vibration in elderly individuals with knee osteoarthritis. Archives of Physical Medicine and Rehabilitation 2012 Oct;93(10):1692-1700. doi: 10.1016/j.apmr.2012.04.017. PMID: 22546535.
1173. Simmerman SM, Sizer PS, Dedrick GS, Apte GG, Brismee JM. Immediate changes in spinal height and pain after aquatic vertical traction in patients with persistent low back symptoms: a crossover clinical trial. PM&R 2011 May;3(5):447-457. doi: 10.1016/j.pmrj.2011.01.010. PMID: 21570033.
1174. Simon C, Schweitzer B, Oujaa M, Wagner A, Arveiler D, Triby E, Copin N, Blanc S, Platat C. Successful overweight prevention in adolescents by increasing physical activity: a 4-year randomized controlled intervention. International Journal of Obesity 2008 Oct;32(10):1489-1498. doi: 10.1038/ijo.2008.99. PMID: 18626482.
1175. Sims J, Galea M, Taylor N, Dodd K, Jespersen S, Joubert L, Joubert J. Regenerate: assessing the feasibility of a strength-training program to enhance the physical and mental health of chronic post stroke patients with depression. International Journal of Geriatric Psychiatry 2009 Jan;24(1):76-83. doi: 10.1002/gps.2082. PMID: 18613281.
1176. Sin DD, Wong E, Mayers I, Lien DC, Feeny D, Cheung H, Gan W, Man SFP. Effects of nocturnal noninvasive mechanical ventilation on heart rate variability of patients with advanced COPD. Chest 2007 Jan;131(1):156-163. doi: 10.1378/chest.06-1423. PMID: 17218570.
1177. Sinaki M, Wahner HW, Offord KP, Hodgson SF. Efficacy of nonloading exercises in prevention of vertebral bone loss in postmenopausal women: a controlled trial. Mayo Clinic Proceedings 1989;64(7):762-769. PMID: 2671517.
1178. Singh AS, Chin A Paw MJM, Brug J, van Mechelen W. Dutch obesity intervention in teenagers: effectiveness of a school-based program on body composition and behavior. Archives of Pediatrics & Adolescent Medicine 2009 Apr;163(4):309-317. doi: 10.1001/archpediatrics.2009.2. PMID: 19349559.
1179. Singh NA, Clements KM, Fiatarone MA. A randomized controlled trial of progressive resistance training in depressed elders. The Journals of Gerontology. Series A, Biological Sciences and Medical Sciences 1997 Jan;52(1):M27-M35. PMID: 9008666.
1180. Singh NA, Stavrinos TM, Scarbek Y, Galambos G, Liber C, Fiatarone Singh MA. A randomized controlled trial of high versus low intensity weight training versus general practitioner care for clinical depression in older adults. The Journals of Gerontology. Series A, Biological Sciences and Medical Sciences 2005 Jun;60(6):768-776. PMID: 15983181.
1181. Singh S, Soni R, Singh KP, Tandon OP. Effect of yoga practices on pulmonary function tests including transfer factor of lung for carbon monoxide (TLCO) in asthma patients. Indian Journal of Physiology and Pharmacology 2012 Jan-Mar;56(1):63-68. PMID: 23029966.
1182. Singhal N, Misra A, Shah P, Gulati S. Effects of controlled school-based multi-component model of nutrition and lifestyle interventions on behavior modification, anthropometry and metabolic risk profile of urban Asian Indian adolescents in North India. European Journal of Clinical Nutrition 2010 Apr;64(6):364-373. doi: 10.1038/ejcn.2009.150. PMID: 20087379.
1183. Skillgate E, Vingard E, Alfredsson L. Naprapathic manual therapy or evidence-based care for back and neck pain: a randomized, controlled trial. The Clinical Journal of Pain 2007 Jun;23(5):431-439. doi: 10.1097/AJP.0b013e31805593d8. PMID: 17515742.
1184. Skouen JS, Grasdal AL, Haldorsen EM, Ursin H. Relative cost-effectiveness of extensive and light multidisciplinary treatment programs versus treatment as usual for patients with chronic low back pain on long-term sick leave: randomized controlled study. Spine 2002 May 1;27(9):901-909. PMID: 11979157.
1185. Sleep J, Grant A. Pelvic floor exercises in postnatal care. Midwifery 1987 Dec;3(4):158-164. doi: 10.1016/S0266-6138(87)80035-9. PMID: 3320686.
1186. Sloop PR, Smith DS, Goldenberg E, Dore C. Manipulation for chronic neck pain. A double-blind controlled study. Spine 1982 Nov-Dec;7(6):532-535. PMID: 6762670.
1187. Smania N, Corato E, Fiaschi A, Pietropoli P, Aglioti SM, Tinazzi M. Therapeutic effects of peripheral repetitive magnetic stimulation on myofascial pain syndrome. Clinical Neurophysiology 2003 Feb;114(2):350-358. doi: 10.1016/S1388-2457(02)00367-X. PMID: 12559244.
1188. Smania N, Corato E, Fiaschi A, Pietropoli P, Aglioti SM, Tinazzi M. Repetitive magnetic stimulation: a novel therapeutic approach for myofascial pain syndrome. Journal of Neurology 2005 Mar;252(3):307-314. doi: 10.1007/s00415-005-0642-1. PMID: 15726272.
1189. Smeets RJ, Vlaeyen JWS, Hidding A, Kester AD, van der Heijden GJ, van Geel AC, Knottnerus JA. Active rehabilitation for chronic low back pain: cognitive-behavioral, physical, or both? First direct post-treatment results from a randomized controlled trial. BMC Musculoskeletal Disorders 2006 Jan 20;7(5):Epub. doi: 10.1186/1471-2474-7-5. PMID: 16426449.
1190. Smidt GL, Lin SY, O'Dwyer KD, Blanpied PR. The effect of high-intensity trunk exercise on bone mineral density of postmenopausal women. Spine 1992 Mar;17(3):280-285. PMID: 1566165.
1191. Smidt N, Giffel MAB, Gerards-Last TMAJ, de Vet HCW. Effectiviteit van myofeedback bij vrouwen met streeincontinentie [Dutch]. Nederlands Tijdschrift voor Fysiotherapie [Dutch Journal of Physical Therapy] 1997 Sep;107(5):121-127.
1192. Smith BJ, Appleton SL, Bennett PW, Roberts GC, del Fante P, Adams R, Trott CM, Allan DP, Southcott AM, Ruffin RE. The effect of a respiratory home nurse intervention in patients with chronic obstructive pulmonary disease (COPD). Australian and New Zealand Journal of Medicine 1999 Oct;29(5):718-725. doi: 10.1111/j.1445-5994.1999.tb01621.x. PMID: 10630654.
1193. Smith J, Stevens J, Taylor M, Tibbey J. A randomized, controlled trial comparing bandaging and cold therapy in postoperative total knee replacement surgery. Orthopaedic Nursing 2002 Mar-Apr;21(2):61-66. PMID: 11949239.
1194. Smith PS, Thompson M. Treadmill training post stroke: are there any secondary benefits? A pilot study. Clinical Rehabilitation 2008 Oct-Nov;22(10-11):997-1002. doi: 10.1177/0269215508088988. PMID: 18955431.
1195. Soderlund A, Lindberg P. Cognitive behavioural components in physiotherapy management of chronic whiplash associated disorders (WAD) -- a randomised group study. Physiotherapy Theory and Practice 2001;17(4):229-238. doi: 10.1080/095939801753385735.
1196. Sodhi C, Singh S, Dandona PK. A study of the effect of yoga training on pulmonary functions in patients with bronchial asthma. Indian Journal of Physiology and Pharmacology 2009 Apr-Jun;53(2):169-174. PMID: 20112821.
1197. Sondenaa K, Hoigaard U, Smith D, Alho A. Immobilization of operated ankle fractures. Acta Orthopaedica Scandinavica 1986 Feb;57(1):59-61. PMID: 3083644.
1198. Song R, Lee EO, Lam P, Bae SC. Effects of Tai Chi exercise on pain, balance, muscle strength, and perceived difficulties in physical functioning in older women with osteoarthritis: a randomized clinical trial. The Journal of Rheumatology 2003 Sep;30(9):2039-2044. PMID: 12966613.
1199. Soriano F, Rios R. Gallium arsenide laser treatment of chronic low back pain: a prospective, randomized and double blind study. Laser Therapy 1998;10(4):175-180. doi: 10.5978/islsm.10.175.
1200. Soukup MG, Glomsrod B, Lonn JH, Bo K, Larsen S. The effect of a mensendieck exercise program as secondary prophylaxis for recurrent low back pain: a randomised, controlled trial with 12-month follow-up. Spine 1999 Aug 1;24(15):1585-1592. PMID: 10457579.
1201. Sparks KE, Shaw DK, Eddy D, Hanigosky P, Vantrese J. Alternatives for cardiac rehabilitation patients unable to return to a hospital-based program. Heart & Lung 1993 Jul-Aug;22(4):298-303. PMID: 8360063.
1202. Speck RM, Gross CR, Hormes JM, Ahmed RL, Lytle LA, Hwang WT, Schmitz KH. Changes in the Body Image and Relationship Scale following a one-year strength training trial for breast cancer survivors with or at risk for lymphedema. Breast Cancer Research and Treatment 2009 Jun;121(2):421-430. doi: 10.1007/s10549-009-0550-7. PMID: 19771507.
1203. Sriboonreung T, Wongtra-Ngan S, Eungpinichpong W, Laopaiboon M. Effectiveness of pelvic floor muscle training in incontinent women at Maharaj Nakorn Chiang Mai Hospital: a randomized controlled trial. Chotmaihet Thangphaet [Journal of the Medical Association of Thailand] 2011 Jan;94(1):1-7. PMID: 21425721.
1204. Sridhar M, Taylor R, Dawson S, Roberts NJ, Partridge MR. A nurse led intermediate care package in patients who have been hospitalised with an acute exacerbation of chronic obstructive pulmonary disease. Thorax 2008 Mar;63(3):194-200. doi: 10.1136/thx.2007.077578. PMID: 17901162.
1205. Staal JB, Hlobil H, Twisk JWR, Smid T, Koke AJA, van Mechelen W. Graded activity for low back pain in occupational health care: a randomized, controlled trial. Annals of Internal Medicine 2004 Jan 20;140(2):77-84. doi: 10.7326/0003-4819-140-2-200401200-00007. PMID: 14734329.
1206. Stafne SN, Salvesen KA, Romundstad PR, Eggebo TM, Carlsen SM, Morkved S. Regular exercise during pregnancy to prevent gestational diabetes: a randomized controlled trial. Obstetrics and Gynecology 2012 Jan;119(1):29-36. doi: 10.1097/AOG.0b013e3182393f86. PMID: 22183208.
1207. Stafne SN, Salvesen KA, Romundstad PR, Stuge B, Morkved S. Does regular exercise during pregnancy influence lumbopelvic pain? A randomized controlled trial. Acta Obstetricia et Gynecologica Scandinavica 2012 May;91(5):552-559. doi: 10.1111/j.1600-0412.2012.01382.x. PMID: 22364387.
1208. Stamford BA. Physiological effects of training upon institutionalized geriatric men. Journal of Gerontology 1972 Oct;27(4):451-455. doi: 10.1093/geronj/27.4.451. PMID: 5075488.
1209. Stankovic R, Johnell O. Conservative treatment of acute low-back pain. A prospective randomized trial: McKenzie method of treatment versus patient education in "mini back school". Spine 1990 Feb;15(2):120-123. PMID: 2139241.
1210. Steen M. A randomised controlled trial to evaluate the effectiveness of localised cooling treatments in alleviating perineal trauma: the APT study. MIDIRS Midwifery Digest 2002 Sep;12(3):373-376.
1211. Steen M, Cooper K, Marchant P, Griffiths-Jones M, Walker J. A randomised controlled trial to compare the effectiveness of icepacks and epifoam with cooling maternity gel pads at alleviating postnatal perineal trauma. Midwifery 2000 Mar;16(1):48-55. doi: 10.1054/midw.1999.0188. PMID: 11139861.
1212. Steenstra IA, Anema JR, Bongers PM, de Vet HC, Knol DL, van Mechelen W. The effectiveness of graded activity for low back pain in occupational healthcare. Occupational and Environmental Medicine 2006 Nov;63(11):718-725. doi: 10.1136/oem.2005.021675. PMID: 16847036.
1213. Sterling M, Pedler A, Chan C, Puglisi M, Vuvan V, Vicenzino B. Cervical lateral glide increases nociceptive flexion reflex threshold but not pressure or thermal pain thresholds in chronic whiplash associated disorders: a pilot randomised controlled trial. Manual Therapy 2010 Apr;15(2):149-153. doi: 10.1016/j.math.2009.09.004. PMID: 19884037.
1214. Stevens J, Killeen M. A randomised controlled trial testing the impact of exercise on cognitive symptoms and disability of residents with dementia. Contemporary Nurse 2006 Feb-Mar;21(1):32-40. PMID: 16594879.
1215. Stewart AH, Smith FC, Baird RN, Lamont PM. Local versus systemic mechanisms underlying supervised exercise training for intermittent claudication. Vascular and Endovascular Surgery 2008 Aug-Sep;42(4):314-320. doi: 10.1177/1538574408314442. PMID: 18319355.
1216. Stewart AL, Verboncoeur CJ, McLellan BY, Gillis DE, Rush S, Mills KM, King AC, Ritter P, Brown BW Jr, Bortz WM 2nd. Physical activity outcomes of CHAMPS II: a physical activity promotion program for older adults. The Journals of Gerontology. Series A, Biological Sciences and Medical Sciences 2001 Aug;56(8):M465-M470. PMID: 11487597.
1217. Stigt JA, Uil SM, van Riesen SJH, Simons FJNA, Denekamp M, Shahin GM, Groen HJM. A randomized controlled trial of postthoracotomy pulmonary rehabilitation in patients with resectable lung cancer. Journal of Thoracic Oncology 2013 Feb;8(2):214-221. doi: 10.1097/JTO.0b013e318279d52a. PMID: 23238118.
1218. Stock MC, Downs JB, Gauer PK, Alster JM, Imrey PB. Prevention of postoperative pulmonary complications with CPAP, incentive spirometry, and conservative therapy. Chest 1985 Feb;87(2):151-157. doi: 10.1378/chest.87.2.151. PMID: 3881226.
1219. Stockle U, Konig B, Tempka A, Sudkamp NP. (Cast immobilization versus vacuum stabilizing system. Early functional results after osteosynthesis of ankle joint fractures) [German]. Der Unfallchirurg [The Accident Surgeon] 2000 Mar;103(3):215-219. doi: 10.1007/s001130050525. PMID: 10800385.
1220. Storheim K, Brox JI, Holm I, Koller AK, Bo K. Intensive group training versus cognitive intervention in sub-acute low back pain: short-term results of a single-blind randomized controlled trial. Journal of Rehabilitation Medicine 2003 May;35(3):132-140. doi: 10.1080/16501970310010484. PMID: 12809196.
1221. Straw LB, Harley RK. Assessment and training in orientation and mobility for older persons: program developing and testing. Journal of Visual Impairment & Blindness 1991;85(3):291-296.
1222. Straw LB, Harley RK, Zimmermann GJ. A program in orientation and mobility for visually impaired persons over age 60. Journal of Visual Impairment & Blindness 1991;85(3):108-113.
1223. Strijbos JH, Postma DS, van Altena R, Gimeno F, Koeter GH. A comparison between an outpatient hospital-based pulmonary rehabilitation program and a home-care pulmonary rehabilitation program in patients with COPD. A follow-up of 18 months. Chest 1996 Feb;109(2):366-372. doi: 10.1378/chest.109.2.366. PMID: 8620707.
1224. Strong J. Incorporating cognitive-behavioral therapy with occupational therapy: a comparative study with patients with low back pain. Journal of Occupational Rehabilitation 1998 Mar;8(1):61-71. doi: 10.1023/A:1023008632131.
1225. Strumpf DA, Millman RP, Carlisle CC, Grattan LM, Ryan SM, Erickson AD, Hill NS. Nocturnal positive-pressure ventilation via nasal mask in patients with severe chronic obstructive pulmonary disease. The American Review of Respiratory Disease 1991 Dec;144(6):1234-1239. doi: 10.1164/ajrccm/144.6.1234. PMID: 1741532.
1226. Strumse YA, Stanghelle JK, Utne L, Utne P, Svendsby EK. Treatment of patients with postpolio syndrome in a warm climate. Disability and Rehabilitation 2003;25(2):77-84. doi: 10.1080/0963828021000007923. PMID: 12554382.
1227. Strunk RG, Hondras MA. A feasibility study assessing manual therapies to different regions of the spine for patients with subacute or chronic neck pain. Journal of Chiropractic Medicine 2008 Mar;7(1):1-8. doi: 10.1016/j.jcme.2007.10.004. PMID: 19674713.
1228. Stuart PR, Brumby C, Smith SR. Comparative study of functional bracing and plaster cast treatment of stable lateral malleolar fractures. Injury 1989 Nov;20(6):323-326. doi: 10.1016/0020-1383(89)90003-X. PMID: 2516838.
1229. Stuckey SJ, Jacobs A, Goldfarb J. EMG biofeedback training, relaxation training, and placebo for the relief of chronic back pain. Perceptual and Motor Skills 1986 Dec;63(3):1023-1036. doi: 10.2466/PMS.63.7.1023. PMID: 2949196.
1230. Sun J-X, Yin M-X, Shao H, Li Z-S, Li S-W. (Effect of respiratory muscle gymnastics on lung function and quality of life in the old patients with chronic obstructive pulmonary disease) [Chinese - simplified characters]. Zhongguo Linchuang Kangfu [Chinese Journal of Clinical Rehabilitation] 2003 Nov 5;7(27):3698-3699.
1231. Sung K. The effects of 16-week group exercise program on physical function and mental health of elderly Korean women in long-term assisted living facility. The Journal of Cardiovascular Nursing 2009 Sep-Oct;24(5):344-351. doi: 10.1097/JCN.0b013e3181a80faf. PMID: 19652619.
1232. Suputtitada A, Wacharapreechanont T, Chaisayan P. Effect of the "sitting pelvic tilt exercise" during the third trimester in primigravidas on back pain. Chotmaihet Thangphaet [Journal of the Medical Association of Thailand] 2002;85(Suppl 1):S170-179. PMID: 12188409.
1233. Sutbeyaz ST, Sezer N, Koseoglu BF. The effect of pulsed electromagnetic fields in the treatment of cervical osteoarthritis: a randomized, double-blind, sham-controlled trial. Rheumatology International 2006 Feb;26(4):320-324. doi: 10.1007/s00296-005-0600-3. PMID: 15986086.
1234. Svernlov B, Larsson M, Rehn K, Adolfsson L. Conservative treatment of the cubital tunnel syndrome. The Journal of Hand Surgery - European Volume 2009 Apr;34(2):201-207. doi: 10.1177/1753193408098480. PMID: 19282413.
1235. Svetkey LP, Stevens VJ, Brantley PJ, Appel LJ, Hollis JF, Loria CM, Vollmer WM, Gullion CM, Funk K, Smith P, Samuel-Hodge C, Myers V, Lien LF, Laferriere D, Kennedy B, Jerome GJ, Heinith F, Harsha DW, Evans P, Erlinger TP, Dalcin AT, Coughlin J, Charleston J, Champagne CM, Bauck A, Ard JD, Aicher K, for the Weight Loss Maintenance Collaborative Research Group. Comparison of strategies for sustaining weight loss: the weight loss maintenance randomized controlled trial. JAMA 2008 Mar 12;299(10):1139-1148. doi: 10.1001/jama.299.10.1139. PMID: 18334689.
1236. Sweetman BJ, Heinrich I, Anderson JAD. A randomized controlled trial of exercises, short wave diathermy, and traction for low back pain, with evidence of diagnosis-related response to treatment. Journal of Orthopaedic Rheumatology 1993;6(4):159-166.
1237. Szpalski M, Hayez JP. How many days of bed rest for acute low back pain? Objective assessment of trunk function. European Spine Journal 1992;1(1):29-31. doi: 10.1007/BF00302139. PMID: 20054944.
1238. Taimela S, Takala EP, Asklof T, Seppala K, Parviainen S. Active treatment of chronic neck pain: a prospective randomized intervention. Spine 2000 Apr 15;25(8):1021-1027. PMID: 10767816.
1239. Tak E, Staats P, van Hespen A, Hopman-Rock M. The effects of an exercise program for older adults with osteoarthritis of the hip. The Journal of Rheumatology 2005 Jun;32(6):1106-1113. PMID: 15940775.
1240. Takami A, Wakayama S. Effects of partial body weight support while training acute stroke patients to walk backwards on a treadmill -- a controlled clinical trial using randomized allocation. Journal of Physical Therapy Science 2010 May;22(2):177-187. doi: 10.1589/jpts.22.177.
1241. Tal-Akabi A, Rushton A. An investigation to compare the effectiveness of carpal bone mobilisation and neurodynamic mobilisation as methods of treatment for carpal tunnel syndrome. Manual Therapy 2000 Nov;5(4):214-222. doi: 10.1054/math.2000.0355. PMID: 11052900.
1242. Talbot LA, Gaines JM, Huynh TN, Metter EJ. A home-based pedometer-driven walking program to increase physical activity in older adults with osteoarthritis of the knee: a preliminary study. Journal of the American Geriatrics Society 2003 Mar;51(3):387-392. doi: 10.1046/j.1532-5415.2003.51113.x. PMID: 12588583.
1243. Tamplin J, Baker FA, Grocke D, Brazzale DJ, Pretto JJ, Ruehland WR, Buttifant M, Brown DJ, Berlowitz DJ. The effect of singing on respiratory function, voice, and mood following quadriplegia: a randomized controlled trial. Archives of Physical Medicine and Rehabilitation 2013 Mar;94(3):426-434. doi: 10.1016/j.apmr.2012.10.006. PMID: 23103430.
1244. Tang M-F, Liou T-H, Lin C-C. Improving sleep quality for cancer patients: benefits of a home-based exercise intervention. Supportive Care in Cancer 2010 Oct;18(10):1329-1339. doi: 10.1007/s00520-009-0757-5. PMID: 19834744.
1245. Tappen RM. The effect of skill training on functional abilities of nursing home residents with dementia. Research in Nursing & Health 1994 Jun;17(3):159-165. doi: 10.1002/nur.4770170303. PMID: 8184127.
1246. Tappen RM, Roach KE, Applegate EB, Stowell P. Effect of a combined walking and conversation intervention on functional mobility of nursing home residents with Alzheimer disease. Alzheimer Disease & Associated Disorders 2000 Oct-Dec;14(4):196-201. PMID: 11186596.
1247. Targ EF, Levine EG. The efficacy of a mind-body-spirit group for women with breast cancer: a randomized controlled trial. General Hospital Psychiatry 2002 Jul-Aug;24(4):238-248. doi: 10.1016/S0163-8343(02)00191-3. PMID: 12100834.
1248. Targino RA, Imamura M, Kaziyama HHS, Souza LPM, Hsing WT, Furlan AD, Imamura ST, Neto RSA. A randomized controlled trial of acupuncture added to usual treatment for fibromyalgia. Journal of Rehabilitation Medicine 2008 Jul;40(7):582-588. doi: 10.2340/16501977-0216. PMID: 18758677.
1249. Tate DF, Wing RR, Winett RA. Using internet technology to deliver a behavioral weight loss program. JAMA 2001 Mar 7;285(9):1172-1177. doi: 10.1001/jama.285.9.1172. PMID: 11231746.
1250. Teixeira-Salmela LF, Olney SJ, Nadeau S, Brouwer B. Muscle strengthening and physical conditioning to reduce impairment and disability in chronic stroke survivors. Archives of Physical Medicine and Rehabilitation 1999 Oct;80(10):1211-1218. PMID: 10527076.
1251. Tejero M, Marco E, Boza R, Selva F, Piqueras M, Guillen A, Castillo MT, Muniesa JM. Incontinencia urinaria de esfuerzo y ejercicios del suelo pelvico. Estudio comparativo entre la eficacia de un entrenamiento individualizado frente a instrucciones basicas (Stress urinary incontinence and pelvic floor muscle exercises: effectiveness two different training intensive versus home instructions) [Spanish]. Trauma 2008 Jul-Sep;19(3):171-177.
1252. Tesio L, Merlo A. Autotraction versus passive traction: an open controlled study in lumbar disc herniation. Archives of Physical Medicine and Rehabilitation 1993 Aug;74(8):871-876. PMID: 8347073.
1253. Tew G, Nawaz S, Zwierska I, Saxton JM. Limb-specific and cross-transfer effects of arm-crank exercise training in patients with symptomatic peripheral arterial disease. Clinical Science 2009 Dec;117(12):405-413. doi: 10.1042/CS20080688. PMID: 19388883.
1254. Thangaraju P, Moey CB. Perineal cold pads versus oral analgesics in the relief of postpartum perineal wound pain. Singapore General Hospital Proceedings 2006;15(1):8-12.
1255. Theander K, Jakobsson P, Jorgensen N, Unosson M. Effects of pulmonary rehabilitation on fatigue, functional status and health perceptions in patients with chronic obstructive pulmonary disease: a randomized controlled trial. Clinical Rehabilitation 2009 Feb;23(2):125-136. doi: 10.1177/0269215508096174. PMID: 19164400.
1256. Thomas IL, Nicklin J, Pollock H, Faulkner K. Evaluation of a maternity cushion (Ozzlo pillow) for backache and insomnia in late pregnancy. Australian & New Zealand Journal of Obstetrics & Gynaecology 1989 May;29(2):133-138. doi: 10.1111/j.1479-828X.1989.tb01702.x. PMID: 2529841.
1257. Thomas KS, Muir KR, Doherty M, Jones AC, O'Reilly SC, Bassey EJ. Home based exercise programme for knee pain and knee osteoarthritis: randomised controlled trial. BMJ 2002 Oct 5;325(7367):752-756. doi: 10.1136/bmj.325.7367.752. PMID: 12364304.
1258. Thomas M, McKinley RK, Freeman E, Foy C, Prodger P, Price D. Breathing retraining for dysfunctional breathing in asthma: a randomised controlled trial. Thorax 2003 Feb;58(2):110-115. doi: 10.1136/thorax.58.2.110. PMID: 12554890.
1259. Thomas M, McKinley RK, Mellor S, Watkin G, Holloway E, Scullion J, Shaw DE, Wardlaw A, Price D, Pavord I. Breathing exercises for asthma: a randomised controlled trial. Thorax 2009 Jan;64(1):55-61. doi: 10.1136/thx.2008.100867. PMID: 19052047.
1260. Thomsen NO, Petersen MS, Hovgaard C. Treatment of hyperextension injuries to the PIP joint. Journal of Hand Surgery - British Volume 1995 Jun;20(3):383-384. doi: 10.1016/S0266-7681(05)80098-1. PMID: 7561417.
1261. Thorsen L, Skovlund E, Stromme SB, Hornslien K, Dahl AA, Fossa SD. Effectiveness of physical activity on cardiorespiratory fitness and health-related quality of life in young and middle-aged cancer patients shortly after chemotherapy. Journal of Clinical Oncology 2005 Apr 1;23(10):2378-2388. doi: 10.1200/JCO.2005.04.106. PMID: 15800330.
1262. Thorstensson CA, Roos EM, Petersson IF, Ekdahl C. Six-week high-intensity exercise program for middle-aged patients with knee osteoarthritis: a randomized controlled trial. BMC Musculoskeletal Disorders 2005 May 30;6(27):Epub. doi: 10.1186/1471-2474-6-27. PMID: 15924620.
1263. Thuile C, Walzl M. Evaluation of electromagnetic fields in the treatment of pain in patients with lumbar radiculopathy or the whiplash syndrome. Neurorehabilitation 2002;17(1):63-67. PMID: 12016348.
1264. Tibaek S, Klarskov P, Hansen BL, Thomsen H, Andresen H, Jensen CS, Olsen MN. Pelvic floor muscle training before transurethral resection of the prostate: a randomized, controlled, blinded study. Scandinavian Journal of Urology and Nephrology 2007 Sep;41(4):329-334. doi: 10.1080/00365590601183584. PMID: 17763226.
1265. Tienforti D, Sacco E, Marangi F, d'Addessi A, Racioppi M, Gulino G, Pinto F, Totaro A, d'Agostino D, Bassi P. Efficacy of an assisted low-intensity programme of perioperative pelvic floor muscle training in improving the recovery of continence after radical prostatectomy: a randomized controlled trial. BJU International 2012 Oct;110(7):1004-1010. doi: 10.1111/j.1464-410X.2012.10948.x. PMID: 22332815.
1266. Timm KE. A randomized-control study of active and passive treatments for chronic low back pain following L5 laminectomy. The Journal of Orthopaedic and Sports Physical Therapy 1994 Dec;20(6):276-286. PMID: 7849747.
1267. Tisi PV, Hulse M, Chulakadabba A, Gosling P, Shearman CP. Exercise training for intermittent claudication: does it adversely affect biochemical markers of the exercise-induced inflammatory response?. European Journal of Vascular and Endovascular Surgery 1997 Nov;14(5):344-350. PMID: 9413374.
1268. Tittiranonda P, Rempel D, Armstrong T, Burastero S. Effect of four computer keyboards in computer users with upper extremity musculoskeletal disorders. American Journal of Industrial Medicine 1999 Jun;35(6):647-661. doi: 10.1002/(SICI)1097-0274(199906)35:6<647::AID-AJIM12>3.0.CO;2-5. PMID: 10332518.
1269. Tobia I, Gonzalez MS, Martinez P, Tejerizo JC, Gueglio G, Damia O, Marti MI, Giudice CA. Estudio randomizado sobre continencia urinaria postprostatectomia radical con rehabilitacion perineal kiesica previa (Randomized study on urinary continence after radical prostatectomy with previous kinesic perineal physiotherapy) [Spanish]. Archivos Espanoles de Urologia 2008 Sep;61(7):793-798. PMID: 18972914.
1270. Toledano-Zarhi A, Tanne D, Carmeli E, Katz-Leurer M. Feasibility, safety and efficacy of an early aerobic rehabilitation program for patients after minor ischemic stroke: a pilot randomized controlled trial. Neurorehabilitation 2011;28(2):85-90. doi: 10.3233/NRE-2011-0636. PMID: 21447908.
1271. Tolomio S, Lalli A, Travain G, Zaccaria M. Effetti di un programma combinato di esercizio con e senza sovraccarico (acqua termale) sulla massa e qualita ossea in un gruppo di donne in eta post-menopausale con deficit minerale osseo (Effects of a combined weight and non weight-bearing (water) exercise program on bone mass and quality in postmenopausal women with low bone mineral density) [Italian]. La Clinica Terapeutica 2009 Mar-Apr;160(2):105-109. PMID: 19452097.
1272. Topp R, Woolley S, Hornyak J III, Khuder S, Kahaleh B. The effect of dynamic versus isometric resistance training on pain and functioning among adults with osteoarthritis of the knee. Archives of Physical Medicine and Rehabilitation 2002 Sep;83(9):1187-1195. doi: 10.1053/apmr.2002.33988. PMID: 12235596.
1273. Toussaint ND, Polkinghorne KR, Kerr PG. Impact of intradialytic exercise on arterial compliance and B-type natriuretic peptide levels in hemodialysis patients. Hemodialysis International 2008 Apr;12(2):254-263. doi: 10.1111/j.1542-4758.2008.00262.x. PMID: 18394060.
1274. Toya S, Motegi M, Inomata K, Ohshiro T, Maeda T. Report on a computer-randomized double blind clinical trial to determine the effectiveness of the GaAlAs (830 NM) diode laser for pain attenuation in selected pain groups. Laser Therapy 1994;6(3):143-148. doi: 10.5978/islsm.94-OR-08.
1275. Trappenburg JCA, Monninkhof EM, Bourbeau J, Troosters T, Schrijvers AJP, Verheij TJM, Lammers J-WJ. Effect of an action plan with ongoing support by a case manager on exacerbation-related outcome in patients with COPD: a multicentre randomised controlled trial. Thorax 2011 Nov;66(11):977-984. doi: 10.1136/thoraxjnl-2011-200071. PMID: 21785156.
1276. Treat-Jacobson D, Bronas UG, Leon AS. Efficacy of arm-ergometry versus treadmill exercise training to improve walking distance in patients with claudication. Vascular Medicine 2009 Aug;14(3):203-213. doi: 10.1177/1358863X08101858. PMID: 19651669.
1277. Trevino RP, Yin Z, Hernandez A, Hale DE, Garcia OA, Mobley C. Impact of the Bienestar school-based diabetes mellitus prevention program on fasting capillary glucose levels: a randomized controlled trial. Archives of Pediatrics & Adolescent Medicine 2004 Sep;158(9):911-917. doi: 10.1001/archpedi.158.9.911. PMID: 15351759.
1278. Trock DH, Bollet AJ, Markoll R. The effect of pulsed electromagnetic fields in the treatment of osteoarthritis of the knee and cervical spine. Report of randomized, double blind, placebo controlled trials. The Journal of Rheumatology 1994 Oct;21(10):1903-1911. PMID: 7837158.
1279. Tropp H, Norlin R. Ankle performance after ankle fracture: a randomized study of early mobilization. Foot & Ankle International 1995 Feb;16(2):79-83. doi: 10.1177/107110079501600205. PMID: 7767451.
1280. Tsai JC, Chan P, Wang CH, Jeng C, Hsieh MH, Kao PF, Chen YJ, Liu JC. The effects of exercise training on walking function and perception of health status in elderly patients with peripheral arterial occlusive disease. Journal of Internal Medicine 2002 Nov;252(5):448-455. doi: 10.1046/j.1365-2796.2002.01055.x. PMID: 12528763.
1281. Tsai Y-C, Liu C-H. The effectiveness of pelvic floor exercises, digital vaginal palpation and interpersonal support on stress urinary incontinence: an experimental study. International Journal of Nursing Studies 2009 Sep;46(9):1181-1186. doi: 10.1016/j.ijnurstu.2009.03.003. PMID: 19361800.
1282. Tsuyuki K, Kimura Y, Chiashi K, Matsushita C, Ninomiya K, Choh K, Hase H, Dohi S. Oxygen uptake efficiency slope as monitoring tool for physical training in chronic hemodialysis patients. Therapeutic Apheresis and Dialysis 2003 Aug;7(4):461-467. doi: 10.1046/j.1526-0968.2003.00084.x. PMID: 12887732.
1283. Tuomilehto J, Lindstrom J, Eriksson JG, Valle TT, Hamalainen H, Ilanne-Parikka P, Keinanen-Kiukaanniemi S, Laakso M, Louheranta A, Rastas M, Salminen V, Uusitupa M, Finnish Diabetes Prevention Study G. Prevention of type 2 diabetes mellitus by changes in lifestyle among subjects with impaired glucose tolerance. The New England Journal of Medicine 2001 May 3;344(18):1343-1350. doi: 10.1056/NEJM200105033441801. PMID: 11333990.
1284. Turner JA. Comparison of group progressive-relaxation training and cognitive-behavioral group therapy for chronic low back pain. Journal of Consulting and Clinical Psychology 1982 Oct;50(5):757-765. PMID: 6216275.
1285. Turner JA, Clancy S. Comparison of operant behavioral and cognitive-behavioral group treatment for chronic low back pain. Journal of Consulting and Clinical Psychology 1988 Apr;56(2):261-266. doi: 10.1037/0022-006X.56.2.261. PMID: 2967314.
1286. Turner JA, Clancy S, McQuade KJ, Cardenas DD. Effectiveness of behavioral therapy for chronic low back pain: a component analysis. Journal of Consulting and Clinical Psychology 1990 Oct;58(5):573-579. doi: 10.1037/0022-006X.58.5.573. PMID: 2147702.
1287. Turner JA, Jensen MP. Efficacy of cognitive therapy for chronic low back pain. Pain 1993 Feb;52(2):169-177. PMID: 8455964.
1288. Turner S, Eastwood P, Cook A, Jenkins S. Improvements in symptoms and quality of life following exercise training in older adults with moderate/severe persistent asthma. Respiration 2011 Mar;81(4):302-310. doi: 10.1159/000315142. PMID: 20501982.
1289. UK BEAM Trial Team. United Kingdom back pain exercise and manipulation (UK BEAM) randomised trial: effectiveness of physical treatments for back pain in primary care. BMJ 2004 Dec 11;329(7479):1377-1384. doi: 10.1136/bmj.38282.669225.AE. PMID: 15556955.
1290. Unlu Z, Tasci S, Tarhan S, Pabuscu Y, Islak S. Comparison of 3 physical therapy modalities for acute pain in lumbar disc herniation measured by clinical evaluation and magnetic resonance imaging. Journal of Manipulative and Physiological Therapeutics 2008 Mar;31(3):191-198. doi: 10.1016/j.jmpt.2008.02.001. PMID: 18394495.
1291. Urbscheit NL, Wiegand MR. Effect of two exercise programs on balance scores in elderly ambulatory people. Physical & Occupational Therapy in Geriatrics 2001;19(4):49-58. doi: 10.1080/J148v19n04_04.
1292. Uusi-Rasi K, Kannus P, Cheng S, Sievanen H, Pasanen M, Heinonen A, Nenonen A, Halleen J, Fuerst T, Genant H, Vuori I. Effect of alendronate and exercise on bone and physical performance of postmenopausal women: a randomized controlled trial. Bone 2003 Jul;33(1):132-143. doi: 10.1016/S8756-3282(03)00082-6. PMID: 12919708.
1293. Vadiraja HS, Rao MR, Nagarathna R, Nagendra HR, Rekha M, Vanitha N, Gopinath KS, Srinath BS, Vishweshwara MS, Madhavi YS, Ajaikumar BS, Bilimagga SR, Rao N. Effects of yoga program on quality of life and affect in early breast cancer patients undergoing adjuvant radiotherapy: a randomized controlled trial. Complementary Therapies in Medicine 2009 Oct-Dec;17(5-6):274-280. doi: 10.1016/j.ctim.2009.06.004. PMID: 19942107.
1294. Vadiraja HS, Rao RM, Hongasandra NR, Nagarathna R, Rekha M, Vanitha N, Kodaganuru GS, Srinath BS, Vishweshwara MS, Madhavi YS, Basavalingaiah AS, Bilimagga RS, Rao N. Effects of yoga on symptom management in breast cancer patients: a randomized controlled trial. International Journal of Yoga 2009 Jul;2(2):73-79. doi: 10.4103/0973-6131.60048. PMID: 20842268.
1295. Vaile J, Halson S, Gill N, Dawson B. Effect of hydrotherapy on the signs and symptoms of delayed onset muscle soreness. European Journal of Applied Physiology 2008 Mar;102(4):447-455. doi: 10.1007/s00421-007-0605-6. PMID: 17978833.
1296. Valkeinen H, Alen M, Hannonen P, Hakkinen A, Airaksinen O, Hakkinen K. Changes in knee extension and flexion force, EMG and functional capacity during strength training in older females with fibromyalgia and healthy controls. Rheumatology 2004 Feb;43(2):225-228. doi: 10.1093/rheumatology/keh027. PMID: 13130154.
1297. Vallbona C, Hazlewood CF, Jurida G. Response of pain to static magnetic fields in postpolio patients: a double-blind pilot study. Archives of Physical Medicine and Rehabilitation 1997 Nov;78(11):1200-1203. doi: 10.1016/S0003-9993(97)90332-4. PMID: 9365349.
1298. van Baar ME, Dekker J, Oostendorp RA, Bijl D, Voorn TB, Lemmens JA, Bijlsma JW. The effectiveness of exercise therapy in patients with osteoarthritis of the hip or knee: a randomized clinical trial. The Journal of Rheumatology 1998 Dec;25(12):2432-2439. PMID: 9858441.
1299. van de Port IG, Wevers LE, Lindeman E, Kwakkel G. Effects of circuit training as alternative to usual physiotherapy after stroke: randomised controlled trial. BMJ 2012 May 10;344:e2672. doi: 10.1136/bmj.e2672. PMID: 22577186.
1300. van den Hout JH, Vlaeyen JW, Heuts PH, Zijlema JH, Wijnen JA. Secondary prevention of work-related disability in nonspecific low back pain: does problem-solving therapy help? A randomized clinical trial. The Clinical Journal of Pain 2003 Mar-Apr;19(2):87-96. PMID: 12616178.
1301. van der Heijden GJM, Beurskens AJH, Dirx MJM, Bouter LM, Lindeman E. Efficacy of lumbar traction: a randomised clinical trial. Physiotherapy 1995 Jan;81(1):29-35. doi: 10.1016/S0031-9406(05)67032-0.
1302. van der Kooi EL, Vogels OJ, van Asseldonk RJ, Lindeman E, Hendriks JC, Wohlgemuth M, van der Maarel SM, Padberg GW. Strength training and albuterol in facioscapulohumeral muscular dystrophy. Neurology 2004 Aug 24;63(4):702-708. doi: 10.1212/01.WNL.0000134660.30793.1F. PMID: 15326246.
1303. van der Roer N, van Tulder M, Barendse J, Knol D, van Mechelen W, de Vet H. Intensive group training protocol versus guideline physiotherapy for patients with chronic low back pain: a randomised controlled trial. European Spine Journal 2008 Sep;17(9):1193-1200. doi: 10.1007/s00586-008-0718-6. PMID: 18663487.
1304. van Gestel AJR, Kohler M, Steier J, Teschler S, Russi EW, Teschler H. The effects of controlled breathing during pulmonary rehabilitation in patients with COPD. Respiration 2012 Jan;83(2):115-124. doi: 10.1159/000324449. PMID: 21474911.
1305. van Houtte S, Vanlandewijck Y, Kiekens C, Spengler CM, Gosselink R. Patients with acute spinal cord injury benefit from normocapnic hyperpnoea training. Journal of Rehabilitation Medicine 2008 Feb;40(2):119-125. doi: 10.2340/16501977-0140. PMID: 18509576.
1306. van Kampen M, de Weerdt W, van Poppel H, de Ridder D, Feys H, Baert L. Effect of pelvic-floor re-education on duration and degree of incontinence after radical prostatectomy: a randomised controlled trial. Lancet 2000;355:98-102. doi: 10.1016/S0140-6736(99)03473-X. PMID: 10675166.
1307. van Laarhoven CJ, Meeuwis JD, van der Werken C. Postoperative treatment of internally fixed ankle fractures: a prospective randomised study. Journal of Bone and Joint Surgery -- British Volume 1996 May;78-B(3):395-399. PMID: 8636173.
1308. van Mechelen W, Hlobil H, Kemper HC, Voorn WJ, de Jongh HR. Prevention of running injuries by warm-up, cool-down, and stretching exercises. The American Journal of Sports Medicine 1993 Sep-Oct;21(5):711-719. doi: 10.1177/036354659302100513. PMID: 8238713.
1309. van Poppel MNM, Koes BW, van der Ploeg T, Smid T, Bouter LM. Lumbar supports and education for the prevention of low back pain in industry: a randomized controlled trial. JAMA 1998 Jun 10;279(22):1789-1794. doi: 10.1001/jama.279.22.1789. PMID: 9628709.
1310. van Schalkwyk R, Parkin-Smith GF. A clinical trial investigating the possible effect of the supine cervical rotatory manipulation and the supine lateral break manipulation in the treatment of mechanical neck pain: a pilot study. Journal of Manipulative and Physiological Therapeutics 2000 Jun;23(5):324-331. doi: 10.1067/mint.2000.106868. PMID: 10863252.
1311. van Tiggelen D, Wickes S, Coorevits P, Dumalin M, Witvrouw E. Sock systems to prevent foot blisters and the impact on overuse injuries of the knee joint. Military Medicine 2009 Feb;174(2):183-189. PMID: 19317200.
1312. van Tiggelen D, Witvrouw E, Roget P, Cambier D, Danneels L, Verdonk R. Effect of bracing on the prevention of anterior knee pain -- a prospective randomized study. Knee Surgery, Sports Traumatology, Arthroscopy 2004;12(5):434-439. doi: 10.1007/s00167-003-0479-z. PMID: 15064923.
1313. van Veldhoven NHMJ, Vermeer A, Bogaard JM, Hessels MGP, Wijnroks L, Colland VT, van Essen-Zandvliet EEM. Children with asthma and physical exercise: effects of an exercise programme. Clinical Rehabilitation 2001 Aug;15(4):360-370. doi: 10.1191/026921501678310162. PMID: 11518437.
1314. van Vilsteren MC, de Greef MH, Huisman RM. The effects of a low-to-moderate intensity pre-conditioning exercise programme linked with exercise counselling for sedentary haemodialysis patients in The Netherlands: results of a randomized clinical trial. Nephrology, Dialysis, Transplantation 2005 Jan;20(1):141-146. doi: 10.1093/ndt/gfh560. PMID: 15522901.
1315. van Wetering CR, Hoogendoorn M, Mol SM, Rutten-van Molken MP, Schols AM. Short- and long-term efficacy of a community-based COPD management program in less advanced COPD: a randomized controlled trial. Thorax 2010 Jan;65(1):7-13. doi: 10.1136/thx.2009.118620. PMID: 19703824.
1316. Veale D, Le Fevre K, Pantelis C, de Souza V, Mann A, Sargeant A. Aerobic exercise in the adjunctive treatment of depression: a randomized controlled trial. Journal of the Royal Society of Medicine 1992 Sep;85(9):541-544. PMID: 1433121.
1317. Vedanthan PK, Kesavalu LN, Murthy KC, Duvall K, Hall MJ, Baker S, Nagarathna S. Clinical study of yoga techniques in university students with asthma: a controlled study. Allergy and Asthma Proceedings 1998 Jan-Feb;19(1):3-9. doi: 10.2500/108854198778557971. PMID: 9532318.
1318. Velikonja O, Curic K, Ozura A, Jazbec SS. Influence of sports climbing and yoga on spasticity, cognitive function, mood and fatigue in patients with multiple sclerosis. Clinical Neurology and Neurosurgery 2010 Sep;112(7):597-601. doi: 10.1016/j.clineuro.2010.03.006. PMID: 20371148.
1319. Vempati R, Bijlani RL, Deepak KK. The efficacy of a comprehensive lifestyle modification programme based on yoga in the management of bronchial asthma: a randomized controlled trial. BMC Pulmonary Medicine 2009 Jul 30;9(37):Epub. doi: 10.1186/1471-2466-9-37. PMID: 19643002.
1320. Venzke L, Calvert JF Jr, Gilbertson B. A randomized trial of acupuncture for vasomotor symptoms in post-menopausal women. Complementary Therapies in Medicine 2010 Apr;18(2):59-66. doi: 10.1016/j.ctim.2010.02.002. PMID: 20430288.
1321. Verschueren SMP, Roelants M, Delecluse C, Swinnen S, Vanderschueren D, Boonen S. Effect of 6-month whole body vibration training on hip density, muscle strength, and postural control in postmenopausal women: a randomized controlled pilot study. Journal of Bone and Mineral Research 2004 Mar;19(3):352-359. doi: 10.1359/JBMR.0301245. PMID: 15040822.
1322. Victor CR, Triggs E, Ross F, Lord J, Axford JS. Lack of benefit of a primary care-based nurse-led education programme for people with osteoarthritis of the knee. Clinical Rheumatology 2005 Aug;24(4):358-364. doi: 10.1007/s10067-004-1001-9. PMID: 15937632.
1323. Vince KG, Kelly MA, Beck J, Insall JN. Continuous passive motion after total knee arthroplasty. The Journal of Arthroplasty 1987;2(4):281-284. PMID: 3430154.
1324. Vincent A, Barton DL, Mandrekar JN, Cha SS, Zais T, Wahner-Roedler DL, Keppler MA, Kreitzer MJ, Loprinzi C. Acupuncture for hot flashes: a randomized, sham-controlled clinical study. Menopause 2007 Jan-Feb;14(1):45-52. doi: 10.1097/01.gme.0000227854.27603.7d. PMID: 17019380.
1325. Vinter CA, Jensen DM, Ovesen P, Beck-Nielsen H, Tanvig M, Lamont RF, Jorgensen JS. Postpartum weight retention and breastfeeding among obese women from the randomized controlled Lifestyle in Pregnancy (LiP) trial. Acta Obstetricia et Gynecologica Scandinavica 2014 Aug;93(8):794-801. doi: 10.1111/aogs.12429. PMID: 24834792.
1326. Vioreanu M, Dudeney S, Hurson B, Kelly E, O'Rourke K, Quinlan W. Early mobilization in a removable cast compared with immobilization in a cast after operative treatment of ankle fractures: a prospective randomized study. Foot & Ankle International 2007 Jan;28(1):13-19. doi: 10.3113/FAI.2007.0003. PMID: 17257532.
1327. Vogiatzis I, Nanas S, Roussos C. Interval training as an alternative modality to continuous exercise in patients with COPD. The European Respiratory Journal 2002 Jul;20(1):12-19. doi: 10.1183/09031936.02.01152001. PMID: 12166558.
1328. Vogiatzis I, Terzis G, Nanas S, Stratakos G, Simoes DCM, Georgiadou O, Zakynthinos S, Roussos C. Skeletal muscle adaptations to interval training in patients with advanced COPD. Chest 2005 Dec;128(6):3838-3845. doi: 10.1378/chest.128.6.3838. PMID: 16354852.
1329. von Korff M, Balderson BH, Saunders K, Miglioretti DL, Lin EH, Berry S, Moore JE, Turner JA. A trial of an activating intervention for chronic back pain in primary care and physical therapy settings. Pain 2005 Feb;113(3):323-330. doi: 10.1016/j.pain.2004.11.007. PMID: 15661440.
1330. von Piekartz H, Ludtke K. Effect of treatment of temporomandibular disorders (TMD) in patients with cervicogenic headache: a single-blind, randomized controlled study. Cranio 2011 Jan;29(1):43-56. PMID: 21370769.
1331. von Stengel S, Kemmler W, Mayer S, Engelke K, Klarner A, Kalender WA. Effekte eines ganzkorpervibrationstrainings auf parameter des frakturrisikos. Einjahres-ergebnisse der randomisierten kontrollierten ELVIS-studie (Effect of whole body vibration exercise on osteoporotic risk factors. Results of the controlled randomized longitudinal ELVIS study after one year) [German]. Deutsche Medizinische Wochenschrift 2009 Jul;134(30):1511-1516. doi: 10.1055/s-0029-1233971. PMID: 19603365.
1332. Vroomen PC, de Krom MC, Wilmink JT, Kester AD, Knottnerus JA. Lack of effectiveness of bed rest for sciatica. The New England Journal of Medicine 1999 Feb 11;340(6):418-423. doi: 10.1056/NEJM199902113400602. PMID: 9971865.
1333. Waagen GN, Haldeman S, Cook G, Lopez D, de Boer KF. Short term trial of chiropractic adjustments for the relief of chronic low back pain. Manual Medicine 1986;2:63-67.
1334. Wadell K, Sundelin G, Henriksson-Larsen K, Lundgren R. High intensity physical group training in water -- an effective training modality for patients with COPD. Respiratory Medicine 2004 May;98(5):428-438. doi: 10.1016/j.rmed.2003.11.010. PMID: 15139572.
1335. Wakabayashi R, Motegi T, Yamada K, Ishii T, Jones RC, Hyland ME, Gemma A, Kida K. Efficient integrated education for older patients with chronic obstructive pulmonary disease using the Lung Information Needs Questionnaire. Geriatrics & Gerontology International 2011 Oct;11(4):422-430. doi: 10.1111/j.1447-0594.2011.00696.x. PMID: 21447136.
1336. Walker L, Svenkerud T, Weber H. Traksjonsbehandling ved lumbago-ischias [Norwegian]. Fysioterapeuten 1982 Apr;49:161-163,177.
1337. Walker RH, Morris BA, Angulo DL, Schneider J, Colwell CW Jr. Postoperative use of continuous passive motion, transcutaneous electrical nerve stimulation, and continuous cooling pad following total knee arthroplasty. The Journal of Arthroplasty 1991 Jun;6(2):151-156. PMID: 1875206.
1338. Walker WC, Metzler M, Cifu DX, Swartz Z. Neutral wrist splinting in carpal tunnel syndrome: a comparison of night-only versus full-time wear instructions. Archives of Physical Medicine and Rehabilitation 2000 Apr;81(4):424-429. doi: 10.1053/mr.2000.3856. PMID: 10768530.
1339. Walter HJ, Hofman A, Vaughan RD, Wynder EL. Modification of risk factors for coronary heart disease. Five-year results of a school-based intervention trial. The New England Journal of Medicine 1988 Apr 28;318(17):1093-1100. doi: 10.1056/NEJM198804283181704. PMID: 3281016.
1340. Wand BM, Bird C, McAuley JH, Dore CJ, MacDowell M, de Souza LH. Early intervention for the management of acute low back pain: a single-blind randomized controlled trial of biopsychosocial education, manual therapy, and exercise. Spine 2004 Nov 1;29(21):2350-2356. PMID: 15507794.
1341. Wang AC, Wang YY, Chen MC. Single-blind, randomized trial of pelvic floor muscle training, biofeedback-assisted pelvic floor muscle training, and electrical stimulation in the management of overactive bladder. Urology 2004 Jan;63(1):61-66. doi: 10.1016/j.urology.2003.08.047. PMID: 14751349.
1342. Wang CJ, Liu HC, Fu TH. The effects of extracorporeal shock wave on acute high-energy long bone fractures of the lower extremity. Archives of Orthopaedic and Trauma Surgery 2007 Feb;127(2):137-142. doi: 10.1007/s00402-006-0236-0. PMID: 17053946.
1343. Wang C-L, Liang L, Fu J-F, Zou C-C, Hong F, Xue J-Z, Lu J-R, Wu X-M. Effect of lifestyle intervention on non-alcoholic fatty liver disease in Chinese obese children. World Journal of Gastroenterology 2008 Mar 14;14(10):1598-1602. doi: 10.3748/wjg.14.1598. PMID: 18330955.
1344. Wang G, Qu F. Treatment of 482 cases of cervical spondylopathy by combining point-injection and needle-warming via moxibustion. Chung I Tsa Chih Ying Wen Pan [Journal of Traditional Chinese Medicine] 2001 Mar;21(1):31-33. PMID: 11360535.
1345. Wang J-S, Hung W-P. The effects of a swimming intervention for children with asthma. Respirology 2009 Aug;14(6):838-842. doi: 10.1111/j.1440-1843.2009.01567.x. PMID: 19703065.
1346. Wang LY, Gutin B, Barbeau P, Moore JB, Hanes J Jr, Johnson MH, Cavnar M, Thornburg J, Yin Z. Cost-effectiveness of a school-based obesity prevention program. The Journal of School Health 2008 Dec;78(12):619-624. doi: 10.1111/j.1746-1561.2008.00357.x. PMID: 19000237.
1347. Wang S-M, de Zinno P, Lin EC, Lin H, Yue JJ, Berman MR, Braveman F, Kain ZN. Auricular acupuncture as a treatment for pregnant women who have low back and posterior pelvic pain: a pilot study. American Journal of Obstetrics and Gynecology 2009 Sep;201(3):271.e271-271.e279. doi: 10.1016/j.ajog.2009.04.028. PMID: 19560110.
1348. Wang T-J, Lee S-C, Liang S-Y, Tung H-H, Wu S-FV, Lin Y-P. Comparing the efficacy of aquatic exercises and land-based exercises for patients with knee osteoarthritis. Journal of Clinical Nursing 2011 Sep;20(17-18):2609-2622. doi: 10.1111/j.1365-2702.2010.03675.x. PMID: 21539629.
1349. Wang X-H, Zhang L-M, Han M, Zhang K-Q. (Clinical application of functional exercise and staged therapy in treatment of facial nerve paralysis) [Chinese - simplified characters]. Zhongguo Linchuang Kangfu [Chinese Journal of Clinical Rehabilitation] 2004 Feb 5;8(4):616-617.
1350. Wang Y-J, Boehmke M, Wu Y-W, Dickerson SS, Fisher N. Effects of a 6-week walking program on Taiwanese women newly diagnosed with early-stage breast cancer. Cancer Nursing 2011 Mar-Apr;34(2):E1-E13. doi: 10.1097/NCC.0b013e3181e4588d. PMID: 20697267.
1351. Warming S, Ebbehoj NE, Wiese N, Larsen LH, Duckert J, Tonnesen H. Little effect of transfer technique instruction and physical fitness training in reducing low back pain among nurses: a cluster randomised intervention study. Ergonomics 2008;51(10):1530-1548. doi: 10.1080/00140130802238606. PMID: 18803093.
[truncated: 24,625 more chars]
